# Global Case Fatality of Bacterial Meningitis During an 80-Year Period: A Systematic Review and Meta-Analysis

**DOI:** 10.1001/jamanetworkopen.2024.24802

**Published:** 2024-08-02

**Authors:** Cornelis N. van Ettekoven, Fabian D. Liechti, Matthijs C. Brouwer, Merijn W. Bijlsma, Diederik van de Beek

**Affiliations:** 1 Department of Neurology, Amsterdam Neuroscience, Amsterdam UMC, University of Amsterdam, Amsterdam, The Netherlands; 2 Department of Neurology, HagaZiekenhuis, The Hague, the Netherlands; 3 Department of General Internal Medicine, Inselspital, Bern University Hospital, University of Bern, Bern, Switzerland; 4 Department of Pediatrics, Amsterdam Neuroscience, Amsterdam, the Netherlands

## Abstract

**Importance:**

The impact of vaccination, antibiotics, and anti-inflammatory treatment on pathogen distribution and outcome of bacterial meningitis over the past century is uncertain.

**Objective:**

To describe worldwide pathogen distribution and case fatality ratios of community-acquired bacterial meningitis.

**Data Sources:**

Google Scholar and MEDLINE were searched in January 2022 using the search terms *bacterial meningitis* and *mortality*.

**Study Selection:**

Included studies reported at least 10 patients with bacterial meningitis and survival status. Studies that selected participants by a specific risk factor, had a mean observation period before 1940, or had more than 10% of patients with health care–associated meningitis, tuberculous meningitis, or missing outcome were excluded.

**Data Extraction and Synthesis:**

Data were extracted by 1 author and verified by a second author. The study followed the Preferred Reporting Items for Systematic Reviews and Meta-Analyses. Random-effects models stratified by age (ie, neonates, children, adults), Human Development Index (ie, low-income or high-income countries), and decade and meta-regression using the study period’s year as an estimator variable were used.

**Main Outcome and Measure:**

Case fatality ratios of bacterial meningitis.

**Results:**

This review included 371 studies performed in 108 countries from January 1, 1935, to December 31, 2019, describing 157 656 episodes. Of the 33 295 episodes for which the patients’ sex was reported, 13 452 (40%) occurred in females. Causative pathogens were reported in 104 598 episodes with *Neisseria meningitidis* in 26 344 (25%) episodes, *Streptococcus pneumoniae* in 26 035 (25%) episodes, *Haemophilus influenzae* in 22 722 (22%), other bacteria in 19 161 (18%) episodes, and unidentified pathogen in 10 336 (10%) episodes. The overall case fatality ratio was 18% (95% CI, 16%-19%), decreasing from 32% (95% CI, 24%-40%) before 1961 to 15% (95% CI, 12%-19%) after 2010. It was highest in meningitis caused by *Listeria monocytogenes* at 27% (95% CI, 24%-31%) and pneumococci at 24% (95% CI, 22%-26%), compared with meningitis caused by meningococci at 9% (95% CI, 8%-10%) or *H influenzae* at 11% (95% CI, 10%-13%). Meta-regression showed decreasing case fatality ratios overall and stratified by *S pneumoniae, Escherichia coli*, or *Streptococcus agalactiae* (*P* < .001).

**Conclusions and Relevance:**

In this meta-analysis with meta-regression, declining case fatality ratios of community-acquired bacterial meningitis throughout the last century were observed, but a high burden of disease remained.

## Key Points

**Question:**

What is the case fatality ratio (CFR) of bacterial meningitis and how has it changed throughout the last 80 years?

**Findings:**

In this systematic review and meta-analysis of 371 studies and 157 656 episodes, there was an overall CFR of 18%, decreasing from 32% before 1961 to 15% after 2010. Decreasing CFRs were also observed in stratified analyses of *S pneumoniae, E coli*, and *S agalactiae* meningitis.

**Meaning:**

These findings suggest that worldwide trends in decreasing overall CFRs were accounted for by declining CFRs in pneumococcal meningitis.

## Teaser

This systematic review and meta-analysis examines the worldwide pathogen distribution and case fatality ratios of community-acquired bacterial meningitis from 1935 to 2022.

## Introduction

The causative pathogens and the prognosis of community-acquired bacterial meningitis have changed throughout the last century.^1^ The distribution of pathogens causing community-acquired bacterial meningitis has changed over time with profound impact of conjugate vaccines against the most common bacteria.^2,3^ Worldwide implementation of conjugate vaccines for children against *Haemophilus influenzae* type B, specific serogroups of *Neisseria meningitidis,* and serotypes of *Streptococcus pneumoniae* have reduced the incidence of bacterial meningitis in both high-income and lower-income settings.^4^ Likewise, bacterial meningitis, which used to be uniformly lethal, has developed into a treatable disease after the introduction of antisera and antibiotics.^2^ Modern medical care, including intensive care and the introduction of the adjunctive anti-inflammatory treatment dexamethasone, has further improved outcomes for a subset of patients in higher income countries and meningitis due to several pathogens.^5,6,7,8,9,10,11,12^ The impact of these therapeutic and preventive measurements has been evaluated in randomized clinical trials, meta-analyses, and cohort studies, but the overall impact on pathogen distribution and outcomes in bacterial meningitis remains uncertain.^4,11,13,14^ We performed a systematic review and meta-analysis with meta-regression of studies on community-acquired bacterial meningitis throughout an 80-year period to describe temporal trends of pathogen distribution and case fatality ratios (CFR) overall and for specific age groups and pathogens.

## Methods

This systematic review and meta-analysis followed the Preferred Reporting Items for Systematic Reviews and Meta-analyses (PRISMA) reporting guideline and Meta-analysis of Observational Studies in Epidemiology (MOOSE) reporting guideline.^15^ We searched Google Scholar and MEDLINE (via PubMed.org, last search on January 1, 2022) (eTable 1 in Supplement 1) and included studies on community-acquired bacterial meningitis with mean study period after 1940. Duplicates were removed using Rayyan and Endnote version 21.0.1 (Clarivate) before an author (C.N.v.E.) screened titles and abstracts.^16^ Full texts were then assessed and if necessary, discussed with a second author (F.D.L.) who also confirmed inclusion of all studies.

### Statistical Analysis

Studies were included if 10 or more patients with at least CFR were described. Meningitis was defined according to the studies’ authors with patients having at least characteristic clinical symptoms. Studies reporting only on cohorts of patients selected by a specific risk factor (eg, immunosuppression) or disease severity (eg, admission to intensive care) were excluded. Studies were excluded if they included at least 10% of patients with health care–associated meningitis, ventriculitis, tuberculous meningitis, or missing outcomes.^17,18,19^ We only considered the largest or latest publication for studies with overlapping patient populations (eg, in time and region). Due to the severity of the disease, we expected that most patients would seek medical advice and mortality would usually occur during hospitalization. Because our aim was to analyze crude case fatality from diverse settings throughout an 80-year span, we did not do formal bias assessment and addressed heterogeneity via subgroup and meta-regression analyses. We checked for small-study effects and publication bias by visual inspection of funnel plots. Studies reporting only on specific pathogens were included in the analyses for the specific pathogens, but not the main analysis.

One author (C.N.v.E.) extracted data on main outcome, study design, inclusion criteria and period, age, sex, country, and causative pathogens according to a nonregistered protocol. A second author (F.D.L.) validated database entries by cross-checking all total CFRs and reviewing all parameters with identified inconsistencies and outliers. When more than 1 inclusion period or age group was described in a study, the sample was split into study periods (k); however, in some cases not all information was given for all participants on an individual level and the total number of participants for the pathogens combined may differ from the total number of patients included in the overall analysis of the same age group. If no inclusion period was provided, we used the publication year instead. We used logit transformation before applying random-effects meta-analysis models.^20^ We performed separate analyses according to age groups (neonates aged younger than 2 months [regardless of gestational age at birth]; children aged 2 months to 16 years; adults aged older than 16 years), and Human Development Index (HDI) in the year 2018 (low-income countries, less than 0.7; high-income countries, 0.7 or more) and show 95% prediction intervals (PIs).^21,22^ We performed separate analyses for the 3 most prevalent pathogens from each age group. If less than 3 studies were available, we relinquished to present pooled effect estimates. In the meta-regression model, we used the observation period’s mean year as the estimator variable.^20^ We used R version 4.2.1 (R Project for Statistical Computing) for all analyses (eTables 2 and 3 in Supplement 1). Data were collected between September 5, 2018, and January 31, 2024, and analyzed from January to May 2024.

## Results

We retrieved 371 studies with 427 study periods performed from January 1, 1935, to December 31, 2019, in 108 countries, describing 157 656 episodes of community-acquired bacterial meningitis.^5,23,24,25,26,27,28,29,30,31,32,33,34,35,36,37,38,39,40,41,42,43,44,45,46,47,48,49,50,51,52,53,54,55,56,57,58,59,60,61,62,63,64,65,66,67,68,69,70,71,72,73,74,75,76,77,78,79,80,81,82,83,84,85,86,87,88,89,90,91,92,93,94,95,96,97,98,99,100,101,102,103,104,105,106,107,108,109,110,111,112,113,114,115,116,117,118,119,120,121,122,123,124,125,126,127,128,129,130,131,132,133,134,135,136,137,138,139,140,141,142,143,144,145,146,147,148,149,150,151,152,153,154,155,156,157,158,159,160,161,162,163,164,165,166,167,168,169,170,171,172,173,174,175,176,177,178,179,180,181,182,183,184,185,186,187,188,189,190,191,192,193,194,195,196,197,198,199,200,201,202,203,204,205,206,207,208,209,210,211,212,213,214,215,216,217,218,219,220,221,222,223,224,225,226,227,228,229,230,231,232,233,234,235,236,237,238,239,240,241,242,243,244,245,246,247,248,249,250,251,252,253,254,255,256,257,258,259,260,261,262,263,264,265,266,267,268,269,270,271,272,273,274,275,276,277,278,279,280,281,282,283,284,285,286,287,288,289,290,291,292,293,294,295,296,297,298,299,300,301,302,303,304,305,306,307,308,309,310,311,312,313,314,315,316,317,318,319,320,321,322,323,324,325,326,327,328,329,330,331,332,333,334,335,336,337,338,339,340,341,342,343,344,345,346,347,348,349,350,351,352,353,354,355,356,357,358,359,360,361,362,363,364,365,366,367,368,369,370,371,372,373,374,375,376,377,378,379,380,381,382,383,384,385,386,387,388,389,390,391,392,393^ Of the 33 295 episodes for which the patients’ sex was reported, 13 452 (40%) occurred in females (Table 1 and eFigures 1 and 2 and eTable 4 in Supplement 1). The design was observational in 330 studies (94%) and interventional in 20 (6%) studies, with 12 to 22 831 (median [IQR], 115 [61-270]) episodes per study, and the inclusion period’s length varying from 3 months to 36 years (median [IQR], 5 [2-10] years). Studies included neonates (59 studies [16%]; 6549 episodes [4%]),^25,29,32,38,42,43,45,49,55,63,70,86,93,97,101,105,121,126,127,132,133,138,142,157,159,160,196,200,212,214,216,220,235,241,258,275,281,294,297,299,307,319,321,323,324,336,340,345,346,358,365,368,373,377,379,382,389,390,393^ children (101 studies [27%]; 21 511 episodes [14%]),^33,44,57,58,60,61,67,69,73,74,75,79,87,88,90,94,102,104,106,111,115,117,118,124,130,131,134,139,144,147,148,153,156,162,163,164,165,166,168,169,171,172,179,180,181,182,183,184,186,187,188,191,192,193,198,202,206,215,218,219,222,225,226,232,234,236,237,239,233,243,250,251,252,255,257,259,260,261,268,269,278,280,284,287,303,310,312,326,327,328,330,331,338,339,347,361,363,364,372,378,391^ adults (64 studies [17%]; 15 362 episodes [10%]),^64,84,103,109,123,136,145,146,149,161,178,185,195,197,204,207,209,210,211,213,223,229,231,242,244,245,246,249,253,256,265,267,272,276,279,285,289,290,292,296,301,304,306,309,316,320,332,337,342,348,350,353,354,357,362,366,369,371,381,383,384,386,388,392^ neonates and children (68 studies [18%]; 26 992 episodes [17%]),^24,34,40,46,48,56,62,68,78,85,92,95,96,107,108,110,112,114,116,121,122,125,128,137,140,141,152,154,158,167,170,175,176,177,194,201,203,205,217,221,238,240,247,249,263,264,266,274,282,286,288,293,298,300,315,317,318,325,329,333,341,343,344,352,374,375,376,385^ children and adults (19 studies [5%]; 3972 episodes [3%]), ^35,50,76,83,98,99,129,135,174,189,190,208,228,248,270,277,283,308,334,335^ or did not describe age groups (60 studies [16%]; 83 270 episodes [53%]).^23,26,27,28,30,31,36,37,39,41,47,51,52,53,54,59,65,66,71,72,77,80,81,82,89,91,100,113,119,143,150,151,155,173,199,224,227,230,254,262,271,273,291,295,302,305,311,313,314,322,349,352,355,356,359,360,367,370,380,387^

**Table 1.** Study Characteristics and Unadjusted Case Fatality Ratios in Bacterial Meningitis per Subgroup

Subgroup	Study periods, No.	No.		Case fatality ratio, % (95% CI)
		Episodes	Deaths	
All studies	427^a^	157 656	23 728	15.1 (14.9-15.2)
Age group				
Neonates	83	8708	1682	19.3 (18.4-20.2)
Children	134	33 747	4988	14.8 (14.4-15.2)
Adults	78	17 288	3339	19.3 (18.7-20.0)
Human Development Index				
High-income countries	285	98 152	13 457	13.7 (13.5-13.9)
Low-income countries	142	59 504	10 271	17.3 (16.9-17.6)
Mean observation period				
Before 1961	34	4887	1152	23.6 (22.2-24.9)
1961-1970	29	6010	1016	16.9 (15.9-17.9)
1971-1980	47	25 787	4477	17.4 (16.9-17.9)
1981-1990	92	18 946	2576	13.6 (13.1-14.1)
1991-2000	114	30 940	4977	16.1 (15.6-16.5)
2001-2010	73	58 158	7789	13.4 (13.1-13.7)
After 2010	38	12 928	1741	13.5 (12.8-14.1)

^a^ From 371 studies.

### Pathogen Distribution

**Figure 1.**
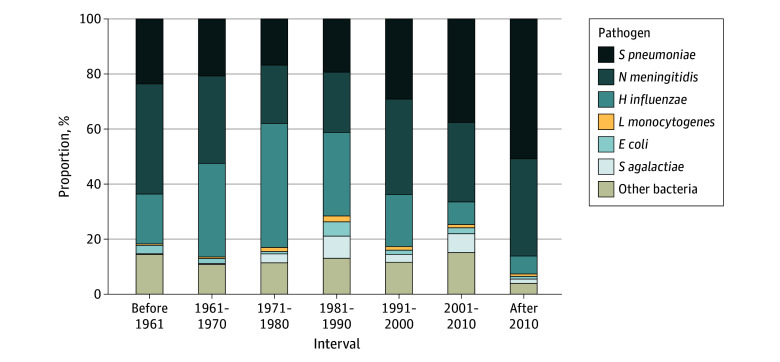
Causative Pathogens Proportions of identified causative pathogens per decade (interval indicates the study periods' mean observation year; before 1961: 3684 identified episodes; 1961-1970: 4082 identified episodes; 1971-1980: 22 533 identified episodes; 1981-1990: 15 739 identified episodes; 1991-2000: 17 567 identified episodes; 2001-2010: 24 305 identified episodes; after 2010: 6352 identified episodes.).

The causative pathogen was described in 104 598 of 157 656 episodes (66%). There were 26 344 episodes (25%) caused by *N meningitidis*, 26 035 (25%) by *Streptococcus pneumoniae,* 22 722 (22%) by *H influenzae*, 4293 (4%) by *Streptococcus agalactiae*, 2044 (2%) by *Escherichia coli,* 1265 (1%) by *Listeria monocytogenes*, 11 559 (11%) by other bacteria, and 10 336 (10%) by an unidentified pathogen. Before 1961, the pathogen was identified in 3684 of 4684 episodes (79%), whereas from 2001 to 2010, the pathogen was identified in 24 305 of 25 786 episodes (94%). The relative proportions and the number of episodes identified varied over time with increasing proportions for *S pneumoniae* and decreasing proportions for *H influenzae* (Figure 1 and eTable 5 in Supplement 1).

The causative pathogen varied according to age groups and over time (eTable 6 and eFigure 3 in Supplement 1). The most common pathogens among neonates were *S agalactiae* (2367 of 6896 episodes with an identified pathogen [34%]), *E coli* (162 of 6869 episodes [24%]) and *S pneumoniae* (403 of 6869 episodes [6%]). Among children aged 2 months to 16 years, the most frequent pathogens reported were *N meningitidis* (7315 of 25 480 episodes [31%]), *H influenzae* (7188 of 25 480 episodes [30%]), and *S pneumoniae* (6650 of 25 480 episodes [29%]). *S pneumoniae* became relatively more common in more recent years. In adults, the most frequent pathogens were *S pneumoniae* (3853 of 9386 episodes [45%]), *N meningitidis* (2224 of 9386 episodes [26%]), and *L monocytogenes* (394 of 9386 episodes [5%]), with no clear prevalence trends over time.

In high-income countries, the most common pathogens were *H influenzae* (18 357 of 68 085 episodes [27%]), *S pneumoniae* (16 529 of 68 085 episodes [27%]), and *N meningitidis* (16 362 of 68 085 episodes [24%]). The relative frequency of *S pneumoniae* increased since the 1980s, while the frequency of *H influenzae* decreased since the 1990s (eFigure 4 in Supplement 1). In low-income countries, the most frequent pathogens were *N meningitidis* (9982 of 26 177 episodes [38%]), *S pneumoniae* (9520 of 26 177 episodes [36%]), and *H influenzae* (4365 of 26 177 episodes [17%]). The relative frequency of *S pneumoniae* increased over time but the frequency of *H influenzae* decreased only after 2000.

### CFRs

**Figure 2.**
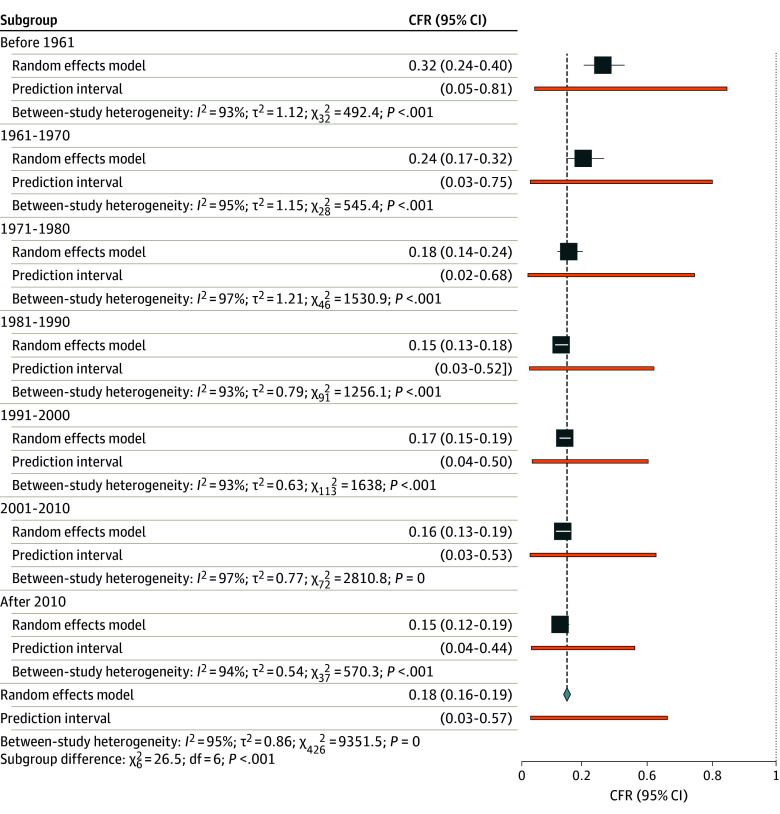
Meta-Analysis Results Case fatality ratios (CFR) in bacterial meningitis (Forest plot with individual studies suppressed) indicating the overall pooled estimate and the estimates of the time intervals as subgroups (before 1961: 34 study periods; 1961-1970, 29 study periods; 1971-1980: 47 study periods; 1981-1990: 92 study periods; 1991-2000: 114 study periods; 2001-2010: 73 study periods; after 2010: 38 study periods) with prediction intervals.

**Figure 3.**
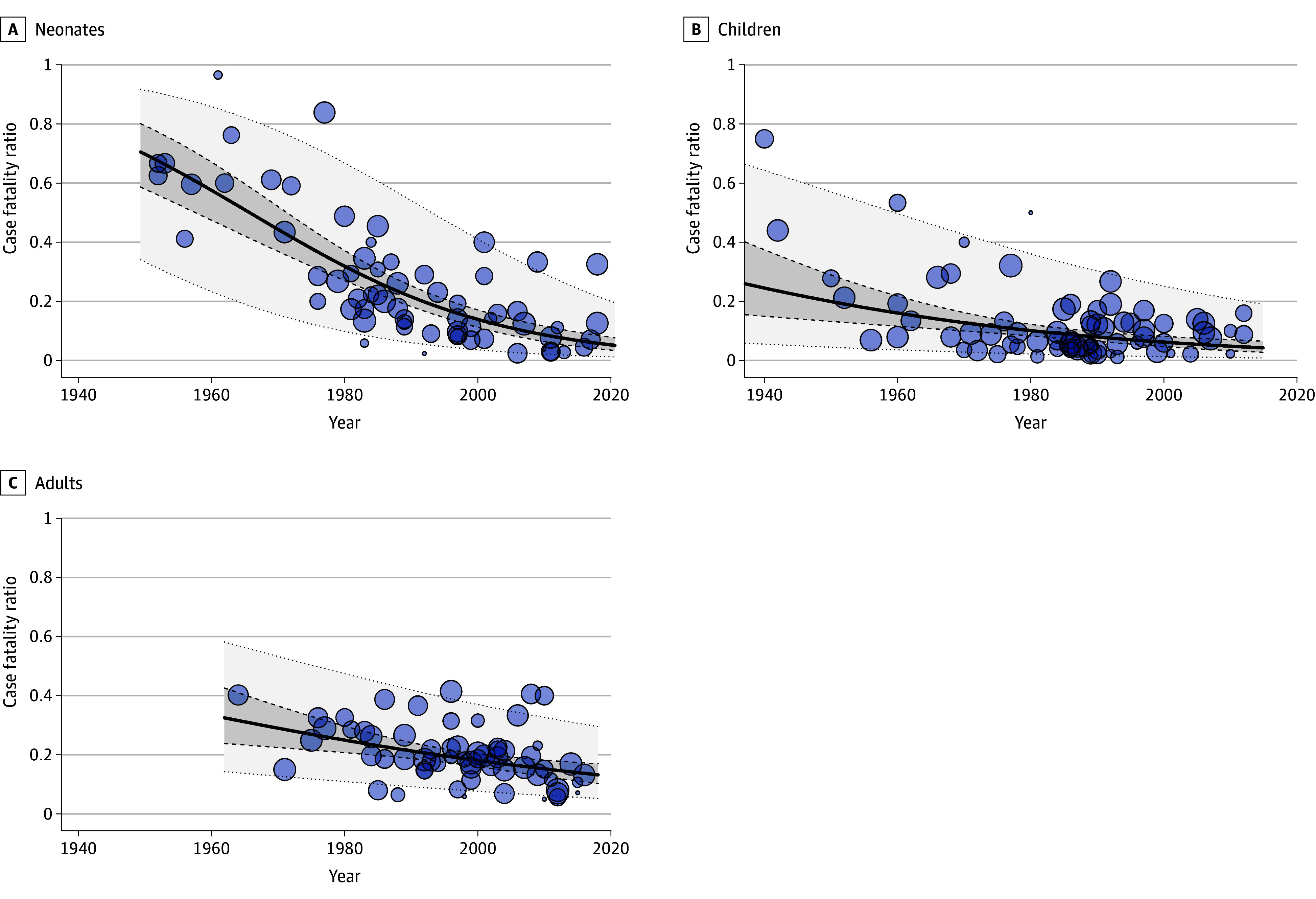
Meta-Regression Results Case fatality ratios of patients with bacterial meningitis using a meta-regression model with the studies’ mean observation year as estimator variable, stratified according to age groups in high-income countries (A, neonates, aged younger than 2 months; B, children, aged 2 months to 16 years; C, adults, aged older than 16 years). Solid lines are regression lines, dashed lines indicate 95% CI, dotted lines indicate 95% prediction interval. Circles indicate individual study periods with the circles' sizes corresponding to the model weights.

The CFR differed among countries and observation periods (eFigure 5 in Supplement 1). In the random-effects model, the overall CFR was 18% (95% CI, 16% to 19%) (Figure 2). Despite high heterogeneity we found no indication for small study effects or publication bias (eFigure 6 and 7 in Supplement 1). The CFR per subgroup was lowest in children of high-income countries and highest in neonates and adults of low-income countries (Table 2 and eFigures 8 to 13 in Supplement 1). Before 1961, CFR was 32% (95% CI, 24% to 40%) and the CFR was 15% (95% CI, 12% to 19%) after 2010. These results were confirmed in the meta-regression model with a decreasing CFR when including all studies (−1.5% per year; 95% CI, −2.0% to −0.9%; *P* < .001) (eFigure 14 and eTable 7 in Supplement 1) and in all of the subgroups (Figure 3), except for adults in low-income countries, where studies before the year 2000 are lacking (eFigure 15 in Supplement 1).

**Table 2.** Results of the Meta-Analyses of Case Fatality Ratios in Bacterial Meningitis Per Subgroup

Human Development Index	Study periods, No.	Case fatality ratio, % (95% CI)	*I^2^*, %
High and low-income countries, combined ages	427	17.7 (16.4-19.1)	95
High-income countries			
Neonates	18	34.7 (27.6-42.4)	79
Children	59	21.4 (18.5-24.7)	94
Adults	16	37.8 (29.5-46.8)	89
Low-income countries			
Neonates	65	22.1 (17.3-27.8)	92
Children	75	9.1 (7.4-11.2)	95
Adults	62	19.1 (16.9-21.6)	91

We found additional studies specifically reporting on *S pneumoniae* (113 studies; 24 553 episodes) (eTable 8 in Supplement 1),^394,395,396,397,398,399,400,401,402,403,404,405,406,407,408,409,410,411,412,413,414,415,416,417,418,419,420,421,422,423,424,425,426,427,428,429,430,431,432,433,434,435,436,437,438,439,440,441,442,443,444,445,446,447,448,449,450,451,452,453,454,455,456,457,458,459,460,461,462,463,464,465,466,467,468,469,470,471,472,473,474,475,476,477,478,479,480,481,482,483,484,485,486,487,488,489,490,491,492,493,494,495,496,497,498,499,500,501,502,503,504,505,506^ *N meningitidis* (75 studies; 100 278 episodes) (eTable 9 in Supplement 1),^396,496,507,508,509,510,511,512,513,514,515,516,517,518,519,520,521,522,523,524,525,526,527,528,529,530,531,532,533,534,535,536,537,538,539,540,541,542,543,544,545,546,547,548,549,550,551,552,553,554,555,556,557,558,559,560,561,562,563,564,565,566,567,568,569,570,571,572,573,574,575,576,577,578,579^ *H influenzae* (49 studies; 28 796 episodes) (eTable 10 in Supplement 1),^396,407,412,433,437,459,525,580,581,582,583,584,585,586,587,588,589,590,591,592,593,594,595,596,597,598,599,600,601,602,603,604,605,606,607,608,609,610,611,612,613,614,615,616,617,618,619,620,621^ *L monocytogenes* (15 studies; 985 episodes) (eTable 11 in Supplement 1),^407,489,622,623,624,625,626,627,628,629,630,631,632,633,634^ *E coli* (7 studies; 943 episodes) (eTable 12 in Supplement 1),^625,635,636,637,638,639,640^ and *S agalactiae* (14 studies; 1243 episodes) (eTable 13 in Supplement 1).^407,640,641,642,643,644,645,646,647,648,649,650,651,652^

*S pneumoniae* was among the most frequent pathogens in all 3 age groups (319 studies [86%]; 362 study periods [85%]; 46 597 episodes [30%]) (eTable 14 and eFigure 16 in Supplement 1). The overall CFR in the random-effects model was 24% (95% CI, 22% to 26%) (eFigure 17 in Supplement 1); CFRs decreased from 38% (95% CI, 29% to 47%) before 1961 to 19% (95% CI, 16% to 23%) in the 2010s. In all age-groups, CFRs were higher in low-income countries with highest CFRs in neonates of low-income countries (43%; 95% CI, 28% to 60%) and lowest CFRs in children of high-income countries (14%; 95% CI, 12% to 17%) (eTable 15 in Supplement 1). The overall CFR decreased (−1.7% per year; 95% CI, −2.3% to −1.1%; *P* < .001) (eFigure 18 in Supplement 1), an effect that was also significant in adults (−3.3% per year; 95% CI, −4.2% to −2.4% per year; *P* < .001) and children (−2.0% per year; 95% CI, −3.4% to −0.5% per year; *P* = .007) of high-income countries only and neonates of low-income countries only (−4.3% per year; 95% CI, −8.3% to −0.2% per year; *P* = .04) (eFigures 19 to 26 in Supplement 1).

*N meningitidis* was among the most frequent pathogens in children and adults (250 studies; 274 study periods; 123 830 episodes) (eTable 16 and eFigure 27 in Supplement 1).^23,24,25,26,27,28,30,31,33,34,35,36,37,39,40,41,44,45,47,48,50,51,52,53,54,58,59,60,61,62,64,65,66,67,71,72,77,78,81,82,83,84,85,86,87,88,89,93,94,95,96,98,99,102,103,104,108,109,111,112,113,114,115,118,119,121,122,123,124,125,129,130,131,134,135,141,143,144,147,148,149,150,152,154,155,161,163,164,165,166,167,169,170,172,173,177,182,183,185,186,187,188,189,190,198,199,200,201,205,208,211,216,217,219,221,223,225,227,229,230,237,238,240,244,247,248,251,255,260,261,262,265,266,268,270,271,273,278,280,281,282,286,287,288,291,293,295,296,298,300,302,304,310,311,322,328,329,333,336,337,344,351,352,358,360,361,372,374,375,376,380,381,384,387,393,396,496,507,508,509,510,511,512,513,514,515,516,517,518,519,520,521,522,523,524,525,526,527,528,529,530,531,532,533,534,535,536,537,538,539,540,541,542,543,544,545,546,547,548,549,550,551,552,553,554,555,556,557,558,559,560,561,562,563,564,565,566,567,568,569,570,571,572,573,574,575,576,577,578,579^ This sample was dominated by a Nigerian study describing an outbreak in 2009 with more than 50 000 patients.^575^ The overall CFR was 8.8% (95% CI, 8.0% to 9.7%) (eFigure 28 in Supplement 1); CFR was 11% (95% CI, 8% to 16%) before 1961 and 7.2% (95% CI, 5.8.% to 8.7%) in the 2000s. In both age-groups, CFRs were higher in low-income countries, with highest CFRs in adults of low-income countries (17%; 95% CI, 12% to 24%) and lowest CFRs in children of high-income countries (6.0%; 95% CI, 4.7% to 7.6%) (eTable 17 in Supplement 1). We found no evidence for a decrease in the overall CFR (−0.2% per year; 95% CI, −0.9 to 0.4 per year; *P* = .46) (eFigure 29 in Supplement 1) or in any of the subgroups (eFigures 30 to 31 in Supplement 1).

*H influenzae* was among the most frequent pathogens in children (226 of 630 studies [36%]; 249 of 733 study periods [34%]; 49 539 of 314 454 episodes [16%]) (eTable 18 and eFigure 32 in Supplement 1).^23,24,25,26,27,28,30,31,32,33,34,35,36,37,39,40,41,42,44,45,47,48,49,50,51,52,53,54,58,59,60,61,62,65,66,67,71,72,77,78,80,81,82,83,84,85,86,87,88,89,93,94,95,96,97,98,99,102,104,107,108,111,112,113,115,116,118,119,121,122,123,124,125,129,130,131,134,140,141,143,144,147,148,149,150,152,154,155,161,163,164,165,166,167,169,170,172,173,177,182,183,185,187,188,189,190,194,198,199,201,205,208,212,216,217,219,221,223,225,227,230,232,237,238,240,241,247,249,251,255,260,261,262,265,266,268,270,271,273,278,280,281,282,284,286,287,288,291,293,295,296,298,300,302,310,311,318,322,325,328,329,333,336,337,343,344,347,351,352,358,360,361,367,372,374,375,376,387,396,407,412,433,437,459,525,580,581,582,583,584,585,586,587,588,589,590,591,592,593,594,595,596,597,598,599,600,601,602,603,604,605,606,607,608,609,610,611,612,613,614,615,616,617,618,619,620,621^ The overall CFR was 11% (95% CI, 10% to 13%) (eFigure 33 in Supplement 1); CFR was 13% (95% CI, 10% to 18%) before 1961 and 10% (95% CI, 7% to 16%) after 2010. CFR was higher in low-income countries (23%; 95% CI, 19% to 29%) compared with high-income countries (7%; 95% CI, 5% to 9%) (eTable 19 in Supplement 1) and did not decrease substantially during the period (0.7% per year; 95% CI, −0.3% to 1.7% per year; *P* = .17) (eFigures 34 to 36 in Supplement 1).

*L monocytogenes* was among the most frequent pathogens in adults (70 of 630 studies [11%]; 77 of 733 study periods [11%]; 1829 of 314 454 episodes [0.6%]) (eTable 20 and eFigure 37 in Supplement 1).^23,25,31,37,38,45,47,49,53,62,64,65,70,71,81,84,86,89,97,103,119,120,121,122,123,132,143,149,161,177,185,189,201,208,209,212,220,223,230,235,248,273,304,311,333,336,337,342,352,358,359,360,384,387,393,407,489,622,623,624,625,626,627,628,629,630,631,632,633,634^ The overall CFR was 27% (95% CI, 24% to 31%) (eFigure 38 in Supplement 1); CFR was 35% (95% CI, 24% to 47%) before 1961 and 25% (95% CI, 20% to 30%) in the 2000s. An insufficient number of studies were available to analyze the CFR in adults of low-income countries (eTable 21 in Supplement 1). The analyses showed a decrease in the overall CFR during the study period (−1.1% per year; 95% CI, −2.2% to −0.1% per year; *P* = .03) (eFigures 39 to 41 in Supplement 1).

*E coli* was among the most frequent pathogens in neonates (90 of 630 studies [14%]; 100 of 733 study periods [14%]; 2137 of 314 454 episodes [1.5%]) (eTable 22 and eFigure 42 in Supplement 1).^23,24,25,30,31,32,33,35,37,38,40,42,44,45,49,53,55,62,63,64,65,70,71,84,86,93,95,96,97,102,103,108,111,115,120,121,122,127,132,133,141,142,143,147,148,149,152,164,169,177,185,187,190,200,201,208,210,212,216,217,220,223,229,230,235,241,244,249,260,261,281,293,300,312,333,336,337,344,347,352,358,367,393,625,635,636,637,638,639,640^ The overall CFR was 34% (95% CI, 28% to 41%) (eFigure 43 in Supplement 1); CFR was 63% (95% CI, 53% to 73%) before 1961 and 10% (95% CI, 8% to 12%) in the 2000s. CFR was higher in low-income countries (48%; 95% CI, 32% to 64%) compared with high-income countries (29%; 95% CI, 20% to 41%) (eTable 23 in Supplement 1) and overall decreasing (−5% per year; 95% CI, −6% to −4% per year; *P* < .001) (eFigures 44 to 46 in Supplement 1).

*S agalactiae* was among the most frequent pathogens in neonates (76 of 630 studies [12%]; 86 of 733 study periods [12%]; 4584 of 314 454 episodes [1.5%])^25,32,38,49,53,63,65,70,71,85,86,89,93,96,97,102,108,116,119,120,121,122,127,132,133,141,144,147,148,149,152,177,187,194,200,201,212,216,217,220,223,230,235,241,266,268,282,300,304,311,329,333,336,337,344,347,352,358,360,361,373,393,407,640,641,642,643,644,645,646,647,648,649,650,651,652^ (eTable 24 and eFigure 47 in Supplement 1). The overall CFR was 16% (95% CI, 13% to 19%) (eFigure 48 in Supplement 1); CFR was 60% (95% CI, 33% to 82%) before 1961 and 9% (95% CI, 6% to 13%) in the 2000s. CFR was higher in low-income countries (32%; 95% CI, 11% to 65%) compared with high-income countries (14%; 95% CI, 10% to 19%) (eTable 25 in Supplement 1) and overall decreasing (−5% per year; 95% CI, −6% to −3% per year; *P* < .001) (eFigures 49 to 51 in Supplement 1).

## Discussion

Our study provides a systematic, worldwide, 80-year overview of pathogen distributions and CFRs of community-acquired bacterial meningitis. Our findings underscore substantial advantages in treatment and prevention of this disease.^1,2,3^ The proportion of meningitis caused by *H influenzae* has dramatically dropped after introduction of routine vaccination against this pathogen; conversely, despite the availability of serotype-specific vaccines, the proportion of pneumococcal meningitis has increased over time.^4^ The observed reduction in the overall CFR from 32% before 1961 to 15% in the 2010s aligns with the outcomes of studies encompassing diverse geographic regions included in our meta-analysis. The decline in CFRss can be attributed to the overalll enhancement of medical care, including increased accessibility, advancements in critical care, and new antibiotic therapies.^653^ Decreases in CFRs are comparable with those reported for other diseases. The global disease burden analysis showed a decrease in mortality due to communicable diseases, driven by reduction of deaths due to human immunodeficiency virus (HIV), malaria, tuberculosis, and diarrheal diseases, which are the large contributors to global mortality.^654^ Within our meta-analysis, a substantial decline in CFRs was present in patients with pneumococcal meningitis. Additionally, declining CFRs were also apparent in pathogens predominantly affecting neonates (*S agalactiae*, *E coli*).

CFRs were highest for meningitis caused by *L monocytogenes* and *S pneumoniae* whereas CFRs for meningitis caused by *E coli*, *S agalactiae*, *H influenzae*, and *N meningitidis* were lower after 2000. The overall decrease in CFR started to stagnate around 1990. A potentially increasing CFR was observed in adults of low-income countries. These observations may have several explanations. First, it coincides with the origin and rise of HIV, which increases the risk of serious infections, including bacterial meningitis, and also worse outcomes.^655,656,657^ Second, widespread use of conjugate vaccines against *H influenzae* type B led to a strong decrease of this pathogen in high-income countries shortly after initial licensure in the 1990s and with a delay in many low-income countries.^4,658^ This virtual elimination of meningitis due to *H influenzae* led to relative increase of pneumococcal meningitis, which is associated with higher age-adjusted CFRs. Our data confirm that the decline of the proportion of *H influenzae* in low-income countries occurred one decade after the decline in high-income countries.

From 2000 to 2019, when the proportion of pneumococcal meningitis increased, the overall CFR remained stable. Two important changes in prevention and management occurred during this time. First, pneumococcal conjugate vaccines were introduced in high-income countries. The introduction of the 7-valent conjugate pneumococcal vaccine in the US in 2000 led to a decrease of 90% of invasive pneumococcal disease, including meningitis, for the serotypes included in the vaccine.^4,659^ Although the increase in the relative proportion of pneumococcal meningitis may lead to worse outcomes for the overall CFR, the decreasing CFR within the pneumococcal subgroup was most pronounced, at least in high-income countries. The introduction of adjunctive anti-inflammatory treatment, dexamethasone, contributed to this decline. Cohort studies have shown improved outcomes of bacterial meningitis after implementation of adjunctive dexamethasone therapy in several countries.^332,463^ This practice gained widespread adoption following a landmark trial of dexamethasone in adults with bacterial meningitis in 2002.^8^ After its publication,^8^ a meta-analyses^660^ showed that adjunctive treatment with corticosteroids resulted in a reduction in hearing loss and neurological sequelae. The beneficial effect of dexamethasone was particularly evident in the most severely ill patients, specifically those with pneumococcal meningitis. No beneficial effects of adjunctive corticosteroids have been identified in studies done in low-income countries.^10,13^ Naturally, other factors could have influenced the improved outcome, such as increased awareness of the importance of fast administration of antibiotic therapy. Several studies showed faster treatment in bacterial meningitis patients is associated with improved outcomes.^661,662,663,664^ The 2016 European Society of Clinical Microbiology and Infectious Diseases guideline advises to start treatment as soon as possible but at least within 1 hour of arrival in the emergency department.^665^

We found a consistent disparity between outcomes in higher and low-income countries. This can be explained by a lower level of access to health care with limited resources and treatment options and a higher rate of coexisting HIV infection and malnutrition.^666^ Furthermore, limitations in the availability of diagnostic tests and surveillance systems hinder the differentiation between malaria and tuberculous meningitis with bacterial meningitis, which may delay appropriate treatment and lead to underreporting.^666^ Finally, a higher bacterial resistance rate to common antimicrobial treatments and lack of effect of adjunctive dexamethasone may contribute to the identified difference in mortality rate.^13^ The impact of mortality and morbidity on a population level remains much higher for low-income countries.^667^ Our findings underscore the critical role of enhanced medical care accessibility and the development of specific treatments in driving improvements in CFRs, particularly in the most severe cases and vulnerable populations.

### Limitations

This study has limitations. First, many of the included studies were retrospective and may not be representative for the entire population of bacterial meningitis patients. Future studies should preferentially be large prospective cohorts or from nationwide registry data to avoid selection bias. Second, we cannot exclude a selection bias due to limitations to the languages and databases included. However, we did not find any funnel plot asymmetries. Third, we did not systematically assess the quality of the included studies because we aimed to include studies from diverse settings and using unadjusted proportions. Instead, we addressed heterogeneity with subgroup analysis and meta-regression. The consistently high residual heterogeneity potentially reflects the discrepancies in a clinical setting across differing populations and unequal health care systems rather than the original studies’ discrepancies in methods. As our meta-analysis included more than 150 000 patients who were not selected by a specific pathogen, the estimates on overall case fatality from bacterial meningitis are most representative globally. This allowed us to draw conclusions about general trends of pathogen distributions and case fatality ratios. Finally, our data do not provide a complete picture of the burden of bacterial meningitis. Patients who survive bacterial meningitis frequently have neurological sequelae, which include deafness and neurological and neuropsychological deficits.^668,669^ In addition to the physical disability, this may lead to functional, social, and economic impairment.

## Conclusions

In this study, we observed declining CFRs of bacterial meningitis overall and in relevant subgroups (neonates, children, and adults; high- and low-income countries), except for adults in low-income countries, where we found no evidence for a decline. Reduced CFRs in pneumococcal meningitis primarily drove this decline, despite the relative increase of meningitis caused by *S pneumoniae*. Because of the substantial mortality and morbidity, there remains an urgent need to optimally deploy existing vaccines worldwide and develop new prevention strategies and treatment options.

## Notes

Supplement 1. **eTable 1.** Search Strategies Used **eTable 2.** R Commands **eTable 3.** R Packages **eTable 4.** Studies Reporting on Multiple Pathogens **eTable 5.** Proportions of the Causative Pathogens, Stratified by Mean Study Periods **eTable 6.** Proportions of the Causative Pathogens, Stratified by Age Group **eTable 7.** Results of the Meta-Regression Model, Stratified by Human Development Index (HDI, High-Income Countries-Income Countries, Low-Income Countries-Income Countries) and Age Group **eTable 8.** Studies Reporting on *Streptococcus pneumoniae* Specifically **eTable 9.** Studies Reporting on *Neisseria meningitidis* Specifically **eTable 10.** Studies Reporting on *Haemophilus influenzae* Specifically **eTable 11.** Studies Reporting on *Listeria monocytogenes* Specifically **eTable 12.** Studies Reporting on *Escherichia coli* Specifically **eTable 13.** Studies Reporting on *Streptococcus agalactiae* Specifically **eTable 14.** Study Characteristics and Unadjusted Case Fatality Ratios in Pneumococcal Meningitis Per Subgroup **eTable 15.** Results of the Meta-Analyses of Case Fatality Ratios in Pneumococcal Meningitis Per Subgroup **eTable 16.** Study Characteristics and Unadjusted Case Fatality Ratios in Meningococcal Meningitis Per Subgroup **eTable 17.** Results of the Meta-Analyses of Case Fatality Ratios in Meningococcal Meningitis Per Subgroup **eTable 18.** Study Characteristics and Unadjusted Case Fatality Ratios in *H influenzae* Meningitis Per Subgroup **eTable 19.** Results of the Meta-Analyses of Case Fatality Ratios in *H influenzae* Meningitis Per Subgroup **eTable 20.** Study Characteristics and Unadjusted Case Fatality Ratios in *L monocytogenes* Meningitis Per Subgroup **eTable 21.** Results of the Meta-Analyses of Case Fatality Ratios in *L monocytogenes *Meningitis Per Subgroup **eTable 22.** Study Characteristics and Unadjusted Case Fatality Ratios in *E coli* Meningitis Per Subgroup **eTable 23.** Results of the Meta-Analyses of Case Fatality Ratios in *E coli* Meningitis Per Subgroup **eTable 24.** Study Characteristics and Unadjusted Case Fatality Ratios in *S agalactiae* Meningitis Per Subgroup **eTable 25.** Results of the Meta-Analyses of Case Fatality Ratios in *S agalactiae* Meningitis Per Subgroup **eFigure 1.** Flow Chart of the Study Selection Process **eFigure 2.** Geographic Distribution of Studies Evaluating Case Fatality Ratios in Bacterial Meningitis and Included Into the Meta-Analysis **eFigure 3.** Proportions of Causative Pathogens Per Time Interval, Stratified by Age Group (Neonates, 0 to 2 Months; Children, 2 Months to 16 Years; Adults) **eFigure 4.** Proportions of Causative Pathogens Per Time Interval, Stratified by Country Group (High-Income, Low-Income) **eFigure 5.** Mean Case Fatality Ratios of Bacterial Meningitis Per Country, Stratified by Time Intervals **eFigure 6.** Funnel Plot of All Studies Included **eFigure 7.** Funnel Plots of the Meta-Analyses, Stratified by Age Groups (Neonates, 0 to 2 Months; Children, 2 Months to 16 Years; Adults) and Country Group **eFigure 8.** Forest Plot of Studies in High-Income Countries on Neonates **eFigure 9. **Forest Plot of Studies in High-Income Countries on Children **eFigure 10.** Forest Plot of Studies in High-Income Countries on Adults **eFigure 11.** Forest Plot of Studies in Low-Income Countries on Neonates **eFigure 12.** Forest Plot of Studies in Low-Income Countries on Children **eFigure 13.** Forest Plot of Studies in Low-Income Countries on Adults **eFigure 14.** Case Fatality Ratio of Patients With Bacterial Meningitis Using a Meta-Regression Model With the Studies’ Mean Observation Year as Estimator Variable **eFigure 15.** Case Fatality Ratios of Patients With Bacterial Meningitis Using a Meta-Regression Model With the Studies’ Mean Observation Year as Estimator Variable, Stratified According to Age Groups in Low-Income Countries **eFigure 16.** Geographic Distribution of Studies Evaluating Case Fatality Ratios in Pneumococcal Meningitis and Included Into the Meta-Analysis **eFigure 17.** Case Fatality Ratios in *S pneumoniae* Meningitis (Forest Plot With Individual Studies Suppressed) Indicating the Overall Pooled Estimate and the Estimates of the Intervals as Subgroups **eFigure 18.** Case Fatality Ratios of Patients With Pneumococcal Meningitis Using a Meta-Regression Model With the Studies’ Mean Observation Year as Estimator Variable **eFigure 19.** Forest Plot of Studies in High-Income Countries on Neonates With Pneumococcal Meningitis **eFigure 20.** Forest Plot of Studies in High-Income Countries on Children With Pneumococcal Meningitis **eFigure 21.** Forest PLOT of Studies in High-Income Countries on Adults With Pneumococcal Meningitis **eFigure 22.** Forest Plot of Studies in Low-Income Countries on Neonates With Pneumococcal Meningitis **eFigure 23.** Forest Plot of Studies in Low-Income Countries on Children With Pneumococcal Meningitis **eFigure 24.** Forest Plot of Studies in Low-Income Countries on Adults With Pneumococcal Meningitis **eFigure 25.** Case Fatality Ratios of Patients With Pneumococcal Meningitis Using a Meta-Regression Model With the Studies’ Mean Observation Year as Estimator Variable, Stratified According to the Age Group (Neonates, 0 to 2 Months; Children, 2 Months to 16 Years; Adults) and the Human Development Index (High-Income Countries, Low-Income Countries) **eFigure 26. **Geographic Distribution of Studies Evaluating Case Fatality Ratios in Pneumococcal Meningitis and Included Into the Meta-Analysis **eFigure 27.** Geographic Distribution of Studies Evaluating Case Fatality Ratios in Meningococcal Meningitis and Included Into the Meta-Analysis **eFigure 28.** Case Fatality Ratios in *N meningitidis* Meningitis (Forest Plot With Individual Studies Suppressed) Indicating the Overall Pooled Estimate and the Estimates of the Intervals as Subgroups **eFigure 29.** Case Fatality Ratios of Patients With Meningococcal Meningitis Using a Meta-Regression Model With the Studies’ Mean Observation Year as Estimator Variable **eFigure 30.** Case Fatality Ratios of Patients With Meningococcal Meningitis Using a Meta-Regression Model With the Studies’ Mean Observation Year as Estimator Variable, Stratified According to the Age Group (Children, 2 Months to 16 Years; Adults) and the Human Development Index (High-Income Countries, Low-Income Countries) **eFigure 31.** Geographic Distribution of Studies Evaluating Case Fatality Ratios in Meningococcal Meningitis and Included Into the Meta-Analysis **eFigure 32.** Geographic Distribution of Studies Evaluating Case Fatality Ratios in *H influenzae* Meningitis and Included Into the Meta-Analysis **eFigure 33.** Case Fatality Ratios in *H influenzae* Meningitis (Forest Plot With Individual Studies Suppressed) Indicating the Overall Pooled Estimate and the Estimates of the Intervals as Subgroups **eFigure 34.** Case Fatality Ratios of Patients With *H influenzae* Meningitis Using a Meta-Regression Model With the Studies’ Mean Observation Year as Estimator Variable **eFigure 35. **Case Fatality Ratios of Patients With *H influenzae* Meningitis Using a Meta-Regression Model With the Studies’ Mean Observation Year as Estimator Variable, Stratified According to the Age Group (Children, 2 Months to 16 Years) and the Human Development Index (High-Income Countries, Low-Income Countries) **eFigure 36.** Geographic Distribution of Studies Evaluating Case Fatality Ratios in *H influenzae* meningitis and Included into the Meta-Analysis **eFigure 37.** Geographic Distribution of Studies Evaluating Case Fatality Ratios In Hib Meningitis and Included Into the Meta-Analysis **eFigure 38.** Case Fatality Ratios in *L monocytogenes* Meningitis Indicating the Overall Pooled Estimate and the Estimates of the Intervals as Subgroups **eFigure 39.** Case Fatality Ratios of Patients With *L monocytogenes* Meningitis Using a Meta-Regression Model With the Studies’ Mean Observation Year as Estimator Variable **eFigure 40. **Case Fatality Ratios of Adult Patients in High-Income Countries With *L monocytogenes* Meningitis Using Meta-Regression Model With the Studies’ Mean Observation Year as Estimator Variable **eFigure 41.** Geographic Distribution of Studies Evaluating Case Fatality Ratios in *L monocytogenes* Meningitis and Included Into the Meta-Analysis **eFigure 42.** Geographic Distribution of Studies Evaluating Case Fatality Ratios in *E coli* Meningitis and Included Into the Meta-Analysis **eFigure 43.** Case Fatality Ratios in *E coli* (Forest Plot With Individual Studies Suppressed) Indicating the Overall Pooled Estimate and the Estimates of the Intervals as Subgroups **eFigure 44.** Case Fatality Ratios of Patients With *E coli* Meningitis Using a Meta-Regression Model With the Studies’ Mean Observation Year as Estimator Variable **eFigure 45.** Case Fatality Ratios of Patients With *E coli* Meningitis Using a Meta-Regression Model With the Studies’ Mean Observation Year as Estimator Variable, Stratified According to the Age Group (Neonates, 0 to 2 months) and the Human Development Index (High-Income Countries, Low-Income Countries) **eFigure 46.** Geographic Distribution of Studies Evaluating Case Fatality Ratios in *E coli* Meningitis and Included Into the Meta-Analysis **eFigure 47. **Geographic Distribution of Studies Evaluating Case Fatality Ratios in *S agalactiae* Meningitis and Included Into the Meta-Analysis **eFigure 48.** Case Fatality Ratios in *S agalactiae* Meningitis (Forest Plot With Individual Studies Suppressed) Indicating the Overall Pooled Estimate and the Estimates of the Intervals as Subgroups **eFigure 49.** Case Fatality Ratios of Patients With *S agalactiae* Meningitis Using a Meta-Regression Model With the Studies’ Mean Observation Year as Estimator Variable **eFigure 50.** Case Fatality Ratios of Patients With *S agalactiae* Meningitis Using a Meta-Regression Model With the Studies’ Mean Observation Year as Estimator Variable, Stratified According to the Age Group (Neonates, 0 to 2 Months) and the Human Development Index (High-Income Countries, Low-Income Countries) **eFigure 51.** Geographic Distribution of Studies Evaluating Case Fatality Ratios in *S agalactiae* Meningitis and Included Into the Meta-Analysis

Supplement 2.
Data Sharing Statement
