# Supplementary material for: Global Case Fatality of Bacterial Meningitis During an 80-Year Period: A Systematic Review and Meta-Analysis
Source: JAMA Netw Open. 2024 Aug 2;7(8):e2424802. doi: 10.1001/jamanetworkopen.2024.24802 (PMC11297475; doi:10.1001/jamanetworkopen.2024.24802)
Supplement: Supplement 1. — eTable 1. Search Strategies Used eTable 2. R Commands eTable 3. R Packages eTable 4. Studies Reporting on Multiple Pathogens eTable 5. Proportions of the Causative Pathogens, Stratified by Mean Study Periods eTable 6. Proportions of the Causative Pathogens, Stratified by Age Group eTable 7. Results of the Meta-Regression Model, Stratified by Human Development Index (HDI, High-Income Countries-Income Countries, Low-Income Countries-Income Countries) and Age Group eTable 8. Studies Reporting on Streptococcus pneumoniae Specifically eTable 9. Studies Reporting on Neisseria meningitidis Specifically eTable 10. Studies Reporting on Haemophilus influenzae Specifically eTable 11. Studies Reporting on Listeria monocytogenes Specifically eTable 12. Studies Reporting on Escherichia coli Specifically eTable 13. Studies Reporting on Streptococcus agalactiae Specifically eTable 14. Study Characteristics and Unadjusted Case Fatality Ratios in Pneumococcal Meningitis Per Subgroup eTable 15. Results of the Meta-Analyses of Case Fatality Ratios in Pneumococcal Meningitis Per Subgroup eTable 16. Study Characteristics and Unadjusted Case Fatality Ratios in Meningococcal Meningitis Per Subgroup eTable 17. Results of the Meta-Analyses of Case Fatality Ratios in Meningococcal Meningitis Per Subgroup eTable 18. Study Characteristics and Unadjusted Case Fatality Ratios in H influenzae Meningitis Per Subgroup eTable 19. Results of the Meta-Analyses of Case Fatality Ratios in H influenzae Meningitis Per Subgroup eTable 20. Study Characteristics and Unadjusted Case Fatality Ratios in L monocytogenes Meningitis Per Subgroup eTable 21. Results of the Meta-Analyses of Case Fatality Ratios in L monocytogenes Meningitis Per Subgroup eTable 22. Study Characteristics and Unadjusted Case Fatality Ratios in E coli Meningitis Per Subgroup eTable 23. Results of the Meta-Analyses of Case Fatality Ratios in E coli Meningitis Per Subgroup eTable 24. Study Characteristics and Unadjusted Case Fatality Ratios [file jamanetwopen-e2424802-s001.pdf]

Supplemental Online Content

van Ettekoven CN, Liechti FD, Brouwer MC, Bijlsma MW, van de Beek D. Global case fatality of bacterial meningitis over during an 80-year period: a systematic review and meta-analysis. *JAMA Netw Open*. 2024;7(7):e2424802. doi:10.1001/jamanetworkopen.2024.24802

eTable 1. Search Strategies Used
eTable 2. R Commands
eTable 3. R Packages
eTable 4. Studies Reporting on Multiple Pathogens
eTable 5. Proportions of the Causative Pathogens, Stratified by Mean Study Periods
eTable 6. Proportions of the Causative Pathogens, Stratified by Age Group
eTable 7. Results of the Meta-Regression Model, Stratified by Human Development Index (HDI, High-Income Countries-Income Countries, Low-Income Countries-Income Countries) and Age Group
eTable 8. Studies Reporting on *Streptococcus pneumoniae* Specifically
eTable 9. Studies Reporting on *Neisseria meningitidis* Specifically
eTable 10. Studies Reporting on *Haemophilus influenzae* Specifically
eTable 11. Studies Reporting on *Listeria monocytogenes* Specifically
eTable 12. Studies Reporting on *Escherichia coli* Specifically
eTable 13. Studies Reporting on *Streptococcus agalactiae* Specifically
eTable 14. Study Characteristics and Unadjusted Case Fatality Ratios in Pneumococcal Meningitis Per Subgroup
eTable 15. Results of the Meta-Analyses of Case Fatality Ratios in Pneumococcal Meningitis Per Subgroup
eTable 16. Study Characteristics and Unadjusted Case Fatality Ratios in Meningococcal Meningitis Per Subgroup
eTable 17. Results of the Meta-Analyses of Case Fatality Ratios in Meningococcal Meningitis Per Subgroup
eTable 18. Study Characteristics and Unadjusted Case Fatality Ratios in *H influenzae* Meningitis Per Subgroup
eTable 19. Results of the Meta-Analyses of Case Fatality Ratios in *H influenzae* Meningitis Per Subgroup
eTable 20. Study Characteristics and Unadjusted Case Fatality Ratios in *L monocytogenes* Meningitis Per Subgroup
eTable 21. Results of the Meta-Analyses of Case Fatality Ratios in *L monocytogenes* Meningitis Per Subgroup
eTable 22. Study Characteristics and Unadjusted Case Fatality Ratios in *E coli* Meningitis Per Subgroup
eTable 23. Results of the Meta-Analyses of Case Fatality Ratios in *E coli* meningitis per subgroup
eTable 24. Study Characteristics and Unadjusted Case Fatality Ratios in *S agalactiae* Meningitis Per Subgroup
eTable 25. Results of the Meta-Analyses of Case Fatality Ratios in *S agalactiae* Meningitis Per Subgroup
eFigure 1. Flow Chart of the Study Selection Process
eFigure 2. Geographic Distribution of Studies Evaluating Case Fatality Ratios in Bacterial Meningitis and Included Into the Meta-Analysis
eFigure 3. Proportions of Causative Pathogens Per Time Interval, Stratified by Age Group (Neonates, 0 to 2 Months; Children, 2 Months to 16 Years; Adults)
eFigure 4. Proportions of Causative Pathogens Per Time Interval, Stratified by Country Group (High-Income, Low Income)
eFigure 5. Mean Case Fatality Ratios of Bacterial Meningitis Per Country, Stratified by Time Intervals
eFigure 6. Funnel Plot of All Studies Included
eFigure 7. Funnel Plots of the Meta-Analyses, Stratified by Age Groups (Neonates, 0 to 2 Months; Children, 2 Months to 16 Years; Adults) and Country Group
eFigure 8. Forest Plot of Studies in High-Income Countries on Neonates
eFigure 9. Forest Plot of Studies in High-Income Countries on Children
eFigure 10. Forest Plot of Studies in High-Income Countries on Adults

© 2024 van Ettekoven CN et al. *JAMA Network Open.*

eFigure 11. Forest Plot of Studies in low-income Countries on Neonates
eFigure 12. Forest Plot of Studies in Low-Income Countries on Children
eFigure 13. Forest Plot of Studies in Low-Income Countries on Adults
eFigure 14. Case Fatality Ratio of Patients With Bacterial Meningitis Using a Meta-Regression Model With the Studies' Mean Observation Year as Estimator Variable
eFigure 15. Case Fatality Ratios of Patients With Bacterial Meningitis Using a Meta-Regression Model With the Studies' Mean Observation Year as Estimator Variable, Stratified According to Age Groups (Neonates, younger than 2 months; Children, 2 months to 16 Years; Adults, Older Than 16 Years) in Low-Income Countries
eFigure 16. Geographic Distribution of Studies Evaluating Case Fatality Ratios in Pneumococcal Meningitis and Included Into the Meta-Analysis
eFigure 17. Case Fatality Ratios in *S pneumoniae* Meningitis (Forest Plot With Individual Studies Suppressed) Indicating the Overall Pooled Estimate and the Estimates of the Intervals as Subgroups
eFigure 18. Case Fatality Ratios of Patients With Pneumococcal Meningitis Using a Meta-Regression Model With the Studies' Mean Observation Year as Estimator Variable
eFigure 19. Forest Plot of Studies in High-Income Countries on Neonates With Pneumococcal Meningitis
eFigure 20. Forest Plot of Studies in High-Income Countries on Children With Pneumococcal Meningitis
eFigure 21. Forest PLOT of Studies in High-Income Countries on Adults With Pneumococcal Meningitis
eFigure 22. Forest Plot of Studies in Low-Income Countries on Neonates With Pneumococcal Meningitis
eFigure 23. Forest Plot of Studies in Low-Income Countries on Children With Pneumococcal Meningitis
e**Figure 24.** Forest Plot of Studies in Low-Income Countries on Adults With Pneumococcal Meningitis
eFigure 25. Case fatality Ratios of Patients With Pneumococcal Meningitis Using a Meta-Regression Model With the Studies' Mean Observation Year as Estimator Variable, Stratified According to the Age Group (Neonates, 0 to 2 Months; Children, 2 Months to 16 Years; Adults) and the Human Development Index (High-Income Countries, Low-Income Countries)
eFigure 26. Geographic Distribution of Studies Evaluating Case Fatality Ratios in Pneumococcal Meningitis and Included Into the Meta-Analysis
eFigure 27. Geographic Distribution of Studies Evaluating Case Fatality Ratios in Meningococcal Meningitis and Included Into the Meta-Analysis
eFigure 28. Case Fatality Ratios in *N Meningitidis* Meningitis (Forest Plot With Individual Studies Suppressed) Indicating the Overall Pooled Estimate and the Estimates of the Intervals as Subgroups
eFigure 29. Case Fatality Ratios of Patients With Meningococcal Meningitis Using a Meta-Regression Model With the Studies' Mean Observation Year as Estimator Variable
eFigure 30. Case Fatality Ratios of Patients With Meningococcal Meningitis Using a Meta-Regression Model With the Studies' Mean Observation Year as Estimator Variable, Stratified According to the Age Group (Children, 2 Months to 16 Years; Adults) and the Human Development Index (High-Income Countries, Low-Income Countries)
eFigure 31. Geographic Distribution of Studies Evaluating Case Fatality Ratios in Meningococcal Meningitis and Included Into the Meta-Analysis
eFigure 32. Geographic Distribution of Studies Evaluating Case Fatality Ratios in *H influenzae* Meningitis and Included Into the Meta-Analysis
eFigure 33. Case Fatality Ratios in *H influenzae* Meningitis (Forest Plot With Individual Studies Suppressed) Indicating the Overall Pooled Estimate and the Estimates of the Intervals as Subgroups
eFigure 34. Case Fatality Ratios of Patients With *H. influenzae* Meningitis Using a Meta-Regression Model With the Studies' Mean Observation Year as Estimator Variable
eFigure 35. Case Fatality Ratios of Patients With *H influenzae* Meningitis Using a Meta-Regression Model With the Studies' Mean Observation Year as Estimator Variable, Stratified According to The Age Group (Children, 2 Months to 16 Years) and the Human Development Index (High-Income Countries, Low-Income Countries)
eFigure 36. Geographic Distribution of Studies Evaluating Case Fatality Ratios in *H influenzae meningitis* and Included into the Meta-Analysis
eFigure 37. Geographic Distribution of Studies Evaluating Case Fatality Ratios In Hib Meningitis and Included Into the Meta-Analysis
eFigure 38. Case Fatality Ratios in *L monocytogenes* Meningitis Indicating the Overall Pooled Estimate and the Estimates of the Intervals as Subgroups
eFigure 39. Case Fatality Ratios of Patients With *L monocytogenes* Meningitis Using a Meta-Regression Model With the Studies' Mean Observation Year as Estimator Variable

© 2024 van Ettekoven CN et al. *JAMA Network Open.*

eFigure 40. Case Fatality Ratios of Adult Patients in High-Income Countries With *L monocytogenes* Meningitis Using Meta-Regression Model With the Studies' Mean Observation Year as Estimator Variable
eFigure 41. Geographic Distribution of Studies Evaluating Case Fatality Ratios in *L monocytogenes* Meningitis and Included Into the Meta-Analysis
eFigure 42. Geographic Distribution of Studies Evaluating Case Fatality Ratios in *E coli* Meningitis and Included Into the Meta-Analysis
eFigure 43. Case Fatality Ratios in *E coli* (Forest Plot With Individual Studies Suppressed) Indicating the Overall Pooled Estimate and the Estimates of the Intervals as Subgroups
eFigure 44. Case Fatality Ratios of Patients With *E coli* Meningitis Using a Meta-Regression Model With the Studies' Mean Observation Year as Estimator Variable
eFigure 45. Case Fatality Ratios of Patients With *E coli* Meningitis Using a Meta-Regression Model With the Studies' Mean Observation Year as Estimator Variable, Stratified According to the Age Group (Neonates, 0 to 2 months) and the Human Development Index (High-Income Countries, Low-Income Countries
eFigure 46. Geographic Distribution of Studies Evaluating Case Fatality Ratios in *E coli* Meningitis and Included Into the Meta-Analysis
eFigure 47. Geographic Distribution of Studies Evaluating Case Fatality Ratios in *S agalactiae* Meningitis and Included Into the Meta-Analysis
eFigure 48. Case Fatality Ratios in *S agalactiae* Meningitis (Forest Plot With Individual Studies Suppressed) Indicating the Overall Pooled Estimate and the Estimates of the Intervals as Subgroups
eFigure 49. Case Fatality Ratios of Patients With *S agalactiae* Meningitis Using a Meta-Regression Model With the Studies' Mean Observation Year as Estimator Variable
eFigure 50. Case Fatality Ratios of Patients With *S agalactiae* Meningitis Using a Meta-Regression Model With the Studies' Mean Observation Year as Estimator Variable, Stratified According to the Age Group (Neonates, 0 to 2 Months) and the Human Development Index (High-Income Countries, Low-Income Countries)
eFigure 51. Geographic Distribution of Studies Evaluating Case Fatality Ratios in *S agalactiae* Meningitis and Included Into the Meta-Analysis

This supplemental material has been provided by the authors to give readers additional information about their work.

© 2024 van Ettekoven CN et al. *JAMA Network Open.*

eTable 1. Search strategies used.

Database	Search term
**Medline (PubMed) (1970 to January 1, 2022)**	(("meningitis, bacterial"[MeSH Terms] OR ("meningitis"[All Fields] AND "bacterial"[All Fields]) OR "bacterial meningitis"[All Fields]<br><br>OR ("bacterial"[All Fields] AND "meningitis"[All Fields])) AND ("mortality"[Subheading] OR "mortality"[All Fields] OR "mortality"[MeSH Terms])) AND ("humans"[MeSH Terms] AND (English[lang] OR French[lang] OR German[lang] OR Spanish[lang]))
**Google Scholar (first 100 results)**	(bacterial AND meningitis) AND (mortality OR "case fatality") -animal

© 2024 van Ettekoven CN et al. *JAMA Network Open.*

Statistical analysis

eTable 2. R commands

Primary analysis	`data_ma <- metafor::escalc(xi = Deaths, ni = Patients, data = data,`<br>`measure = "PLO") # logit transformation`<br><br>`ma_all <- meta::metaprop(Deaths, Patients, studlab = Studyauthor, data = data_ma,`<br>`sm = "PLO", method.tau = "REML", method.ci = "NAsm",`<br>`prediction = TRUE, subgroup = Interval)`<br><br>`metareg_all <- meta::metareg(ma_all, Periodmean, method = "REML")`<br><br>`metafor::regplot(metareg_all, pi = TRUE, mod = "Periodmean", transf = transf.ilogit,`<br>`legend = FALSE, bg = scales::alpha("#3C5488B2", .3),`<br>`xlim = c(1940, 2020), ylim = c(0, 1),`<br>`ylab = "Case fatality ratio")`
Subgroup analysis[1]	`ma_int <- meta::metaprop(Deaths, Patients, studlab = Studyauthor, data = data_ma,`<br>`sm = "PLO", method.tau = "REML", method.ci = "NAsm",`<br>`control = list(maxiter = 1000),`<br>`prediction = TRUE, subgroup = Interval, tau.common = FALSE)`<br><br>`metareg_int <- meta::metareg(ma_int, Periodmean, method = "REML",`<br>`control = list(maxiter = 1000))`<br><br>`metafor::regplot(metareg_int, pi = TRUE, mod = "Periodmean", transf = transf.ilogit,`<br>`legend = FALSE, bg = scales::alpha("#3C5488B2", .3),`<br>`xlim = c(1940, 2020), ylim = c(0, 1),`<br>`ylab = "Case fatality ratio", xlab = "Year")`

[1] In the subgroup analyses, we used the DerSimonian and Laird method (method.tau = “DL”) instead of the restricted maximum likelihood method (“REML”) if the Fisher algorithm did not converge, i.e. for the meta-regression analysis of *S. pneumoniae* in neonates from high-income countries.

© 2024 van Ettekoven CN et al. *JAMA Network Open.*

eTable 3. R packages

- Balduzzi S, Rücker G, Schwarzer G (2019). “How to perform a meta-analysis with R: a practical tutorial.” _Evidence-Based Mental Health_, 153-160.
- Bates D, Maechler M, Jagan M (2024). _Matrix: Sparse and Dense Matrix Classes and Methods_. R package version 1.6-5, <https://CRAN.R-project.org/package=Matrix>.
- Gilbert P, Varadhan R (2019). _numDeriv: Accurate Numerical Derivatives_. R package version 2016.8-1.1, <https://CRAN.R-project.org/package=numDeriv>.
- Grolemund G, Wickham H (2011). “Dates and Times Made Easy with lubridate.” _Journal of Statistical Software_, *40*(3), 1-25. <https://www.jstatsoft.org/v40/i03/>.
- Müller K, Wickham H (2023). _tibble: Simple Data Frames_. R package version 3.2.1, <https://CRAN.R-project.org/package=tibble>.
- Pebesma E, Bivand R (2023). _Spatial Data Science: With applications in R_. Chapman and Hall/CRC. doi:10.1201/9780429459016 <https://doi.org/10.1201/9780429459016>, <https://r-spatial.org/book/>. Pebesma E (2018). “Simple Features for R: Standardized Support for Spatial Vector Data.” _The R Journal_, *10*(1), 439-446. doi:10.32614/RJ-2018-009 <https://doi.org/10.32614/RJ-2018-009>, <https://doi.org/10.32614/RJ-2018-009>.
- R Core Team (2022). _R: A Language and Environment for Statistical Computing_. R Foundation for Statistical Computing, Vienna, Austria. <https://www.R-project.org/>.
- Sjoberg D, Whiting K, Curry M, Lavery J, Larmarange J (2021). “Reproducible Summary Tables with the gtsummary Package.” _The R Journal_, *13*, 570-580. doi:10.32614/RJ-2021-053 <https://doi.org/10.32614/RJ-2021-053>, <https://doi.org/10.32614/RJ-2021-053>.
- Viechtbauer W (2010). “Conducting meta-analyses in R with the metafor package.” _Journal of Statistical Software_, *36*(3), 1-48. doi:10.18637/jss.v036.i03 <https://doi.org/10.18637/jss.v036.i03>.
- White T, Noble D, Senior A, Hamilton W, Viechtbauer W (2022). _metadat: Meta-Analysis Datasets_. R package version 1.2-0, <https://CRAN.R-project.org/package=metadat>.
- Wickham H (2016). _ggplot2: Elegant Graphics for Data Analysis_. Springer-Verlag New York. ISBN 978-3-319-24277-4, <https://ggplot2.tidyverse.org>.
- Wickham H (2023). _forcats: Tools for Working with Categorical Variables (Factors)_. R package version 1.0.0, <https://CRAN.R-project.org/package=forcats>.
- Wickham H (2023). _stringr: Simple, Consistent Wrappers for Common String Operations_. R package version 1.5.1, <https://CRAN.R-project.org/package=stringr>.
- Wickham H, Averick M, Bryan J, Chang W, McGowan LD, François R, Grolemund G, Hayes A, Henry L, Hester J, Kuhn M, Pedersen TL, Miller E, Bache SM, Müller K, Ooms J, Robinson D, Seidel DP, Spinu V, Takahashi K, Vaughan D, Wilke C, Woo K, Yutani H (2019). “Welcome to the tidyverse.” _Journal of Open Source Software_, *4*(43), 1686. doi:10.21105/joss.01686 <https://doi.org/10.21105/joss.01686>.
- Wickham H, François R, Henry L, Müller K, Vaughan D (2023). _dplyr: A Grammar of Data Manipulation_. R package version 1.1.4, <https://CRAN.R-project.org/package=dplyr>.
- Wickham H, Henry L (2023). _purrr: Functional Programming Tools_. R package version 1.0.2, <https://CRAN.R-project.org/package=purrr>.
- Wickham H, Hester J, Bryan J (2024). _readr: Read Rectangular Text Data_. R package version 2.1.5, <https://CRAN.R-project.org/package=readr>.
- Wickham H, Vaughan D, Girlich M (2023). _tidyr: Tidy Messy Data_. R package version 1.3.0, <https://CRAN.R-project.org/package=tidyr>.

© 2024 van Ettekoven CN et al. *JAMA Network Open.*

Publication lists

eTable 4. Studies reporting on multiple pathogens.

First author [reference]	Pub. year	Country	HDI[2]	Inclusion period	Age group	Patients, n	Deaths, n
**Brainerd** [1]	1947	United States	high	1943-01-01 – 1946-02-28	Not specified	250	73
**Smith** [2]	1954	United States	high	1944-01-01 – 1953-12-31	Neonates and children	354	88
**Watson** [3]	1957	Multinational	high	1949-10-31 – 1955-12-31	Neonates	44	29
**Shaper** [4]	1958	Uganda	low	1957-01-01 – 1957-12-31	Not specified	110	59
**Eigler** [5]	1961	United States	high	1948-01-01 – 1958-01-01	Not specified	294	50
**Esrachowitz** [6]	1961	South Africa	low	1955-01-01 – 1957-12-31	Not specified	303	28
**Groover** [7]	1961	United States	high	1948-01-01 – 1959-12-31	Neonates	39	26
**Carpenter** [8]	1962	United States	high	1950-01-01 – 1960-01-01	Not specified	209	83
**Quaade** [9]	1962	Denmark	high	1949-01-01 – 1959-12-31	Not specified	658	75
**Yu** [10]	1963	Australia	high	1953-07-01 – 1961-12-31	Neonates	47	28
**Gossage** [11]	1964	Canada	high	1942-01-01 – 1962-12-31	Children	255	66
**Heycock** [12]	1964	United Kingdom	high	1950-01-01 – 1962-12-31	Neonates and children	337	29
**Wilson** [13]	1964	United States	high	1942-10-01 – 1963-06-30	Children and adults	94	39
**Schmuziger** [14]	1965	Switzerland	high	1950-01-01 – 1962-12-31	Not specified	122	32
**Swartz** [15]	1965	United States	high	1956-01-01 – 1962-12-31	Not specified	207	49
**Berman** [16]	1966	United States	high	1958-01-01 – 1965-01-01	Neonates	29	28
**Fortuine** [17]	1966	United States	high	1957-01-01 – 1964-12-31	Not specified	69	21
**McNiel** [18]	1966	Saudi Arabia	high	1956-01-01 – 1964-12-31	Neonates and children	91	23
**Donald** [19]	1968	United Kingdom	high	NA - NA	Not specified	210	7

[2] Human Development Index

First author [reference]	Pub. year	Country	HDI[2]	Inclusion period	Age group	Patients, n	Deaths, n
**Fosson [20]**	1968	United States	high	1960-06-01 – 1966-06-01	Neonates	21	16
**Chevrie [21]**	1969	France	high	NA – NA	Neonates	36	22
**Justitz [22]**	1970	Switzerland	high	1955-01-01 – 1966-12-31	Children	240	19
**Overall [23]**	1970	United States	high	1959-01-01 – 1966-12-31	Neonates	25	15
**Seriki [24]**	1970	Nigeria	low	1964-01-01 – 1966-12-31	Neonates and children	156	38
**Jonsson [25]**	1971	Sweden	high	1956-01-01 – 1967-12-31	Not specified	472	89
**Kendall [26]**	1971	Zimbabwe	low	1967-01-01 – 1969-12-31	Neonates and children	197	78
**McDonald [27]**	1972	South Africa	low	1956-01-01 – 1970-12-31	Neonates	82	41
**Wiebe [28]**	1972	United States	high	1965-01-01 – 1965-12-31	Children and adults	194	23
**Fraser [29]**	1973	United States	high	1935-01-01 – 1946-12-31	Not specified	30	20
**Fraser [30]**	1973	United States	high	1961-01-01 – 1971-06-30	Not specified	260	52
**Floyd [31]**	1974	United States	high	1963-01-01 – 1971-12-31	Not specified	389	83
**Fraser [32]**	1974	United States	high	1964-01-01 – 1971-12-31	Not specified	179	42
**Santhanakrishnan [33]**	1974	India	low	1972-01-01 – 1973-12-31	Neonates	69	34
**Chintu [34]**	1975	Zambia	low	1973-08-01 – 1974-07-31	Neonates and children	85	35
**Fraser [35]**	1975	United States	high	1967-01-01 – 1970-12-31	Children	126	10
**Hashemi [36]**	1975	Iran	high	1961-01-01 – 1971-01-01	Children	160	45
**Hodges [37]**	1975	United States	high	1949-01-01 – 1973-12-31	Not specified	349	90
**Kaiser [38]**	1975	Hungary	high	1964-01-01 – 1973-12-31	Children	34	10
**Dawson [39]**	1976	New Zealand	high	NA – NA	Children	53	7
**Goldacre [40]**	1976	United Kingdom	high	1969-01-01 – 1973-12-31	Neonates and children	738	94
**Yeung [41]**	1976	Hong Kong	high	NA – NA	Neonates	20	4

© 2024 van Ettekoven CN et al. *JAMA Network Open.*

First author [reference]	Pub. year	Country	HDI[2]	Inclusion period	Age group	Patients, n	Deaths, n
**Agranat** [42]	1977	Israel	high	1954-01-01 – 1975-12-31	Adults	87	35
**Finland** [43]	1977	United States	high	1947-01-01 – 1972-12-31	Not specified	365	146
**Gilsdorf** [44]	1977	United States	high	1971-07-01 – 1974-06-30	Not specified	39	1
**Lang** [45]	1977	New Zealand	high	1971-09-01 – 1976-09-01	Children	227	20
**Moazami** [46]	1977	Iran	high	1968-01-01 – 1975-01-01	Neonates and children	383	96
**Hailemeskel** [47]	1978	Ethiopia	low	1975-01-01 – 1976-12-31	Children	120	26
**Bieler** [48]	1979	Switzerland	high	1967-05-01 – 1978-05-31	Neonates	22	13
**Chattopadhyay** [49]	1980	United Kingdom	high	1971-01-01 – 1978-12-31	Not specified	48	9
**Geiseler** [50]	1980	United States	high	1954-01-01 – 1976-12-31	Not specified	1,289	95
**Horwitz** [51]	1980	United States	high	1967-01-01 – 1976-12-31	Children	302	10
**Perez-Yarza** [52]	1980	Spain	high	1976-09-01 – 1979-08-31	Children	140	13
**Shann** [53]	1981	Papua New Guinea	low	1977-09-01 – 1979-05-31	Children	73	19
**Wotton** [54]	1981	Canada	high	1972-10-01 – 1977-02-01	Children and adults	37	5
**Davey** [55]	1982	United Kingdom	high	1968-01-01 – 1977-12-31	Not specified	270	30
**Guggenbichler** [56]	1982	Austria	high	1970-01-01 – 1979-12-31	Neonates and children	312	50
**Helwig** [57]	1982	Germany	high	1972-01-01 – 1982-12-31	Children	74	4
**Onile** [58]	1982	Nigeria	low	1976-01-01 – 1979-12-31	Not specified	447	137
**Bohr** [59]	1983	Denmark	high	1966-01-01 – 1976-12-31	Not specified	875	93
**Guirguis** [60]	1983	Egypt	low	1977-01-01 – 1978-12-31	Not specified	350	125
**Ispahani** [61]	1983	United Kingdom	high	1974-01-01 – 1980-06-30	Children and adults	141	28
**Gorse** [62]	1984	United States	high	1970-01-01 – 1982-12-31	Adults	86	28

© 2024 van Ettekoven CN et al. *JAMA Network Open.*

First author [reference]	Pub. year	Country	HDI[2]	Inclusion period	Age group	Patients, n	Deaths, n
**McCracken** [63]	1984	United States	high	1969-01-01 – 1982-12-31	Neonates and children	1,787	114
**Mulder** [64]	1984	Netherlands	high	1976-01-01 – 1982-12-31	Neonates	280	75
**Mulla** [65]	1984	South Africa	low	1979-01-01 – 1980-12-31	Children	358	122
**Rodriguez** [66]	1985	Dominican Rep.	low	NA – NA	Children	100	20
**Schlech** [67]	1985	United States	high	1978-01-01 – 1981-12-31	Not specified	13,974	1,847
**Shann** [68]	1985	Papua New Guinea	low	1979-05-01 – 1983-06-01	Children	367	97
**Skoch** [69]	1985	USA	high	NA - NA	Not specified	130	10
**Valmari** [70]	1985	Finland	high	1978-01-01 – 1978-12-31	Neonates and children	130	5
**Benderly** [71]	1986	Israel	high	1979-01-01 – 1984-12-31	Neonates	17	5
**Jadavji** [72]	1986	Canada	high	1979-01-01 – 1983-06-30	Children	235	15
**Rantakallio** [73]	1986	Finland	high	1966-01-01 – 1981-12-31	Neonates and children	54	8
**Yost** [74]	1986	USA	high	1978-01-01 - 1983-12-31	Neonates and children	101	8
**Bennhagen** [75]	1987	Sweden	high	1976-01-01 – 1983-12-31	Neonates	52	11
**Girgis** [76 77]	1987	Egypt	low	1985-03-01 – 1986-03-31	Children and adults	100	17
**Kilpatrick** [78]	1987	Egypt	low	1979-04-01 – 1980-12-31	Not specified	99	24
**Mir** [79]	1987	Pakistan	low	1985-01-01 – 1985-12-31	Neonates	17	11
**Salwen** [80]	1987	Sweden	high	1956-01-01 – 1975-12-31	Children	201	16
**Zimmerli** [81]	1987	Switzerland	high	1978-01-01 – 1982-12-31	Adults	46	15
**Dawson** [82]	1988	New Zealand	high	1975-01-01 – 1987-12-31	Children	144	2
**Bell** [83]	1989	Ireland	high	1973-01-01 – 1986-12-31	Neonates	41	20
**Bhat** [84]	1989	India	low	1972-01-01 – 1980-12-31	Children	256	78
**Cisse** [85]	1989	Senegal	low	1983-02-01 – 1988-02-01	Neonates and children	409	135

© 2024 van Ettekoven CN et al. *JAMA Network Open.*

First author [reference]	Pub. year	Country	HDI[2]	Inclusion period	Age group	Patients, n	Deaths, n
**Lim [86]**	1989	Singapore	high	1984-01-01 – 1987-12-31	Neonates and children	36	5
**Martinez [87]**	1989	Spain	high	1984-01-01 – 1986-12-31	Adults	200	16
**Rosenthal [88]**	1989	Israel	high	1981-01-01 – 1985-12-31	Neonates and children	107	13
**Sakakihara [89]**	1989	Japan	high	1940-01-01 – 1981-12-31	Children	68	37
**Shaltout [90]**	1989	Kuwait	high	1981-09-01 – 1987-03-31	Neonates and children	92	5
**Bryan [91]**	1990	Brazil	high	1973-01-01 – 1982-12-31	Not specified	3,973	1,312
**Carter [92]**	1990	United Kingdom	high	1946-01-01 – 1987-01-01	Neonates and children	559	39
**Choo [93]**	1990	Malaysia	high	1985-07-01 – 1987-12-31	Children	58	11
**de Bary [94]**	1990	Côte d'Ivoire	low	1985-09-01 – 1986-08-31	Neonates and children	150	49
**Pomeroy [95]**	1990	United States	high	1973-01-01 – 1977-07-01	Children	191	4
**Salih [96]**	1990	Sudan	low	1985-01-01 – 1985-12-31	Children	43	8
**Wenger [97]**	1990	United States	high	1986-01-01 – 1986-12-31	Not specified	2,158	228
**Zaki [98]**	1990	Kuwait	high	1983-07-01 – 1988-06-30	Neonates	45	10
**Zaki [99]**	1990	Kuwait	high	1981-01-01 – 1987-12-31	Neonates and children	107	8
**de Louvois [100]**	1991	United Kingdom	high	1985-09-01 – 1987-08-31	Children	1,430	78
**Dufour [101]**	1991	Switzerland	high	1980-01-01 – 1986-12-31	Adults	104	29
**Hanna [102]**	1991	Australia	high	1984-01-01 – 1988-12-31	Children	270	16
**Kabra [103]**	1991	India	low	1989-01-01 – 1989-12-31	Neonates and children	852	137
**Minutillo [104]**	1991	Australia	high	1975-01-01 – 1989-12-01	Neonates	43	9
**Nathoo [105]**	1991	Zimbabwe	low	1987-01-01 – 1988-12-31	Neonates	94	39
**Olanrewaju [106]**	1991	Nigeria	low	1986-01-01 – 1987-12-31	Neonates and children	48	12

First author [reference]	Pub. year	Country	HDI[2]	Inclusion period	Age group	Patients, n	Deaths, n
**Pecoul** [107]	1991	Multinational	low	1989-05-01 – 1990-06-01	Children and adults	528	157
**Al-Jurayyan** [108]	1992	Saudi Arabia	high	1982-06-01 – 1990-08-31	Children	71	5
**Craig** [109]	1992	New Zealand	high	1987-01-01 – 1991-06-30	Children	62	3
**Francis** [110]	1992	Australia	high	1987-01-01 – 1989-12-31	Neonates	115	30
**Franco** [111]	1992	USA	high	1993-01-01 - 2002-12-31	Neonates	26	5
**Mackie** [112]	1992	Ghana	low	1989-08-01 – 1990-03-31	Children	69	19
**Pallangyo** [113]	1992	Tanzania	low	1989-05-01 – 1990-12-01	Children and adults	78	19
**Rasmussen** [114]	1992	Denmark	high	1976-01-01 – 1988-01-01	Adults	42	12
**Rothrock** [115]	1992	United States	high	1979-01-01 – 1990-07-01	Neonates and children	258	23
**Shattuck** [116]	1992	United States	high	1974-01-01 – 1989-01-01	Neonates	98	17
**Srair** [117]	1992	Saudi Arabia	high	1988-01-01 – 1991-12-31	Children	50	6
**Tefuarani** [118]	1992	Papua New Guinea	low	1989-05-01 – 1990-05-01	Neonates and children	108	18
**Thomas** [119]	1992	Australia	high	1979-01-01 – 1989-12-31	Neonates and children	80	5
**Airede** [120]	1993	Nigeria	low	1988-01-01 – 1990-01-01	Neonates	36	12
**Ballantyne** [121]	1993	United Kingdom	high	1964-01-01 – 1991-12-01	Not specified	97	19
**Boehme** [122]	1993	Chile	high	1988-01-01 – 1991-12-31	Children	90	12
**Brivet** [123]	1993	France	high	1981-01-01 – 1992-12-01	Adults	80	31
**Durand** [124]	1993	USA	high	1962-01-01 - 1988-12-31	Adults	253	63
**Fortnum** [125]	1993	United Kingdom	high	1980-01-01 – 1989-12-01	Children	262	25
**Liu** [126]	1993	Taiwan	high	1988-11-01 – 1992-10-31	Children	41	7
**Pfister** [127]	1993	Germany	high	1984-01-01 – 1989-12-31	Adults	86	15

© 2024 van Ettekoven CN et al. *JAMA Network Open.*

First author [reference]	Pub. year	Country	HDI[2]	Inclusion period	Age group	Patients, n	Deaths, n
**Ara [128]**	1994	Spain	high	1985-01-01 – 1988-12-31	Not specified	340	35
**Carroll [129]**	1994	Vanuatu	low	1988-08-01 – 1991-07-31	Not specified	83	13
**Chotpitayasunondh [130]**	1994	Thailand	high	1980-01-01 – 1990-12-31	Neonates and children	618	129
**Commey [131]**	1994	Ghana	low	1991-11-01 – 1993-03-31	Children	103	22
**Dagan [132]**	1994	Israel	high	1988-10-01 – 1991-09-30	Neonates and children	467	18
**Ford [133]**	1994	South Africa	low	1991-02-01 – 1992-07-01	Not specified	85	33
**Kallio [134]**	1994	Finland	high	1984-01-01 – 1991-12-31	Children	325	12
**Moreno [135]**	1994	Panama	high	1975-01-01 – 1992-12-01	Neonates	107	37
**Ozumba [136]**	1994	Nigeria	low	1989-04-01 – 1993-03-31	Neonates and children	76	22
**Synnott [137]**	1994	United Kingdom	high	1975-01-01 – 1991-12-01	Neonates	1,846	249
**Ali [138]**	1995	Trinidad and Tobago	high	1988-01-01 – 1990-12-31	Neonates	50	7
**Almirante [139]**	1995	Spain	high	1985-01-01 – 1994-12-31	Adults	210	39
**Ciana [140]**	1995	Mozambique	low	1989-03-01 – 1989-10-31	Children	70	20
**Daoud [141]**	1995	Jordan	high	1990-01-01 – 1992-12-31	Children	118	13
**Gedlu [142]**	1995	Ethiopia	low	1990-01-01 – 1994-12-31	Children	132	37
**Kaaresen [143]**	1995	Norway	high	1980-01-01 – 1993-12-31	Children	92	4
**Kilpi [144]**	1995	Finland	high	1987-03-01 - 1991-02-01	Children	122	2
**Lecour [145]**	1995	Portugal	high	1981-01-01 – 1994-12-01	Neonates and children	256	16
**Lutsar [146]**	1995	Estonia	high	1983-01-01 – 1990-12-31	Children	84	3
**Patwari [147]**	1995	India	low	1987-05-01 – 1988-06-30	Children	60	16
**Salaun-Saraux [148]**	1995	Rwanda	low	1983-01-01 – 1990-12-31	Neonates and children	262	99

© 2024 van Ettekoven CN et al. *JAMA Network Open.*

First author [reference]	Pub. year	Country	HDI[2]	Inclusion period	Age group	Patients, n	Deaths, n
**Singhi [149]**	1995	India	low	NA – NA	Children	50	9
**Ahmed [150]**	1996	Sudan	low	1989-04-15 – 1990-10-15	Children	56	16
**Berg [151]**	1996	Sweden	high	1987-01-01 – 1989-12-31	Not specified	921	85
**Bergemann [152]**	1996	South Africa	low	1994-05-01 – 1995-04-01	Children and adults	64	27
**Gomes [153]**	1996	Brazil	high	1993-04-01 – 1993-12-31	Neonates and children	276	56
**Ichiyama [154]**	1996	Japan	high	1984-01-01 – 1994-12-01	Neonates and children	13	1
**Ishikawa [155]**	1996	Japan	high	1984-01-01 – 1993-12-01	Neonates and children	320	24
**Laguna [156]**	1996	Spain	high	1982-01-01 – 1995-06-30	Adults	77	5
**Macaluso [157]**	1996	Brazil	high	1991-01-01 – 1992-12-31	Children	179	34
**Qazi [158]**	1996	Pakistan	low	1990-04-01 – 1992-03-01	Children	89	17
**Awasthi [159]**	1997	India	low	NA – NA	Children	97	18
**Grobler [160]**	1997	South Africa	low	1990-01-01 – 1995-12-31	Children	61	12
**Hussey [161]**	1997	South Africa	low	1991-08-01 – 1992-07-01	Children	201	10
**Imuekehme [162]**	1997	Nigeria	low	1988-03-01 – 1988-10-31	Children	40	4
**Sigurdardottir [163]**	1997	Iceland	high	1975-01-01 – 1994-12-01	Adults	127	25
**Sung [164]**	1997	Hong Kong	high	1984-12-01 – 1993-11-01	Children	41	2
**Chang [165]**	1998	Taiwan	high	1989-01-01 – 1995-12-01	Children	101	27
**Fernandez-Jaen [166]**	1998	Spain	high	1986-01-01 – 1995-12-31	Children	166	6
**Gutierrez [167]**	1998	Spain	high	1995-03-21 – 1997-03-21	Children and adults	40	4
**Honnas [168]**	1998	Kenya	low	1996-09-01 – 1996-12-31	Children and adults	32	12
**Hussain [169]**	1998	Malaysia	high	1995-01-01 – 1995-12-31	Children	71	9
**Imananagha [170]**	1998	Nigeria	low	1991-01-01 – 1994-12-31	Children	62	14

© 2024 van Ettekoven CN et al. *JAMA Network Open.*

First author [reference]	Pub. year	Country	HDI[2]	Inclusion period	Age group	Patients, n	Deaths, n
**Kim** [171]	1998	Korea	high	1986-01-01 – 1995-12-01	Children	140	17
**Molyneux** [172]	1998	Malawi	low	1996-04-01 – 1997-03-01	Neonates and children	260	104
**Müller** [173]	1998	Germany	high	1992-06-01 – 1996-12-31	Adults	47	8
**Rios-Reategui** [174]	1998	Mexico	high	1990-01-01 – 1995-07-31	Neonates	37	9
**Schutte** [175]	1998	South Africa	low	NA – NA	Adults	33	7
**Shembesh** [176]	1998	Libya	high	1994-04-01 – 1995-05-31	Children	77	10
**Campagne** [177]	1999	Niger	low	1989-01-01 – 1996-12-31	Not specified	4,177	641
**Daoud** [178]	1999	Jordan	high	1993-01-01 – 1995-10-01	Neonates	52	12
**Dawson** [179]	1999	United States	high	NA - NA	Neonates and children	793	27
**Moyen** [180]	1999	Congo	low	1994-12-01 – 1996-12-01	Children	138	60
**Nathoo** [181]	1999	Zimbabwe	low	1995-06-01 – 1996-05-31	Neonates and children	125	41
**Okome** [182]	1999	Gabon	high	1991-01-01 – 1995-12-31	Adults	85	15
**Palmer** [183]	1999	Gambia	low	1991-01-01 – 1994-12-31	Neonates and children	420	111
**Pena** [184]	1999	Venezuela	high	1996-01-01 – 1998-12-31	Children	152	12
**Silber** [185]	1999	South Africa	low	1996-07-01 – 1996-11-01	Adults	12	2
**Struillou** [186]	1999	France	high	1995-05-01 – 1998-04-30	Children and adults	100	17
**Tang** [187]	1999	Taiwan	high	1981-01-01 – 1998-06-01	Adults	263	70
**Chotmongkol** [188]	2000	Thailand	high	1984-01-01 – 1998-12-31	Adults	71	26
**Gordon** [189]	2000	Malawi	low	1998-04-01 – 1999-03-31	Adults	248	123
**Klinger** [190]	2000	Canada	high	1979-01-01 – 1998-12-01	Neonates	101	13
**Moller** [191]	2000	Denmark	high	1997-02-01 – 1999-01-01	Adults	12	1

© 2024 van Ettekoven CN et al. *JAMA Network Open.*

First author [reference]	Pub. year	Country	HDI[2]	Inclusion period	Age group	Patients, n	Deaths, n
**Nel [192]**	2000	South Africa	low	1981-07-01 – 1992-06-01	Neonates	88	30
**Ray [193]**	2000	India	low	NA – NA	Children	32	14
**Zanelli [194]**	2000	France	high	1982-01-01 – 1997-12-31	Neonates	35	4
**Almuneef [195]**	2001	Saudi Arabia	high	NA - NA	Neonates and children	76	4
**Berkley [196]**	2001	Kenya	low	1999-03-01 – 2000-02-29	Children	45	15
**Bonsu [197]**	2001	United States	high	1984-01-01 – 1996-12-31	Children	288	6
**Holt [198]**	2001	United Kingdom	high	1996-07-01 – 1997-12-31	Neonates	144	14
**Johnson [199]**	2001	Nigeria	low	1992-01-01 – 1996-12-31	Neonates and children	71	30
**Madhi [200]**	2001	South Africa	low	1997-03-01 – 1999-02-01	Children	147	29
**McMillan [201]**	2001	USA	high	1970-01-01 - 1998-12-31	Adults	294	77
**Miner [202]**	2001	United States	high	1987-06-01 – 1997-06-01	Not specified	118	9
**Neuman [203]**	2001	United States	high	1988-01-01 – 1998-12-31	Children	216	12
**Sahai [204]**	2001	India	low	1994-09-01 – 1996-04-01	Children	100	25
**Weiss [205]**	2001	Brazil	high	1997-01-01 – 1998-12-31	Not specified	274	24
**Ahsan [206]**	2002	Pakistan	low	1998-05-01 – 2000-04-30	Children and adults	68	6
**Barboza [207]**	2002	Argentina	high	1988-01-01 – 1998-12-31	Adults	87	19
**Beyrer [208]**	2002	Germany	high	1998-07-01 – 2000-12-31	Not specified	256	20
**Chan [209]**	2002	Singapore	high	1993-01-01 – 2000-12-31	Adults	26	5
**Chinchankar [210]**	2002	India	low	1997-04-01 – 1999-03-01	Children	54	17
**Duke [211]**	2002	Papua New Guinea	low	1997-09-01 – 2000-10-31	Children	346	59
**Hemalatha [212]**	2002	India	low	1998-01-01 – 2000-01-01	Children	120	20

© 2024 van Ettekoven CN et al. *JAMA Network Open.*

First author [reference]	Pub. year	Country	HDI[2]	Inclusion period	Age group	Patients, n	Deaths, n
**Lopez [213]**	2002	Spain	high	1997-01-01 – 1998-12-31	Neonates	82	7
**Migliani [214]**	2002	Madagascar	low	1998-06-11 – 2000-06-09	Children	83	26
**Molyneux [215]**	2002	Malawi	low	1997-07-01 – 2001-03-31	Children	598	187
**Mwangi [216]**	2002	Kenya	low	1994-01-01 – 1998-06-30	Neonates and children	223	74
**Oostenbrink [217]**	2002	Netherlands	high	1988-01-01 – 1998-12-31	Children	170	2
**Al-Mazrou [218]**	2003	Saudi Arabia	high	1999-06-01 – 2001-05-01	Neonates and children	203	5
**Chang [219]**	2003	Taiwan	high	1986-01-01 – 2001-12-31	Neonates	60	6
**Flores-Cordero [220]**	2003	Spain	high	1995-01-01 – 2000-12-31	Adults	108	9
**Kirimi [221]**	2003	Turkey	high	1999-02-01 – 2000-12-31	Children	48	6
**Rabbani [222]**	2003	Pakistan	low	1995-01-01 – 2002-07-01	Adults	192	43
**Celal [223]**	2004	Turkey	high	1996-06-01 – 2002-12-01	Adults	186	29
**Khwannimit [224]**	2004	Thailand	high	1982-01-01 – 2001-12-31	Adults	74	11
**Luca [225]**	2004	Romania	high	2000-03-01 – 2002-03-31	Neonates and children	56	3
**Ostergaard [226]**	2004	Denmark	high	1988-01-01 – 2002-12-31	Children and adults	54	8
**Sallam [227]**	2004	Yemen	low	2001-01-01 – 2002-08-23	Neonates and children	20	3
**Singhi [228]**	2004	India	low	1993-07-01 – 1996-12-01	Children	222	19
**van de Beek [229]**	2004	Netherlands	high	1998-10-01 – 2002-04-30	Adults	696	143
**Wiersinga [230]**	2004	Tanzania	low	1999-04-01 – 2002-03-31	Children	130	66
**Amsalu [231]**	2005	Ethiopia	low	1998-09-01 – 2003-08-01	Children	151	20
**Bekondi [232]**	2005	Central African Rep.	low	1999-04-01 – 2003-01-01	Adults	60	40
**Deeks [233]**	2005	Canada	high	1994-04-01 – 2001-03-31	Not specified	7,227	813

© 2024 van Ettekoven CN et al. *JAMA Network Open.*

First author [reference]	Pub. year	Country	HDI[2]	Inclusion period	Age group	Patients, n	Deaths, n
**Farag** [234]	2005	Egypt	high	NA - NA	Children	202	28
**Hui** [235]	2005	Hong Kong	high	1992-01-01 – 2001-12-31	Adults	35	11
**Lucena** [236]	2005	Brazil	high	1997-03-01 – 1997-12-31	Children	83	14
**May** [237]	2005	Australia	high	1992-01-01 – 2002-12-31	Neonates	78	11
**Odetola** [238]	2005	United States	high	1995-01-01 – 2000-12-31	Children	334	34
**Ogunlesi** [239]	2005	Nigeria	low	1998-01-01 – 2003-12-31	Children	124	33
**Al Khoransani** [240]	2006	Yemen	low	1999-05-01 – 2001-06-30	Children	160	16
**Bregani** [241]	2006	Chad	low	2001-01-01 – 2001-04-01	Not specified	595	52
**Elsaid** [242]	2006	Qatar	high	1998-01-01 – 2002-12-31	Neonates and children	64	1
**Garges** [243]	2006	United States	high	1997-01-01 – 2004-12-31	Neonates and children	73	4
**Mbelesso** [244]	2006	Central African Rep.	low	1998-07-01 – 2003-06-01	Adults	502	160
**Molyneux** [245]	2006	United Kingdom	high	1984-01-01 – 1991-12-31	Neonates and children	197	14
**Pizon** [246]	2006	United States	high	1992-11-01 – 2003-03-31	Adults	38	7
**Shabani** [247]	2006	Kuwait	high	2001-01-01 – 2001-12-31	Children	42	1
**Singhi** [248]	2006	India	low	NA – NA	Children	16	1
**Afifi** [249]	2007	Egypt	low	1998-06-01 – 2004-07-31	Children and adults	1,101	239
**Boisier** [250]	2007	Niger	low	2003-01-01 – 2006-12-31	Not specified	1,935	304
**Dauchy** [251]	2007	France	high	2001-01-01 – 2004-12-31	Adults	60	13
**Faustini** [252]	2007	Italy	high	1996-01-01 – 2000-12-31	Not specified	525	98
**Johnson** [253]	2007	Nigeria	low	NA – NA	Neonates and children	62	28
**Krebs** [254]	2007	Brazil	high	1994-01-01 – 2004-12-31	Neonates	87	10
**Lepur** [255]	2007	Croatia	high	1990-01-01 – 2004-12-31	Adults	286	65

© 2024 van Ettekoven CN et al. *JAMA Network Open.*

First author [reference]	Pub. year	Country	HDI[2]	Inclusion period	Age group	Patients, n	Deaths, n
**Nguyen [256]**	2007	Vietnam	low	1996-11-01 – 2005-06-30	Children and adults	300	31
**Peltola [257]**	2007	Multinational	low	1996-01-01 – 2003-12-31	Children	654	86
**Scarborough [258]**	2007	Malawi	low	2002-05-01 – 2005-01-31	Adults	322	162
**Theodoridou [259]**	2007	Greece	high	1974-01-01 – 2005-12-31	Children	1,331	34
**Airede [260]**	2008	Nigeria	low	1992-01-01 – 1995-12-31	Neonates	50	17
**Bercion [261]**	2008	Central African Rep.	low	2004-10-01 – 2005-09-30	Neonates and children	130	51
**Franco [262]**	2008	Mexico	high	1993-01-01 – 2002-12-31	Children and adults	218	36
**Lagunju [263]**	2008	Nigeria	low	2004-05-01 – 2007-03-01	Children	97	26
**Lazzarini [264]**	2008	Italy	high	2002-10-01 – 2005-06-30	Adults	289	20
**Mongelluzzo [265]**	2008	United States	high	2001-01-01 – 2006-12-31	Neonates and children	2,780	117
**Pelkonen [266]**	2008	Angola	low	2004-01-01 – 2004-12-31	Children	403	133
**Sigauque [267]**	2008	Mozambique	low	1998-07-01 – 2003-11-30	Neonates and children	65	23
**Cabellos [268]**	2009	Spain	high	1977-01-01 – 2006-12-31	Adults	675	124
**Dzupova [269]**	2009	Czech Rep.	high	1997-01-01 – 2006-12-31	Adults	279	55
**Gurley [270]**	2009	Bangladesh	low	2003-06-01 – 2005-07-01	Not specified	189	26
**Ishihara [271]**	2009	Japan	high	1998-04-01 – 2007-12-31	Adults	71	16
**Roca [272]**	2009	Mozambique	low	2006-01-09 – 2007-01-08	Neonates and children	43	11
**Tiskumara [273]**	2009	Multinational	low	2005-01-01 – 2005-12-31	Neonates	76	15
**Traore [274]**	2009	Multinational	low	2002-04-01 – 2006-12-31	Not specified	873	262
**Abdulrab [275]**	2010	Yemen	low	2006-01-01 – 2007-12-31	Adults	59	16
**Aletayeb [276]**	2010	Iran	high	1997-01-01 – 2007-12-31	Neonates	14	2

© 2024 van Ettekoven CN et al. *JAMA Network Open.*

First author [reference]	Pub. year	Country	HDI[2]	Inclusion period	Age group	Patients, n	Deaths, n
**Ba** [277]	2010	Senegal	low	2006-01-01 – 2008-12-31	Neonates and children	206	79
**Bentlin** [278]	2010	Brazil	high	1997-01-01 – 2006-12-31	Neonates	22	6
**Cho** [279]	2010	Korea	high	1996-01-01 – 2005-12-31	Neonates and children	402	38
**Erdem** [280]	2010	Turkey	high	2001-01-01 – 2008-12-31	Adults	159	34
**Hudeckova** [281]	2010	Slovakia	high	1997-01-01 – 2007-12-31	Not specified	1,210	148
**Mankhambo** [282]	2010	Malawi	low	2004-04-01 – 2006-10-30	Children	211	58
**Moon** [283]	2010	Korea	high	1998-01-01 – 2008-12-31	Adults	172	33
**Perez** [284]	2010	Cuba	high	1998-01-01 – 2007-12-31	Not specified	4,798	1,157
**Su** [285]	2010	Taiwan	high	1986-01-01 – 2007-12-31	Adults	217	90
**Talbert** [286]	2010	Kenya	low	2001-01-01 – 2009-12-31	Neonates	152	29
**Vibha** [287]	2010	India	low	2004-06-01 – 2008-09-30	Children and adults	380	34
**Ajdukiewicz** [288]	2011	Malawi	low	2006-09-10 – 2008-08-23	Adults	116	64
**Pelkonen** [289]	2011	Angola	low	2005-07-18 – 2008-06-26	Children	723	272
**Thigpen** [290]	2011	United States	high	NA - NA	Not specified	3,157	466
**Vashishtha** [291]	2011	India	low	2009-01-01 – 2010-12-31	Children	67	7
**World Health Organization** [292]	2011	Multinational	low	2010-01-01 – 2010-12-31	Not specified	22,831	2,415
**Fonseca de Souza** [293]	2012	Brazil	high	2004-01-01 – 2009-12-31	Not specified	1,049	168
**Juganariu** [294]	2012	Romania	high	2005-01-01 – 2010-12-31	Neonates and children	100	5
**Kra** [295]	2012	Côte d'Ivoire	low	2004-01-01 – 2004-12-31	Adults	15	5
**Namani** [296]	2012	Kosovo	low	1997-01-01 – 2002-12-31	Neonates and children	257	12
**Nansera** [297]	2012	Uganda	low	2003-04-01 – 2008-12-31	Neonates and children	51	23

© 2024 van Ettekoven CN et al. *JAMA Network Open.*

First author [reference]	Pub. year	Country	HDI[2]	Inclusion period	Age group	Patients, n	Deaths, n
**Tarvij Eslami [298]**	2012	Iran	high	2005-01-01 – 2007-12-31	Neonates	60	10
**Vazquez [299]**	2012	Argentina	high	2009-05-01 – 2009-10-31	Adults	13	3
**Ben Hamouda [300]**	2013	Tunisia	high	1996-01-01 – 2010-12-31	Neonates	44	7
**Butsashvili [301]**	2013	Georgia	high	2006-01-01 – 2010-12-31	Not specified	100	9
**Ergaz [302]**	2013	Israel	high	1993-01-01 – 2009-08-31	Neonates	109	8
**Kavuncuoglu [303]**	2013	Turkey	high	2003-01-01 – 2010-06-30	Neonates	325	8
**Khowaja [304]**	2013	Pakistan	low	2008-07-01 – 2011-12-31	Neonates and children	188	64
**Mahmoudi [305]**	2013	Iran	high	2009-08-01 – 2011-02-28	Children	20	2
**Porobic [306]**	2013	Bosnia and Herz.	high	1999-05-01 – 2009-06-01	Children	140	3
**Scott [307]**	2013	Mongolia	high	2002-01-01 – 2010-12-31	Children	254	24
**Snaebjarnardottir [308]**	2013	Iceland	high	1975-01-01 – 2010-12-31	Neonates and children	477	21
**Teleb [309]**	2013	Multinational	low	2004-01-01 – 2010-12-31	Children	1,263	121
**Banajeh [310]**	2014	Yemen	low	2000-01-01 – 2012-12-31	Children	2,280	165
**Bodilsen [311]**	2014	Denmark	high	1998-01-01 – 2012-12-31	Adults	172	33
**Levy [312]**	2014	France	high	2001-01-01 – 2012-12-31	Neonates and children	4,808	414
**Molyneux [313]**	2014	Malawi	low	2008-03-01 – 2012-03-31	Children and adults	360	93
**Namani [314]**	2014	Kosovo	high	2000-01-01 – 2010-12-31	Children and adults	83	8
**Okike [315]**	2014	United Kingdom	high	2010-07-01 – 2011-07-31	Neonates	282	22
**Thornorethardottir [316]**	2014	Iceland	high	1995-01-01 – 2010-12-31	Adults	111	18
**Correa-Lima [317]**	2015	Brazil	high	2004-01-01 – 2008-12-31	Children	270	34
**Hu [318]**	2015	China	high	2012-03-01 – 2013-03-01	Children	25	4

© 2024 van Ettekoven CN et al. *JAMA Network Open.*

First author [reference]	Pub. year	Country	HDI[2]	Inclusion period	Age group	Patients, n	Deaths, n
**Kamoun** [319]	2015	Tunisia	high	1990-01-01 – 2012-12-31	Neonates	55	22
**Lin** [320]	2015	Taiwan	high	1984-01-01 – 2012-12-31	Neonates and children	291	50
**Mora Mora** [321]	2015	Argentina	high	2003-01-01 – 2013-01-01	Adults	69	28
**Olson** [322]	2015	Guatemala	low	2000-01-01 – 2007-12-31	Neonates and children	800	192
**Shresta** [323]	2015	Nepal	low	2012-05-01 – 2013-04-30	Neonates and children	18	6
**Softic** [324]	2015	Bosnia and Herz.	high	2012-07-01 – 2013-06-30	Neonates	18	2
**Tan** [325]	2015	China	high	2008-01-01 – 2014-06-01	Neonates	232	7
**Bari** [326]	2016	Pakistan	low	2012-01-01 – 2012-12-31	Children	199	20
**Baunbaek** [327]	2016	Denmark	high	2003-01-01 – 2010-12-31	Adults	147	49
**Coldiron** [328]	2016	Niger	low	2015-01-01 – 2015-06-30	Not specified	473	70
**Glimaker** [329]	2016	Sweden	high	1995-01-01 – 2014-12-31	Adults	1,746	214
**Kambire** [330]	2016	Burkina Faso	low	2011-01-01 – 2013-12-31	Not specified	2,858	472
**Wee** [331]	2016	Singapore	high	1998-01-01 – 2013-05-31	Neonates and children	112	7
**Gudina** [332]	2017	Ethiopia	low	2013-03-01 – 2015-12-31	Adults	64	15
**Hasbun** [333]	2017	United States	high	2011-01-01 – 2014-12-31	Adults	3,692	299
**Kaburi** [334]	2017	Ghana	low	2010-01-01 – 2015-12-31	Not specified	83	10
**Kafle** [335]	2017	Nepal	low	2015-04-01 – 2016-03-01	Not specified	21	1
**Lien** [336]	2017	Taiwan	high	2006-01-01 – 2015-12-31	Adults	50	20
**Ouchenir** [337]	2017	Canada	high	NA - NA	Neonates	113	8
**Park** [338]	2017	Korea	high	2009-01-01 – 2016-05-01	Not specified	80	13
**Polkowska** [339]	2017	Finland	high	2004-01-01 – 2014-12-31	Not specified	633	65

© 2024 van Ettekoven CN et al. *JAMA Network Open.*

First author [reference]	Pub. year	Country	HDI[2]	Inclusion period	Age group	Patients, n	Deaths, n
**Sadeq** [340]	2017	Kuwait	high	2010-01-01 – 2014-12-31	Children	57	5
**Wall** [341]	2017	Malawi	low	2012-01-01 – 2013-10-31	Adults	117	66
**Amare** [342]	2018	Ethiopia	low	2011-09-01 – 2013-09-01	Children	80	6
**Jumanne** [343]	2018	Tanzania	low	2011-11-01 – 2012-04-01	Children	13	5
**Kumar** [344]	2018	India	low	NA – NA	Neonates	89	10
**Brown** [345]	2019	United States	high	2015-07-01 – 2016-06-30	Adults	14	1
**De Almeida** [346]	2019	Brazil	high	2006-01-01 – 2017-12-31	Not specified	49	9
**El-Naggar** [347]	2019	Canada	high	NA - NA	Neonates	246	31
**Fuentes-Antras** [348]	2019	Spain	high	2007-01-01 – 2014-12-31	Adults	79	12
**Haydar** [349]	2019	Lebanon	high	2008-01-01 – 2016-12-31	Not specified	46	4
**Larsen** [350]	2019	Denmark	high	2015-01-01 – 2017-12-31	Adults	379	50
**Mwenda** [351]	2019	Multinational	low	2011-01-01 – 2016-12-31	Children	1,670	186
**Pruitt** [352]	2019	United States	high	2011-07-01 – 2016-06-30	Neonates	71	2
**Sonko** [353]	2019	Senegal	low	2010-01-01 – 2016-12-01	Neonates and children	115	18
**Tagbo** [354]	2019	Nigeria	low	2010-01-01 – 2016-12-01	Neonates and children	153	23
**Tsolenyanu** [355]	2019	Togo	low	2010-01-01 – 2016-12-01	Neonates and children	98	21
**Huang** [356]	2020	Taiwan	high	2007-01-01 – 2013-12-31	Neonates	12	0
**Johansson** [357]	2020	Sweden	high	1986-01-01 – 2015-12-01	Children	101	6
**Liu** [358]	2020	China	high	2016-01-01 – 2018-01-01	Neonates	111	5
**Loutfi** [359]	2020	Morocco	low	2014-01-01 – 2018-12-31	Not specified	35	10
**Matulyte** [360]	2020	Lithuania	high	2009-01-01 – 2016-12-01	Adults	159	9
**Peros** [361]	2020	Netherlands	high	2004-01-01 – 2016-01-01	Neonates	45	15

© 2024 van Ettekoven CN et al. *JAMA Network Open.*

First author [reference]	Pub. year	Country	HDI[2]	Inclusion period	Age group	Patients, n	Deaths, n
**Pomar** [362]	2020	Spain	high	1982-01-01 – 2017-12-01	Adults	715	124
**Tubiana** [363]	2020	France	high	2013-02-01 – 2015-07-01	Adults	533	90
**Adil** [364]	2021	United States	high	2008-01-01 – 2015-12-31	Neonates and children	1,632	62
**Aimbudlop** [365]	2021	Thailand	high	2013-01-01 – 2017-12-01	Adults	28	3
**Bumburidi** [366]	2021	Kazakhstan	high	2017-02-01 – 2018-01-31	Not specified	37	2
**Kumar** [367]	2021	India	low	NA - NA	Adults	39	17
**Pelkonen** [368]	2021	Angola	low	2016-02-01 – 2017-10-23	Neonates	139	34
**Salmanov** [369]	2021	Ukraine	high	2017-01-01 – 2019-12-31	Neonates	86	28
**Savonius** [370]	2021	Angola	low	2012-01-22 – 2017-01-21	Children	375	148
**Sunwoo** [371]	2021	Korea	high	2007-01-01 – 2016-12-01	Adults	43	5
**Wong** [372]	2021	Hong Kong	high	2004-01-01 – 2019-12-31	Neonates	139	4

© 2024 van Ettekoven CN et al. *JAMA Network Open.*

eTable 5. Proportions of the causative pathogens, stratified by mean study periods.

Interval	Pathogen	Deaths	Episodes	Proportion per time interval [%]
**Before 1961**	*S. pneumoniae*	316	870	18.6
**1961–1970**	*S. pneumoniae*	256	846	18.5
**1971–1980**	*S. pneumoniae*	1,137	3,783	15.3
**1981–1990**	*S. pneumoniae*	620	3,045	17.3
**1991–2000**	*S. pneumoniae*	1,046	5,117	25.4
**2001–2010**	*S. pneumoniae*	1,484	9,145	35.5
**After 2010**	*S. pneumoniae*	643	3,229	46.1
**Before 1961**	*N. meningitidis*	143	1,474	31.5
**1961–1970**	*N. meningitidis*	112	1,297	28.3
**1971–1980**	*N. meningitidis*	456	4,777	19.3
**1981–1990**	*N. meningitidis*	183	3,449	19.6
**1991–2000**	*N. meningitidis*	475	6,090	30.3
**2001–2010**	*N. meningitidis*	299	7,015	27.2
**After 2010**	*N. meningitidis*	180	2,242	32.0
**Before 1961**	*H. influenzae*	65	667	14.2
**1961–1970**	*H. influenzae*	104	1,388	30.3
**1971–1980**	*H. influenzae*	865	10,150	41.0
**1981–1990**	*H. influenzae*	287	4,777	27.1
**1991–2000**	*H. influenzae*	405	3,322	16.5
**2001–2010**	*H. influenzae*	347	2,003	7.8
**After 2010**	*H. influenzae*	30	415	5.9
**Before 1961**	*L. monocytogenes*	6	18	0.4
**1961–1970**	*L. monocytogenes*	5	22	0.5
**1971–1980**	*L. monocytogenes*	80	329	1.3
**1981–1990**	*L. monocytogenes*	42	329	1.9
**1991–2000**	*L. monocytogenes*	11	231	1.1
**2001–2010**	*L. monocytogenes*	34	281	1.1
**After 2010**	*L. monocytogenes*	10	55	0.8
**Before 1961**	*E. coli*	64	111	2.4
**1961–1970**	*E. coli*	37	73	1.6
**1971–1980**	*E. coli*	52	193	0.8
**1981–1990**	*E. coli*	60	818	4.6
**1991–2000**	*E. coli*	32	265	1.3
**2001–2010**	*E. coli*	7	521	2.0
**After 2010**	*E. coli*	2	63	0.9
**Before 1961**	*S. agalactiae*	8	13	0.3
**1961–1970**	*S. agalactiae*	9	15	0.3
**1971–1980**	*S. agalactiae*	154	733	3.0
**1981–1990**	*S. agalactiae*	121	1,267	7.2
**1991–2000**	*S. agalactiae*	29	499	2.5
**2001–2010**	*S. agalactiae*	73	1,669	6.5
**After 2010**	*S. agalactiae*	7	97	1.4

© 2024 van Ettekoven CN et al. *JAMA Network Open.*

Interval	Pathogen	Deaths	Episodes	Proportion per time interval [%]
**Before 1961**	*S. aureus*	48	102	2.2
**1961–1970**	*S. aureus*	9	25	0.5
**1971–1980**	*S. aureus*	6	32	0.1
**1981–1990**	*S. aureus*	33	185	1.0
**1991–2000**	*S. aureus*	21	137	0.7
**2001–2010**	*S. aureus*	14	76	0.3
**After 2010**	*S. aureus*	0	0	0
**Before 1961**	*Enterobacter* spp.	0	1	0
**1961–1970**	*Enterobacter* spp.	0	0	0
**1971–1980**	*Enterobacter* spp.	117	137	0.6
**1981–1990**	*Enterobacter* spp.	7	47	0.3
**1991–2000**	*Enterobacter* spp.	13	35	0.2
**2001–2010**	*Enterobacter* spp.	0	7	0
**After 2010**	*Enterobacter* spp.	0	0	0
**Before 1961**	*Pseudomonas* spp.	13	17	0.4
**1961–1970**	*Pseudomonas* spp.	5	6	0.1
**1971–1980**	*Pseudomonas* spp.	18	25	0.1
**1981–1990**	*Pseudomonas* spp.	18	73	0.4
**1991–2000**	*Pseudomonas* spp.	8	28	0.1
**2001–2010**	*Pseudomonas* spp.	3	12	0
**After 2010**	*Pseudomonas* spp.	0	0	0
**Before 1961**	*K. pneumoniae*	2	3	0.1
**1961–1970**	*K. pneumoniae*	2	3	0.1
**1971–1980**	*K. pneumoniae*	1	6	0
**1981–1990**	*K. pneumoniae*	14	111	0.6
**1991–2000**	*K. pneumoniae*	20	47	0.2
**2001–2010**	*K. pneumoniae*	6	26	0.1
**After 2010**	*K. pneumoniae*	0	1	0
**Before 1961**	Other bacteria	190	408	8.7
**1961–1970**	Other bacteria	124	407	8.9
**1971–1980**	Other bacteria	711	2,368	9.6
**1981–1990**	Other bacteria	303	1,638	9.3
**1991–2000**	Other bacteria	283	1,796	8.9
**2001–2010**	Other bacteria	203	3,550	13.8
**After 2010**	Other bacteria	12	250	3.6
**Before 1961**	Unidentified	170	1,000	21.3
**1961–1970**	Unidentified	75	502	11.0
**1971–1980**	Unidentified	407	2,246	9.1
**1981–1990**	Unidentified	175	1,896	10.8
**1991–2000**	Unidentified	308	2,564	12.7
**2001–2010**	Unidentified	191	1,481	5.7
**After 2010**	Unidentified	18	647	9.2
**Before 1961**	Total	1,025	4,684	100

© 2024 van Ettekoven CN et al. *JAMA Network Open.*

Interval	Pathogen	Deaths	Episodes	Proportion per time interval [%]
**1961–1970**	Total	738	4,584	100
**1971–1980**	Total	4,004	24,779	100
**1981–1990**	Total	1,863	17,635	100
**1991–2000**	Total	2,651	20,131	100
**2001–2010**	Total	2,661	25,786	100
**After 2010**	Total	902	6,999	100

© 2024 van Ettekoven CN et al. *JAMA Network Open.*

eTable 6. Proportions of the causative pathogens, stratified by age group.

Age group	Pathogen	Deaths	Episodes	Proportion per age group [%]
**Adults**	S. pneumoniae	711	3,853	40.5
**Adults**	N. meningitidis	161	2,224	23.4
**Adults**	H. influenzae	15	186	2.0
**Adults**	L. monocytogenes	37	394	4.1
**Adults**	E. coli	24	113	1.2
**Adults**	S. agalactiae	11	46	0.5
**Adults**	S. aureus	42	204	2.1
**Adults**	Enterobacter spp.	22	26	0.3
**Adults**	Pseudomonas spp.	7	18	0.2
**Adults**	K. pneumoniae	15	44	0.5
**Adults**	Other bacteria	219	1,168	12.3
**Adults**	Unidentified	152	1,245	13.1
**Children**	S. pneumoniae	1,086	6,650	24.7
**Children**	N. meningitidis	277	7,315	27.2
**Children**	H. influenzae	863	7,188	26.7
**Children**	L. monocytogenes	0	23	0.1
**Children**	E. coli	27	169	0.6
**Children**	S. agalactiae	21	275	1.0
**Children**	S. aureus	6	44	0.2
**Children**	Enterobacter spp.	58	74	0.3
**Children**	Pseudomonas spp.	18	24	0.1
**Children**	K. pneumoniae	3	5	0
**Children**	Other bacteria	311	1,518	5.6
**Children**	Unidentified	477	3,609	13.4
**Neonates**	S. pneumoniae	80	403	5.5
**Neonates**	N. meningitidis	6	197	2.7
**Neonates**	H. influenzae	12	115	1.6
**Neonates**	L. monocytogenes	14	290	4.0
**Neonates**	E. coli	159	1,621	22.1
**Neonates**	S. agalactiae	137	2,367	32.3
**Neonates**	S. aureus	26	137	1.9
**Neonates**	Enterobacter spp.	48	111	1.5
**Neonates**	Pseudomonas spp.	32	106	1.4
**Neonates**	K. pneumoniae	20	135	1.8
**Neonates**	Other bacteria	184	1,414	19.3
**Neonates**	Unidentified	63	423	5.8
**Adults**	Total	1,416	9,521	100
**Children**	Total	3,147	26,894	100
**Neonates**	Total	781	7,319	100

© 2024 van Ettekoven CN et al. *JAMA Network Open.*

eTable 7. Results of the meta-regression model, stratified by Human Development Index (HDI, High-income countries-income countries, Low-income countries-income countries) and age group.

	Age group	Study periods, k	Effect estimate (95% CI) [%]	τ^2	I^2 [%]	R^2 [%]	QEp	QMp
**All countries**	Combined	419	-1.4 (-2 to -0.9)	0.817	97	6	< 0.001	< 0.001
**High**	Neonates	17	-3.3 (-4.3 to -2.2)	0.036	28	91	0.098	<0.001
**High**	Children	58	-0.8 (-2.3 to 0.7)	0.417	93	0	< 0.001	0.300
**High**	Adults	16	2.5 (-2.3 to 7.3)	0.501	93	0	< 0.001	0.312
**Low**	Neonates	62	-5.4 (-6.6 to -4.1)	0.568	87	61	< 0.001	< 0.001
**Low**	Children	73	-2.7 (-4 to -1.4)	0.688	92	19	< 0.001	< 0.001
**Low**	Adults	58	-1.8 (-3.1 to -0.6)	0.252	89	14	< 0.001	0.004
Legend: Effect estimate, change per year; τ2, estimated amount of (residual) heterogeneity; k, number of outcomes included in the model fitting; QEp, p-value for the test of (residual) heterogeneity; QMp, p-value for the omnibus test of coefficients								

© 2024 van Ettekoven CN et al. *JAMA Network Open.*

eTable 8. Studies reporting on *Streptococcus pneumoniae* specifically.

First author [reference]	Pub. year	Country	HDI [3]	Inclusion period	Age group	Patients, n	Deaths, n
**Feibush [373]**	1952	United States	high	1943-01-01 – 1951-06-30	Adults	22	15
**Pengelly [374]**	1955	United Kingdom	high	1946-01-01 – 1953-05-31	Not specified	78	38
**Wehrle [375]**	1967	United States	high	1963-07-01 – 1966-06-30	Not specified	173	49
**Pirame [376]**	1968	Madagascar	low	NA - NA	Not specified	126	76
**Haddock [377]**	1971	Ghana	low	1968-01-01 – 1970-09-01	Children and adults	22	12
**Levin [378]**	1972	United States	high	1954-01-01 – 1968-01-01	Not specified	155	26
**Baird [379]**	1976	Nigeria	low	1971-02-01 – 1976-06-30	Not specified	185	94
**Laxer [380]**	1977	Canada	high	NA - NA	Children	83	9
**Marr [381]**	1977	United States	high	1972-01-01 – 1976-12-31	Not specified	293	187
**Rees [382]**	1977	United Kingdom	high	1960-01-01 – 1969-12-31	Not specified	61	26
**Bademosi [383]**	1979	Nigeria	low	NA – NA	Children and adults	52	23
**Cadoz [384]**	1979	Senegal	low	1973-01-01 – 1977-12-31	Not specified	402	234
**Jacobs [385]**	1979	United States	high	1967-01-01 – 1976-12-31	Children	70	9
**Ostroy [386]**	1979	United States	high	1977-01-01 – 1977-12-31	Not specified	24	10
**Berkowitz [387]**	1981	South Africa	low	1977-09-01 – 1978-08-31	Neonates and children	11	6
**Gallais [388]**	1983	Ivory Coast	low	1975-12-01 – 1979-09-01	Not specified	119	55
**Nottidge [389]**	1983	Nigeria	low	1969-01-01 – 1980-12-31	Children	36	7
**Pedersen [390]**	1983	Denmark	high	1969-01-01 – 1978-12-31	Neonates and children	146	19
**Coulehan [391]**	1984	United States	high	1973-07-01 – 1980-06-01	Children	77	12

[3] Human Development Index

© 2024 van Ettekoven CN et al. *JAMA Network Open.*

First author [reference]	Pub. year	Country	HDI [3]	Inclusion period	Age group	Patients, n	Deaths, n
**Burman** [392]	1985	Sweden	high	1964-01-01 – 1980-12-31	Not specified	125	41
**Chan-Lui** [393]	1985	Hong Kong	high	1963-01-01 – 1982-12-31	Neonates and children	38	11
**Gransden** [394]	1985	United Kingdom	high	NA - NA	Not specified	26	10
**Lecour** [395]	1985	Portugal	high	1981-01-01 – 1984-12-31	Children and adults	46	6
**Gray** [396]	1986	United States	high	1979-01-01 – 1984-12-31	Children	44	3
**Bruyn** [397]	1989	Netherlands	high	1975-01-01 – 1987-01-01	Adults	38	13
**Kennedy** [398]	1991	United States	high	1984-01-01 – 1990-09-30	Children	97	9
**Viladrich** [399]	1991	Spain	high	1988-03-01 – 1989-01-01	Adults	11	1
**Barsic** [400]	1992	Croatia	high	1985-01-01 – 1989-12-31	Children and adults	70	23
**Davidson** [401]	1994	United States	high	1986-01-01 – 1990-12-31	Not specified	59	11
**Kirkpatrick** [402]	1994	United Kingdom	high	1982-01-01 – 1992-12-31	Not specified	77	10
**Kragsbjerg** [403]	1994	Sweden	high	1981-01-01 – 1992-12-31	Adults	31	5
**Voss** [404]	1994	New Zealand	high	1984-01-01 – 1992-12-31	Neonates and children	70	3
**Davis** [405]	1995	Australia	high	1981-01-01 – 1992-12-31	Neonates and children	135	11
**Kanra** [406]	1995	Turkey	high	1990-01-01 – 1993-12-31	Children	56	3
**Kornelisse** [407]	1995	Netherlands	high	1970-01-01 – 1994-04-30	Neonates and children	83	14
**Urwin** [408]	1996	United Kingdom	high	1990-01-01 – 1993-12-31	Not specified	210	44
**Arditi** [409]	1998	United States	high	1993-09-01 – 1996-08-31	Children	181	14
**Venetz** [410]	1998	Switzerland	high	1985-01-01 – 1994-12-31	Children	165	14
**Gomez-Barreto** [411]	1999	Mexico	high	1994-01-01 – 1998-12-31	Children	38	5

© 2024 van Ettekoven CN et al. *JAMA Network Open.*

First author [reference]	Pub. year	Country	HDI [3]	Inclusion period	Age group	Patients, n	Deaths, n
**Muhe** [412]	1999	Ethiopia	low	1993-01-01 – 1995-12-31	Children	46	19
**Schneider** [413]	1999	Multinational	high	1995-01-01 – 1998-12-31	Children and adults	30	6
**Stanek** [414]	1999	United States	high	1978-01-01 – 1997-12-31	Not specified	55	16
**Fiore** [415]	2000	United States	high	1994-11-01 – 1996-04-01	Not specified	109	15
**Goetghebuer** [416]	2000	Gambia	low	1990-01-01 – 1995-12-31	Neonates and children	134	64
**Mencia Bartolome** [417]	2000	Spain	high	1990-01-01 – 1999-12-31	Children	28	4
**Soult Rubio** [418]	2001	Spain	high	1977-01-01 – 2000-12-31	Children	53	2
**Chomarat** [419]	2002	France	high	1999-01-01 – 1999-12-31	Children and adults	35	7
**Ma** [420]	2002	Taiwan	high	1990-01-01 – 2000-04-30	Neonates and children	28	9
**Ulloa-Gutierrez** [421]	2003	Costa Rica	high	1995-01-01 – 2001-12-31	Neonates and children	56	9
**Ispahani** [422]	2004	United Kingdom	high	1980-01-01 – 1999-12-31	Neonates and children	86	17
**Buckingham** [423]	2005	United States	high	1991-01-01 – 2001-12-31	Children	114	10
**Lovera** [424]	2005	Paraguay	high	1990-01-01 – 2003-12-31	Children	72	24
**McIntyre** [425]	2005	Australia	high	1994-01-01 – 1999-12-31	Neonates and children	120	15
**Ostergaard** [426]	2005	Denmark	high	1999-01-01 – 2000-12-31	Not specified	187	39
**Yaro** [427]	2006	Burkina Faso	low	2002-01-01 – 2005-12-31	Not specified	249	115
**Carrol** [428]	2007	Malawi	low	2004-04-01 – 2005-04-01	Children	82	25
**Holliman** [429]	2007	Ghana	low	2002-01-01 – 2005-04-01	Not specified	111	55
**Kallel** [430]	2007	Tunisia	high	1993-01-01 – 2001-12-31	Children and adults	31	8

© 2024 van Ettekoven CN et al. *JAMA Network Open.*

First author [reference]	Pub. year	Country	HDI [3]	Inclusion period	Age group	Patients, n	Deaths, n
**Pagliano** [431]	2007	Italy	high	1997-01-01 – 2005-12-31	Children	64	2
**Thabet** [432]	2007	Tunisia	high	1995-01-01 – 2002-12-31	Children	71	10
**Chong** [433]	2008	Singapore	high	1997-01-01 – 2004-12-31	Neonates and children	23	7
**Ishiwada** [434]	2008	Japan	high	2003-01-01 – 2005-12-31	Neonates and children	16	0
**Manga** [435]	2008	Senegal	low	1995-01-01 – 2004-12-31	Adults	73	51
**Rajasingham** [436]	2008	United States	high	1993-01-01 – 2004-12-31	Neonates and children	86	6
**Tsai** [437]	2008	Taiwan	high	1984-01-01 – 2002-12-31	Neonates and children	49	12
**Falade** [438]	2009	Nigeria	low	2005-02-01 – 2007-06-30	Children	9	8
**Gil Prieto** [439]	2009	Spain	high	1998-01-01 – 2006-12-31	Children	770	61
**Kisakye** [440]	2009	Uganda	low	2001-07-12 – 2006-08-31	Neonates and children	275	56
**Trotman** [441]	2009	Jamaica	high	1995-01-01 – 1999-12-31	Children	25	3
**Brouwer** [442]	2010	Netherlands	high	2006-01-01 – 2009-12-31	Adults	357	70
**Memish** [443]	2010	Saudi Arabia	high	1999-01-01 – 2003-12-31	Neonates and children	19	3
**Ochoa** [444]	2010	Peru	high	2006-05-01 – 2008-04-01	Neonates and children	34	11
**Gouveia** [445]	2011	Brazil	high	1995-01-01 – 2005-12-31	Not specified	545	202
**Lucey** [446]	2011	Ireland	high	1998-01-01 – 2007-12-31	Neonates and children	44	8
**Novaes** [447]	2011	Brazil	high	2004-01-01 – 2006-12-31	Not specified	4,032	1,276
**Nyasulu** [448]	2011	South Africa	low	2003-01-01 – 2005-12-31	Neonates and children	581	205

© 2024 van Ettekoven CN et al. *JAMA Network Open.*

First author [reference]	Pub. year	Country	HDI [3]	Inclusion period	Age group	Patients, n	Deaths, n
**Choi** [449]	2012	Korea	low	1991-01-01 – 2010-12-31	Children and adults	114	25
**Stockmann** [450]	2013	United States	high	1997-01-01 – 2010-12-31	Neonates and children	66	9
**Thomas** [451]	2013	India	low	1993-01-01 – 2008-12-31	Adults	149	55
**Tsai** [452]	2013	Taiwan	high	2002-01-01 – 2008-12-31	Not specified	330	21
**Berberian** [453]	2014	Argentina	high	1999-01-01 – 2010-12-31	Children	111	11
**Browall** [454]	2014	Sweden	high	1998-01-01 – 2008-12-31	Adults	135	24
**Erdem** [455]	2014	Turkey	high	1998-01-01 – 2012-12-31	Adults	306	42
**Levy** [456]	2014	France	high	2001-01-01 – 2012-12-31	Neonates and children	1,406	148
**Paulke** [457]	2014	Austria	high	2001-01-01 – 2012-12-31	Neonates and children	85	8
**von Mollendorf** [458]	2014	South Africa	low	2009-01-01 – 2010-12-31	Not specified	990	416
**Casez** [459]	2015	France	high	2005-01-01 – 2010-12-31	Not specified	411	78
**Grando** [460]	2015	Brazil	high	2007-01-01 – 2012-12-31	Neonates and children	1,311	428
**Jung** [461]	2015	Korea	high	1997-01-01 – 2013-03-01	Adults	56	8
**Navarro** [462]	2015	Multinational	high	2010-01-01 – 2010-12-31	Not specified	329	62
**Buchholz** [463]	2016	Germany	high	1984-01-01 – 2015-12-31	Adults	142	24
**Lundbo** [464]	2016	Denmark	high	1982-07-01 – 2008-04-30	Neonates and children	372	15
**Rojas** [465]	2016	Colombia	high	2008-01-01 – 2014-01-15	Children	18	6
**Saha** [466]	2016	Bangladesh	low	2007-01-01 – 2013-12-31	Neonates and children	540	30
**Webber** [467]	2016	United States	high	2004-01-01 – 2014-12-31	Neonates and children	14	9

© 2024 van Ettekoven CN et al. *JAMA Network Open.*

First author [reference]	Pub. year	Country	HDI [3]	Inclusion period	Age group	Patients, n	Deaths, n
**Lim** [468]	2017	Korea	low	1998-01-01 – 2013-01-01	Adults	103	18
**Moisi** [469]	2017	Togo	low	2010-05-01 – 2013-04-30	Not specified	78	27
**Pirez** [470]	2017	Uruguay	high	2005-01-01 – 2015-12-31	Children	52	16
**Coldiron** [471]	2018	Central African Rep.	low	2016-10-01 – 2017-04-09	Not specified	60	6
**Ben Salah** [472]	2019	Tunisia	high	2014-06-01 – 2015-05-31	Children	21	7
**Irfan** [473]	2019	Pakistan	low	2011-01-01 – 2014-03-01	Not specified	31	7
**Jayaraman** [474]	2019	India	low	2007-01-01 – 2017-07-01	Adults	73	7
**Mihret** [475]	2019	Ethiopia	low	2012-02-01 – 2013-06-01	Children and adults	16	3
**Oligbu** [476]	2019	United Kingdom	high	2000-07-01 – 2016-06-30	Not specified	3,612	631
**Tenforde** [477]	2019	Botswana	low	2004-01-01 – 2015-12-31	Adults	238	105
**Blanco** [478]	2020	Brazil	high	2008-01-01 – 2018-12-01	Children	21	2
**Hernstadt** [479]	2020	Australia	high	2011-01-01 – 2017-05-07	Neonates and children	24	0
**Szymanski** [480]	2020	Poland	high	1998-01-01 – 2018-01-01	Adults	56	12
**Hanada** [481]	2021	Japan	high	2010-04-01 – 2017-03-01	Children and adults	127	20
**Iwata** [482]	2021	Japan	high	2002-01-01 – 2016-12-31	Neonates and children	202	11
**Muller** [483]	2021	South Africa	low	2018-01-01 – 2019-08-01	Not specified	48	17
**Polkowska** [484]	2021	Finland	high	2004-07-01 – 2017-06-30	Not specified	451	64
**Stevens** [485]	2022	United States	high	2010-01-01 – 2018-04-30	Children	21	1

© 2024 van Ettekoven CN et al. *JAMA Network Open.*

eTable 9. Studies reporting on *Neisseria meningitidis* specifically.

First author [reference]	Pub. year	Country	HDI [4]	Inclusion period	Age group	Patients, n	Deaths, n
**Petuelli** [486]	1967	Austria	high	1960-01-01 – 1966-04-01	Neonates and children	46	0
**Wehrle** [375]	1967	United States	high	1963-07-01 – 1966-06-30	Not specified	463	39
**Vassiliadis** [487]	1969	Greece	high	1959-01-01 – 1967-12-31	Not specified	2,027	113
**Gendron** [488]	1972	Burkina Faso	low	1969-01-01 – 1971-12-31	Not specified	865	87
**Greenwood** [489]	1974	Nigeria	low	1971-02-01 – 1972-05-30	Not specified	101	7
**Evans-Jones** [490]	1977	Nigeria	low	1975-01-01 – 1975-04-01	Not specified	100	12
**Andersen** [491]	1978	Norway	high	1966-01-01 – 1976-12-31	Not specified	108	8
**Ellsworth** [492]	1979	Canada	high	1971-01-01 – 1975-12-31	Children	44	2
**Feldman** [493]	1979	Costa Rica	high	1970-01-01 – 1973-12-31	Not specified	312	45
**Olcen** [494]	1979	Sweden	high	1965-01-01 – 1977-12-31	Children and adults	58	3
**Ambrosch** [495]	1980	Austria	high	1960-01-01 – 1979-07-31	Not specified	1,868	158
**Conner** [496]	1980	Canada	high	NA - NA	Children	19	1
**Binkin** [497]	1982	Mali	low	1981-01-01 – 1981-04-30	Not specified	831	88
**Hansman** [498]	1983	Australia	high	1971-01-01 – 1980-12-31	Not specified	69	5

[4] Human Development Index

© 2024 van Ettekoven CN et al. *JAMA Network Open.*

First author [reference]	Pub. year	Country	HDI [4]	Inclusion period	Age group	Patients, n	Deaths, n
**De Wals** [499]	1984	Belgium	high	1975-01-01 – 1979-12-31	Neonates and children	198	7
**Fallon** [500]	1984	United Kingdom	high	1972-01-01 – 1982-12-31	Not specified	597	45
**Mohammed** [501]	1984	Nigeria	low	1978-01-01 – 1981-12-31	Not specified	7,471	556
**PHLS** [502]	1986	United Kingdom	high	1985-01-01 – 1985-12-31	Not specified	549	94
**Spanjaard** [503]	1987	Netherlands	high	1959-01-01 – 1983-12-31	Not specified	1,123	47
**Valmari** [504]	1987	Finland	high	1976-01-01 – 1980-12-31	Not specified	427	23
**Annapurna** [505]	1989	India	low	1985-09-01 – 1986-03-31	Children and adults	27	3
**Halstensen** [506]	1989	Norway	high	1980-01-01 – 1987-12-31	Children and adults	58	3
**Salih** [507]	1990	Sudan	low	1988-01-01 – 1988-12-31	Children	112	7
**Tesoro** [508]	1991	United States	high	1979-12-19 – 1987-12-19	Children	46	2
**Fakhir** [509]	1992	India	low	1983-01-01 – 1990-04-30	Children	247	40
**Fekade** [510]	1992	Ethiopia	low	1988-01-01 – 1988-12-31	Adults	204	43
**Palmer** [511]	1992	United Kingdom	high	1988-01-01 – 1988-12-31	Not specified	85	9
**Kristos** [512]	1993	Ethiopia	low	1987-12-01 – 1989-01-31	Neonates and children	124	3
**Patel** [513]	1993	Australia	high	1987-09-01 – 1991-05-01	Not specified	76	4

© 2024 van Ettekoven CN et al. *JAMA Network Open.*

First author [reference]	Pub. year	Country	HDI [4]	Inclusion period	Age group	Patients, n	Deaths, n
**World Health Organization [514]**	1993	Burundi	low	1992-01-01 – 1992-12-31	Not specified	2,531	279
**Scholten [515]**	1994	Netherlands	high	1989-04-01 – 1990-04-30	Not specified	448	30
**Alhan [516]**	1995	Turkey	high	1989-01-01 – 1993-12-31	Children	43	6
**Flaegstad [517]**	1995	Norway	high	1977-01-01 – 1992-12-31	Children	54	2
**Riordan [518]**	1995	United Kingdom	high	1977-01-01 – 1993-12-31	Children	342	30
**Munro [519]**	1996	Australia	high	1990-01-01 – 1994-12-31	Not specified	43	2
**Semba [520]**	1996	Rwanda	low	1992-09-01 – 1992-11-01	Children	41	8
**Aplogan [521]**	1997	Togo	low	1996-12-02 – 1997-05-18	Not specified	2,992	440
**Juncal [522]**	1997	Spain	high	1990-01-01 – 1997-03-31	Not specified	61	4
**Luaces Cubells [523]**	1997	Spain	high	1988-01-01 – 1992-12-31	Children	213	7
**Ndihokubwayo [524]**	1997	Burundi	low	1996-09-01 – 1996-10-31	Not specified	173	38
**Heyman [525]**	1998	Dem. Rep. Congo	low	1994-07-01 – 1994-08-01	Not specified	45	6
**AMSP [526]**	1999	Australia	high	1998-01-01 – 1998-12-31	Not specified	40	4
**Pancharoen [527]**	2000	Thailand	high	1994-01-01 – 1999-12-31	Not specified	18	0
**Seydi [528]**	2002	Senegal	low	1999-01-01 – 1999-12-31	Children and adults	70	3

© 2024 van Ettekoven CN et al. *JAMA Network Open.*

First author [reference]	Pub. year	Country	HDI [4]	Inclusion period	Age group	Patients, n	Deaths, n
**Karima** [529]	2003	Saudi Arabia	high	2000-02-01 – 2000-04-01	Adults	105	36
**Mengistu** [530]	2003	Ethiopia	low	2001-01-01 – 2002-12-31	Not specified	1,619	154
**Dominguez** [531]	2004	Spain	high	1990-01-01 – 1997-12-31	Not specified	1,247	53
**Tapsall** [532]	2004	Australia	high	2003-01-01 – 2003-12-31	Not specified	39	2
**Nathan** [533]	2005	Niger	low	2003-03-24 – 2003-04-27	Children and adults	308	11
**Sanou** [534]	2006	Burkina Faso	low	2002-01-01 – 2003-12-31	Not specified	148	23
**AMSP** [535]	2007	Australia	high	2006-01-01 – 2006-12-31	Not specified	44	3
**Dash** [536]	2007	United Arab Emirates	high	2000-01-01 – 2005-06-30	Adults	12	0
**Gryniewicz** [537]	2007	Poland	high	1994-01-01 – 2006-12-31	Not specified	1,676	75
**Howitz** [538]	2009	Denmark	high	1980-01-01 – 2007-12-31	Not specified	3,663	234
**Jhamb** [539]	2009	India	low	2005-04-01 – 2006-12-01	Children	80	12
**Mutonga** [540]	2009	Kenya	low	2005-01-01 – 2006-12-31	Not specified	70	11
**Weiss** [541]	2009	United States	high	2005-11-01 – 2006-11-01	Children and adults	15	2
**Cohen** [542]	2010	South Africa	low	2003-01-01 – 2007-12-31	Not specified	226	16
**Tolaj** [543]	2010	Kosovo	low	NA – NA	Not specified	130	9
**Gil Prieto** [544]	2011	Spain	high	1997-01-01 – 2008-12-31	Not specified	6,131	235

© 2024 van Ettekoven CN et al. *JAMA Network Open.*

First author [reference]	Pub. year	Country	HDI [4]	Inclusion period	Age group	Patients, n	Deaths, n
**Steindl** [545]	2011	Austria	high	2010-01-01 – 2010-12-31	Not specified	41	2
**Yameogo** [546]	2011	Burkina Faso	low	2007-01-01 – 2007-12-31	Not specified	861	30
**Levy** [547]	2012	France	high	2001-01-01 – 2009-12-31	Neonates and children	1,661	108
**Xu** [548]	2012	China	high	2000-01-01 – 2010-12-31	Not specified	200	9
**Dass Hazarika** [549]	2013	India	low	2008-01-01 – 2009-06-01	Children	88	3
**Stein-Zamir** [550]	2014	Israel	high	1999-01-01 – 2010-12-31	Neonates and children	123	6
**Osuorah** [551]	2015	Gambia	low	2012-01-01 – 2012-06-30	Neonates and children	89	7
**Sadarangani** [552]	2015	Canada	high	NA - NA	Not specified	504	26
**Stoof** [553]	2015	Netherlands	high	1999-06-01 – 2011-06-01	Not specified	617	36
**Bassey** [554]	2016	Nigeria	low	NA – NA	Not specified	54,766	2,449
**Strelow** [555]	2016	Brazil	high	2006-01-01 – 2011-01-01	Not specified	316	17
**Patel** [556]	2017	Liberia	low	2017-04-21 – 2017-04-30	Not specified	14	11
**Sall** [557]	2017	Sweden	high	1995-01-01 – 2012-12-31	Not specified	58	4
**Menichetti** [558]	2018	Italy	high	2015-01-01 – 2016-12-01	Neonates and children	33	5
**Mihret** [475]	2019	Ethiopia	low	2012-02-01 – 2013-06-01	Not specified	27	2

© 2024 van Ettekoven CN et al. *JAMA Network Open.*

eTable 10. Studies reporting on *Haemophilus influenzae* specifically.

First author [reference]	Pub. year	Country	HDI [5]	Inclusion period	Age group	Patients, n	Deaths, n
**Wehrle** [375]	1967	United States	high	1961-01-01 – 1966-10-31	Not specified	578	41
**Schulkind** [559]	1971	United States	high	1965-01-01 – 1969-08-01	Not specified	37	4
**Barrett** [560]	1972	United States	high	1959-01-01 – 1970-12-31	Children	253	19
**Parke** [561]	1972	United States	high	1966-01-01 – 1970-12-31	Not specified	86	4
**Herrera Labarca** [562]	1977	Chile	high	1970-01-01 – 1976-03-31	Children	100	30
**Koskiniemi** [563]	1978	Finland	high	1960-01-01 – 1974-12-31	Children	131	5
**Ostroy** [386]	1979	United States	high	1977-01-01 – 1977-12-31	Not specified	88	8
**Koo** [564]	1982	Australia	high	1979-12-01 – 1981-11-01	Children	23	0
**Broughton** [565]	1984	United Kingdom	high	1975-01-01 – 1981-12-31	Not specified	41	2
**Coulehan** [391]	1984	United States	high	1973-07-01 – 1980-06-01	Children	206	9
**Istre** [566]	1984	United States	high	1977-01-01 – 1981-12-31	Not specified	286	15
**Nottidge** [567]	1985	Nigeria	low	1977-01-01 – 1980-12-31	Neonates and children	127	33
**Campos** [568]	1986	Spain	high	1981-01-01 – 1984-12-31	Children	35	2
**Dyas** [569]	1986	United Kingdom	high	1973-01-01 – 1984-12-31	Children	42	2

[5] Human Development Index

First author [reference]	Pub. year	Country	HDI [5]	Inclusion period	Age group	Patients, n	Deaths, n
**Nesheim** [570]	1986	United States	high	1974-01-01 – 1984-12-31	Neonates and children	189	2
**Taft** [571]	1986	United States	high	1980-01-01 – 1984-12-31	Children	170	3
**Ward** [572]	1986	United States	high	1980-01-01 – 1982-12-31	Not specified	130	7
**Trollfors** [573]	1987	Sweden	high	NA - NA	Not specified	500	16
**Valmari** [504]	1987	Finland	high	1976-01-01 – 1980-12-31	Not specified	492	20
**Cordtz** [574]	1988	Denmark	high	1981-04-01 – 1987-06-30	Not specified	327	8
**Kaplan** [575]	1988	United States	high	1981-01-01 – 1984-12-31	Children	134	5
**Tudor-Williams** [576]	1989	United Kingdom	high	1985-01-01 – 1988-06-01	Children	142	8
**Ferreccio** [577]	1990	Chile	high	1985-01-01 – 1987-12-31	Neonates and children	242	39
**Peltola** [578]	1990	Multinational	high	1974-01-01 – 1986-12-31	Not specified	1,611	39
**Bijlmer** [579]	1992	Gambia	low	1985-08-01 – 1987-08-01	Neonates and children	77	29
**Murphy** [580]	1992	United States	high	1983-01-01 – 1984-12-31	Neonates and children	457	18
**Gervaix** [581]	1993	Switzerland	high	1976-01-01 – 1989-12-31	Children	76	0
**McIntyre** [582]	1993	Australia	high	1985-01-01 – 1990-12-31	Children	229	7
**Reinert** [583]	1993	France	high	1980-01-01 – 1989-12-31	Neonates and children	177	7
**Spencker** [584]	1993	Germany	high	1971-01-01 – 1989-12-31	Children	94	6

© 2024 van Ettekoven CN et al. *JAMA Network Open.*

First author [reference]	Pub. year	Country	HDI [5]	Inclusion period	Age group	Patients, n	Deaths, n
**Likitnukul** [585]	1994	Thailand	high	1980-01-01 – 1992-12-31	Neonates and children	44	3
**Rauter** [586]	1994	Austria	high	1983-01-01 – 1992-12-31	Children	40	2
**Dabernat** [587]	1996	France	high	1991-10-01 – 1993-06-01	Children	104	3
**Muhe** [412]	1999	Ethiopia	low	1993-01-01 – 1995-12-31	Children	74	28
**Goetghebuer** [416]	2000	Gambia	low	1990-01-01 – 1995-12-31	Neonates and children	123	33
**Limcangco** [588]	2000	Philippines	low	1994-01-01 – 1996-12-31	Neonates and children	118	13
**Steinhoff** [589]	2002	India	low	1993-01-01 – 1997-12-31	Not specified	80	12
**Kim** [590]	2004	Korea	high	1999-09-01 – 2001-12-31	Neonates and children	14	1
**Yaro** [591]	2006	Burkina Faso	low	2002-01-01 – 2005-12-31	Not specified	112	28
**Miranzi** [592]	2007	Brazil	high	1983-01-01 – 2002-12-31	Neonates and children	20,153	3,633
**Ribeiro** [593]	2007	Brazil	high	1996-08-09 – 2004-08-08	Not specified	522	83
**Thoon** [594]	2007	Singapore	high	1994-01-01 – 2003-12-31	Children	31	2
**Lee** [595]	2008	Uganda	low	2002-01-01 – 2005-12-31	Children	41	14
**Rahman** [596]	2008	Bangladesh	low	1999-01-01 – 2003-12-31	Children	46	9
**Falade** [438]	2009	Nigeria	low	2005-02-01 – 2007-06-30	Children	11	5

© 2024 van Ettekoven CN et al. *JAMA Network Open.*

First author [reference]	Pub. year	Country	HDI [5]	Inclusion period	Age group	Patients, n	Deaths, n
**Nyambat** [597]	2011	Vietnam	low	2004-01-01 – 2005-12-31	Neonates and children	25	1
**Rubach** [598]	2011	United States	high	1998-01-01 – 2008-12-31	Adults	15	2
**Ubukata** [599]	2013	Japan	high	2000-01-01 – 2011-12-31	Neonates and children	587	12
**Bamberger** [600]	2014	Israel	high	2003-01-01 – 2012-12-31	Neonates and children	76	0

© 2024 van Ettekoven CN et al. *JAMA Network Open.*

eTable 11. Studies reporting on *Listeria monocytogenes* specifically.

First author [reference]	Pub. year	Country	HDI [6]	Inclusion period	Age group	Patients, n	Deaths, n
**Lavetter** [601]	1971	United States	high	1960-01-01 – 1969-12-31	Not specified	25	8
**Bowmer** [602]	1973	Canada	high	1951-01-01 – 1972-01-01	Not specified	47	14
**Iwarson** [603]	1979	Sweden	high	1958-01-01 – 1977-12-31	Adults	60	16
**Ostroy** [386]	1979	United States	high	1977-01-01 – 1977-12-31	Not specified	6	0
**Cherubin** [604]	1981	United States	high	1972-01-01 – 1979-12-31	Not specified	53	33
**Nau** [605]	1990	Germany	high	1980-01-01 – 1988-12-31	Adults	12	1
**Bula** [606]	1995	Switzerland	high	1983-01-01 – 1987-12-31	Adults	45	9
**Mylonakis** [607]	1998	United States	high	1964-07-01 – 1997-07-01	Adults	43	10
**Amaya-Villar** [608]	2010	Spain	high	NA – NA	Adults	43	12
**Roed** [609]	2012	Denmark	high	1977-01-01 – 2006-12-31	Adults	183	69
**Pelegrin** [610]	2014	Spain	high	1977-01-01 – 2009-12-31	Adults	59	14
**Arslan** [611]	2015	Multinational	high	1990-01-01 – 2014-12-31	Adults	100	25
**Thonnings** [612]	2016	Denmark	high	1997-01-01 – 2012-12-31	Not specified	57	14
**Koopmans** [613]	2017	Netherlands	high	1994-01-01 – 2014-12-31	Not specified	220	69
**Lim** [468]	2017	Korea	low	1998-01-01 – 2013-01-01	Adults	32	10

[6] Human Development Index

© 2024 van Ettekoven CN et al. *JAMA Network Open.*

eTable 12. Studies reporting on *Escherichia coli* specifically.

First author [reference]	Pub. year	Country	HDI [7]	Inclusion period	Age group	Patients, n	Deaths, n
**McCracken [614]**	1974	United States	high	1968-01-01 – 1971-09-01	Neonates and children	57	15
**Heckmatt [615]**	1976	United Kingdom	high	1960-01-01 – 1974-12-31	Neonates	21	6
**Cherubin [604]**	1981	United States	high	1972-01-01 – 1979-12-31	Not specified	64	41
**Houdouin [616]**	2008	France	high	1988-01-01 – 2004-12-31	Neonates	99	14
**Basmaci [617]**	2015	France	high	2001-01-01 – 2013-12-31	Neonates and children	325	30
**Tauzin [618]**	2019	France	high	2001-01-01 – 2016-12-31	Neonates and children	367	38
**Yun [619]**	2019	Korea	high	2011-01-01 – 2017-12-31	Neonates	10	1

[7] Human Development Index

© 2024 van Ettekoven CN et al. *JAMA Network Open.*

eTable 13. Studies reporting on *Streptococcus agalactiae* specifically.

First author [reference]	Pub. year	Country	HDI [8]	Inclusion period	Age group	Patients, n	Deaths, n
**Haslam [620]**	1977	United States	high	1968-01-01 – 1974-12-31	Neonates	18	3
**Ostroy [386]**	1979	United States	high	1977-01-01 – 1977-12-31	Not specified	10	2
**Schrag [621]**	2000	United States	high	1993-01-01 – 1998-12-31	Neonates	95	4
**Madhi [622]**	2003	South Africa	low	1997-01-01 – 1999-12-31	Neonates and children	46	11
**Georget-Bouquinet [623]**	2008	France	high	2001-01-01 – 2005-12-31	Neonates and children	276	39
**Apostol [624]**	2009	United States	high	2000-01-01 – 2006-12-31	Neonates	313	20
**Libster [625]**	2012	United States	high	1998-01-01 – 2006-12-31	Neonates	90	5
**Joubrel [626]**	2015	France	high	2007-01-01 – 2012-12-31	Neonates	155	22
**Bartlett [627]**	2017	Australia	high	2000-01-01 – 2014-12-31	Neonates and children	66	7
**Lo [628]**	2019	Taiwan	high	1998-10-01 – 2014-12-01	Neonates and children	31	3
**von Kassel [629]**	2019	Netherlands	high	1998-10-01 – 2017-12-31	Adults	33	7
**Yun [619]**	2019	Korea	high	2011-01-01 – 2017-12-31	Neonates	20	0
**Geteneh [630]**	2020	Ethiopia	low	2018-06-01 – 2018-10-01	Neonates and children	46	5
**Nakwa [631]**	2020	South Africa	low	2012-11-01 – 2014-02-01	Neonates	44	5

[8] Human Development Index

© 2024 van Ettekoven CN et al. *JAMA Network Open.*

Pneumococcal meningitis

eTable 14. Study characteristics and unadjusted case fatality ratios in pneumococcal meningitis per subgroup.

	Study periods, k	Episodes, n	Deaths, n	Case fatality ratio (95% CI) [%]
**All studies**	319	46,597	11,536	24.8 (24.3–25.2)
**Neonates**	18	101	28	27.7 (17.5–38.0)
**Children**	82	5,564	991	17.8 (16.7–18.9)
**Adults**	40	3,620	978	27.0 (25.3–28.7)
**High-income countries**	220	33,845	7,200	21.3 (20.8–21.8)
**Low-income countries**	99	12,752	4,336	34.0 (33.0–35.0)

eTable 15. Results of the meta-analyses of case fatality ratios in pneumococcal meningitis per subgroup.

Human Development Index	Age group	Study periods, k	Case fatality ratio (95% CI) [%]	I^2 [%]
**High and low-income countries**	Combined	362	24.3 (22.4–26.2)	91
**High-income countries**	Neonates	22	31.4 (18.8–47.6)	53
**High-income countries**	Children	71	14.4 (11.9–17.3)	88
**High-income countries**	Adults	42	26.9 (23–31.1)	80
**Low-income countries**	Neonates	7	43.2 (28–59.9)	18
**Low-income countries**	Children	32	33.4 (28.8–38.3)	86
**Low-income countries**	Adults	11	34.5 (23.5–47.4)	90

© 2024 van Ettekoven CN et al. *JAMA Network Open.*

Meningococcal meningitis

eTable 16. Study characteristics and unadjusted case fatality ratios in meningococcal meningitis per subgroup.

	Study periods, k	Episodes, n	Deaths, n	Case fatality ratio (95% CI) [%]
**All studies**	250	123,830	7,742	6.3 (6.1–6.4)
**Children**	61	4,357	263	6.0 (5.3–6.8)
**Adults**	22	1,351	186	13.8 (11.8–15.7)
**High-income countries**	169	41,157	2,642	6.4 (6.2–6.7)
**Low-income countries**	81	82,673	5,100	6.2 (6.0–6.3)

eTable 17. Results of the meta-analyses of case fatality ratios in meningococcal meningitis per subgroup.

Human Development Index	Age group	Study periods, k	Case fatality ratio (95% CI) [%]	I^2 [%]
**High and low-income countries**	Combined	274	8.8 (8.0–9.7)	89
**High-income countries**	Children	50	6.0 (4.7–7.6)	54
**High-income countries**	Adults	22	9.5 (6.8–13.0)	76
**Low-income countries**	Children	26	10.4 (7.7–14.0)	62
**Low-income countries**	Adults	8	17.3 (12.4–23.8)	63

© 2024 van Ettekoven CN et al. *JAMA Network Open.*

***H. influenzae* meningitis**

eTable 18. Study characteristics and unadjusted case fatality ratios in *H. influenzae* meningitis per subgroup.

	Study periods, k	Episodes, n	Deaths, n	Case fatality ratio (95% CI) [%]
**All studies**	226	49,539	6,365	12.8 (12.5–13.2)
**Children**	71	5,382	586	10.9 (10.0–11.8)
**High-income countries**	154	44,899	5,222	11.6 (11.3–11.9)
**Low-income countries**	72	4,640	1,143	24.6 (23.2–26.1)

eTable 19. Results of the meta–analyses of case fatality ratios in *H. influenzae* meningitis per subgroup.

Human Development Index	Age group	Study periods, k	Case fatality ratio (95% CI) [%]	I^2 [%]
**High and low-income countries**	Combined	249	10.9 (9.5–12.5)	91
**High-income countries**	Children	59	6.6 (5.0–8.5)	92
**Low-income countries**	Children	29	23.3 (18.7–28.6)	76

© 2024 van Ettekoven CN et al. *JAMA Network Open.*

***L. monocytogenes* meningitis**

eTable 20. Study characteristics and unadjusted case fatality ratios in *L. monocytogenes* meningitis per subgroup.

	**Study periods (k)**	**Episodes (n)**	**Deaths (n)**	**Case fatality ratio (95% CI) [%]**
**All studies**	70	1,829	497	27.2 (24.8–29.6)
**Adults**	23	702	203	28.9 (24.9–32.9)
**High-income countries**	68	1,796	487	27.1 (24.7–29.5)
**Low-income countries**	2	33	10	30.3 (11.5–49.1)

eTable 21. Results of the meta–analyses of case fatality ratios in *L. monocytogenes* meningitis per subgroup.

**Human Development Index**	**Age group**	**n**	**Case fatality ratio (95% CI) [%]**	**I^2 [%]**
**High and low-income countries**	Combined	77	27.2 (24.1–30.7)	21
**High-income countries**	Adults	25	29.4 (26.0–32.9)	0
**Low-income countries**	Adults	1	31.2 (17.7–49.0)	NA

© 2024 van Ettekoven CN et al. *JAMA Network Open.*

***E. coli* meningitis**

eTable 22. Study characteristics and unadjusted case fatality ratios in *E. coli* meningitis per subgroup.

	Studies (n)	Episodes (n)	Deaths (n)	Case fatality ratio (95% CI) [%]
**All studies**	90	2,137	412	19.3 (17.4–21.1)
**Neonates**	30	619	156	25.2 (21.2–29.2)
**High-income countries**	74	2,073	382	18.4 (16.6–20.3)
**Low-income countries**	16	64	30	46.9 (30.1–63.6)

eTable 23. Results of the meta–analyses of case fatality ratios in *E. coli* meningitis per subgroup.

Human Development Index	Age group	n	Case fatality ratio (95% CI) [%]	I^2 [%]
**High and low-income countries**	Combined	100	34.0 (27.8–40.8)	72
**High-income countries**	Neonates	33	29.2 (19.5–41.1)	71
**Low-income countries**	Neonates	5	47.5 (31.6–63.9)	0

© 2024 van Ettekoven CN et al. *JAMA Network Open.*

***S. agalactiae* meningitis**

eTable 24. Study characteristics and unadjusted case fatality ratios in *S. agalactiae* meningitis per subgroup.

	Studies (n)	Episodes (n)	Deaths (n)	Case fatality ratio (95% CI) [%]
**All studies**	76	4,584	534	11.6 (10.7–12.6)
**Neonates**	30	1,431	159	11.1 (9.4–12.8)
**High-income countries**	66	4,341	476	11.0 (10.0–12.0)
**Low-income countries**	10	243	58	23.9 (17.7–30.0)

eTable 25. Results of the meta–analyses of case fatality ratios in *S. agalactiae* meningitis per subgroup.

Human Development Index	Age group	n	Case fatality ratio (95% CI) [%]	I^2 [%]
**High and low-income countries**	Combined	86	16.0 (13.1–19.4)	66
**High-income countries**	Neonates	35	13.8 (10.1–18.6)	67
**Low-income countries**	Neonates	3	32.0 (10.6–65.1)	83

© 2024 van Ettekoven CN et al. *JAMA Network Open.*

EFIGURES

Study flow-chart

eFigure 1. Flow–chart of the study selection process.

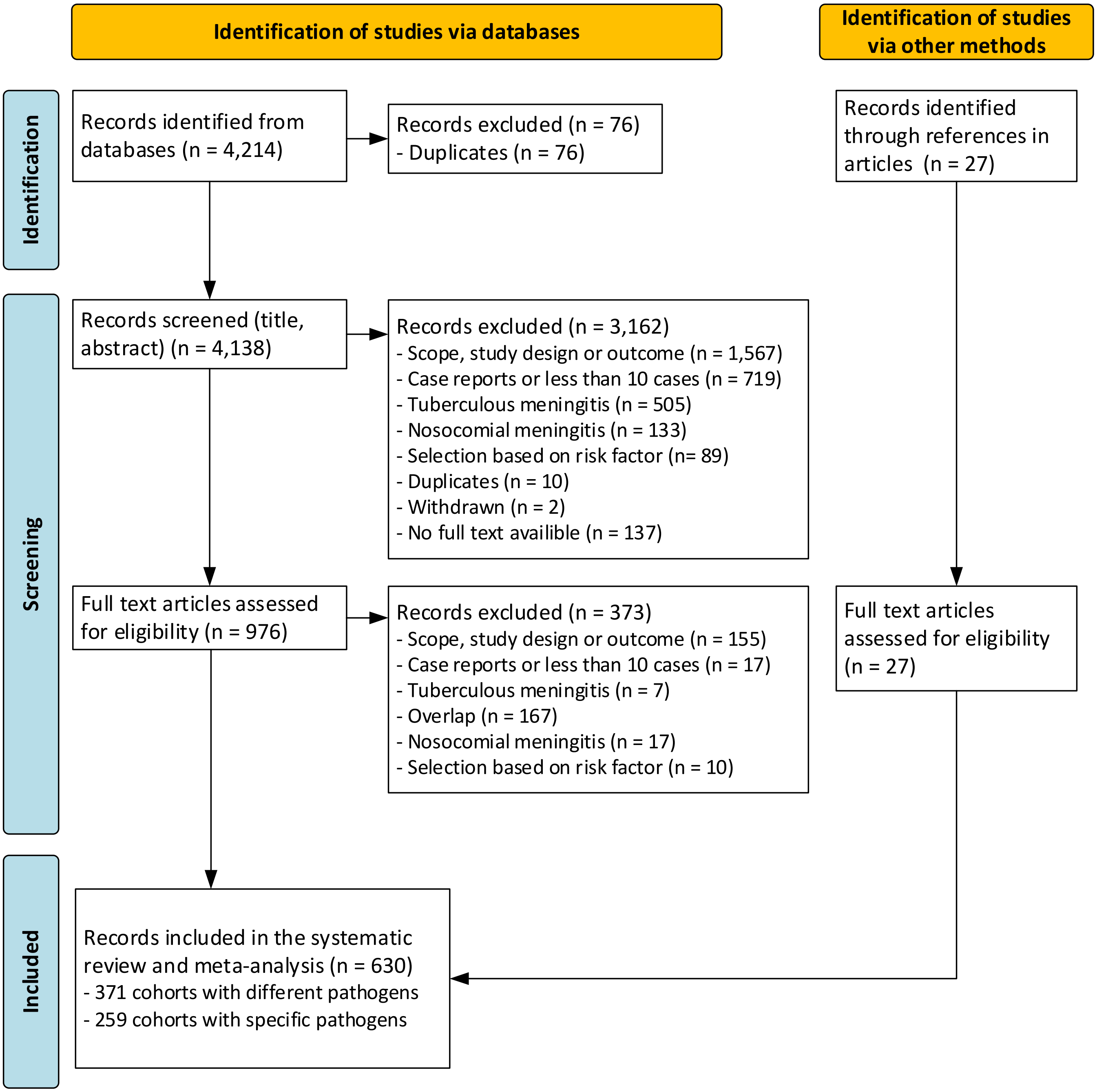

All pathogens

eFigure 2. Geographic distribution of studies evaluating case fatality ratios in bacterial meningitis and included into the meta–analysis. Studies were performed in 96 countries from Africa (n = 94; 49,794 episodes), Europe (n = 93; 31,885 episodes), Asia (n = 91; 14,517 episodes), Americas (n = 78; 59,364 episodes), and Oceania (n = 15; 2,096 episodes).***

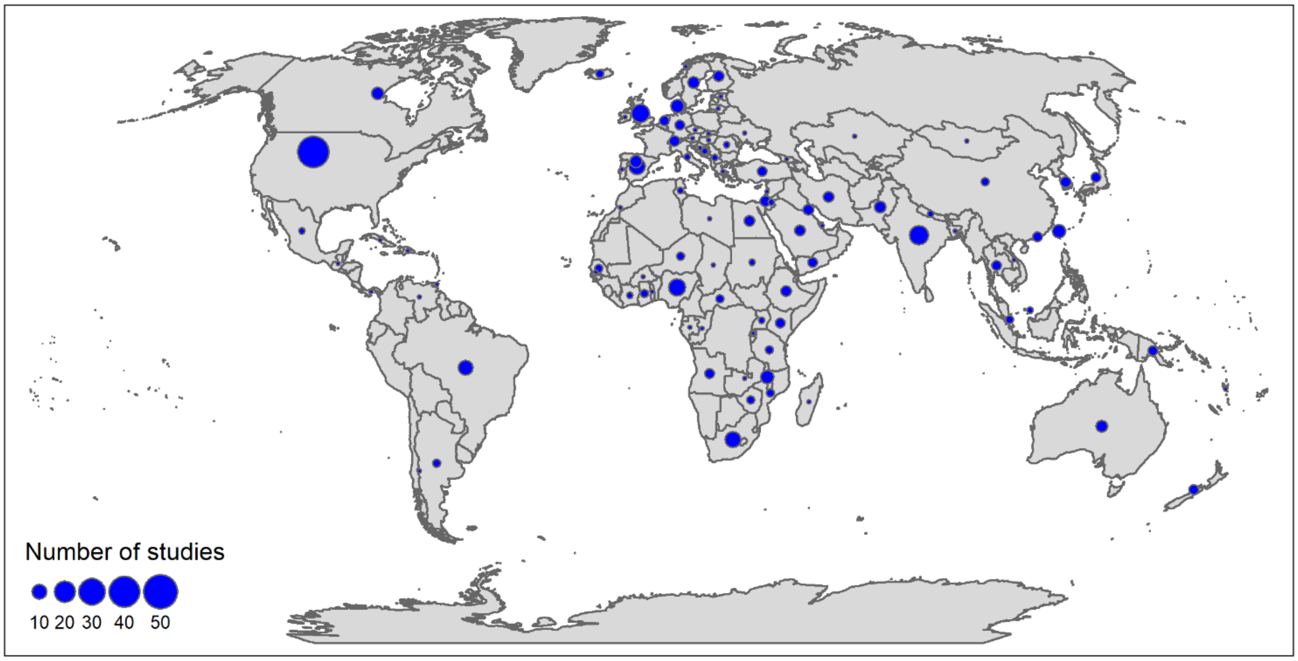

*** 8 multinational studies adding 8 more countries are not shown

efigure 3. Proportions of causative pathogens per time interval, stratified by age group (neonates, 0 to 2 months; children, 2 months to 16 years; adults).

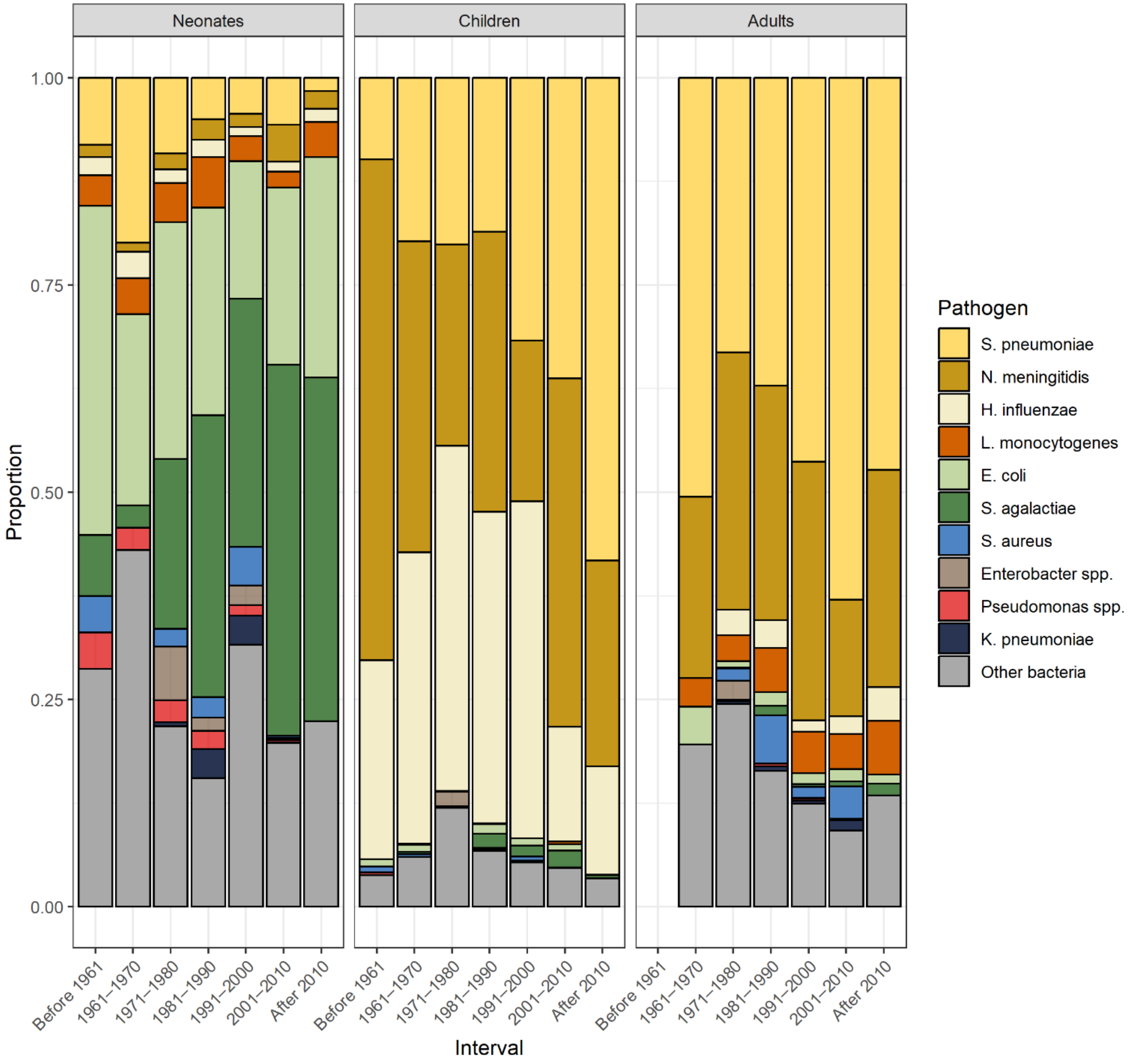

efigure 4. Proportions of causative pathogens per time interval, stratified by country group (high-income, low income).

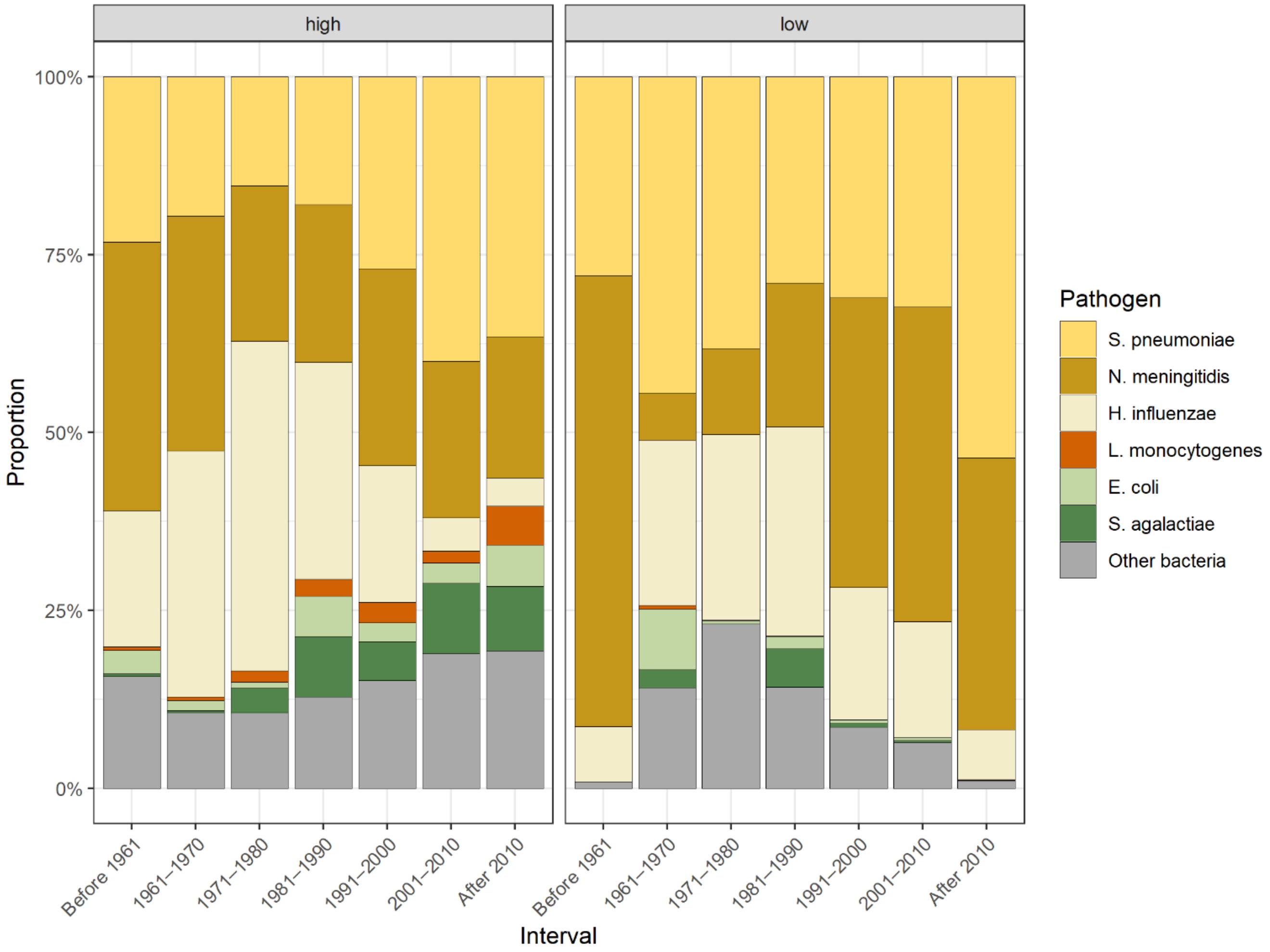

eFigure 5. Mean case fatality ratios of bacterial meningitis per country, stratified by time intervals.

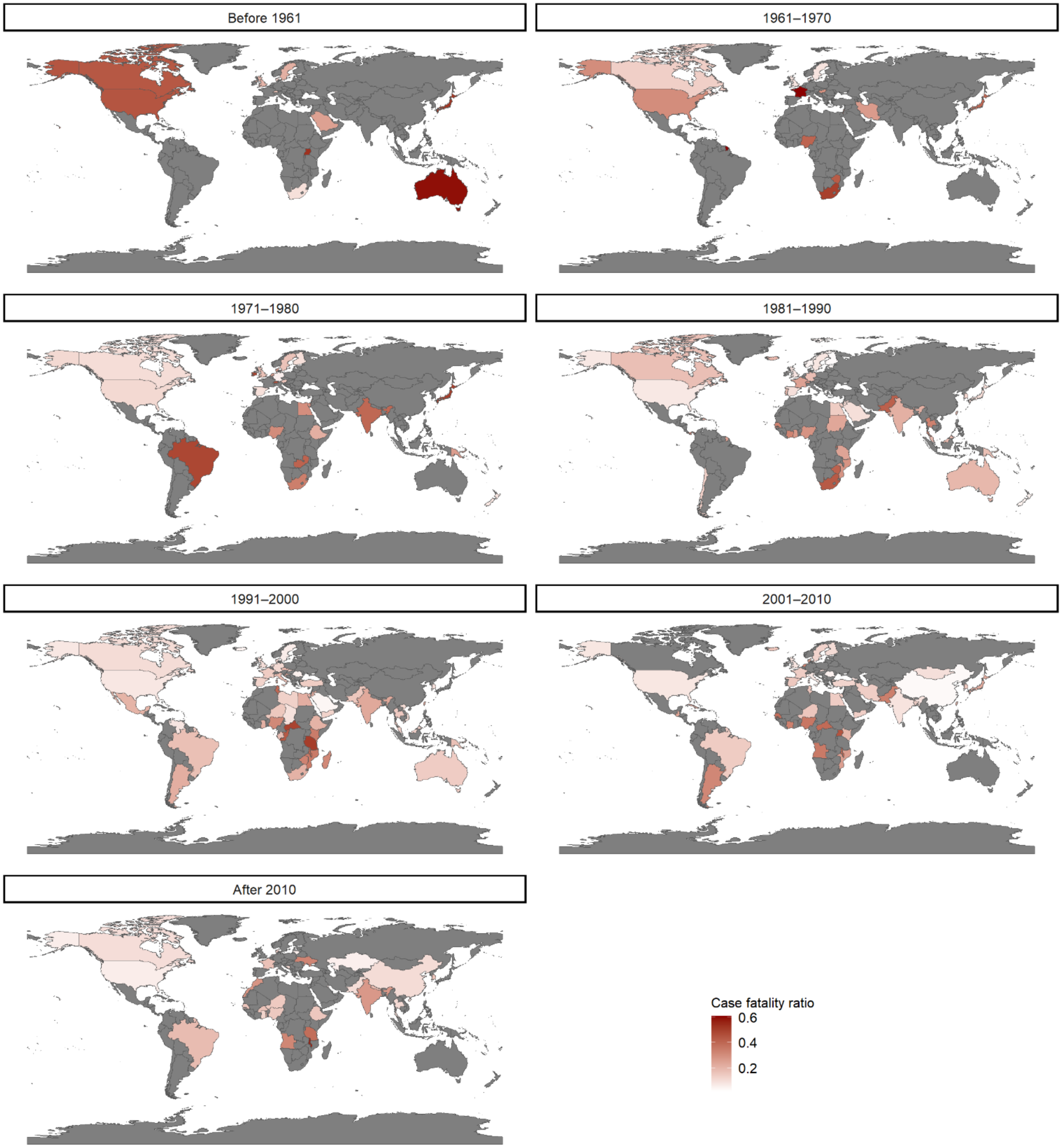

eFigure 6. Funnel plot of all studies included. (dotted line, random effects estimate)

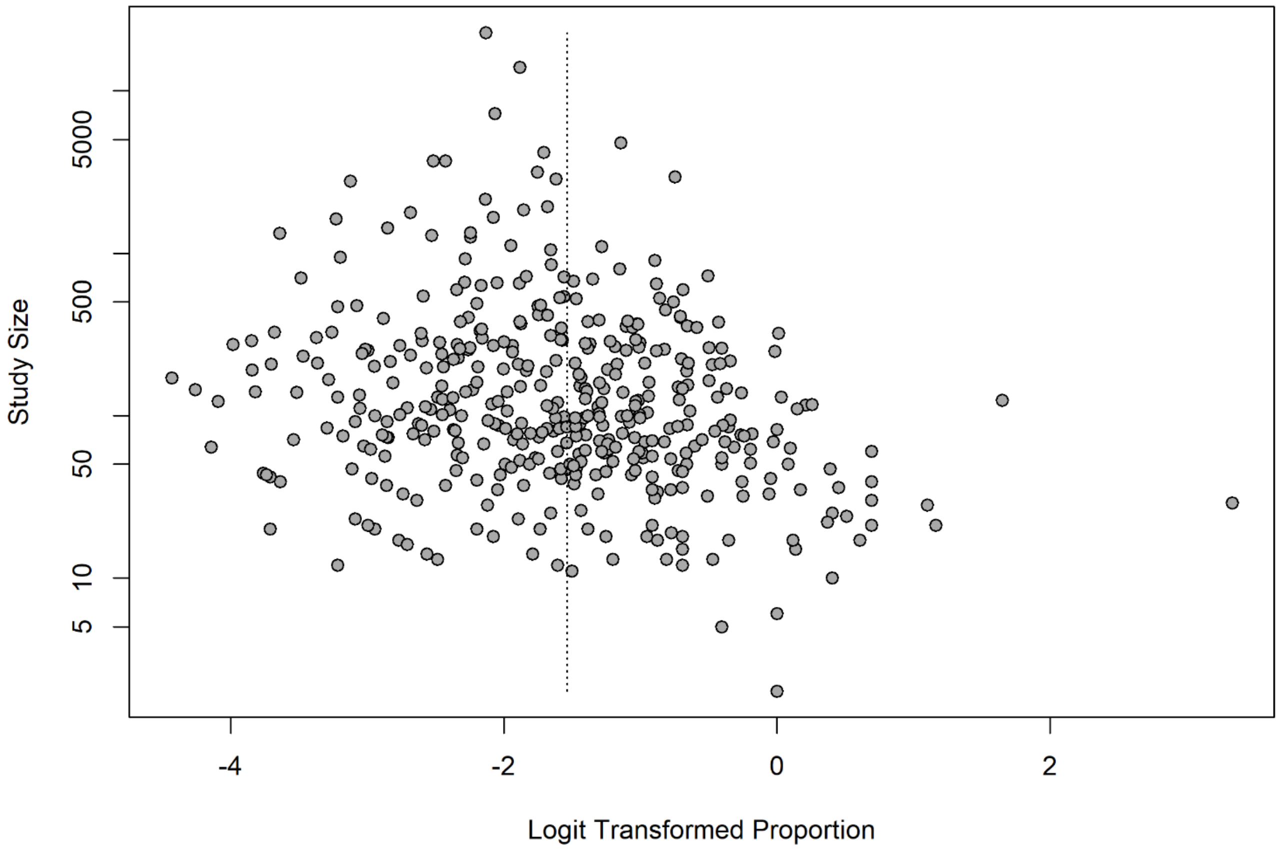

eFigure 7. Funnel plots of the meta–analyses, stratified by age groups (neonates, 0 to 2 months; children, 2 months to 16 years; adults) and country group (high-income, low-income). (dashed line, random effects estimate)

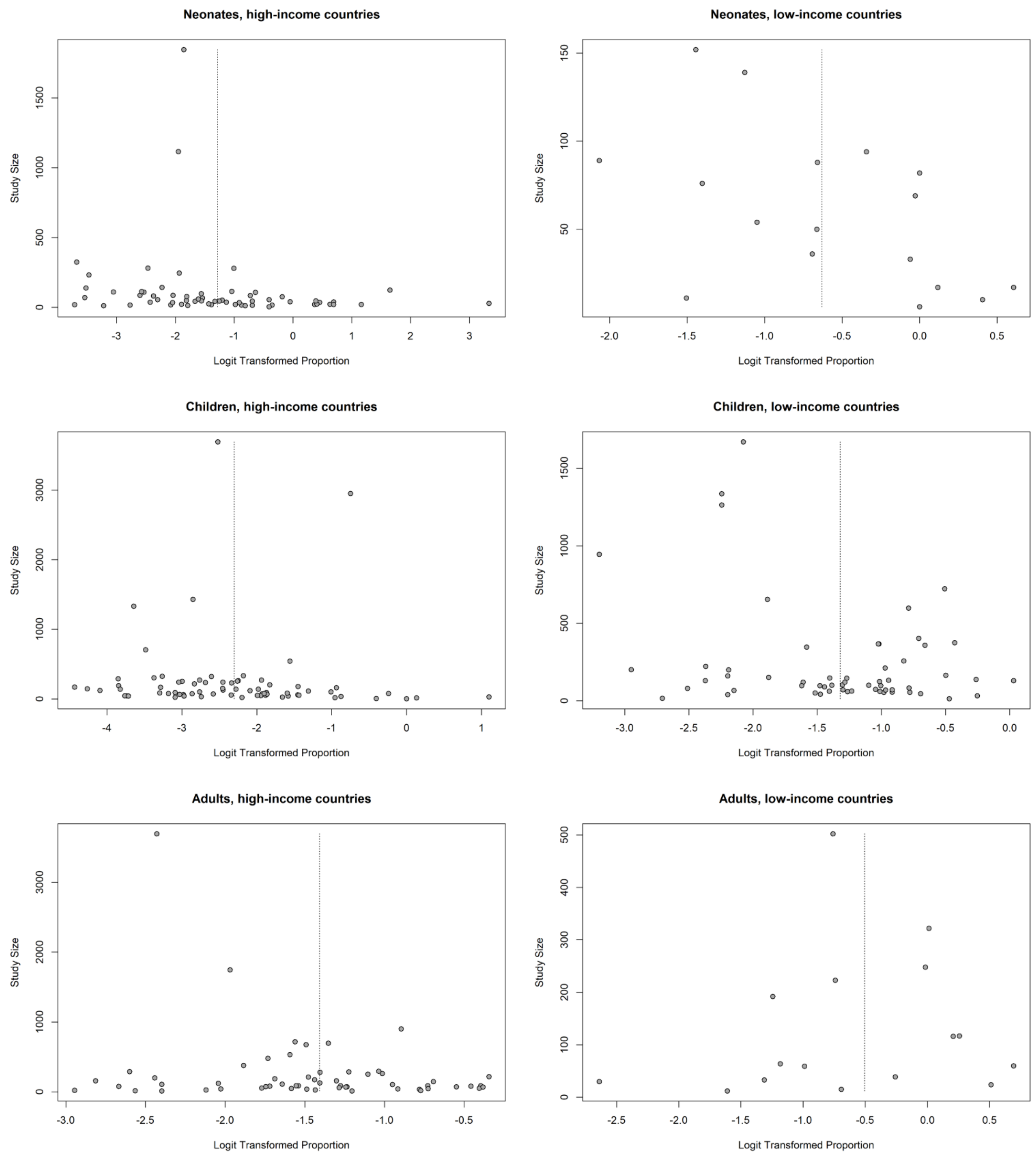

eFigure 8. Forest plot of studies in high-income countries on neonates.

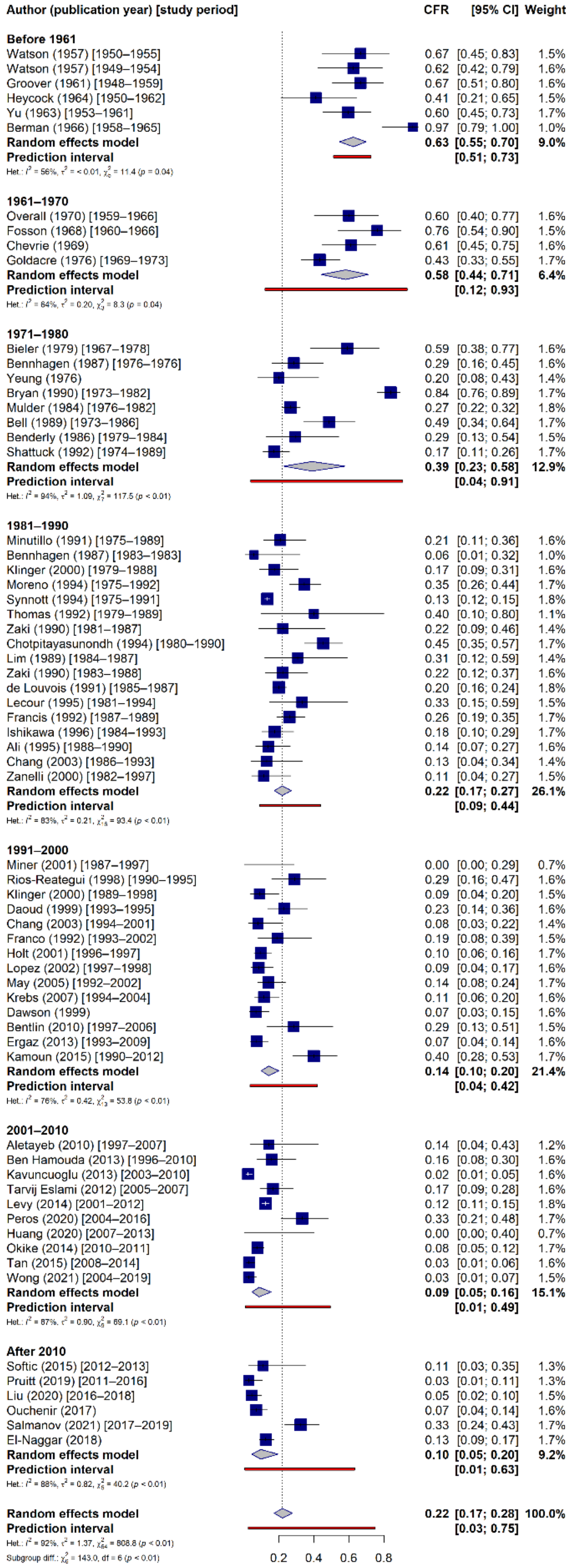

eFigure 9. Forest plot of studies in high-income countries on children.

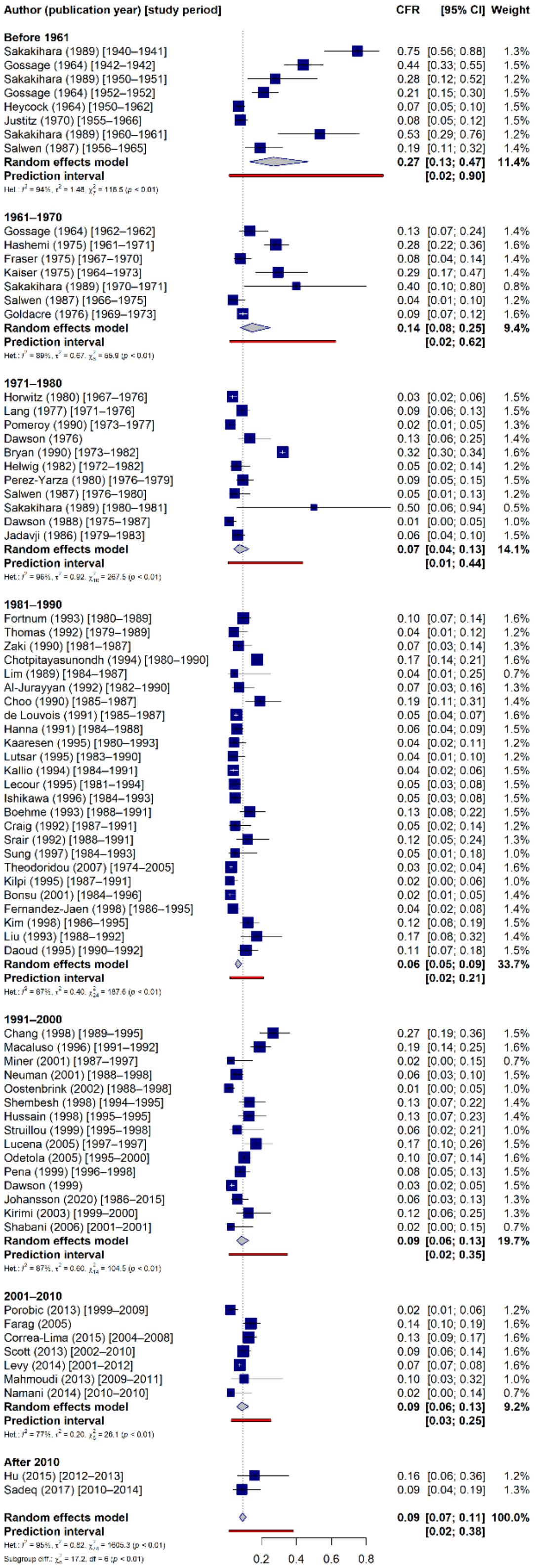

eFigure 10. Forest plot of studies in <u>high-income</u> countries on <u>adults</u>.

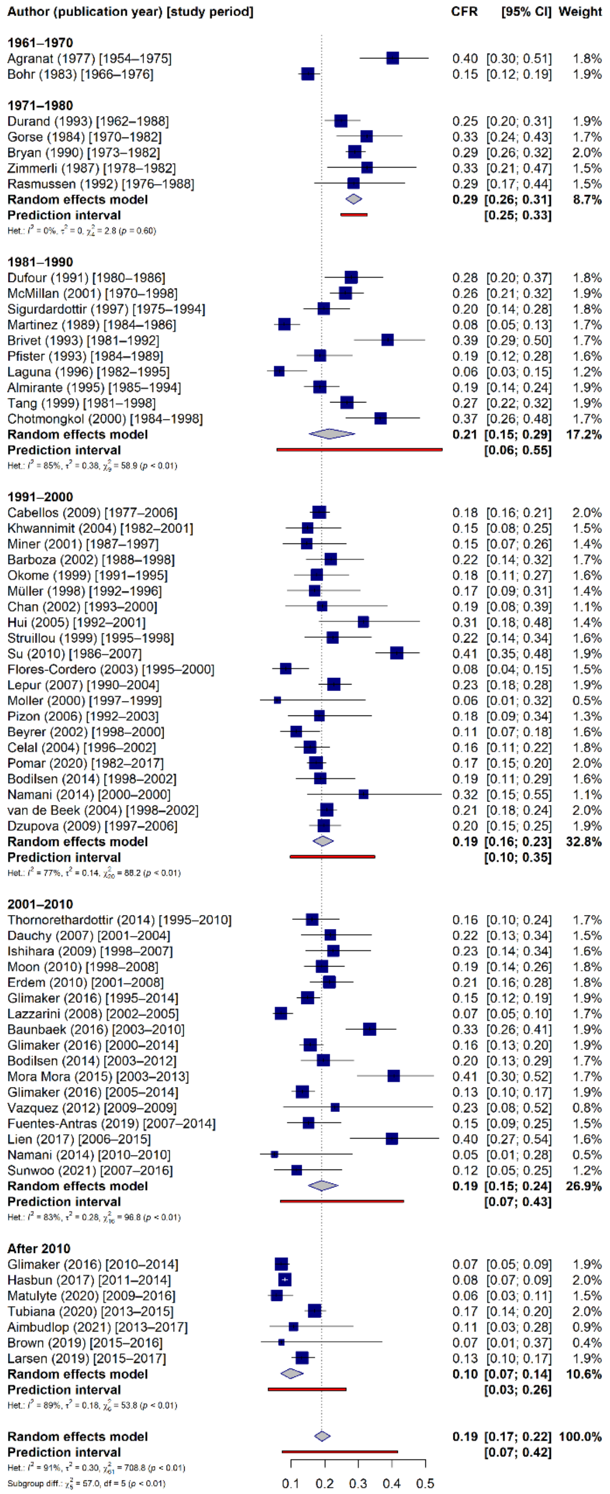

eFigure 11. Forest plot of studies in low-income countries on neonates.

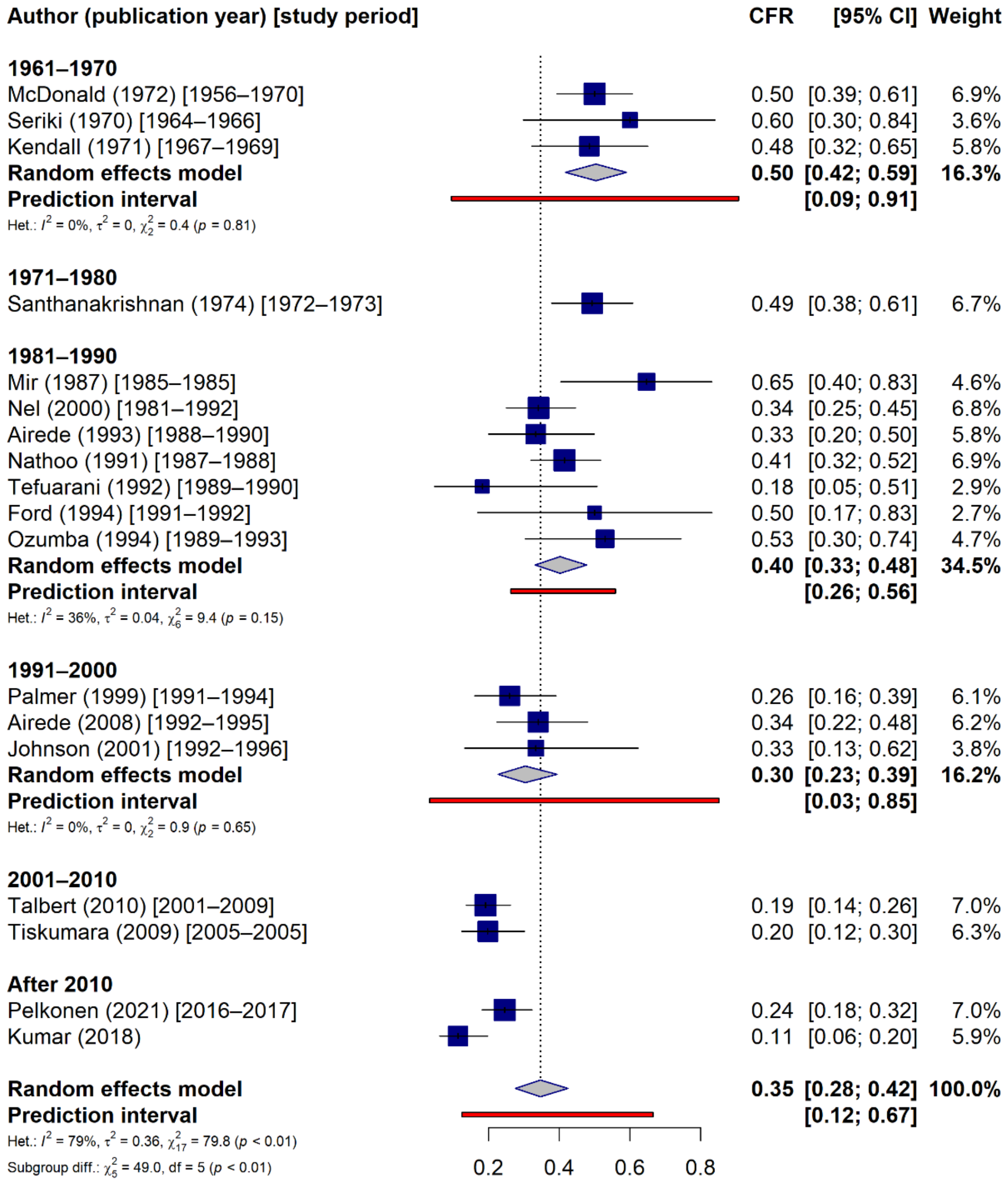

eFigure 12. Forest plot of studies in <u>low-income</u> countries on <u>children</u>.

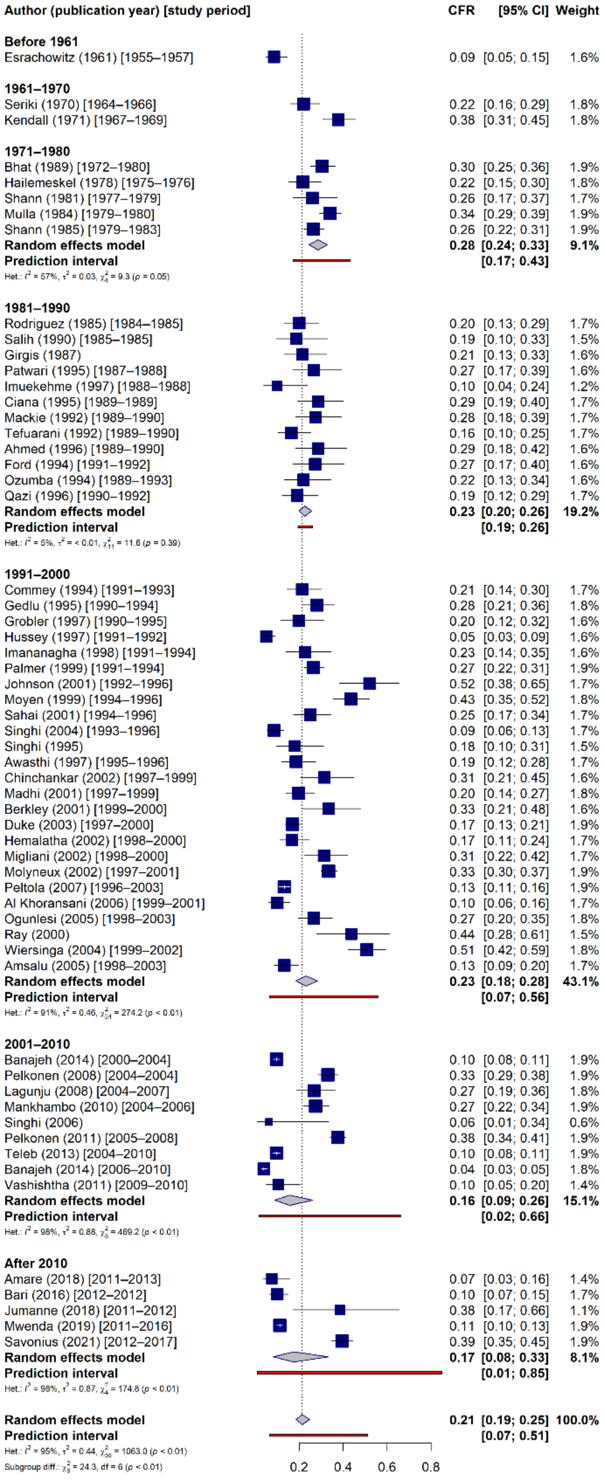

eFigure 13. Forest plot of studies in low-income countries on adults.

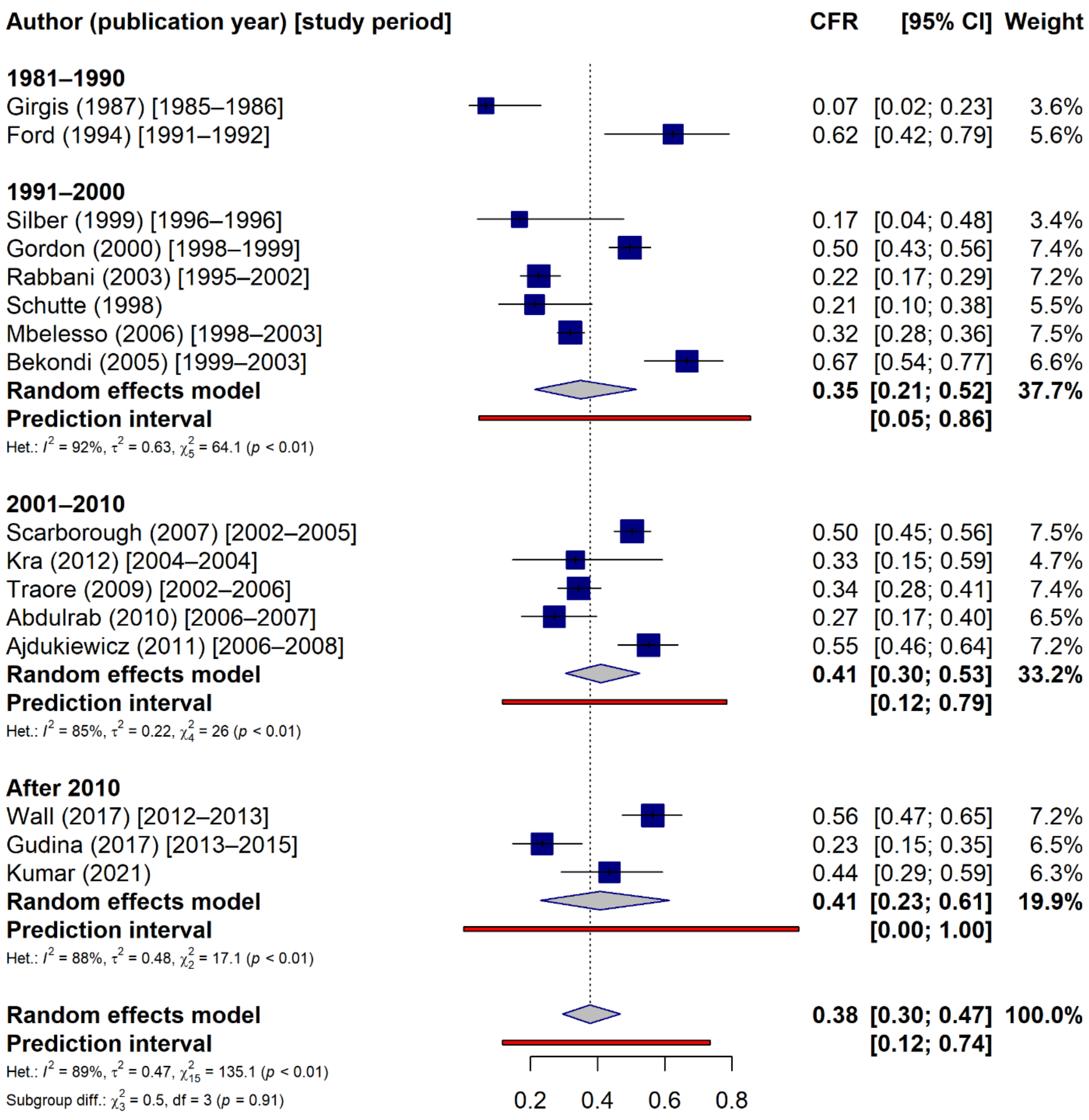

eFigure 14. Case fatality ratio of patients with bacterial meningitis using a meta–regression model with the studies' mean observation year as predictor variable ($P < 0.001$). (dashed lines, 95% CI; dotted lines, prediction interval)

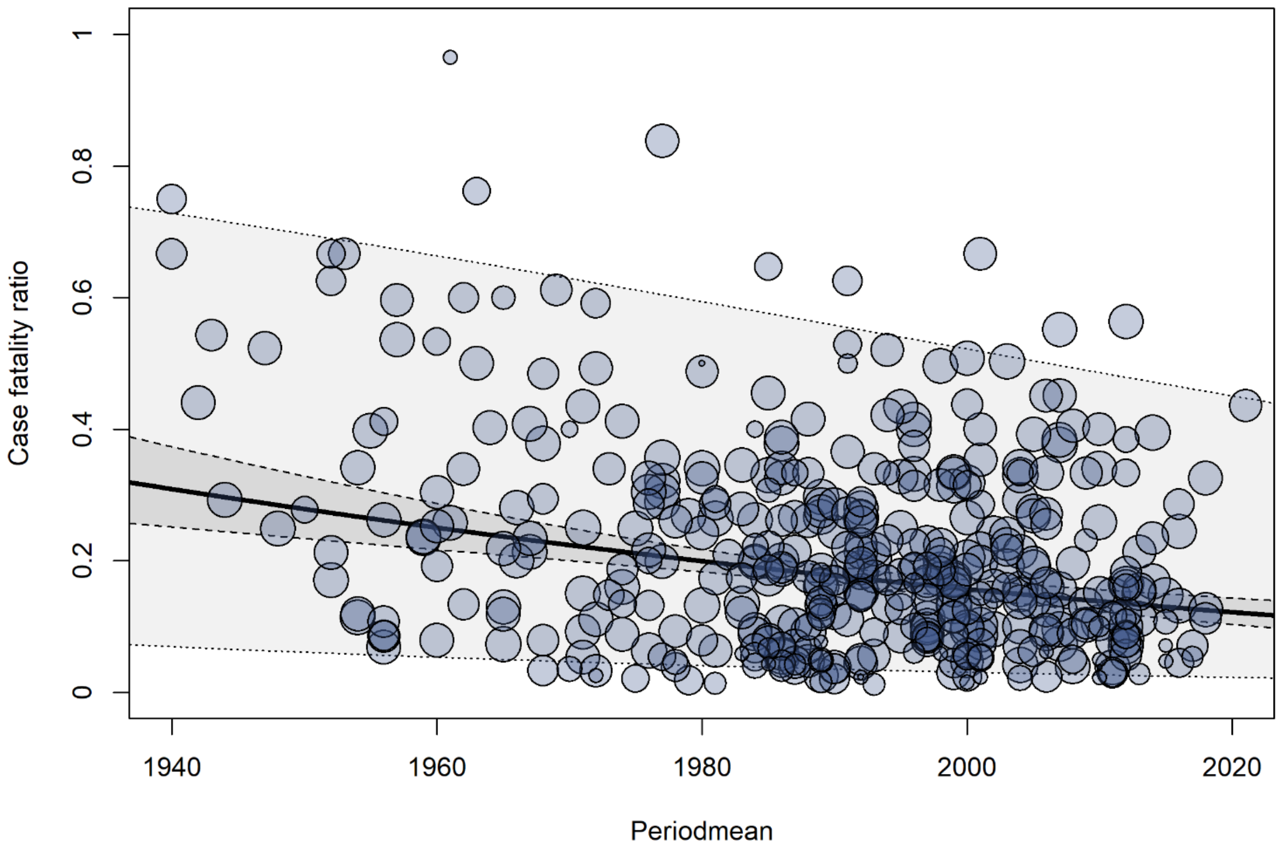

eFigure 15. Case fatality ratios of patients with bacterial meningitis using a meta-regression model with the studies' mean observation year as predictor variable, stratified according to age groups (neonates, < 2 months; children, 2 months to 16 years; adults, >16 years) in low-income countries.

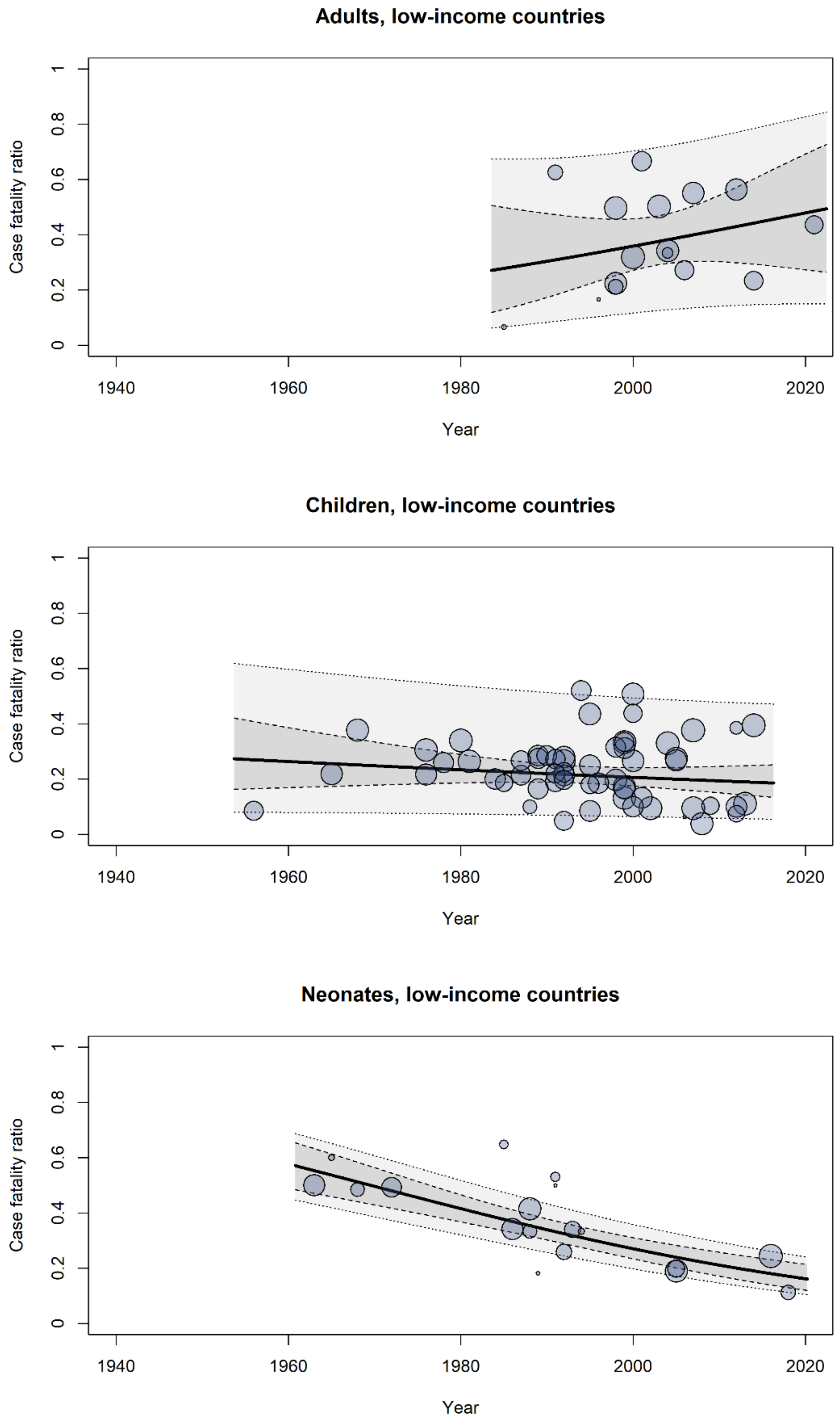

Pneumococcal meningitis

eFigure 16. Geographic distribution of studies evaluating case fatality ratios in pneumococcal meningitis and included into the meta–analysis.

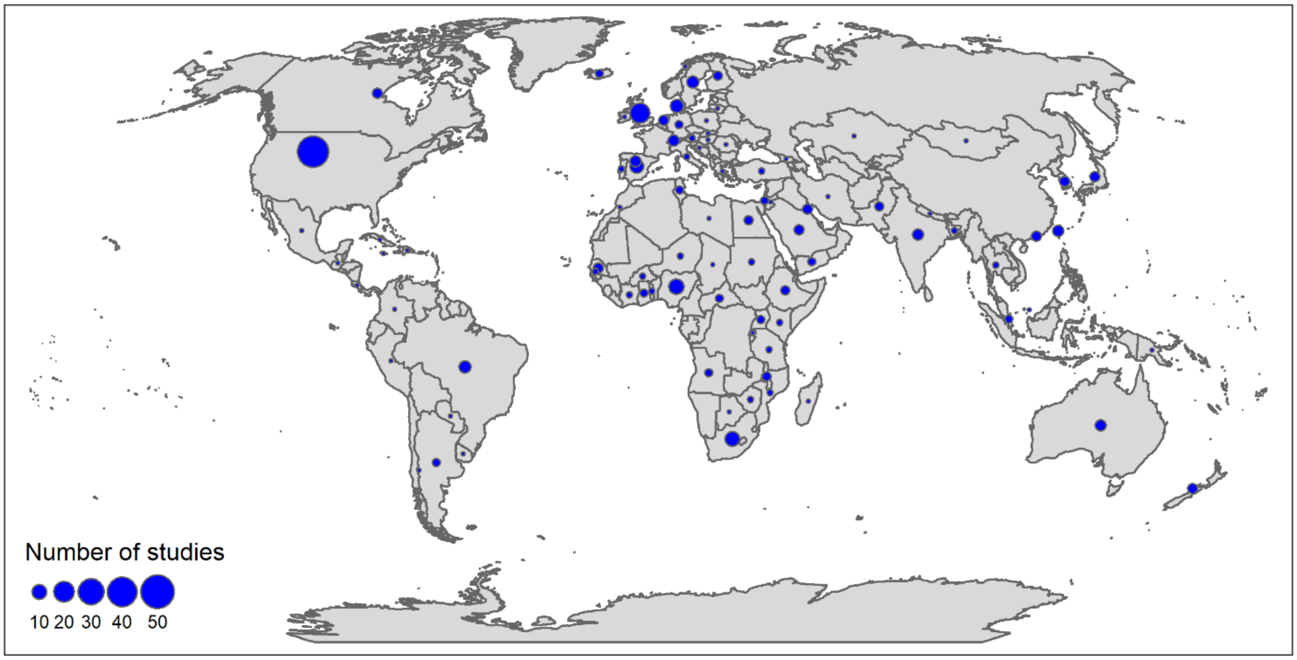

© 2024 van Ettekoven CN et al. *JAMA Network Open.*

eFigure 17. Case fatality ratios in *S. pneumoniae* meningitis (Forest plot with individual studies suppressed) indicating the overall pooled estimate and the estimates of the intervals as subgroups[†††].

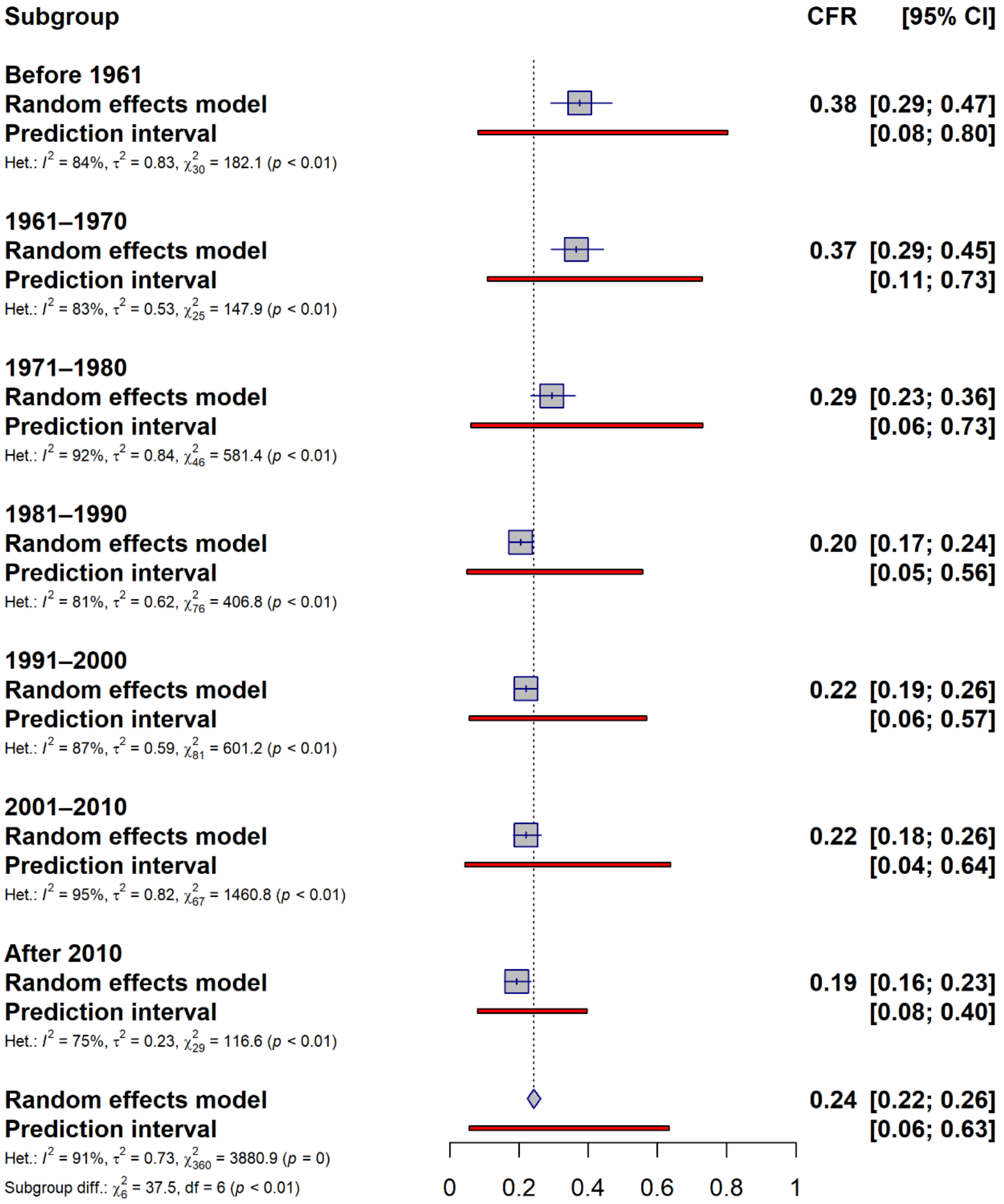

[†††] Before 1961, k = 31; 1961–1970, k = 26; 1971–1980, k = 47; 1981–1990, k = 77; 1991–2000, k = 82; 2001–2010, k = 68; after 2010, k = 30). (Het., between–study heterogeneity

eFigure 18. Case fatality ratios of patients with pneumococcal meningitis using a meta–regression model with the studies' mean observation year as predictor variable ($P < 0.001$). (dashed lines, 95% CI; dotted lines, prediction interval)

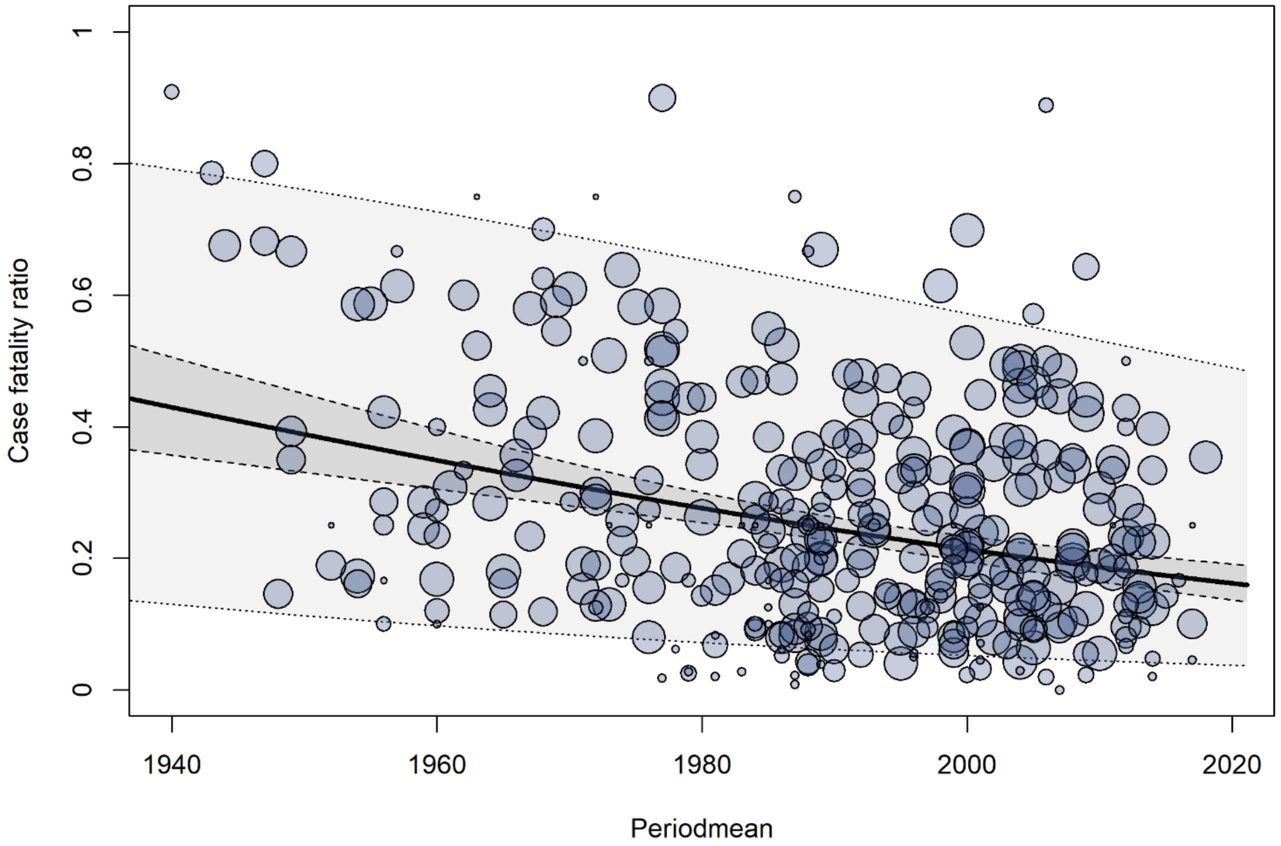

eFigure 19. Forest plot of studies in high-income countries on neonates with pneumococcal meningitis.

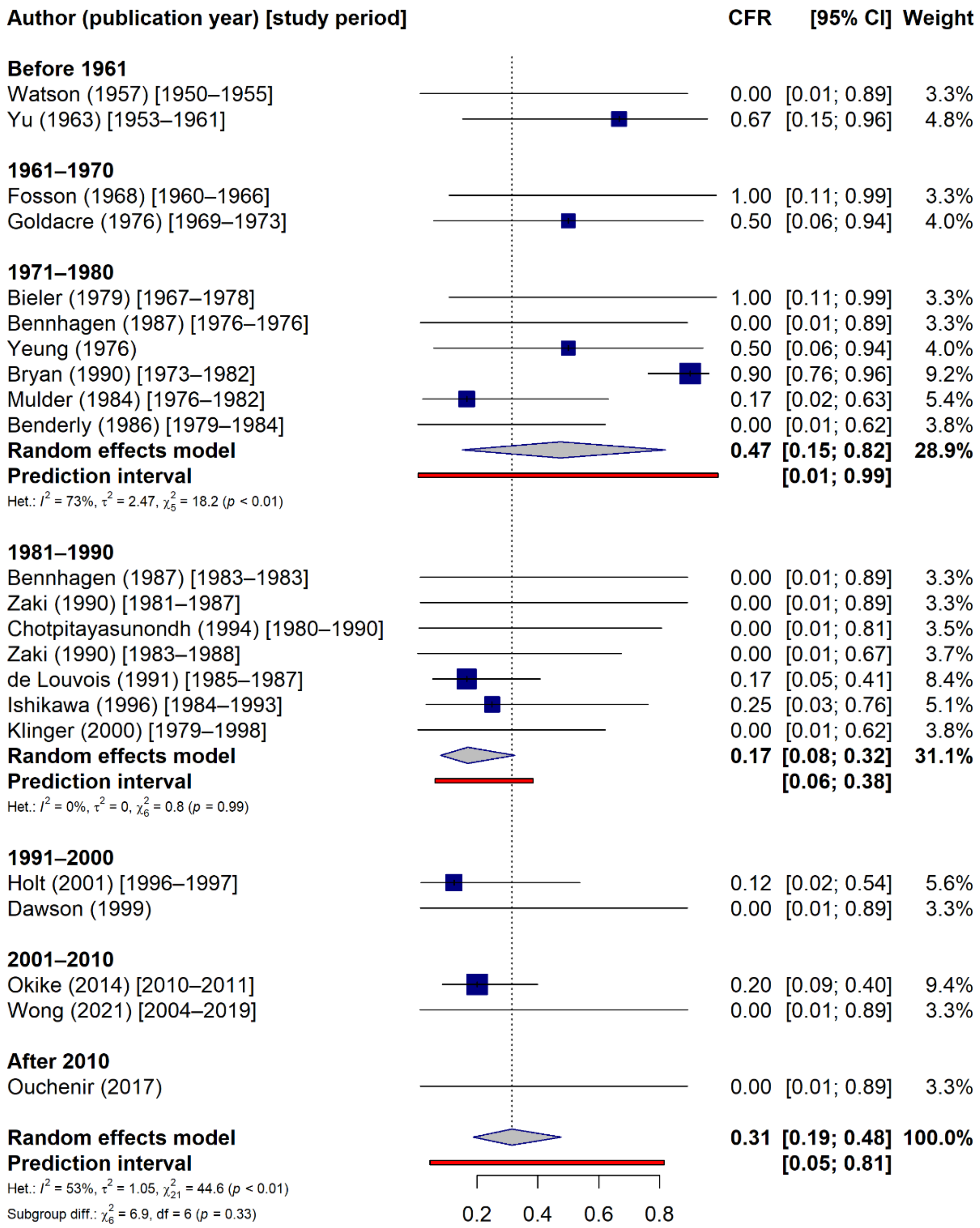

eFigure 20. Forest plot of studies in high-income countries on children with pneumococcal meningitis.

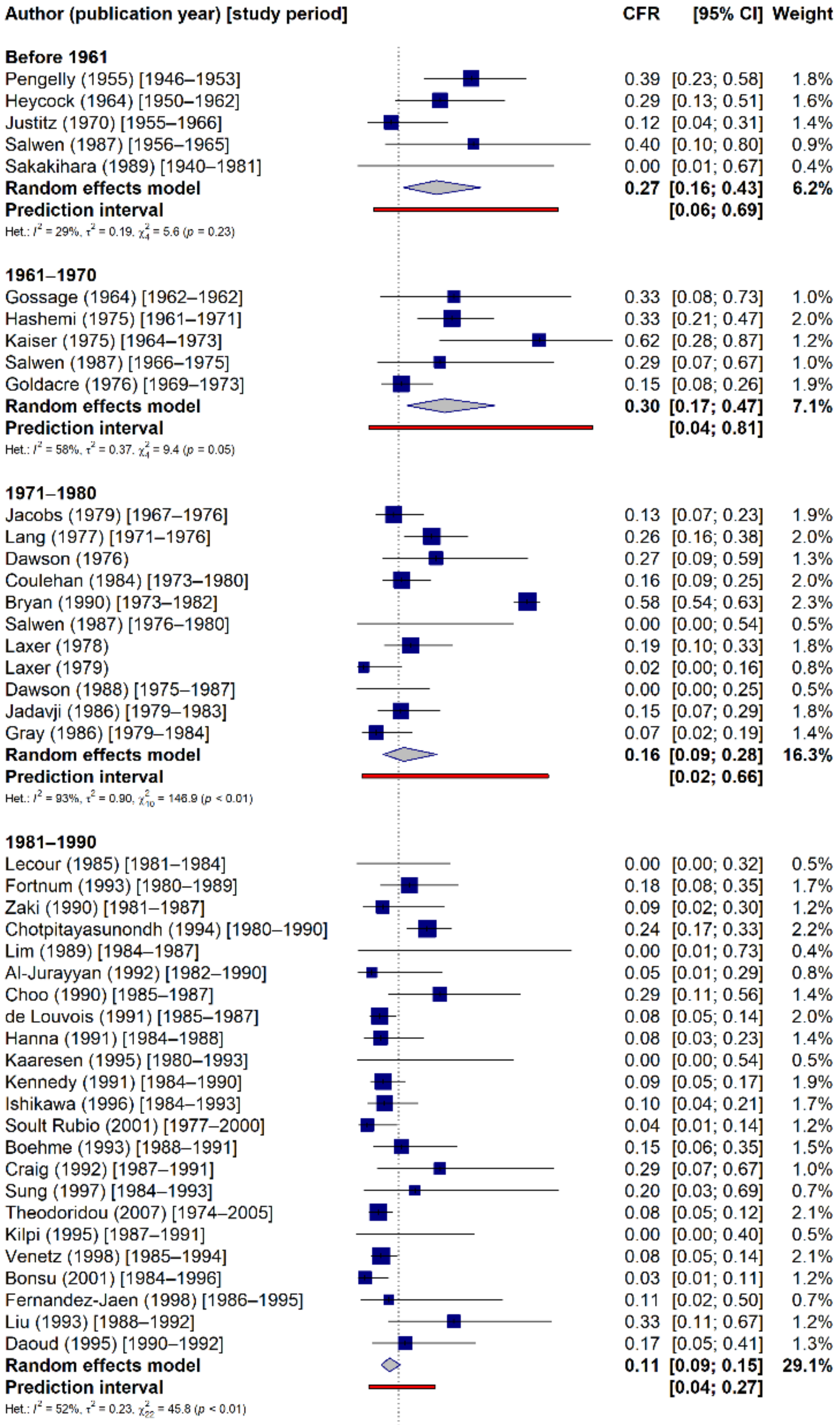

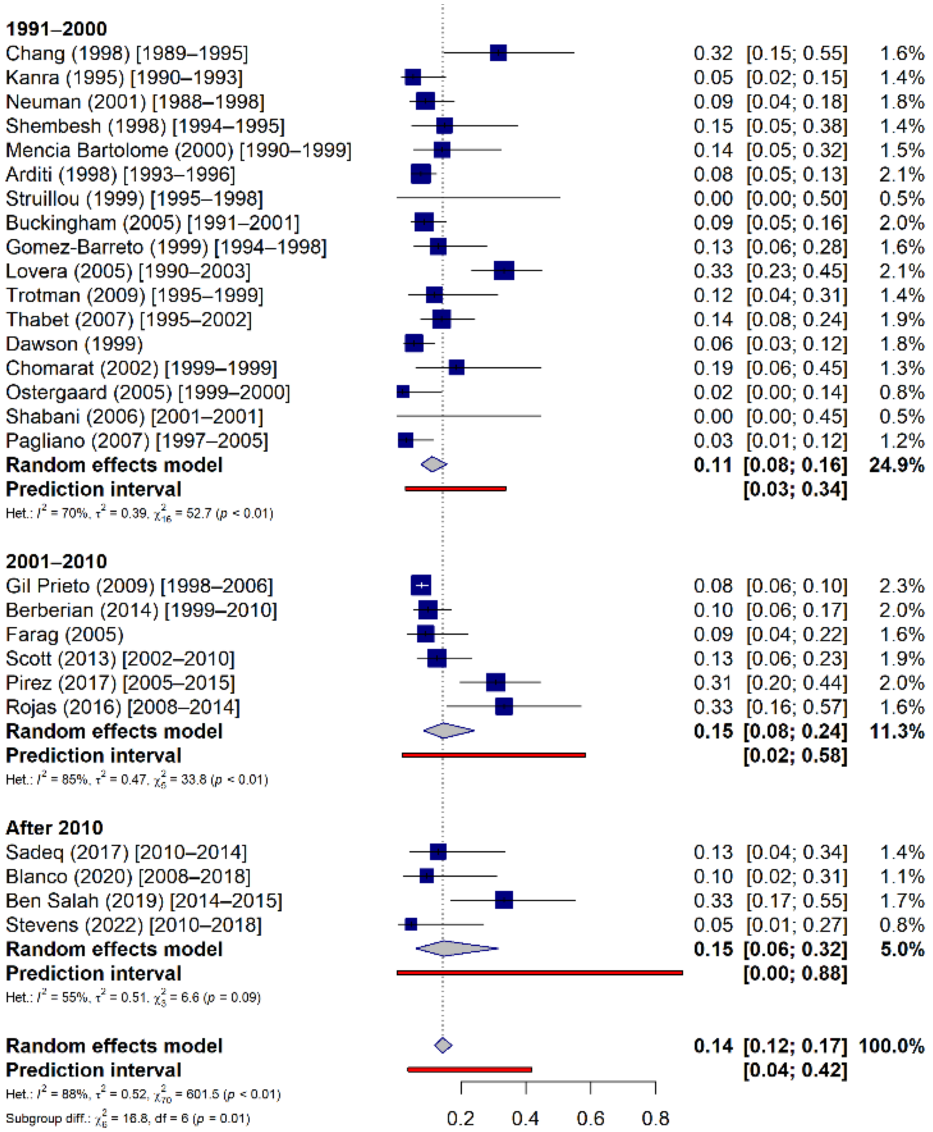
1991–2000
Chang (1998) [1989–1995] 0.32 [0.15; 0.55] 1.6%
Kanra (1995) [1990–1993] 0.05 [0.02; 0.15] 1.4%
Neuman (2001) [1988–1998] 0.09 [0.04; 0.18] 1.8%
Shembesh (1998) [1994–1995] 0.15 [0.05; 0.38] 1.4%
Mencia Bartolome (2000) [1990–1999] 0.14 [0.05; 0.32] 1.5%
Arditi (1998) [1993–1996] 0.08 [0.05; 0.13] 2.1%
Struillou (1999) [1995–1998] 0.00 [0.00; 0.50] 0.5%
Buckingham (2005) [1991–2001] 0.09 [0.05; 0.16] 2.0%
Gomez-Barreto (1999) [1994–1998] 0.13 [0.06; 0.28] 1.6%
Lovera (2005) [1990–2003] 0.33 [0.23; 0.45] 2.1%
Trotman (2009) [1995–1999] 0.12 [0.04; 0.31] 1.4%
Thabet (2007) [1995–2002] 0.14 [0.08; 0.24] 1.9%
Dawson (1999) 0.06 [0.03; 0.12] 1.8%
Chomarat (2002) [1999–1999] 0.19 [0.06; 0.45] 1.3%
Ostergaard (2005) [1999–2000] 0.02 [0.00; 0.14] 0.8%
Shabani (2006) [2001–2001] 0.00 [0.00; 0.45] 0.5%
Pagliano (2007) [1997–2005] 0.03 [0.01; 0.12] 1.2%
Random effects model 0.11 [0.08; 0.16] 24.9%
Prediction interval [0.03; 0.34]
Het.: I^2 = 70%, τ^2 = 0.39, χ^2_{16} = 52.7 (p < 0.01)
2001–2010
Gil Prieto (2009) [1998–2006] 0.08 [0.06; 0.10] 2.3%
Berberian (2014) [1999–2010] 0.10 [0.06; 0.17] 2.0%
Farag (2005) 0.09 [0.04; 0.22] 1.6%
Scott (2013) [2002–2010] 0.13 [0.06; 0.23] 1.9%
Pirez (2017) [2005–2015] 0.31 [0.20; 0.44] 2.0%
Rojas (2016) [2008–2014] 0.33 [0.16; 0.57] 1.6%
Random effects model 0.15 [0.08; 0.24] 11.3%
Prediction interval [0.02; 0.58]
Het.: I^2 = 85%, τ^2 = 0.47, χ^2_5 = 33.8 (p < 0.01)
After 2010
Sadeq (2017) [2010–2014] 0.13 [0.04; 0.34] 1.4%
Blanco (2020) [2008–2018] 0.10 [0.02; 0.31] 1.1%
Ben Salah (2019) [2014–2015] 0.33 [0.17; 0.55] 1.7%
Stevens (2022) [2010–2018] 0.05 [0.01; 0.27] 0.8%
Random effects model 0.15 [0.06; 0.32] 5.0%
Prediction interval [0.00; 0.88]
Het.: I^2 = 55%, τ^2 = 0.51, χ^2_3 = 6.6 (p = 0.09)
Random effects model 0.14 [0.12; 0.17] 100.0%
Prediction interval [0.04; 0.42]
Het.: I^2 = 88%, τ^2 = 0.52, χ^2_{70} = 601.5 (p < 0.01)
Subgroup diff.: χ^2_6 = 16.8, df = 6 (p = 0.01)
0.2
0.4
0.6
0.8

eFigure 21. Forest plot of studies in high-income countries on adults with pneumococcal meningitis.

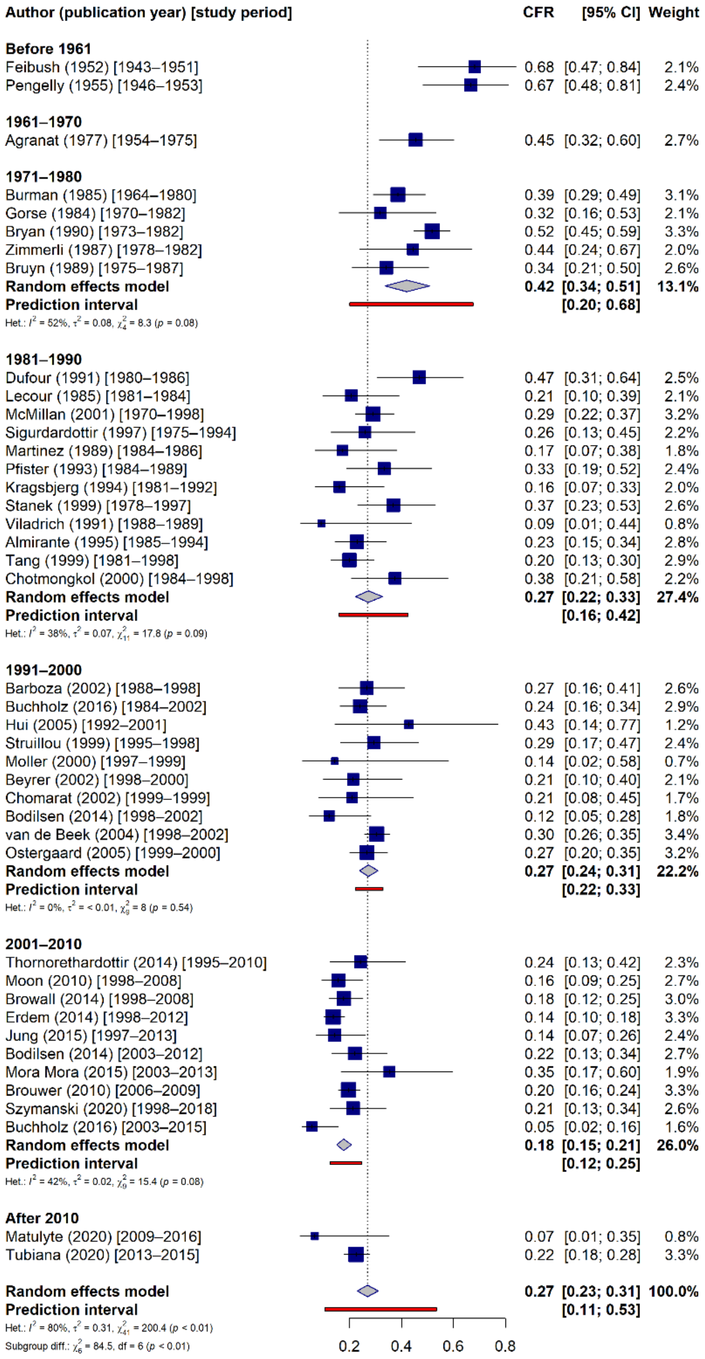

eFigure 22. Forest plot of studies in low-income countries on neonates with pneumococcal meningitis.

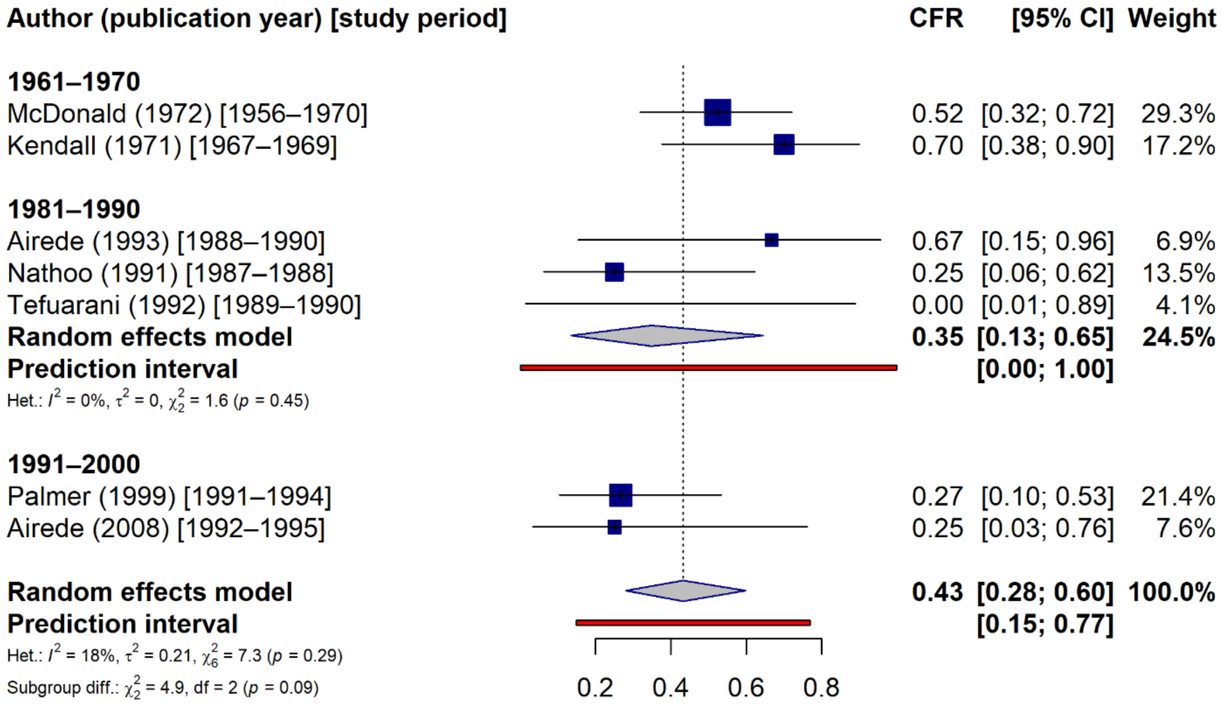

eFigure 23. Forest plot of studies in low-income countries on children with pneumococcal meningitis.

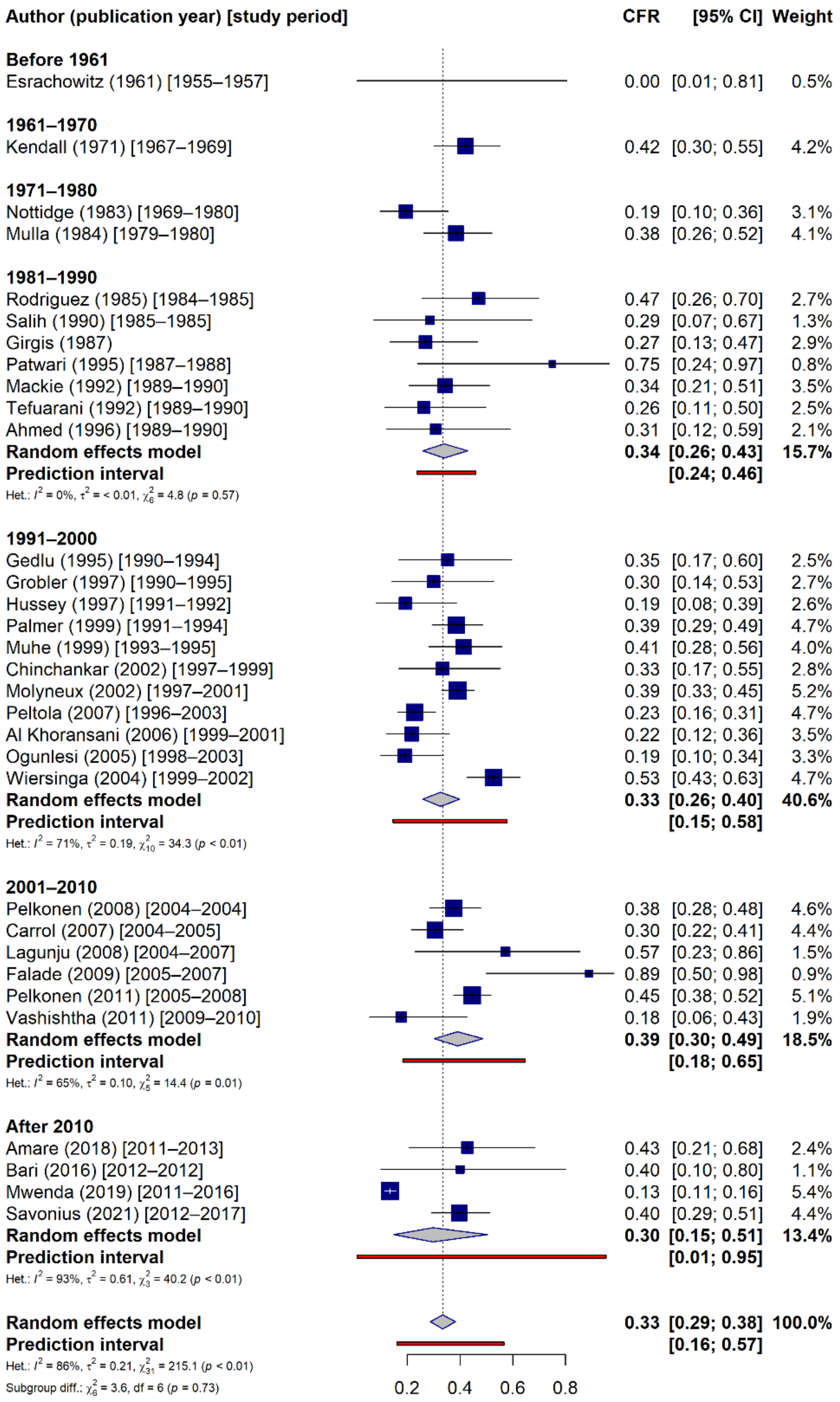

eFigure 24. Forest plot of studies in low-income countries on adults with pneumococcal meningitis.

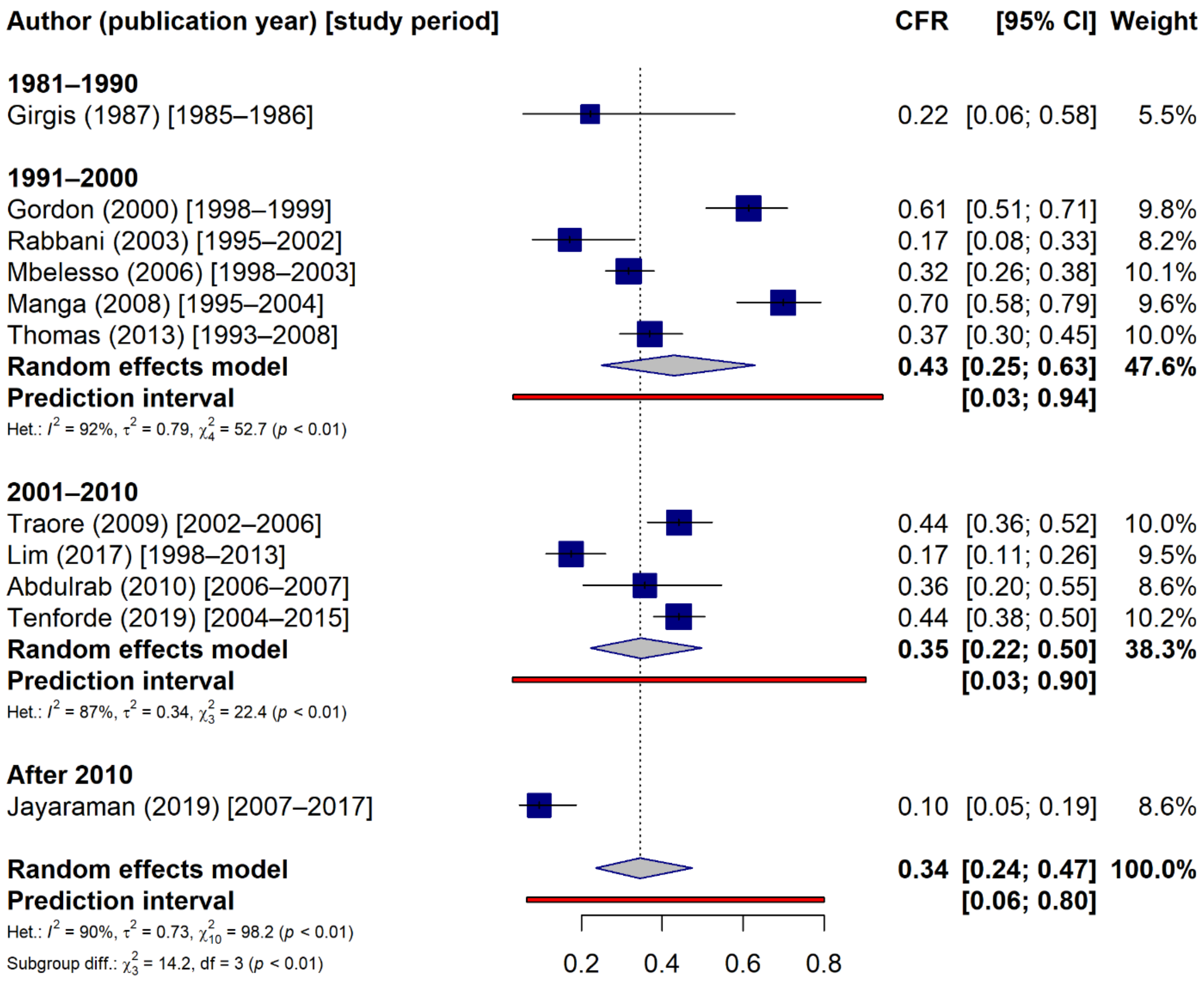

eFigure 25. Case fatality ratios of patients with pneumococcal meningitis using a meta–regression model with the studies' mean observation year as predictor variable, stratified according to the age group (neonates, 0 to 2 months; children, 2 months to 16 years; adults) and the Human Development Index (high-income countries, low-income countries). (dashed lines, 95% CI; dotted lines, prediction interval)

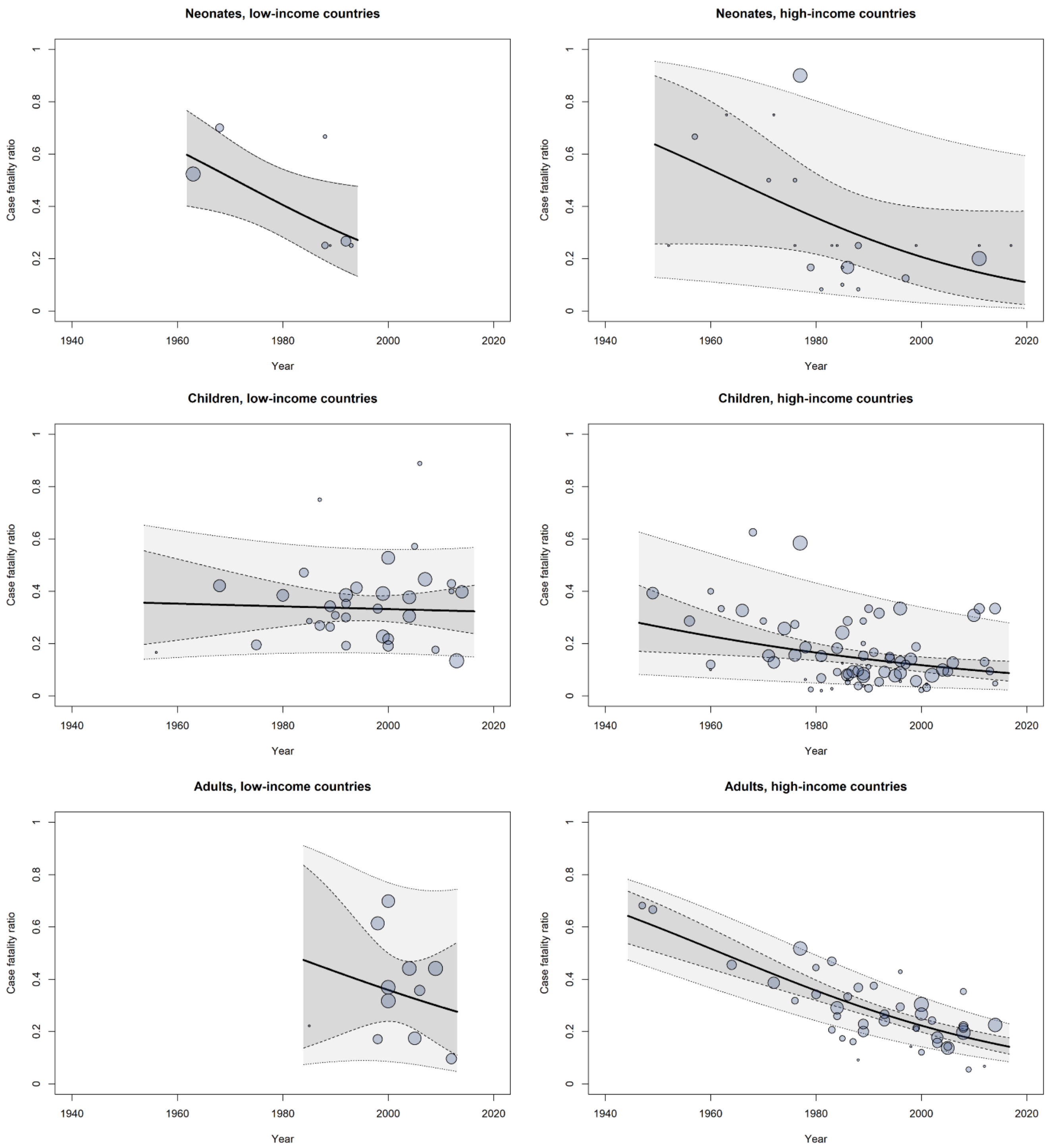

eFigure 26. Geographic distribution of studies evaluating case fatality ratios in pneumococcal meningitis and included into the meta–analysis.

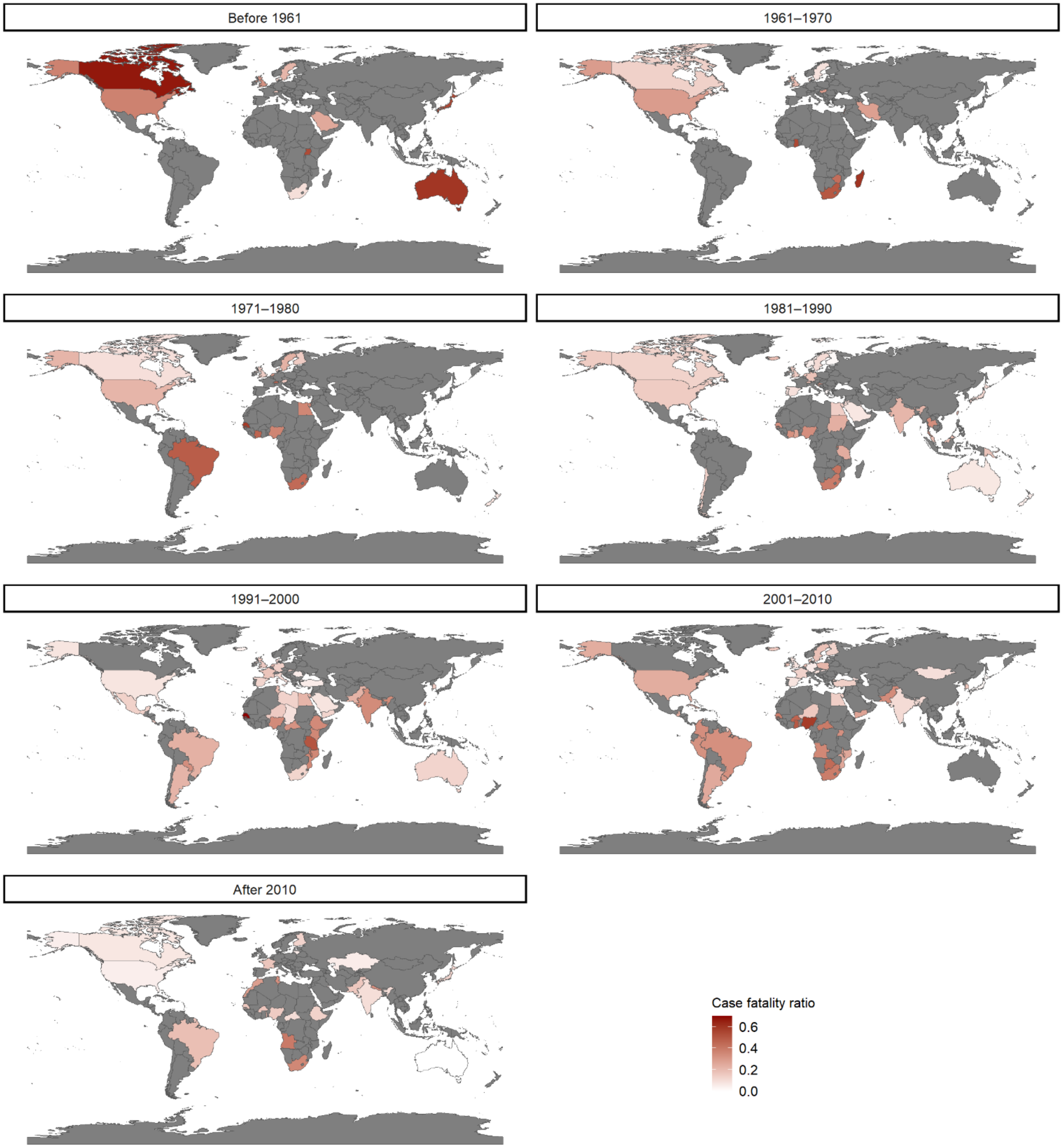

Meningococcal meningitis

eFigure 27. Geographic distribution of studies evaluating case fatality ratios in meningococcal meningitis and included into the meta–analysis.

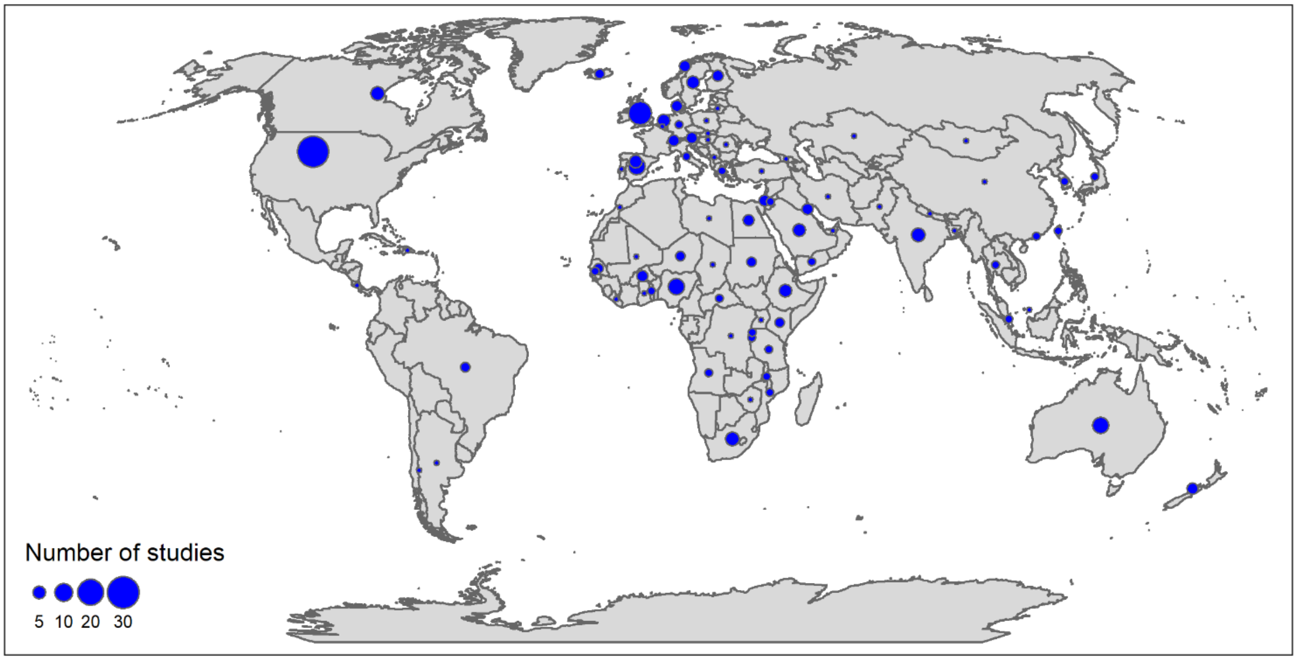

eFigure 28. Case fatality ratios in *N. meningitidis* meningitis (Forest plot with individual studies suppressed) indicating the overall pooled estimate and the estimates of the intervals as subgroups‡‡‡.

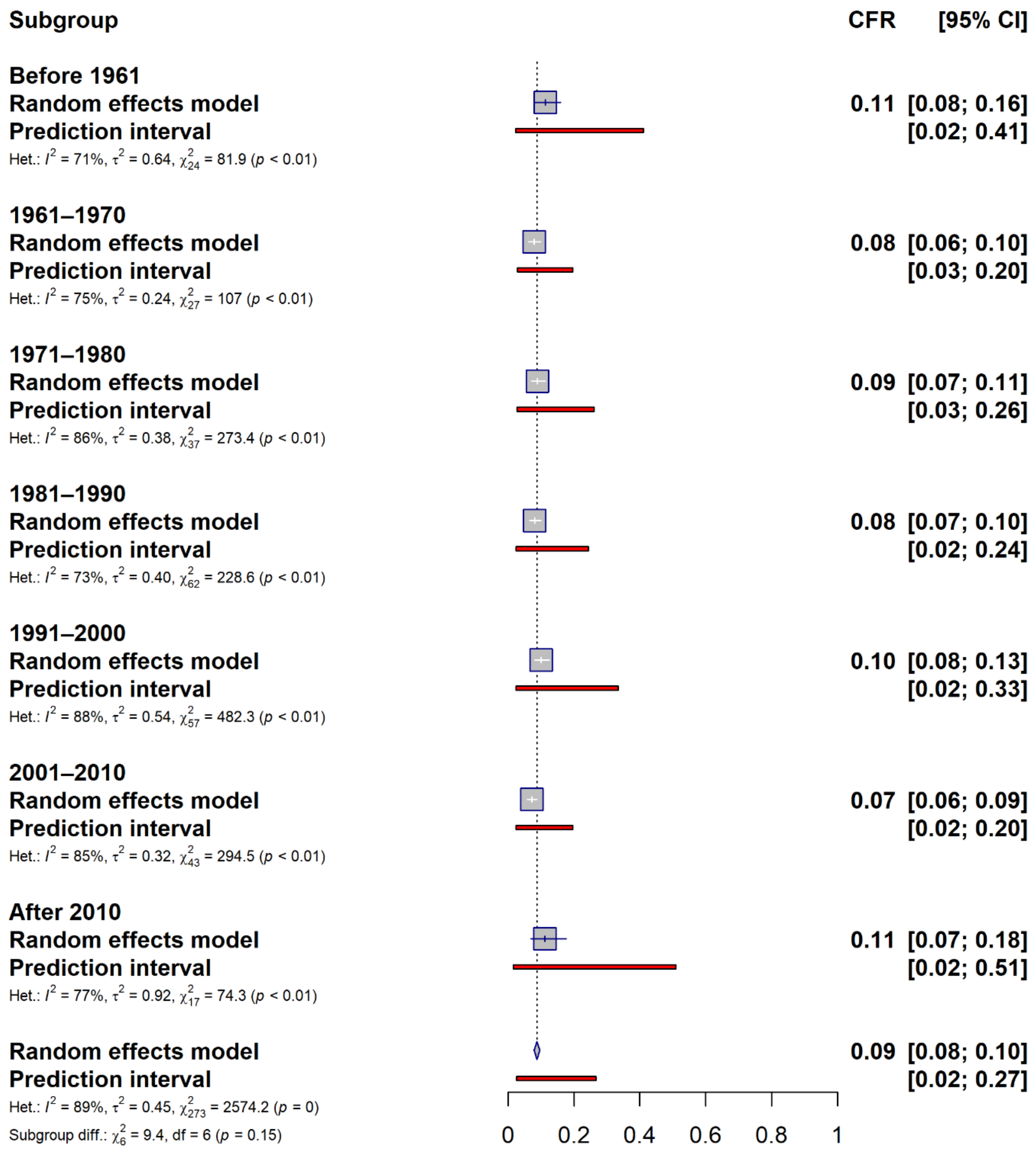

‡‡‡ Before 1961, k = 25; 1961–1970, k = 28; 1971–1980, k = 38; 1981–1990, k = 63; 1991–2000, k = 58; 2001–2010, k = 44; after 2010, k = 18; Het., between–study heterogeneity

eFigure 29. Case fatality ratios of patients with meningococcal meningitis using a meta–regression model with the studies' mean observation year as predictor variable (P = 0.081). (dashed lines, 95% CI; dotted lines, prediction interval)

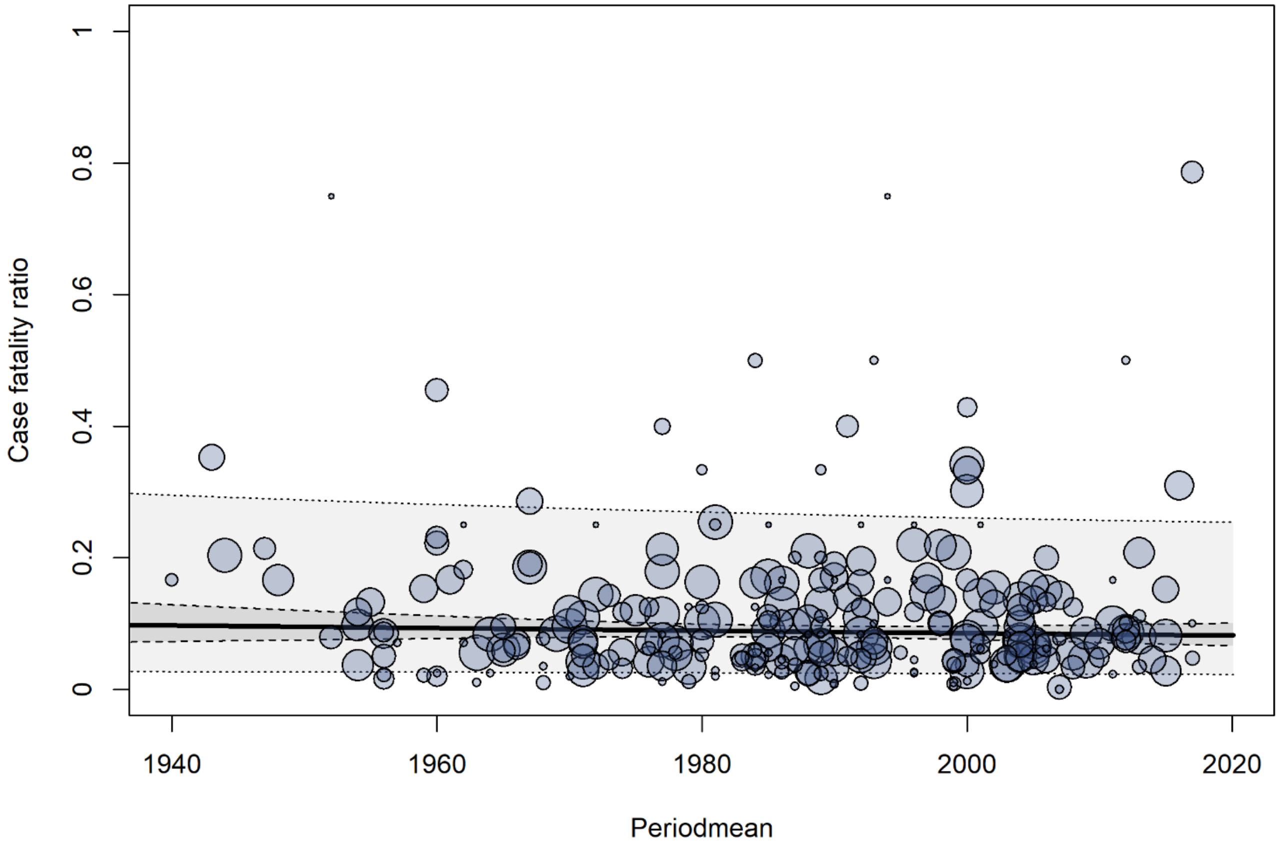

eFigure 30. Case fatality ratios of patients with meningococcal meningitis using a meta–regression model with the studies' mean observation year as predictor variable, stratified according to the age group (children, 2 months to 16 years; adults) and the Human Development Index (high-income countries, low-income countries). (dashed lines, 95% CI; dotted lines, prediction interval)

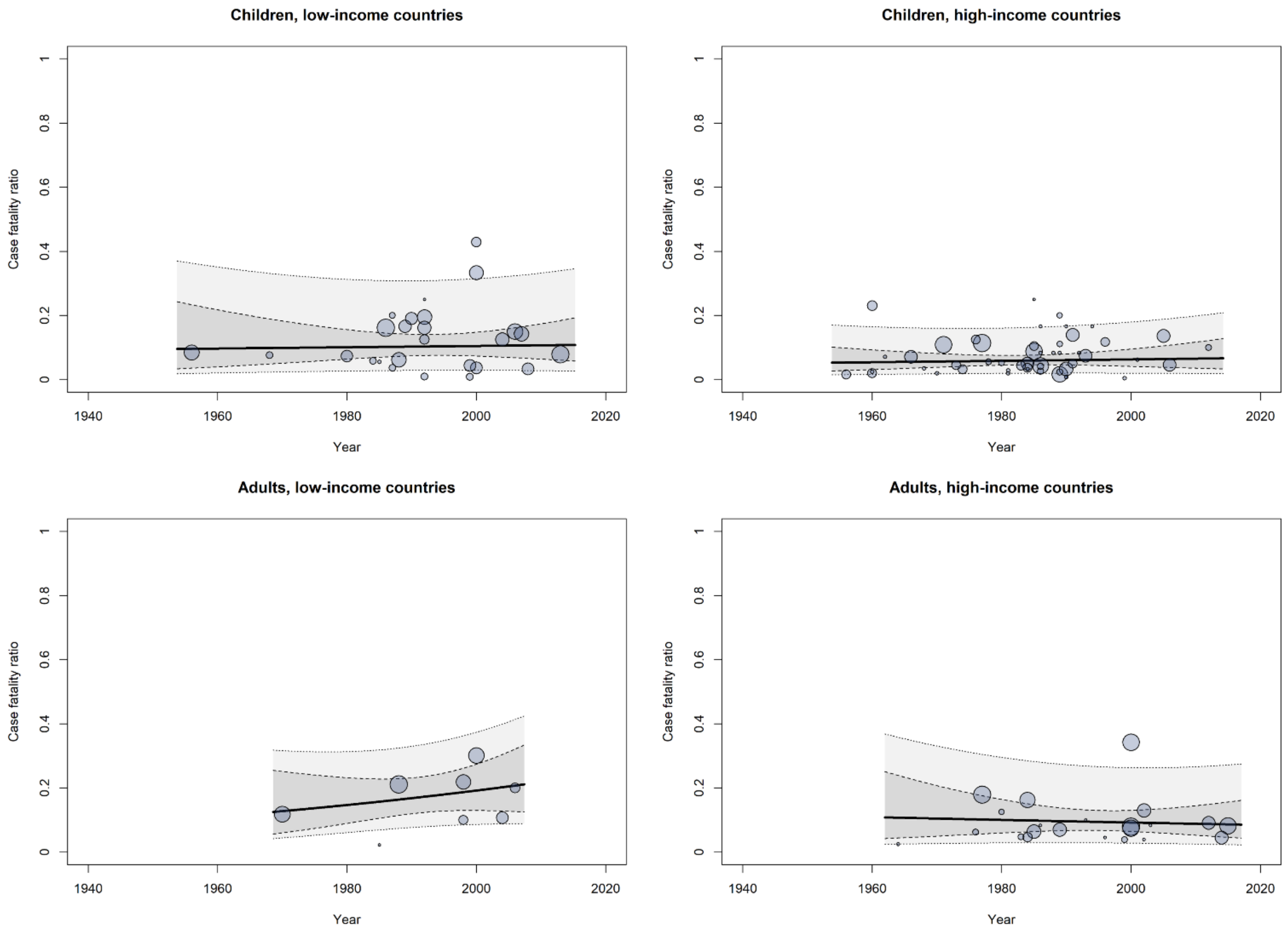

eFigure 31. Geographic distribution of studies evaluating case fatality ratios in meningococcal meningitis and included into the meta–analysis.

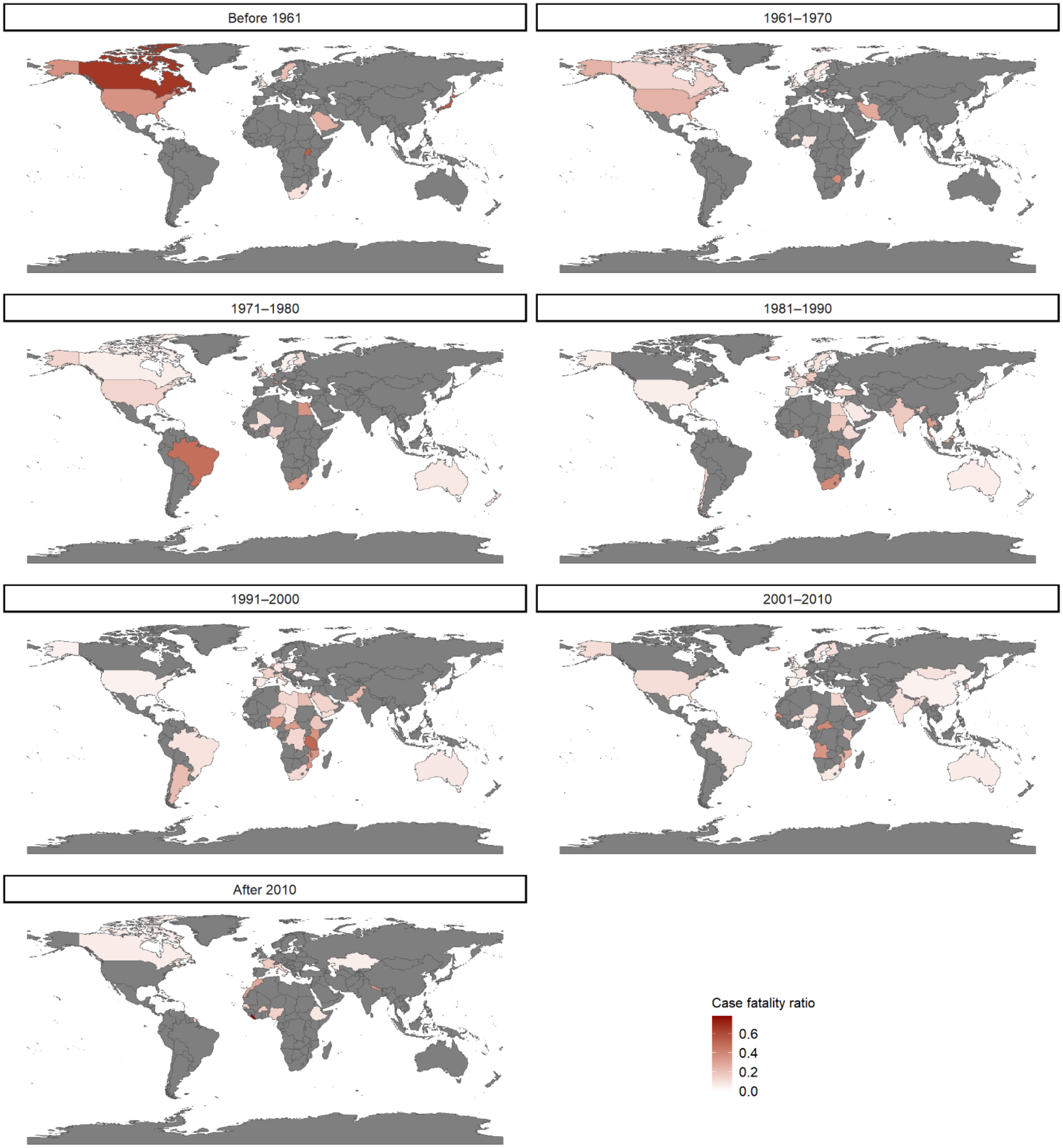

***H. influenzae* meningitis**

eFigure 32. Geographic distribution of studies evaluating case fatality ratios in *H. influenzae* meningitis and included into the meta–analysis.

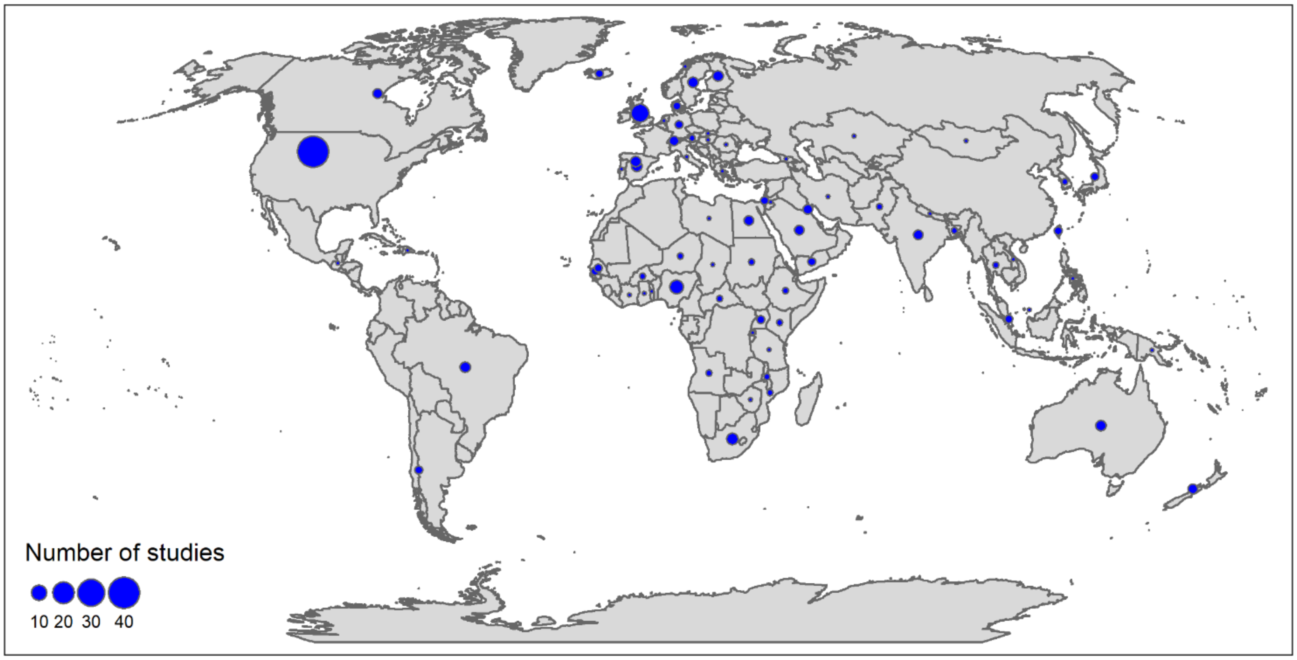

eFigure 33. Case fatality ratios in *H. influenzae* meningitis (Forest plot with individual studies suppressed) indicating the overall pooled estimate and the estimates of the intervals as subgroups.[§§§]

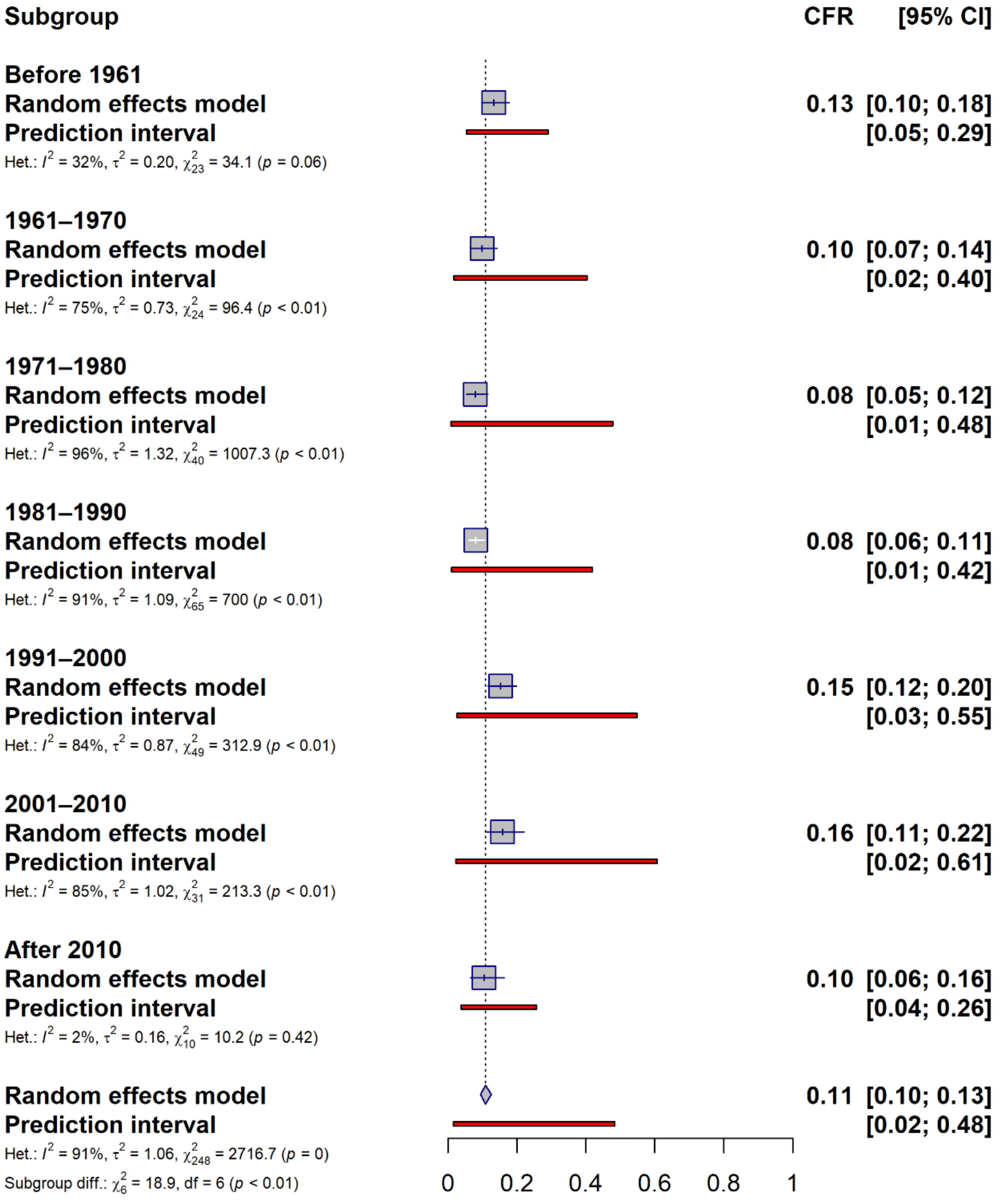

[§§§] Before 1961, k = 24; 1961–1970, k = 25; 1971–1980, k = 41; 1981–1990, k = 66; 1991–2000, k = 50; 2001–2010, k = 32; after 2010, k = 11); Het., between–study heterogeneity

eFigure 34. Case fatality ratios of patients with *H. influenzae* meningitis using a meta–regression model with the studies' mean observation year as predictor variable (*P* = 0.754). (dashed lines, 95% CI; dotted lines, prediction interval)

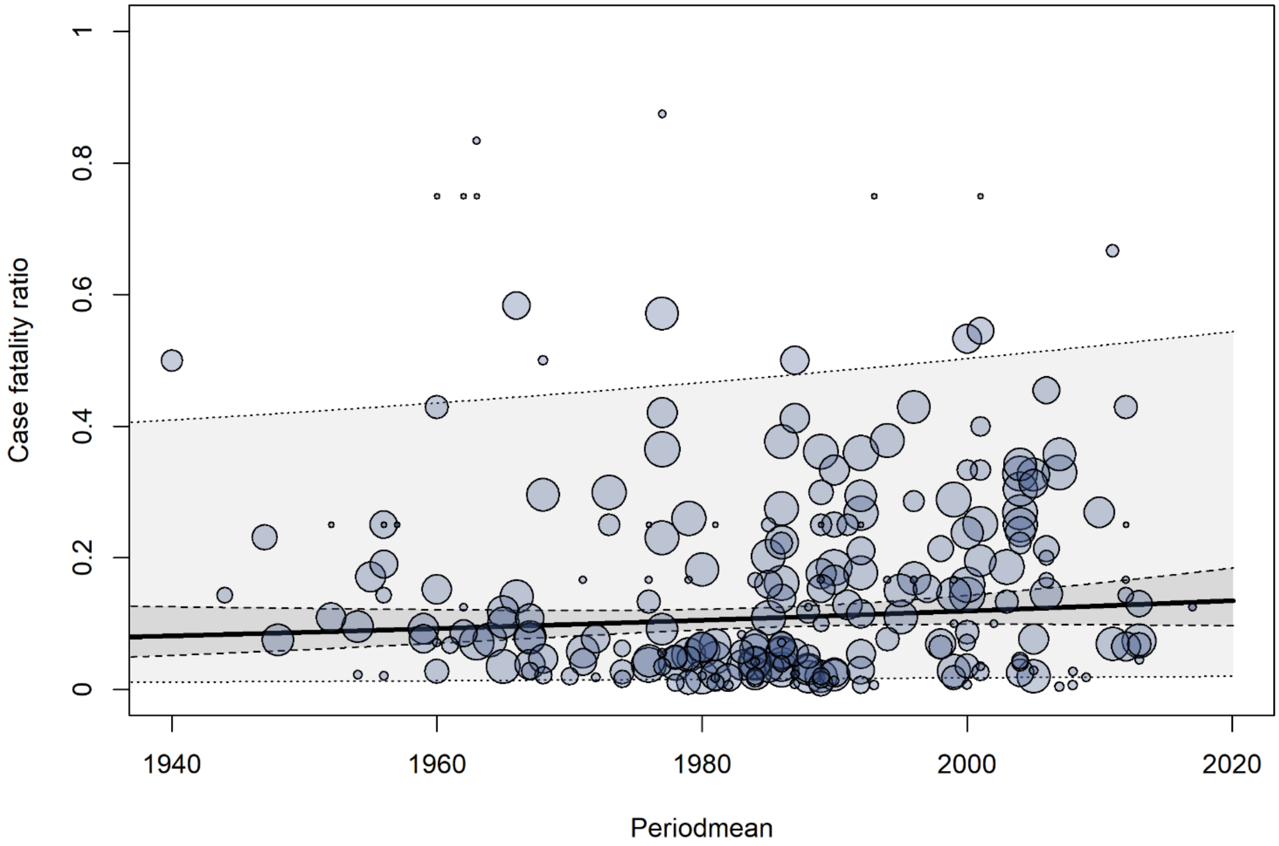

eFigure 35. Case fatality ratios of patients with *H. influenzae* meningitis using a meta–regression model with the studies' mean observation year as predictor variable, stratified according to the age group (children, 2 months to 16 years) and the Human Development Index (high-income countries, low-income countries). (dashed lines, 95% CI; dotted lines, prediction interval)

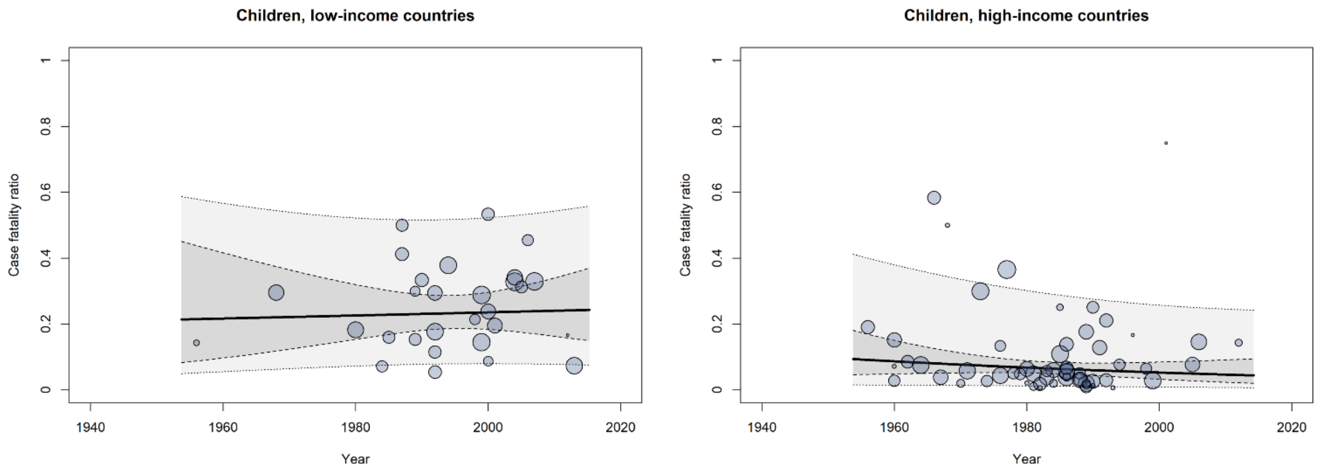

eFigure 36. Geographic distribution of studies evaluating case fatality ratios in *H. influenzae* meningitis and included into the meta–analysis.

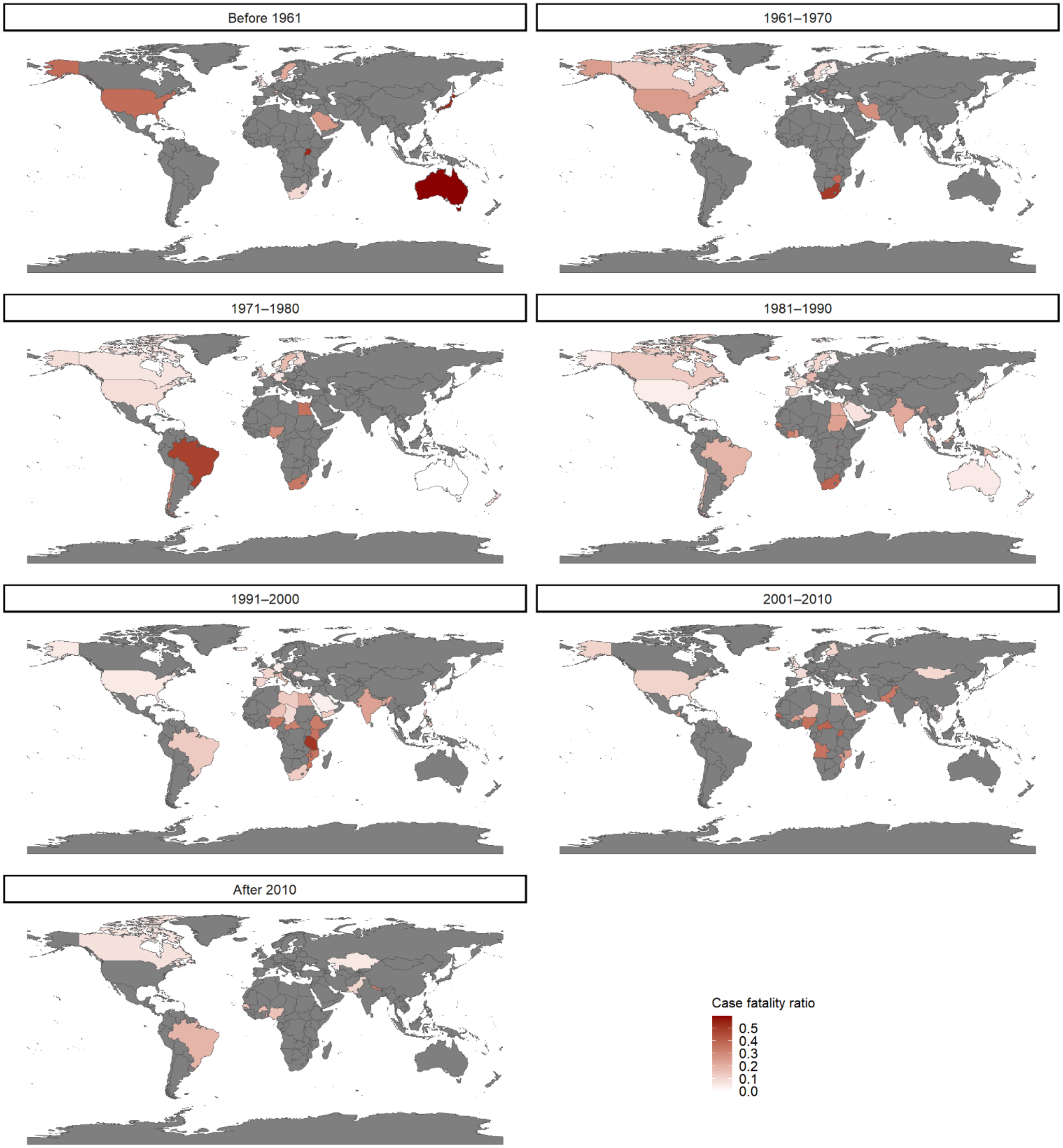

***L. monocytogenes* meningitis**

eFigure 37. Geographic distribution of studies evaluating case fatality ratios in Hib meningitis and included into the meta–analysis.

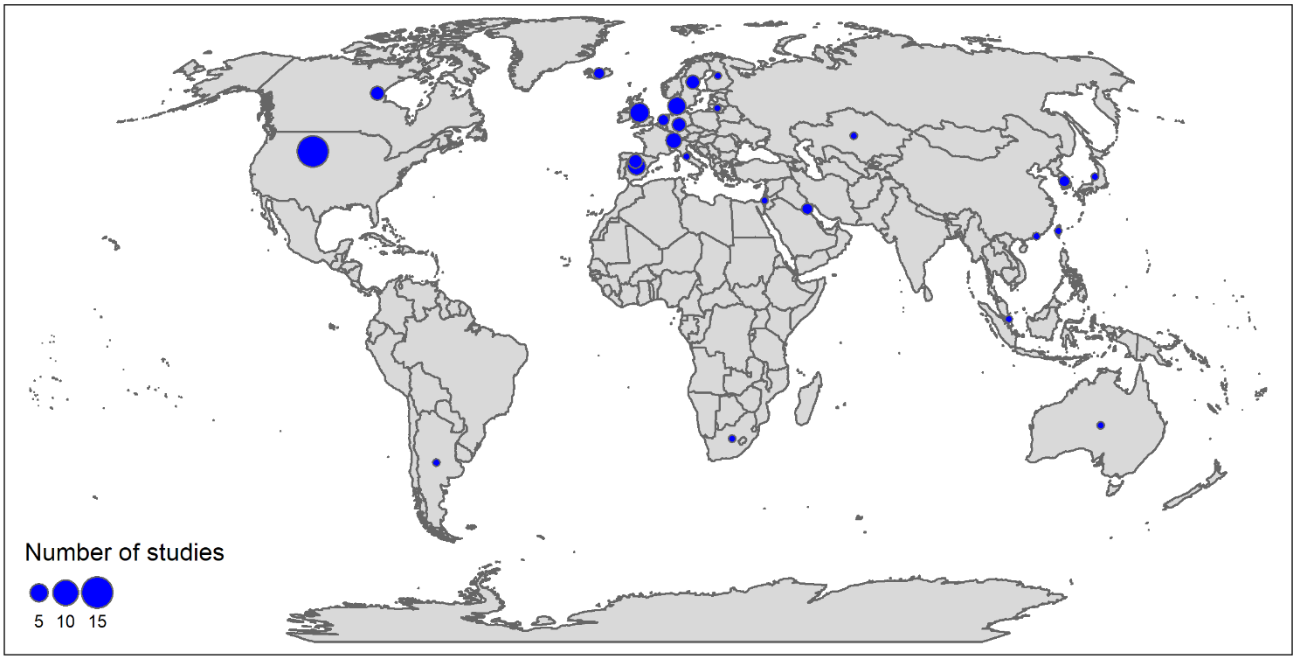

eFigure 38. Case fatality ratios in *L. monocytogenes* meningitis (Forest plot with individual studies suppressed) indicating the overall pooled estimate and the estimates of the intervals as subgroups.****

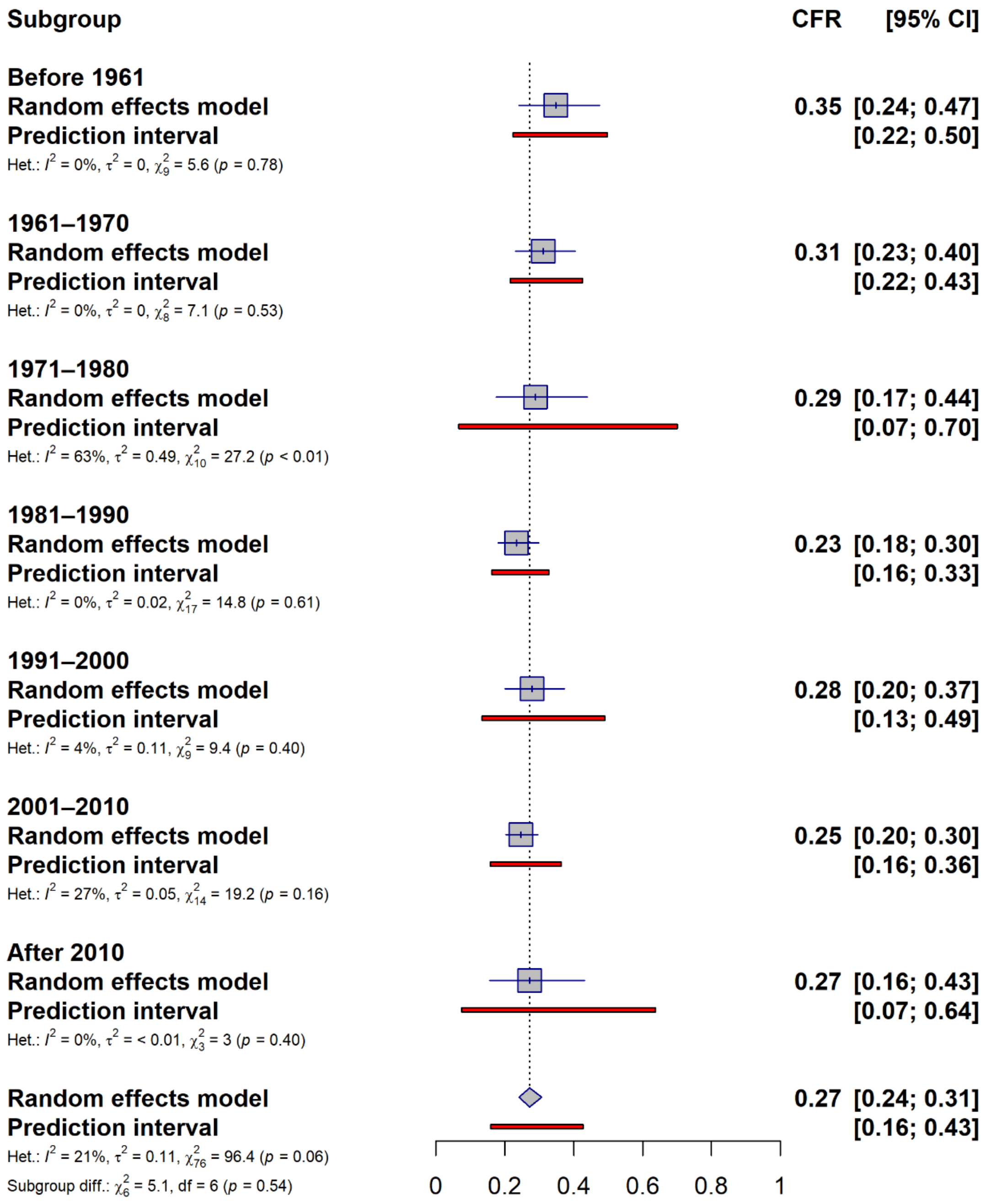

eFigure 39. Case fatality ratios of patients with *L. monocytogenes* meningitis using a meta–regression model with the studies' mean observation year as predictor variable (*P* = 0.082). (dashed lines, 95% CI; dotted lines, prediction interval)

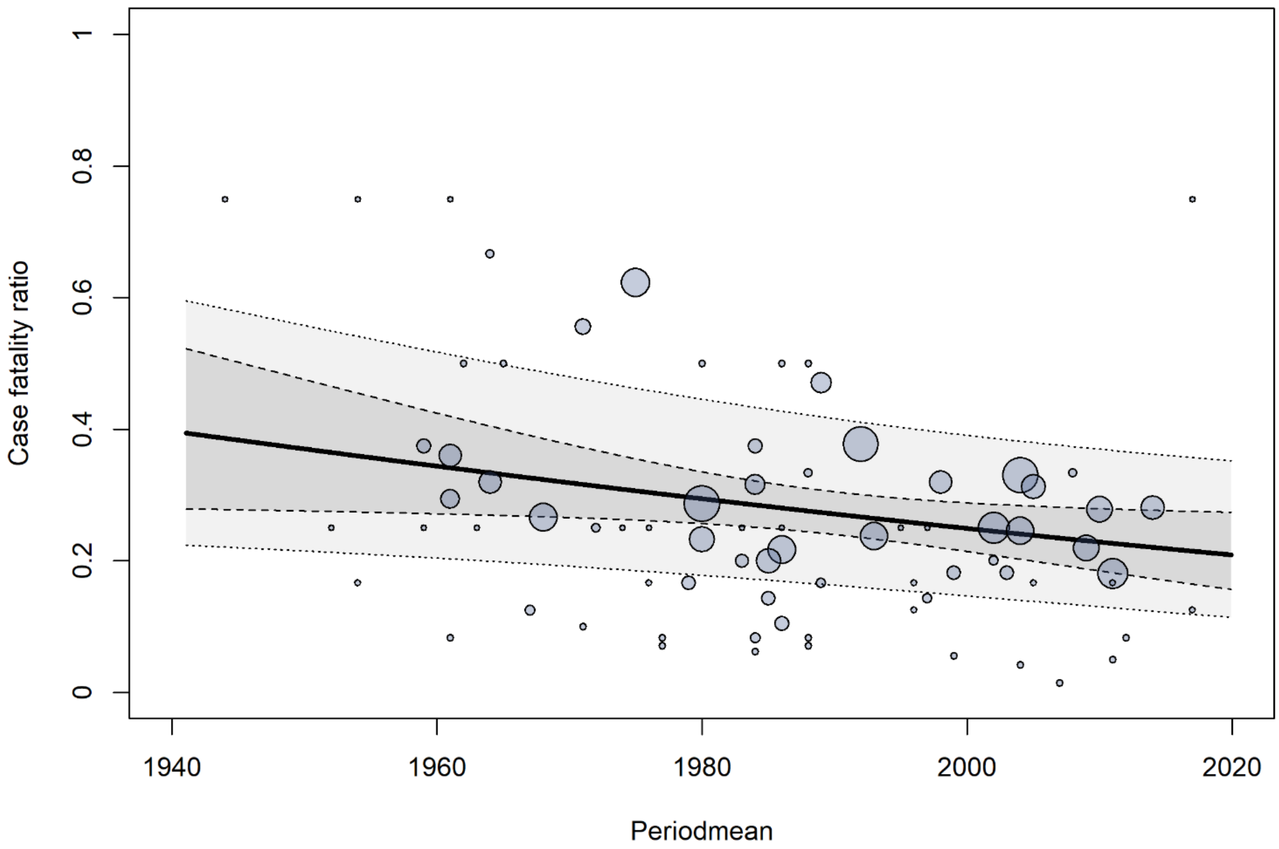

**** Before 1961, k = 10; 1961–1970, k = 9; 1971–1980, k = 11; 1981–1990, k = 18; 1991–2000, k = 10; 2001–2010, k = 15; after 2010, k = 4; Het., between–study heterogeneity

eFigure 40. Case fatality ratios of adult patients in high-income countries with *L. monocytogenes* meningitis using meta–regression model with the studies' mean observation year as predictor variable. (dashed lines, 95% CI; dotted lines, prediction interval)

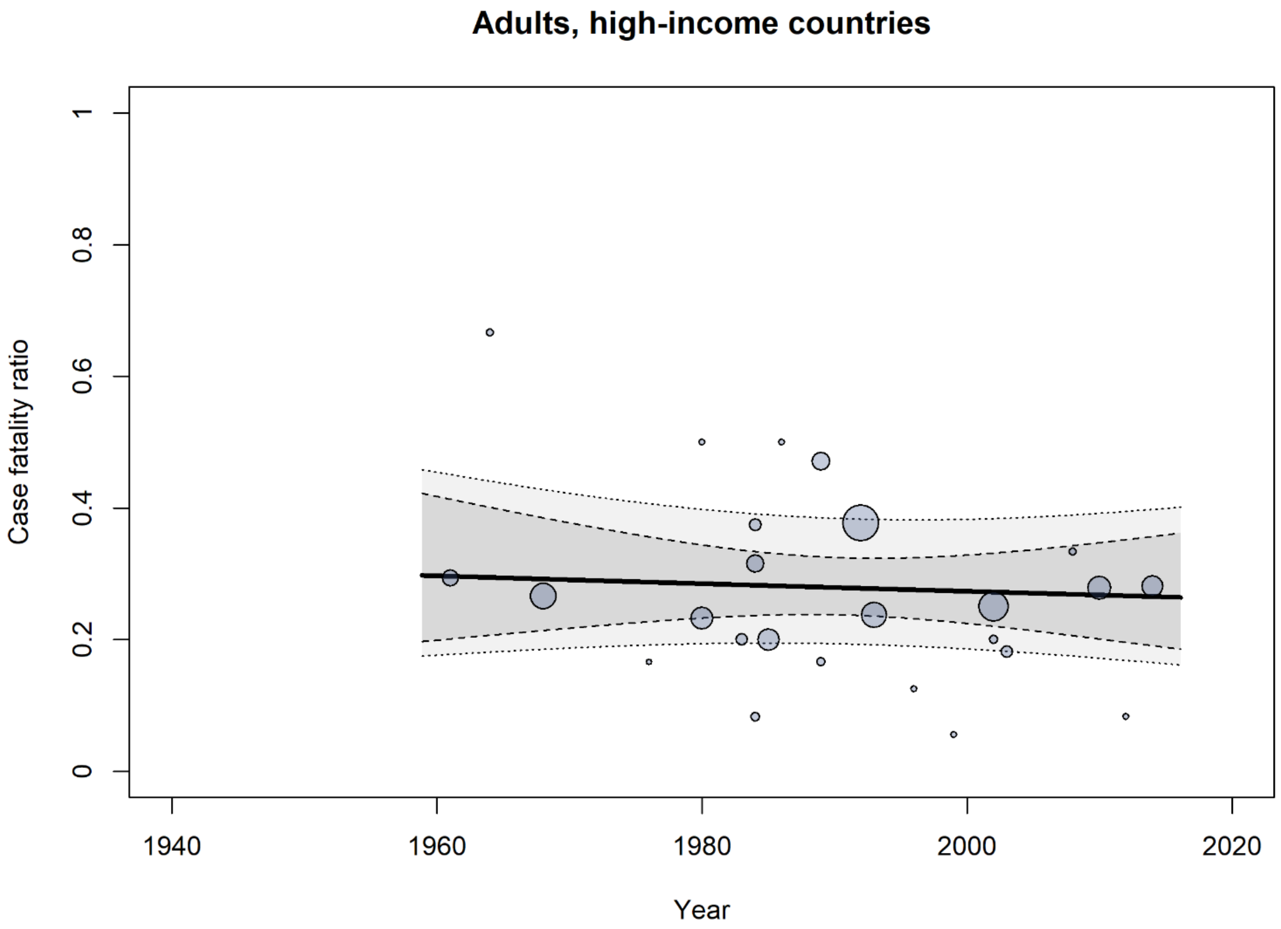

eFigure 41. Geographic distribution of studies evaluating case fatality ratios in *L. monocytogenes* meningitis and included into the meta–analysis.

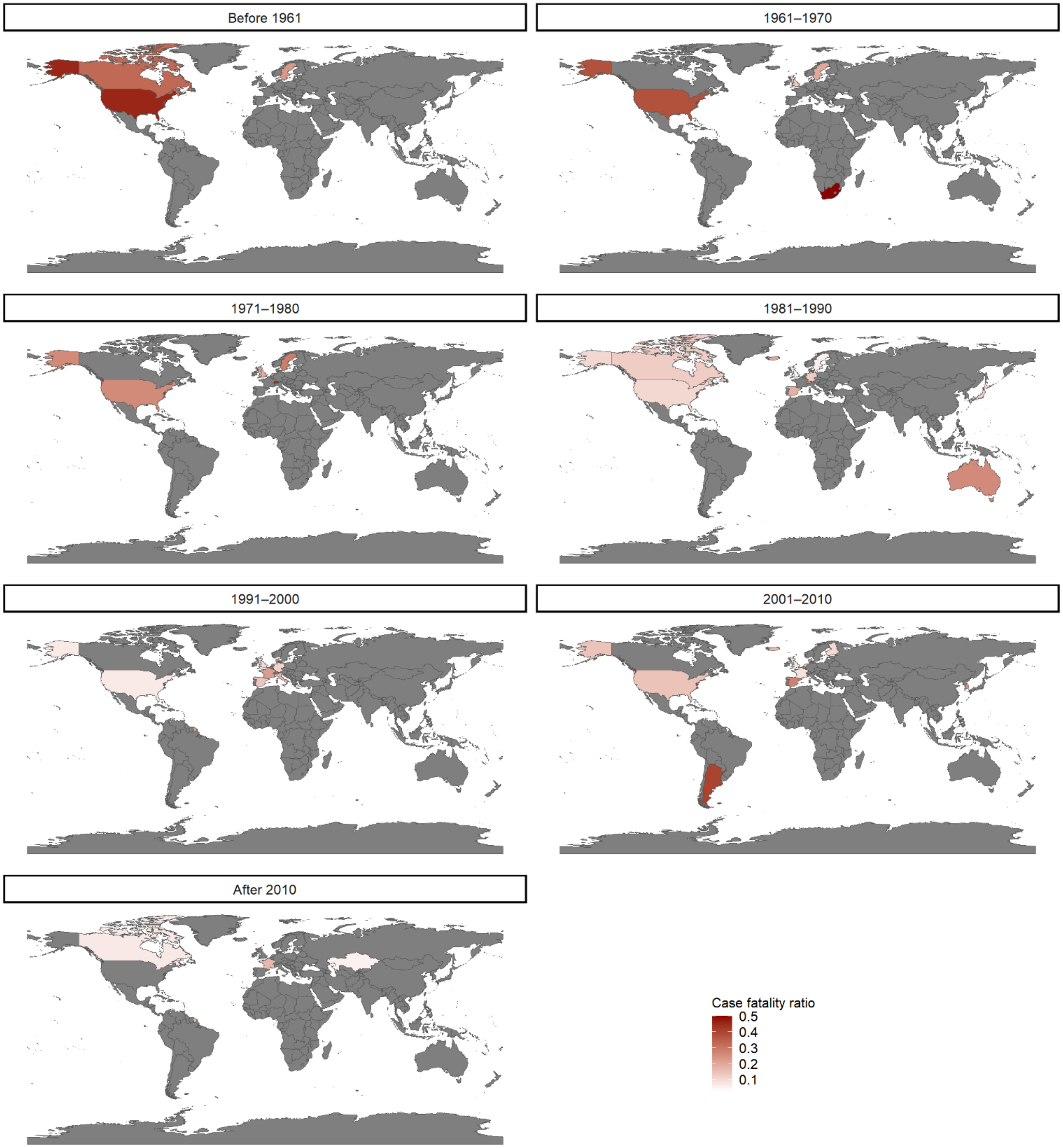

***E. coli* meningitis**

eFigure 42. Geographic distribution of studies evaluating case fatality ratios in *E.coli* meningitis and included into the meta–analysis.

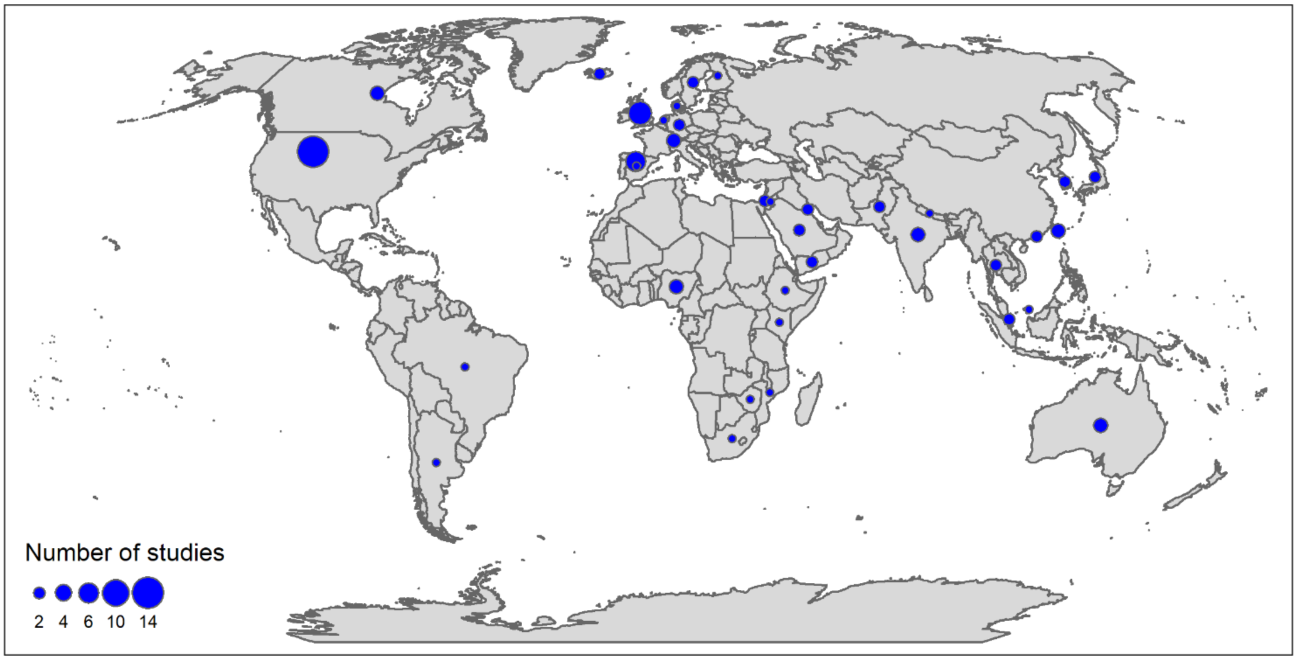

eFigure 43. Case fatality ratios in *E. coli* (Forest plot with individual studies suppressed) indicating the overall pooled estimate and the estimates of the intervals as subgroups.[††††]

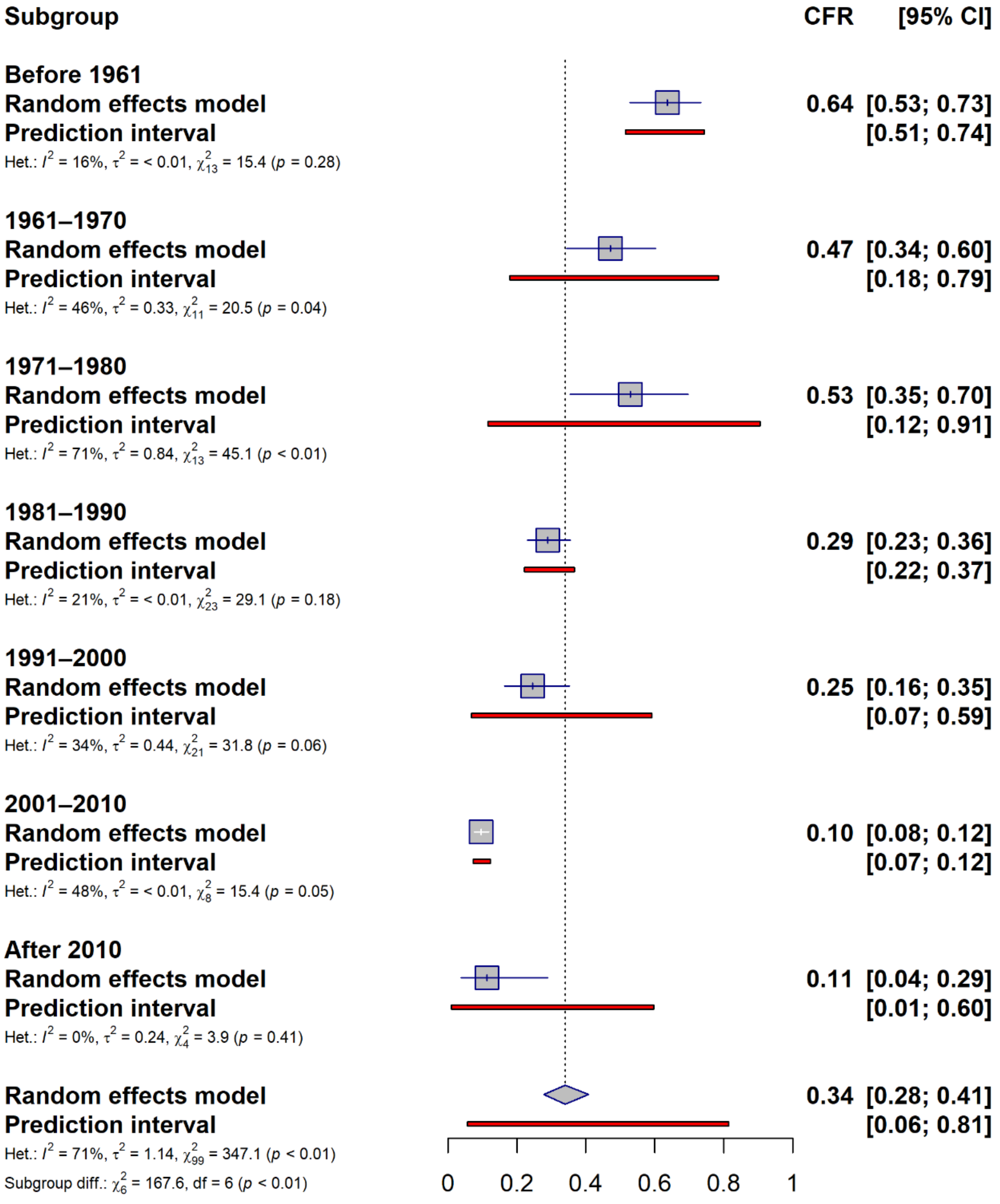

[††††] Before 1961, k = 14; 1961–1970, k = 12; 1971–1980, k = 14; 1981–1990, k = 24; 1991–2000, k = 22; 2001–2010, k = 9; after 2010, k = 5; Het., between–study heterogeneity

eFigure 44. Case fatality ratios of patients with *E. coli* meningitis using a meta–regression model with the studies' mean observation year as predictor variable ($P < 0.001$). (dashed lines, 95% CI; dotted lines, prediction interval)

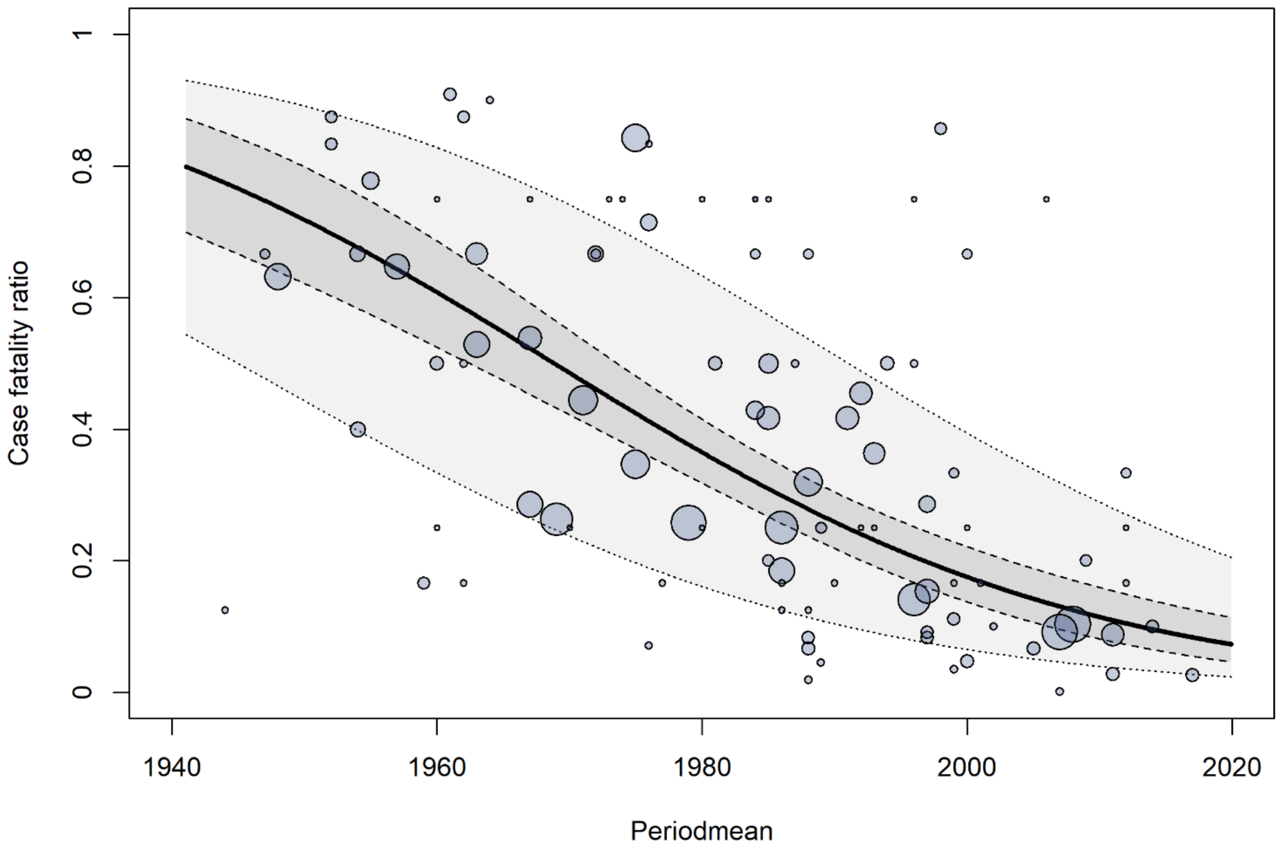

eFigure 45. Case fatality ratios of patients with *E. coli* meningitis using a meta–regression model with the studies' mean observation year as predictor variable, stratified according to the age group (neonates, 0 to 2 months) and the Human Development Index (high-income countries, low-income countries). (dashed lines, 95% CI; dotted lines, prediction interval)

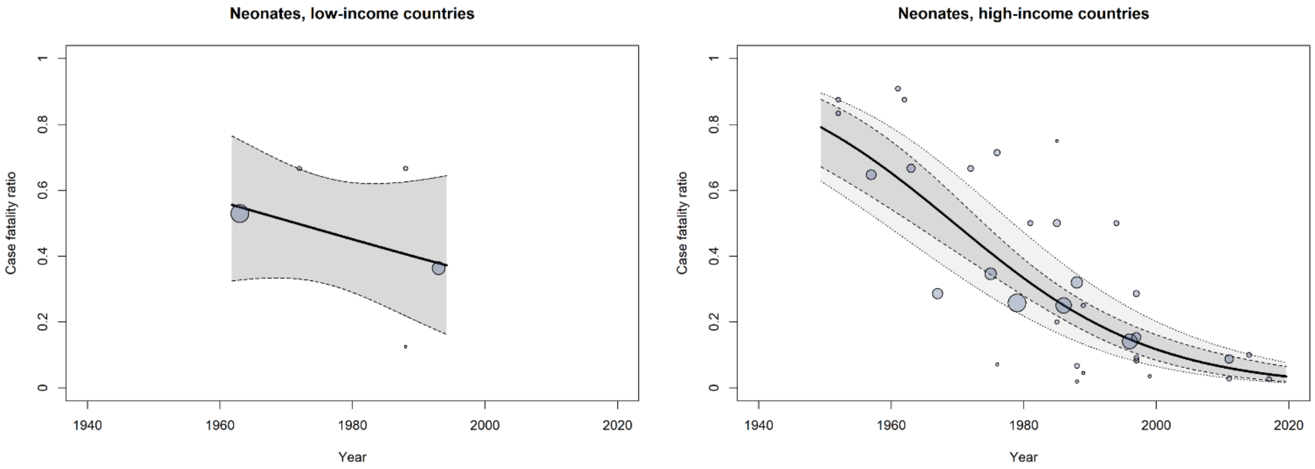

eFigure 46. Geographic distribution of studies evaluating case fatality ratios in *E. coli* meningitis and included into the meta–analysis.

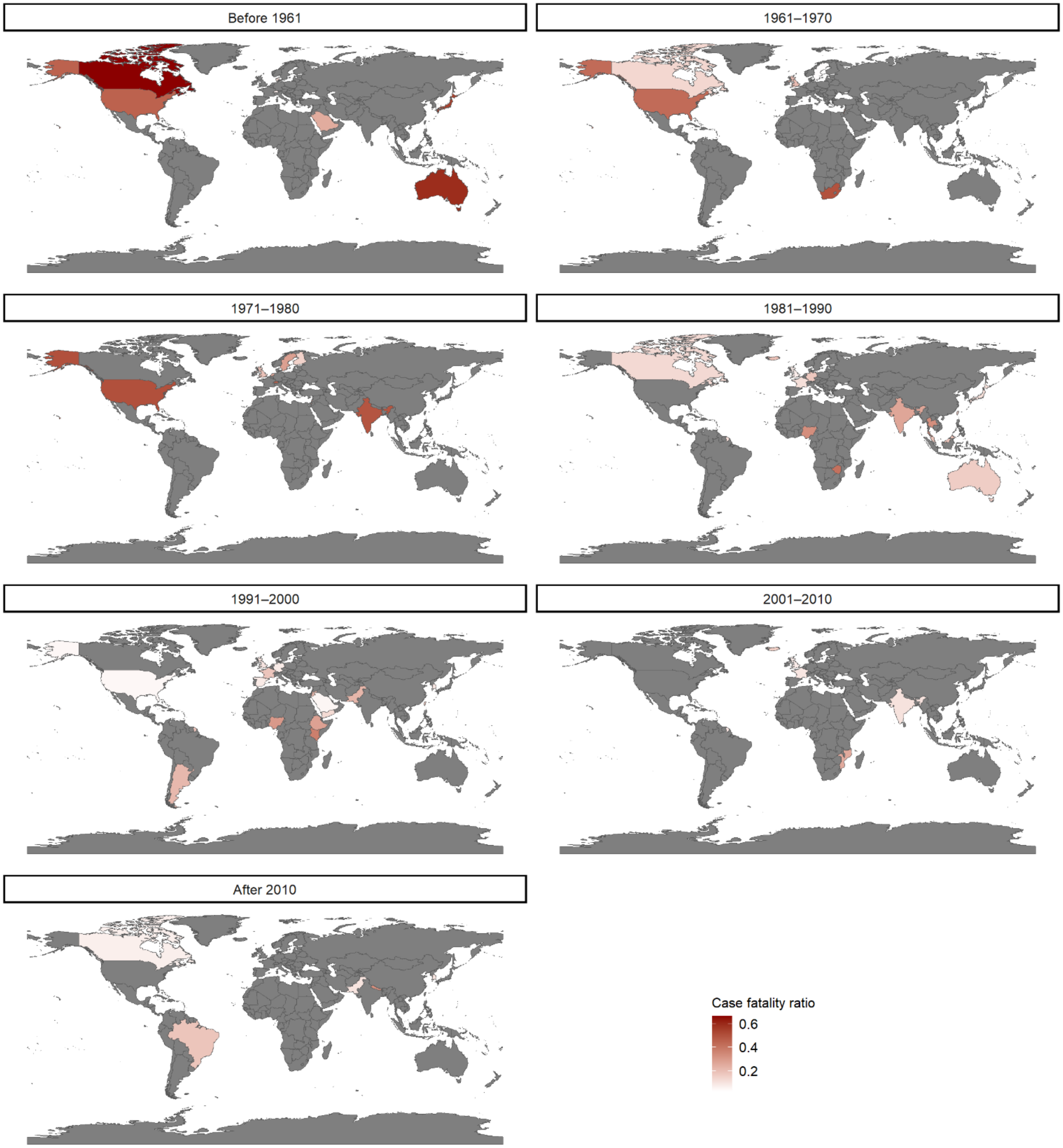

***S. agalactiae* meningitis**

eFigure 47. Geographic distribution of studies evaluating case fatality ratios in *S. agalactiae* meningitis and included into the meta–analysis.

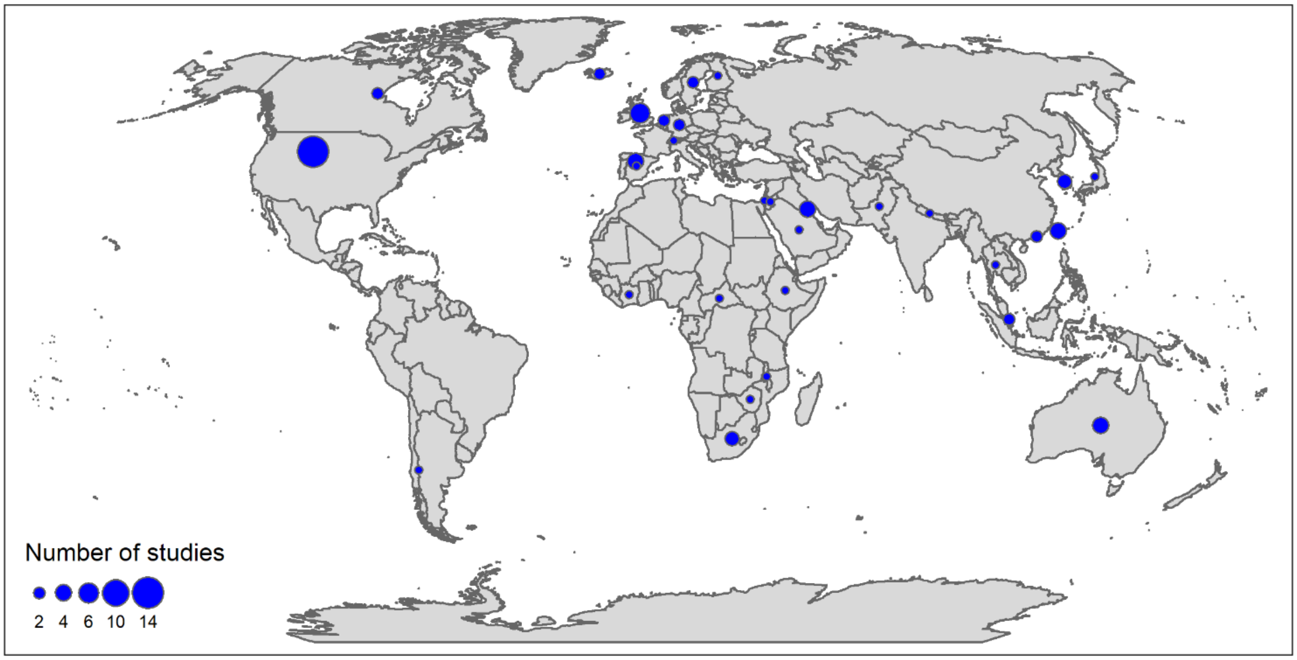

eFigure 48. Case fatality ratios in *S. agalactiae* meningitis (Forest plot with individual studies suppressed) indicating the overall pooled estimate and the estimates of the intervals as subgroups.‡‡‡‡

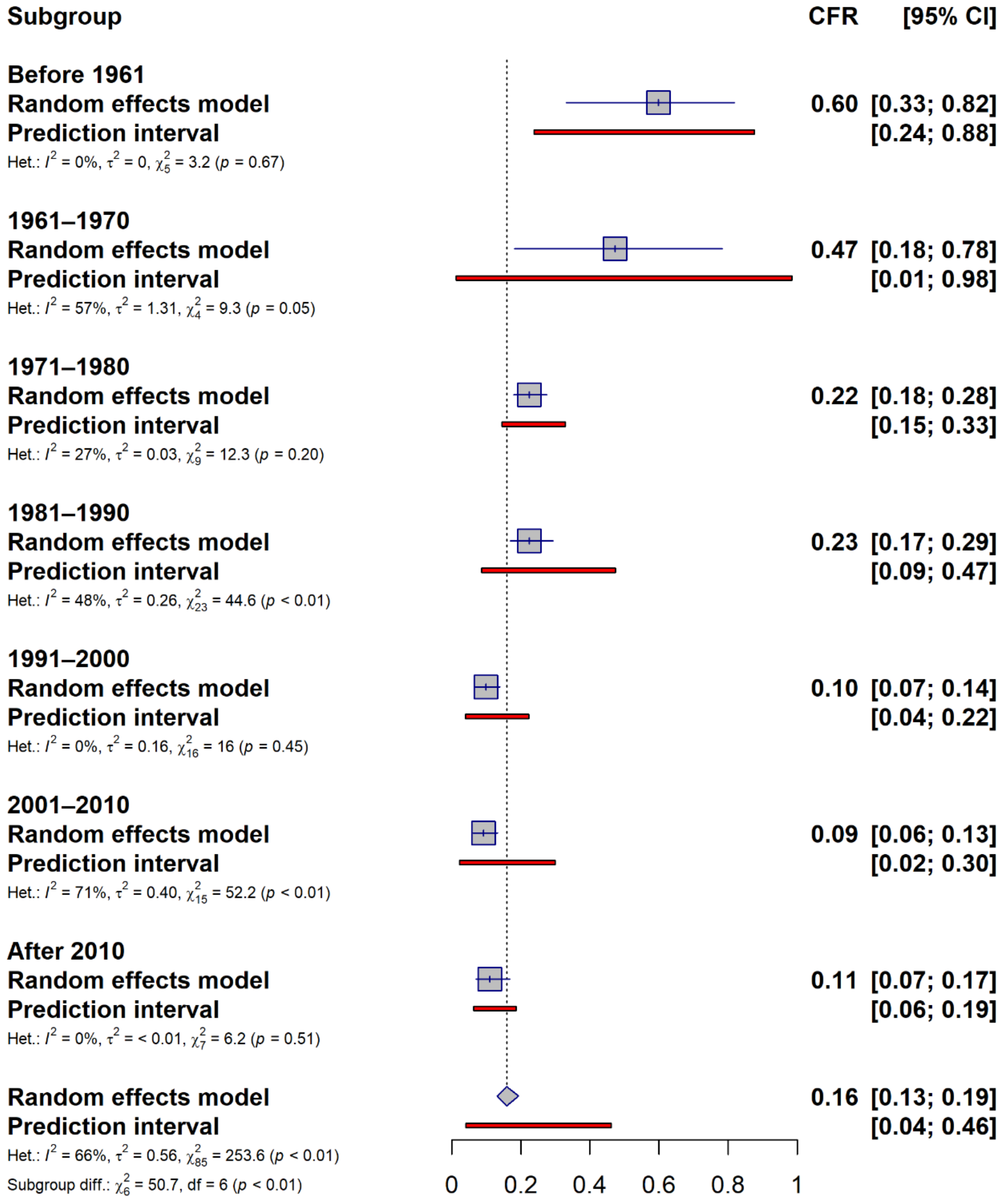

‡‡‡‡ Before 1961, k = 6; 1961–1970, k = 5; 1971–1980, k = 10; 1981–1990, k = 24; 1991–2000, k = 17; 2001–2010, k = 16; after 2010, k = 8; Het., between–study heterogeneity

eFigure 49. Case fatality ratios of patients with *S. agalactiae* meningitis using a meta–regression model with the studies' mean observation year as predictor variable ($P < 0.001$). (dashed lines, 95% CI; dotted lines, prediction interval)

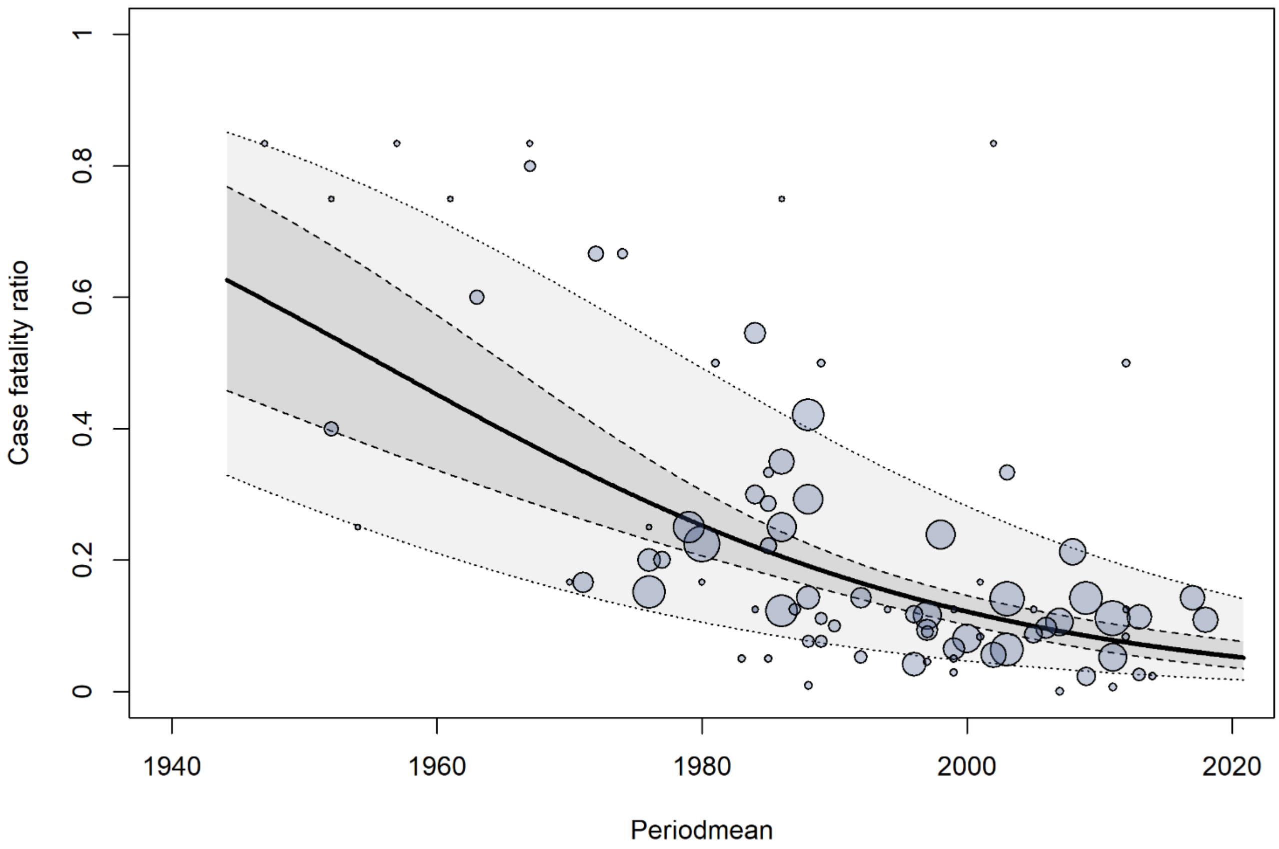

© 2024 van Ettekoven CN et al. *JAMA Network Open.*

eFigure 50. Case fatality ratios of patients with *S. agalactiae* meningitis using a meta–regression model with the studies' mean observation year as predictor variable, stratified according to the age group (neonates, 0 to 2 months) and the Human Development Index (high-income countries, low-income countries). (dashed lines, 95% CI; dotted lines, prediction interval)

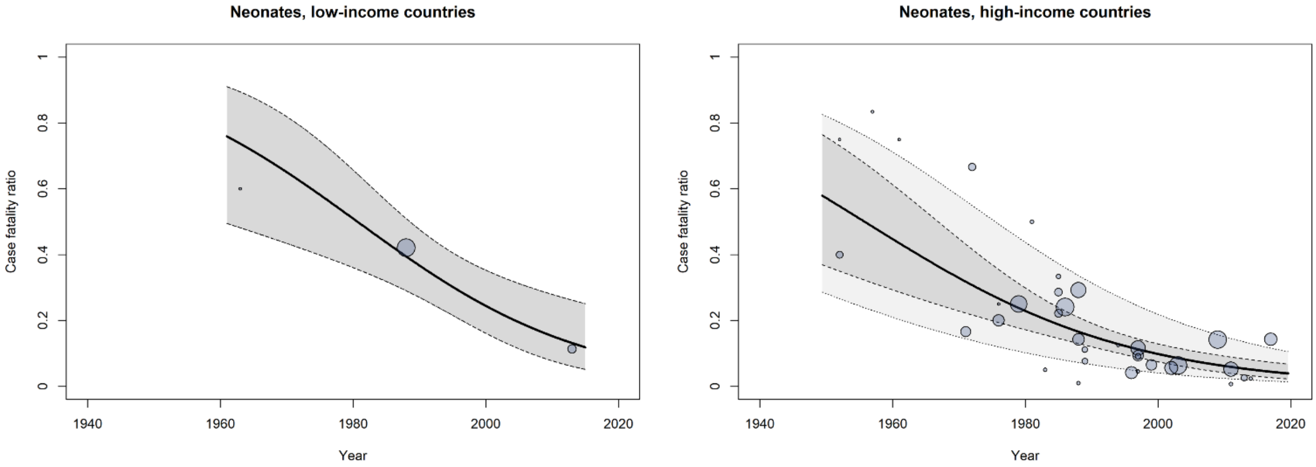

eFigure 51. Geographic distribution of studies evaluating case fatality ratios in *S. agalactiae* meningitis and included into the meta–analysis.

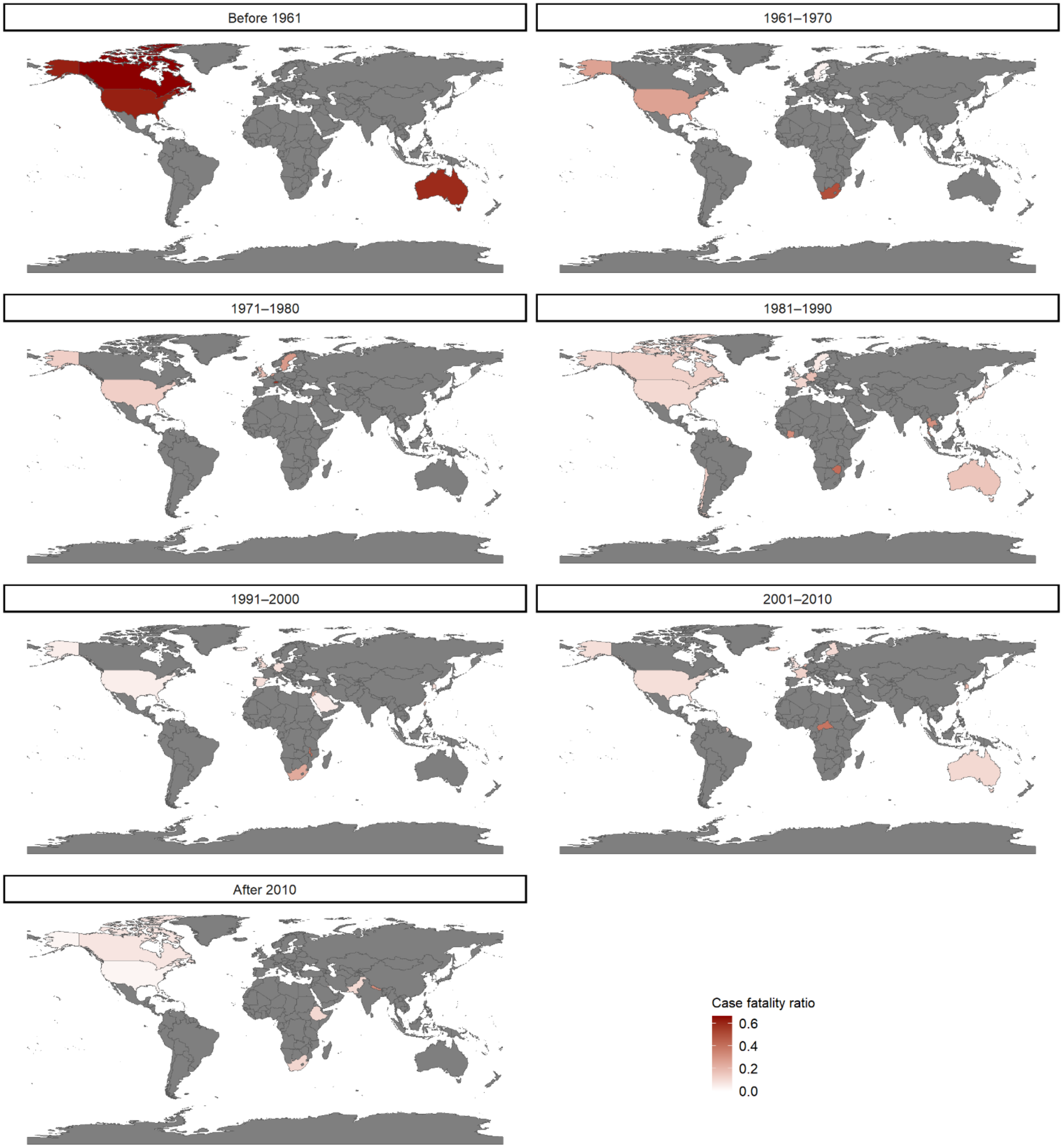

REFERENCES

1. Brainerd H, Bradley E. Treatment of Bacterial Meningitis with Penicillin, Sulfonamides, and Sera. *Calif Med*. Feb 1947;66(2):57-62.
2. Smith ES. Purulent meningitis in infants and children; a review of 409 cases. *J Pediatr*. Oct 1954;45(4):425-36. doi:10.1016/s0022-3476(54)80167-3
3. Watson DG. Purulent neonatal meningitis; a study of forty-five cases. *J Pediatr*. Mar 1957;50(3):352-60. doi:10.1016/s0022-3476(57)80035-3
4. Shaper AG, Shaper L. Analysis of medical admissions to Mulago Hospital, 1957. *East Afr Med J*. Dec 1958;35(12):647-78.
5. Eigler JO, Wellman WE, Rooke ED, Keith HM, Svien HJ. Bacterial meningits. I. General review (294 cases). *Proc Staff Meet Mayo Clin*. Jul 19 1961;36:357-65.
6. Esrachowitz SR. Pyogenic meningitis--a study of 303 cases. *S Afr Med J*. Feb 11 1961;35:101-4.
7. Groover RV, Sutherland JM, Landing BH. Purulent meningitis of newborn infants. Eleven-year experience in the antibiotic era. *N Engl J Med*. Jun 1 1961;264:1115-21. doi:10.1056/NEJM196106012642201
8. Carpenter RR, Petersdorf RG. The clinical spectrum of bacterial meningitis. *Am J Med*. Aug 1962;33:262-75. doi:10.1016/0002-9343(62)90024-4
9. Quaade F, Kristensen KP. Purulent meningitis. A review of 658 cases. *Acta Med Scand*. May 1962;171:543-50.
10. Yu JS, Grauaug A. Purulent Meningitis in the Neonatal Period. *Arch Dis Child*. Aug 1963;38(200):391-6. doi:10.1136/adc.38.200.391
11. Gossage JD. Acute Purulent Meningitis in Children: Experience at the Hospital for Sick Children, Toronto. *Can Med Assoc J*. Mar 7 1964;90(10):615-7.
12. Heycock JB, Noble TC. Pyogenic Meningitis in Infancy and Childhood. *Br Med J*. Mar 14 1964;1(5384):658-62. doi:10.1136/bmj.1.5384.658
13. Wilson FM, Lerner AM. Etiology and Mortality of Purulent Meningitis at the Detroit Receiving Hospital. *N Engl J Med*. Dec 10 1964;271:1235-8. doi:10.1056/NEJM196412102712403
14. Schmuziger P, Wegmann T. [Purulent Meningitis--Therapy and Prognosis]. *Schweiz Med Wochenschr*. Jan 30 1965;95:149-61. Die eitrige meningitis--therapie und prognose.
15. Swartz MN, Dodge PR. Bacterial Meningitis--a Review of Selected Aspects. 1. General Clinical Features, Special Problems and Unusual Meningeal Reactions Mimicking Bacterial Meningitis. *N Engl J Med*. Apr 8 1965;272:725-31 CONTD. doi:10.1056/NEJM196504082721406
16. Berman PH, Banker BQ. Neonatal meningitis. A clinical and pathological study of 29 cases. *Pediatrics*. Jul 1966;38(1):6-24.
17. Fortuine R. Acute purulent meningitis in Alaska natives: epidemiology, diagnosis and prognosis. *Can Med Assoc J*. Jan 1 1966;94(1):19-22.
18. McNiel JR. Acute bacterial meningitis as seen in children of Eastern Saudi Arabia. *Clin Pediatr (Phila)*. Jul 1966;5(7):437-8. doi:10.1177/000992286600500712
19. Donald G, McKendrick W. The treatment of pyogenic meningitis. *J Neurol Neurosurg Psychiatry*. Oct 1968;31(5):528-31. doi:10.1136/jnnp.31.5.528
20. Fosson AR, Fine RN. Neonatal meningitis. Presentation and discussion of 21 cases. *Clin Pediatr (Phila)*. Jul 1968;7(7):404-10. doi:10.1177/000992286800700712
21. Chevrie JJ, Aicardi J. Bacterial meningitis among newborn infants. *Clin Pediatr (Phila)*. Oct 1969;8(10):562-3. doi:10.1177/000992286900801002
22. Justitz B. [Purulent meningitis in childhood with special reference to fatal clinical cases and defective healing since the introduction of antibiotic treatment]. *Arch Kinderheilkd*. May 1970;181(1):40-62. Uber purulente Meningitiden im Kindesalter mit besonder Berucksichtigung von Todesfallen und Defektheilungen seit Einfuhrung der Antibiotika-Therapie.
23. Overall JC, Jr. Neonatal bacterial meningitis. Analysis of predisposing factors and outcome compared with matched control subjects. *J Pediatr*. Apr 1970;76(4):499-511. doi:10.1016/s0022-3476(70)80399-7
24. Seriki O. Pyogenic meningitis in infancy and childhood. A survey of this disease based on observations of 156 African patients with a high mortality rate. *Clin Pediatr (Phila)*. Jan 1970;9(1):17-21. doi:10.1177/000992287000900107
25. Jonsson M, Alvin A. A 12-year review of acute bacterial meningitis in Stockholm. *Scand J Infect Dis*. 1971;3(2):141-50. doi:10.3109/inf.1971.3.issue-2.08
26. Kendall AC. Acute bacterial meningitis in childhood. *Cent Afr J Med*. May 1971;17(5):98-101.
27. McDonald R. Purulent meningitis in newborn babies: observations and comments based on a series of 82 patients. *Clin Pediatr (Phila)*. Aug 1972;11(8):450-4. doi:10.1177/000992287201100808

28. Wiebe RA, Crast FW, Hall RA, Bass JW. Clinical factors relating to prognosis of bacterial meningitis. *South Med J*. Mar 1972;65(3):257-64. doi:10.1097/00007611-197203000-00001
29. Fraser DW, Henke CE, Feldman RA. Changing patterns of bacterial meningitis in Olmsted County, Minnesota, 1935-1970. *J Infect Dis*. Sep 1973;128(3):300-7. doi:10.1093/infdis/128.3.300
30. Fraser DW, Darby CP, Koehler RE, Jacobs CF, Feldman RA. Risk factors in bacterial meningitis: Charleston County, South Carolina. *J Infect Dis*. Mar 1973;127(3):271-7. doi:10.1093/infdis/127.3.271
31. Floyd RF, Federspiel CF, Schaffner W. Bacterial meningitis in urban and rural Tennessee. *Am J Epidemiol*. Jun 1974;99(6):395-407. doi:10.1093/oxfordjournals.aje.a121628
32. Fraser DW, Geil CC, Feldman RA. Bacterial meningitis in Bernalillo County, New Mexico: a comparison with three other American populations. *Am J Epidemiol*. Jul 1974;100(1):29-34. doi:10.1093/oxfordjournals.aje.a112005
33. Santhanakrishnan BR, Baliga R, Raju VB. Purulent meningitis in the new born. *Indian J Pediatr*. Jun 1974;41(317):218-23. doi:10.1007/BF02874900
34. Chintu C, Bathirunathan N. Bacterial meningitis in infancy and childhood in Lusaka (One year prospective sturdy). *Med J Zambia*. Dec-1976 Jan 1975;9(6):150-7.
35. Fraser DW, Mitchell JE, Silverman LP, Feldman RA. Undiagnosed bacterial meningitis in Vermont children. *Am J Epidemiol*. Nov 1975;102(5):394-9. doi:10.1093/oxfordjournals.aje.a112178
36. Hashemi C. Acute bacterial meningitis. Factors related to prognosis. *Indian J Pediatr*. Jul 1975;42(330):209-14. doi:10.1007/BF02752900
37. Hodges GR, Perkins RL. Acute bacterial meningitis: an analysis of factors influencing prognosis. *Am J Med Sci*. Nov-Dec 1975;270(3):427-40. doi:10.1097/00000441-197511000-00003
38. Kaiser E, Fulop T, Szabo K. Purulent meningitis in infancy and childhood. *Acta Paediatr Acad Sci Hung*. 1975;16(1):13-22.
39. Dawson KP, Hammond N. Bacterial meningitis: a review of 53 patients. *N Z Med J*. Nov 10 1976;84(575):351-3.
40. Goldacre MJ. Acute bacterial meningitis in childhood. Incidence and mortality in a defined population. *Lancet*. Jan 3 1976;1(7949):28-31. doi:10.1016/s0140-6736(76)92921-4
41. Yeung CY. Intrathecal antibiotic therapy for neonatal meningitis. *Arch Dis Child*. Sep 1976;51(9):686-90. doi:10.1136/adc.51.9.686
42. Agranat O, Melmed S, Altmann G, Bank H. Bacterial and fungal meningitis in adults: a 22-year survey in a large community hospital in Israel. *Isr J Med Sci*. Dec 1977;13(12):1151-62.
43. Finland M, Barnes MW. Acute bacterial meningitis at Boston City Hospital during 12 selected years, 1935-1972. *J Infect Dis*. Sep 1977;136(3):400-15. doi:10.1093/infdis/136.3.400
44. Gilsdorf JR. Bacterial meningitis in southwestern Alaska. *Am J Epidemiol*. Nov 1977;106(5):388-91. doi:10.1093/oxfordjournals.aje.a112480
45. Lang SD. Bacterial meningitis in children. *N Z Med J*. Dec 14 1977;86(601):511-4.
46. Moazami R, Raafat F, Aftandelians R, Arbabzadeh F, Erfani A. Acute bacterial meningitis in children (a retrospective survey of six and half years at a major pediatric center in Tehran, Iran). *Paediatr Indones*. Sep-Oct 1977;17(9-10):281-8.
47. Hailemeskel H, Tafari N. Bacterial meningitis in childhood in an African city. Factors influencing aetiology and outcome. *Acta Paediatr Scand*. Nov 1978;67(6):725-30. doi:10.1111/j.1651-2227.1978.tb16250.x
48. Bieler-Niederer E. [Bacterial meningitis in newborn infants. A retrospective study from a pediatric clinic 1967-1978]. *Helv Paediatr Acta*. 1979;34(6):563-76. Meningites purulentes du nouveau-ne. Etude retrospective dans un service de pediatrie, 1967-1978.
49. Chattopadhyay B. Mortality from meningitis in a district general hospital--a review of 67 cases. *Public Health*. Mar 1980;94(2):71-7. doi:10.1016/s0033-3506(80)80117-x
50. Geiseler PJ, Nelson KE, Levin S, Reddi KT, Moses VK. Community-acquired purulent meningitis: a review of 1,316 cases during the antibiotic era, 1954-1976. *Rev Infect Dis*. Sep-Oct 1980;2(5):725-45. Not in File. doi:10.1093/clinids/2.5.725
51. Horwitz SJ, Boxerbaum B, O'Bell J. Cerebral herniation in bacterial meningitis in childhood. *Ann Neurol*. Jun 1980;7(6):524-8. doi:10.1002/ana.410070605
52. Perez-Yarza EG, Ruiz Benito C, Zudaire J, Solorzano C, Perez Trallero E. [Infections of the central nervous system. A review of 295 cases (author's transl)]. *An Esp Pediatr*. May 1980;13(5):381-90. Infecciones del sistema nervioso central. Comentarios sobre 295 casos.
53. Shann F, Germer S. Treatment of bacterial meningitis in children without intravenous fluids. *Med J Aust*. May 30 1981;1(11):577-8. doi:10.5694/j.1326-5377.1981.tb135838.x
54. Wotton KA, Stiver HG, Hildes JA. Meningitis in the central Arctic: a 4-year experience. *Can Med Assoc J*. Apr 1 1981;124(7):887-90.

55. Davey PG, Cruikshank JK, McManus IC, Mahood B, Snow MH, Geddes AM. Bacterial meningitis--ten years experience. *J Hyg (Lond)*. Jun 1982;88(3):383-401. doi:10.1017/s002217240007025x
56. Guggenbichler JP. [Purulent meningitis in children. II. Treatment and prognosis]. *Padiatr Padol*. 1982;17(1):43-65. Die eitrige Meningitis im Kindesalter. II Behandlung und Prognose.
57. Helwig H. [Right and wrong ways to treat meningitis (author's transl)]. *Monatsschr Kinderheilkd*. May 1982;130(5):307-11. Wege und Irrwege der Meningitis-Therapie.
58. Onile BA, Montefiore DG, Alausa OK, Ashiru JO. Bacterial meningitis: the first documentation of an epidemic in southern Nigeria. *Trans R Soc Trop Med Hyg*. 1982;76(1):41-4. doi:10.1016/0035-9203(82)90014-1
59. Bohr V, Hansen B, Jessen O, et al. Eight hundred and seventy-five cases of bacterial meningitis. Part I of a three-part series: clinical data, prognosis, and the role of specialised hospital departments. *J Infect*. Jul 1983;7(1):21-30. Not in File. doi:10.1016/s0163-4453(83)90894-0
60. Guirguis N, Hafez K, El Kholy MA, Robbins JB, Gotschlich EC. Bacterial meningitis in Egypt: analysis of CSF isolates from hospital patients in Cairo, 1977-78. *Bull World Health Organ*. 1983;61(3):517-24.
61. Ispahani P. Bacterial meningitis in Nottingham. *J Hyg (Lond)*. Oct 1983;91(2):189-201. doi:10.1017/s0022172400060198
62. Gorse GJ, Thrupp LD, Nudleman KL, Wyle FA, Hawkins B, Cesario TC. Bacterial meningitis in the elderly. *Arch Intern Med*. Aug 1984;144(8):1603-7.
63. McCracken GH, Jr. Management of bacterial meningitis in infants and children. Current status and future prospects. *Am J Med*. May 15 1984;76(5A):215-23. doi:10.1016/0002-9343(84)90267-5
64. Mulder CJ, Zanen HC. A study of 280 cases of neonatal meningitis in The Netherlands. *J Infect*. Sep 1984;9(2):177-84. doi:10.1016/s0163-4453(84)91351-3
65. Mulla MI, Moosajee I, Rubidge CJ, Moosa A. Nutritional status of children with pyogenic meningitis. *J Trop Pediatr*. Dec 1984;30(6):303-6. doi:10.1093/tropej/30.6.303
66. Rodriguez WJ, Khan WN, Gold B, Feris J, Puig J, Sturla C. Ceftazidime in the treatment of meningitis in infants and children over one month of age. *Am J Med*. Aug 9 1985;79(2A):52-5. doi:10.1016/0002-9343(85)90261-x
67. Schlech WF, 3rd, Ward JI, Band JD, Hightower A, Fraser DW, Broome CV. Bacterial meningitis in the United States, 1978 through 1981. The National Bacterial Meningitis Surveillance Study. *JAMA*. Mar 22-29 1985;253(12):1749-54.
68. Shann F, Barker J, Poore P. Chloramphenicol alone versus chloramphenicol plus penicillin for bacterial meningitis in children. *Lancet*. Sep 28 1985;2(8457):681-4. doi:10.1016/s0140-6736(85)92927-7
69. Skoch MG, Walling AD. Meningitis: describing the community health problem. *American journal of public health*. 1985-1-1 1985;75(5):550-2. doi:doi:
70. Valmari P. Primary diagnosis in a life-threatening childhood infection. A nationwide study on bacterial meningitis. *Ann Clin Res*. 1985;17(6):310-5.
71. Benderly A, Shehadeh N, Grief Z, Hayek T, Erde P, Etzioni A. Bacterial meningitis in infants two to six weeks old. *Helv Paediatr Acta*. Oct 1986;41(4):311-5.
72. Jadavji T, Biggar WD, Gold R, Prober CG. Sequelae of acute bacterial meningitis in children treated for seven days. *Pediatrics*. Jul 1986;78(1):21-5.
73. Rantakallio P, Leskinen M, von Wendt L. Incidence and prognosis of central nervous system infections in a birth cohort of 12,000 children. *Scand J Infect Dis*. 1986;18(4):287-94. doi:10.3109/00365548609032339
74. Yost GC, Kaplan AM, Bustamante R, Ellison C, Hargrave AF, Randall DL. Bacterial meningitis in Arizona American Indian children. *American journal of diseases of children (1960)*. 1986-1-1 1986;140(9):943-6. doi:doi:
75. Bennhagen R, Svenningsen NW, Bekassy AN. Changing pattern of neonatal meningitis in Sweden. A comparative study 1976 vs. 1983. *Scand J Infect Dis*. 1987;19(6):587-93. doi:10.3109/00365548709117191
76. Girgis NI, Abu el Ella AH, Farid Z, Woody JN, Lissner C. Ceftriaxone compared with a combination of ampicillin and chloramphenicol in the treatment of bacterial meningitis in adults. *Drugs Exp Clin Res*. 1987;13(8):497-500.
77. Girgis NI, Abu el-Ella AH, Farid Z, Haberberger RL, Woody JN. Ceftriaxone alone compared to ampicillin and chloramphenicol in the treatment of bacterial meningitis. *Chemotherapy*. 1988;34 Suppl 1:16-20. doi:10.1159/000238642
78. Kilpatrick ME, Mikhail IA, Girgis NI. Negative cultures of cerebrospinal fluid in partially treated bacterial meningitis. *Trop Geogr Med*. Oct 1987;39(4):345-9.
79. Mir F, Aman S, Raza Khan S. Neonatal sepsis: a review with a study of 50 cases. *J Trop Pediatr*. Jun 1987;33(3):131-5. doi:10.1093/tropej/33.3.131
80. Salwen KM, Vikerfors T, Olcen P. Increased incidence of childhood bacterial meningitis. A 25-year study in a defined population in Sweden. *Scand J Infect Dis*. 1987;19(1):1-11. doi:10.3109/00365548709032371
81. Zimmerli W, Egli TF, Ritz R. [Prognostic factors in bacterial meningitis in adults. Retrospective analysis of 46 patients]. *Schweiz Med Wochenschr*. Jun 6 1987;117(23):861-7. Prognostische Faktoren bei der bakteriellen Meningitis des Erwachsenen. Eine retrospektive Analyse von 46 Patienten.

82. Dawson KP, Abbott GD, Mogridge N. Bacterial meningitis in childhood: a 13 year review. *N Z Med J*. Nov 9 1988;101(857):758-60.
83. Bell AH, Brown D, Halliday HL, McClure G, McReid M. Meningitis in the newborn--a 14 year review. *Arch Dis Child*. Jun 1989;64(6):873-4. doi:10.1136/adc.64.6.873
84. Bhat BV, Verma IC, Puri RK, Srinivasan S, Nalini P. A profile of pyogenic meningitis in children. *J Indian Med Assoc*. Aug 1991;89(8):224-7.
85. Cisse MF, Sow HD, Ouangre AR, Gaye A, Sow AI, Samb A, Fall M. [Bacterial meningitis in a pediatric hospital in a tropical zone]. *Med Trop (Mars)*. Jul-Sep 1989;49(3):265-9. Meningites bacteriennes dans un hopital pediatrique en zone tropicale.
86. Lim KW, Cheng HK. Bacterial meningitis--a four year survey in a paediatrics unit. *Ann Acad Med Singap*. Nov 1989;18(6):649-54.
87. Martinez-Martin P, Saenz Lope E, Estevez Guerra E, Rapun Pac JL. [Infectious meningitis in the adult: 3-year clinical experience]. *Neurologia*. Apr 1989;4(3):82-7. Meningitis infecciosas del adulto: experiencia clinica de tres anos.
88. Rosenthal J, Golan A, Dagan R. Bacterial meningitis with initial normal cerebrospinal fluid findings. *Isr J Med Sci*. Apr 1989;25(4):186-8.
89. Sakakihara Y, Kamoshita S. Changing spectrum of pediatric neurologic disorders during 18 selected years, 1900-1980, at the Hospital of University of Tokyo. *Brain Dev*. 1989;11(4):251-6. doi:10.1016/s0387-7604(89)80045-2
90. Shaltout AA, Auger LT, Awadallah NB, Hijazi Z, Johny M, Hajj KE, Kandil H. Morbidity and mortality of bacterial meningitis in Arab children. *J Trop Med Hyg*. Dec 1989;92(6):402-6.
91. Bryan JP, de Silva HR, Tavares A, Rocha H, Scheld WM. Etiology and mortality of bacterial meningitis in northeastern Brazil. *Rev Infect Dis*. Jan-Feb 1990;12(1):128-35. Not in File. doi:10.1093/clinids/12.1.128
92. Carter PE, Barclay SM, Galloway WH, Cole GF. Changes in bacterial meningitis. *Arch Dis Child*. May 1990;65(5):495-8. doi:10.1136/adc.65.5.495
93. Choo KE, Ariffin WA, Ahmad T, Lim WL, Gururaj AK. Pyogenic meningitis in hospitalized children in Kelantan, Malaysia. *Ann Trop Paediatr*. Mar 1990;10(1):89-98. doi:10.1080/02724936.1990.11747415
94. de Bary JB, Soro B, Seynaeve V, Schuermann L, Rey JL. [Purulent meningitis at a semi-rural hospital in the forest area of the Ivory Coast]. *Bull Soc Pathol Exot*. 1990;83(4):460-7. Les meningites purulentes dans un hopital semi-rural de la zone forestiere de Cote d'Ivoire.
95. Pomeroy SL, Holmes SJ, Dodge PR, Feigin RD. Seizures and other neurologic sequelae of bacterial meningitis in children. *N Engl J Med*. Dec 13 1990;323(24):1651-7. doi:10.1056/NEJM199012133232402
96. Salih MA. Childhood acute bacterial meningitis in the Sudan: an epidemiological, clinical and laboratory study. *Scand J Infect Dis Suppl*. 1990;66:1-103. doi:10.3109/inf.1989.21.suppl-66.01
97. Wenger JD, Hightower AW, Facklam RR, Gaventa S, Broome CV. Bacterial meningitis in the United States, 1986: report of a multistate surveillance study. The Bacterial Meningitis Study Group. *J Infect Dis*. Dec 1990;162(6):1316-23. Not in File. doi:10.1093/infdis/162.6.1316
98. Zaki M, Daoud AS, al Saleh Q, Abd al Rasool MM. Bacterial meningitis in the newborn: a Kuwaiti experience. *J Trop Pediatr*. Apr 1990;36(2):63-5. doi:10.1093/tropej/36.2.63
99. Zaki M, Daoud AS, ElSaleh Q, West PW. Childhood bacterial meningitis in Kuwait. *J Trop Med Hyg*. Feb 1990;93(1):7-11.
100. de Louvois J, Blackbourn J, Hurley R, Harvey D. Infantile meningitis in England and Wales: a two year study. *Arch Dis Child*. May 1991;66(5):603-7. doi:10.1136/adc.66.5.603
101. Dufour JF, Waldvogel F. [Meningitis in adults in Geneva. Review of 257 cases]. *Schweiz Med Wochenschr Suppl*. 1991;35:1-37. Les meningites de l'adulte a Geneve. Revue de 257 cas.
102. Hanna JN, Wild BE. Bacterial meningitis in children under five years of age in Western Australia. *Med J Aust*. Aug 5 1991;155(3):160-4. doi:10.5694/j.1326-5377.1991.tb142183.x
103. Kabra SK, Kumar P, Verma IC, et al. Bacterial meningitis in India: an IJP survey. *Indian J Pediatr*. Jul-Aug 1991;58(4):505-11. doi:10.1007/BF02750932
104. Minutillo C, Pemberton PJ, Cole M. Neonatal meningitis. *J Paediatr Child Health*. Jun 1991;27(3):191-2. doi:10.1111/j.1440-1754.1991.tb00386.x
105. Nathoo KJ, Pazvakavamba I, Chidede OS, Chirisa C. Neonatal meningitis in Harare, Zimbabwe: a 2-year review. *Ann Trop Paediatr*. 1991 1991;11(1):11-5. Not in File. doi:10.1080/02724936.1991.11747472
106. Olanrewaju DM, Olusanya O, Laditan AA. Acute bacterial meningitis in children. *West Afr J Med*. Jan-Mar 1991;10(1):405-11.
107. Pecoul B, Varaine F, Keita M, et al. Long-acting chloramphenicol versus intravenous ampicillin for treatment of bacterial meningitis. *Lancet*. Oct 5 1991;338(8771):862-6. doi:10.1016/0140-6736(91)91511-r

108. al-Jurayyan NA, al Mazyad AS, al-Nasser MN, al-Eissa YA, Abo-Bakr AM, Boohene AG, Familusi JB. Childhood bacterial meningitis in Al-Baha province, Saudi Arabia. *J Trop Med Hyg*. Jun 1992;95(3):180-5.
109. Craig JC, Abbott GD, Mogridge NB. Ceftriaxone for paediatric bacterial meningitis: a report of 62 children and a review of the literature. *N Z Med J*. Nov 11 1992;105(945):441-4.
110. Francis BM, Gilbert GL. Survey of neonatal meningitis in Australia: 1987-1989. *Med J Aust*. Feb 17 1992;156(4):240-3. doi:10.5694/j.1326-5377.1992.tb139741.x
111. Franco SM, Cornelius VE, Andrews BF. Long-term outcome of neonatal meningitis. *American journal of diseases of children (1960)*. 1992-1-1 1992;146(5):567-71. doi:doi:
112. Mackie EJ, Shears P, Frimpong E, Mustafa-Kutana SN. A study of bacterial meningitis in Kumasi, Ghana. *Ann Trop Paediatr*. 1992;12(2):143-8. doi:10.1080/02724936.1992.11747559
113. Pallangyo K, Hakanson A, Lema L, et al. High HIV seroprevalence and increased HIV-associated mortality among hospitalized patients with deep bacterial infections in Dar es Salaam, Tanzania. *AIDS*. Sep 1992;6(9):971-6. doi:10.1097/00002030-199209000-00010
114. Rasmussen HH, Sorensen HT, Moller-Petersen J, Mortensen FV, Nielsen B. Bacterial meningitis in elderly patients: clinical picture and course. *Age Ageing*. May 1992;21(3):216-20. doi:10.1093/ageing/21.3.216
115. Rothrock SG, Green SM, Wren J, Letai D, Daniel-Underwood L, Pillar E. Pediatric bacterial meningitis: is prior antibiotic therapy associated with an altered clinical presentation? *Ann Emerg Med*. Feb 1992;21(2):146-52. doi:10.1016/s0196-0644(05)80149-0
116. Shattuck KE, Chonmaitree T. The changing spectrum of neonatal meningitis over a fifteen-year period. *Clin Pediatr (Phila)*. Mar 1992;31(3):130-6. doi:10.1177/000992289203100301
117. Srair HA, Aman H, al-Madan M, al-Khater M. Bacterial meningitis in Saudi children. *Indian J Pediatr*. Nov-Dec 1992;59(6):719-21. doi:10.1007/BF02859407
118. Tefuarani N, Vince JD. Purulent meningitis in children: outcome using a standard management regimen with chloramphenicol. *Ann Trop Paediatr*. 1992;12(4):375-83. doi:10.1080/02724936.1992.11747602
119. Thomas DG. Outcome of paediatric bacterial meningitis 1979-1989. *Med J Aust*. Oct 19 1992;157(8):519-20. doi:10.5694/j.1326-5377.1992.tb137345.x
120. Airede AI. Neonatal bacterial meningitis in the middle belt of Nigeria. *Dev Med Child Neurol*. May 1993;35(5):424-30. doi:10.1111/j.1469-8749.1993.tb11664.x
121. Ballantyne ES, Chaseling R, Miller JD. When should patients with bacterial meningitis be referred to a neurosurgical unit? *Scott Med J*. Jun 1993;38(3):77-9. doi:10.1177/003693309303800306
122. Boehme C, Soto L, Rodriguez G, Serra J, Illesca V, Reydet P. [Three years of acute bacterial meningitis in the pediatric service at the Temuco Regional Hospital]. *Rev Med Chil*. Jun 1993;121(6):633-8. Tres anos de meningitis bacteriana aguda en servicio de pediatria del Hospital Regional de Temuco.
123. Brivet FG, Guibert M, Dormont J. Acute bacterial meningitis in adults. *N Engl J Med*. Jun 10 1993;328(23):1712-3.
124. Durand ML, Calderwood SB, Weber DJ, Miller SI, Southwick FS, Caviness VS, Swartz MN. Acute bacterial meningitis in adults. A review of 493 episodes. *The New England journal of medicine*. 1993-1-7 1993;328(1):21-8. doi:doi:
125. Fortnum HM, Davis AC. Epidemiology of bacterial meningitis. *Arch Dis Child*. Jun 1993;68(6):763-7. doi:10.1136/adc.68.6.763
126. Liu CC, Chen JS, Lin CH, Chen YJ, Huang CC. Bacterial meningitis in infants and children in southern Taiwan: emphasis on Haemophilus influenzae type B infection. *J Formos Med Assoc*. Oct 1993;92(10):884-8.
127. Pfister HW, Feiden W, Einhaupl KM. Spectrum of complications during bacterial meningitis in adults. Results of a prospective clinical study. *Arch Neurol*. Jun 1993;50(6):575-81. Not in File. doi:10.1001/archneur.1993.00540060015010
128. Ara JR, Cia P, Arribas JL, Aguirre JM, de Juan F, Marco Tello A. [Clinico-epidemiologic study of bacterial meningitis in Aragon]. *Med Clin (Barc)*. Nov 12 1994;103(16):611-4. Estudio clinicoepidemiologico de las meningitis bacterianas en Aragon.
129. Carroll KJ, Carroll C. A prospective investigation of the long-term auditory-neurological sequelae associated with bacterial meningitis: a study from Vanuatu. *J Trop Med Hyg*. Jun 1994;97(3):145-50.
130. Chotpitayasunondh T. Bacterial meningitis in children: etiology and clinical features, an 11-year review of 618 cases. *Southeast Asian J Trop Med Public Health*. Mar 1994;25(1):107-15.
131. Commey JO, Rodrigues OP, Akita FA, Newman M. Bacterial meningitis in children in southern Ghana. *East Afr Med J*. Feb 1994;71(2):113-7.
132. Dagan R, Isaachson M, Lang R, Karpuch J, Block C, Amir J. Epidemiology of pediatric meningitis caused by Haemophilus influenzae type b, Streptococcus pneumoniae, and Neisseria meningitidis in Israel: a 3-year nationwide prospective study. Israeli Pediatric Bacteremia and Meningitis Group. *J Infect Dis*. Apr 1994;169(4):912-6. doi:10.1093/infdis/169.4.912

133. Ford H, Wright J. Bacterial meningitis in Swaziland: an 18 month prospective study of its impact. *J Epidemiol Community Health*. Jun 1994;48(3):276-80. doi:10.1136/jech.48.3.276
134. Kallio MJ, Kilpi T, Anttila M, Peltola H. The effect of a recent previous visit to a physician on outcome after childhood bacterial meningitis. *JAMA*. Sep 14 1994;272(10):787-91.
135. Moreno MT, Vargas S, Poveda R, Saez-Llorens X. Neonatal sepsis and meningitis in a developing Latin American country. *Pediatr Infect Dis J*. Jun 1994;13(6):516-20. doi:10.1097/00006454-199406000-00010
136. Ozumba UC. Changing pattern of acute bacterial meningitis in Enugu, Nigeria. *East Afr Med J*. May 1994;71(5):300-3.
137. Synnott MB, Morse DL, Hall SM. Neonatal meningitis in England and Wales: a review of routine national data. *Arch Dis Child Fetal Neonatal Ed*. Sep 1994;71(2):F75-80. doi:10.1136/fn.71.2.f75
138. Ali Z. Neonatal meningitis: a 3-year retrospective study at the Mount Hope Women's Hospital, Trinidad, West Indies. *J Trop Pediatr*. Apr 1995;41(2):109-11. doi:10.1093/tropej/41.2.109
139. Almirante B, Cortes E, Pigrau C, Gasser I, del Valle O, Campos L, Pahissa A. [Treatment and outcome of pneumococcal meningitis in adults. Study of a recent series of 70 episodes]. *Med Clin (Barc)*. Nov 25 1995;105(18):681-6. Terapeutica y evolucion de la meningitis neumococica en el adulto. Estudio de una serie reciente de 70 episodios.
140. Ciana G, Parmar N, Antonio C, Pivetta S, Tamburlini G, Cuttini M. Effectiveness of adjunctive treatment with steroids in reducing short-term mortality in a high-risk population of children with bacterial meningitis. *J Trop Pediatr*. Jun 1995;41(3):164-8. Not in File. doi:10.1093/tropej/41.3.164
141. Daoud AS, al-Sheyyab M, Batchoun RG, Rawashdeh MO, Nussair MM, Pugh RN. Bacterial meningitis: still a cause of high mortality and severe neurological morbidity in childhood. *J Trop Pediatr*. Oct 1995;41(5):308-10. doi:10.1093/tropej/41.5.308
142. Gedlu E, Rahlenbeck SI. Pyogenic meningitis in children in north-western Ethiopia. *Ann Trop Paediatr*. Sep 1995;15(3):243-7. doi:10.1080/02724936.1995.11747779
143. Kaaresen PI, Flaegstad T. Prognostic factors in childhood bacterial meningitis. *Acta Paediatr*. Aug 1995;84(8):873-8. doi:10.1111/j.1651-2227.1995.tb13783.x
144. Kilpi T, Peltola H, Jauhiainen T, Kallio MJ. Oral glycerol and intravenous dexamethasone in preventing neurologic and audiologic sequelae of childhood bacterial meningitis. The Finnish Study Group. *Pediatr Infect Dis J*. Apr 1995;14(4):270-8. doi:10.1097/00006454-199504000-00005
145. Lecour H, Miranda AM, Nogueira JA, Abreu C. Update on the use of cefotaxime for pediatric meningitis in Portugal. *Diagn Microbiol Infect Dis*. May-Jun 1995;22(1-2):125-7. doi:10.1016/0732-8893(95)00085-o
146. Lutsar I, Siirde T, Soopold T. Long term follow-up of Estonian children after bacterial meningitis. *Pediatr Infect Dis J*. Jul 1995;14(7):624-5. doi:10.1097/00006454-199507000-00014
147. Patwari AK, Singh BS, Manorama DE. Inappropriate secretion of antidiuretic hormone in acute bacterial meningitis. *Ann Trop Paediatr*. Jun 1995;15(2):179-83. doi:10.1080/02724936.1995.11747769
148. Salaun-Saraux P, Saraux A, Lepage P, et al. [Septic meningitis in children in Rwanda from 1983 to 1990. Retrospective study at the Kigali Hospital Center]. *Med Trop (Mars)*. 1995;55(1):41-5. Les meningites septiques de l'enfant au Rwanda de 1983 a 1990. Etude retrospective au Centre Hospitalier de Kigali.
149. Singhi SC, Singhi PD, Srinivas B, Narakesri HP, Ganguli NK, Sialy R, Walia BN. Fluid restriction does not improve the outcome of acute meningitis. *Pediatr Infect Dis J*. Jun 1995;14(6):495-503. doi:10.1097/00006454-199506000-00006
150. Ahmed AA, Salih MA, Ahmed HS. Post-endemic acute bacterial meningitis in Sudanese children. *East Afr Med J*. Aug 1996;73(8):527-32.
151. Berg S, Trollfors B, Claesson BA, et al. Incidence and prognosis of meningitis due to Haemophilus influenzae, Streptococcus pneumoniae and Neisseria meningitidis in Sweden. *Scand J Infect Dis*. 1996;28(3):247-52. doi:10.3109/00365549609027166
152. Bergemann A, Karstaedt AS. The spectrum of meningitis in a population with high prevalence of HIV disease. *QJM*. Jul 1996;89(7):499-504. doi:10.1093/qjmed/89.7.499
153. Gomes I, Melo A, Lucena R, et al. Prognosis of bacterial meningitis in children. *Arq Neuropsiquiatr*. Sep 1996;54(3):407-11. doi:10.1590/s0004-282x1996000300008
154. Ichiyama T, Hayashi T, Furukawa S. Cerebrospinal fluid concentrations of soluble tumor necrosis factor receptor in bacterial and aseptic meningitis. *Neurology*. Mar 1996;46(3):837-8. doi:10.1212/wnl.46.3.837
155. Ishikawa T, Asano Y, Morishima T, Nagashima M, Sobue G, Watanabe K, Yamaguchi H. Epidemiology of bacterial meningitis in children: Aichi Prefecture, Japan, 1984-1993. *Pediatr Neurol*. Apr 1996;14(3):244-50. doi:10.1016/0887-8994(96)00024-0
156. Laguna del Estal P, Salgado Marques R, Calabrese Sanchez S, Murillas Angoitti J, Martin Alvarez E, Moya Mir MS. [Acute bacterial meningitis in adults: a clinical and developmental analysis of 100 cases]. *An Med Interna*. Nov 1996;13(11):520-6. Meningitis aguda bacteriana en adultos: analisis clinico y evolutivo de 100 casos.

157. Macaluso A, Pivetta S, Maggi RS, Tamburlini G, Cattaneo A. Dexamethasone adjunctive therapy for bacterial meningitis in children: a retrospective study in Brazil. *Ann Trop Paediatr*. Sep 1996;16(3):193-8. doi:10.1080/02724936.1996.11747825
158. Qazi SA, Khan MA, Mughal N, et al. Dexamethasone and bacterial meningitis in Pakistan. *Arch Dis Child*. Dec 1996;75(6):482-8. Not in File. doi:10.1136/adc.75.6.482
159. Awasthi S, Moin S, Iyer SM, Rehman H. Modified Glasgow Coma Scale to predict mortality in children with acute infections of the central nervous system. *Natl Med J India*. Sep-Oct 1997;10(5):214-6.
160. Grobler AC, Hay IT. Bacterial meningitis in children at Kalafong Hospital, 1990-1995. *S Afr Med J*. Aug 1997;87(8 Suppl):1052-4.
161. Hussey G, Schaaf H, Hanslo D, et al. Epidemiology of post-neonatal bacterial meningitis in Cape Town children. *S Afr Med J*. Jan 1997;87(1):51-6.
162. Imuekehme S, Obi J, Alakija W. Cerebro-spinal lactate status in childhood pyogenic meningitis in Nigeria. *J Trop Pediatr*. Dec 1997;43(6):361-3. doi:10.1093/tropej/43.6.361
163. Sigurdardottir B, Bjornsson OM, Jonsdottir KE, Erlendsdottir H, Gudmundsson S. Acute bacterial meningitis in adults. A 20-year overview. *Arch Intern Med*. Feb 24 1997;157(4):425-30. Not in File. doi:10.1001/archinte.1997.00440250077009
164. Sung RY, Senok AC, Ho A, Oppenheimer SJ, Davies DP. Meningitis in Hong Kong children, with special reference to the infrequency of haemophilus and meningococcal infection. *J Paediatr Child Health*. Aug 1997;33(4):296-9. doi:10.1111/j.1440-1754.1997.tb01603.x
165. Chang YC, Huang CC, Wang ST, Liu CC, Tsai JJ. Risk factors analysis for early fatality in children with acute bacterial meningitis. *Pediatr Neurol*. Mar 1998;18(3):213-7. doi:10.1016/s0887-8994(97)00184-7
166. Fernandez-Jaen A, Borque Andres C, del Castillo Martin F, Pena Garcia P, Vidal Lopez ML. [Bacterial meningitis in pediatrics. Study of 166 cases]. *An Esp Pediatr*. May 1998;48(5):495-8. Meningitis bacteriana en la edad pediatrica. Estudio de 166 casos.
167. Gutierrez A, Ramos MA, Sanz JC, Bernal A, Agirrezabal J, Casado Y, Martinez M. [Bacterial meningitis in emergency medicine. Factors associated with delay of antimicrobial therapy]. *Enferm Infecc Microbiol Clin*. Aug-Sep 1998;16(7):302-6. Meningitis purulenta en urgencias. Factores asociados al retraso del tratamiento antibiotico.
168. Honnas A, Petersen LT. Bacterial meningitis in a rural Kenyan hospital. *East Afr Med J*. Jul 1998;75(7):396-401.
169. Hussain IH, Sofiah A, Ong LC, Choo KE, Musa MN, Teh KH, Ng HP. Haemophilus influenzae meningitis in Malaysia. *Pediatr Infect Dis J*. Sep 1998;17(9 Suppl):S189-90. doi:10.1097/00006454-199809001-00021
170. Imananagha KK, Peters EJ, Philip-Ephraim EE, Ekott JU, Imananagha LN, Ekure EN, Esin RA. Acute bacterial meningitis in a developing country: diagnosis related mortality among paediatric patients. *Cent Afr J Med*. Jan 1998;44(1):11-5.
171. Kim KH, Sohn YM, Kang JH, et al. The causative organisms of bacterial meningitis in Korean children, 1986-1995. *J Korean Med Sci*. Feb 1998;13(1):60-4. doi:10.3346/jkms.1998.13.1.60
172. Molyneux E, Walsh A, Phiri A, Molyneux M. Acute bacterial meningitis in children admitted to the Queen Elizabeth Central Hospital, Blantyre, Malawi in 1996-97. *Trop Med Int Health*. Aug 1998;3(8):610-8. Not in File. doi:10.1046/j.1365-3156.1998.00278.x
173. Muller M, Merkelbach S, Hermes M, Konig J, Schimrigk K. Relationship between short-term outcome and occurrence of cerebral artery stenosis in survivors of bacterial meningitis. *J Neurol*. Feb 1998;245(2):87-92. doi:10.1007/s004150050183
174. Rios-Reategui E, Ruiz-Gonzalez L, Murguia-de-Sierra T. [Neonatal bacterial meningitis in a tertiary treatment center]. *Rev Invest Clin*. Jan-Feb 1998;50(1):31-6. Meningitis bacteriana neonatal en una institucion de tercer nivel de atencion.
175. Schutte CM, van der Meyden CH. A prospective study of Glasgow Coma Scale (GCS), age, CSF-neutrophil count, and CSF-protein and glucose levels as prognostic indicators in 100 adult patients with meningitis. *J Infect*. Sep 1998;37(2):112-5. doi:10.1016/s0163-4453(98)80163-1
176. Shembesh NM, el Bargathy SM, Rao BN, Kashbur IM. A prospective study of bacterial meningitis in children from north-eastern Libya. *Ann Trop Paediatr*. Sep 1998;18(3):203-7. doi:10.1080/02724936.1998.11747948
177. Campagne G, Schuchat A, Djibo S, Ousseini A, Cisse L, Chippaux JP. Epidemiology of bacterial meningitis in Niamey, Niger, 1981-96. *Bull World Health Organ*. 1999 1999;77(6):499-508. Not in File.
178. Daoud AS, Batieha A, Al-Sheyyab M, Abuekteish F, Obeidat A, Mahafza T. Lack of effectiveness of dexamethasone in neonatal bacterial meningitis. *Eur J Pediatr*. Mar 1999;158(3):230-3. Not in File. doi:10.1007/s004310051056
179. Dawson KG, Emerson JC, Burns JL. Fifteen years of experience with bacterial meningitis. *Pediatr Infect Dis J*. Sep 1999;18(9):816-22. doi:10.1097/00006454-199909000-00014

180. Moyen G, Mbika-Cardorelle A. [Bacterial meningitis in infants and children at the Brazzaville University Hospital]. *Arch Pediatr*. Jan 1999;6(1):108-9. Meningites bacteriennes du nourrisson et de l'enfant au CHU de Brazzaville. doi:10.1016/s0929-693x(99)80088-8
181. Nathoo KJ, Bannerman CH, Pirie DJ. Pattern of admissions to the paediatric medical wards (1995 to 1996) at Harare Hospital, Zimbabwe. *Cent Afr J Med*. Oct 1999;45(10):258-63. doi:10.4314/cajm.v45i10.8496
182. Okome-Nkoumou M, Loembe PM. [Bacterial meningitis in the adult. Study of 85 cases observed in the infectious disease unit of the Fondation Jeanne Ebori (F.J.E.), Libreville, Gabon]. *Bull Soc Pathol Exot*. Dec 1999;92(5):288-91. Les meningites bacteriennes de l'adulte. Etude de 85 cas observes dans l'unite des maladies infectieuses de la Fondation Jeanne Ebori (F. J. E.), Libreville, Gabon.
183. Palmer A, Weber M, Bojang K, McKay T, Adegbola R. Acute bacterial meningitis in The Gambia: a four-year review of paediatric hospital admissions. *J Trop Pediatr*. Feb 1999;45(1):51-3. doi:10.1093/tropej/45.1.51
184. Pena JA, Jimenez L. [Prognosis of bacterial meningitis]. *Rev Neurol*. Aug 16-31 1999;29(4):311-5. Pronostico de la meningitis bacteriana.
185. Silber E, Sonnenberg P, Ho KC, Koornhof HJ, Eintracht S, Morris L, Saffer D. Meningitis in a community with a high prevalence of tuberculosis and HIV infection. *J Neurol Sci*. Jan 1 1999;162(1):20-6. doi:10.1016/s0022-510x(98)00259-7
186. Struillou L, Ninin E, Berranger C, et al. [Community-acquired bacterial meningitis in the Loire-Atlantic region: evolution of pneumococcal and meningococcal sensitivity to penicillin]. *Presse Med*. Feb 27 1999;28(8):389-94. Les meningites bacteriennes communautaires en Loire-Atlantique: evolution de la sensibilite a la penicilline des pneumocoques et meningocoques.
187. Tang LM, Chen ST, Hsu WC, Lyu RK. Acute bacterial meningitis in adults: a hospital-based epidemiological study. *QJM*. Dec 1999;92(12):719-25. doi:10.1093/qjmed/92.12.719
188. Chotmongkol V, Techoruangwiwat C. Community acquired-bacterial meningitis in adults. *Southeast Asian J Trop Med Public Health*. Sep 2000;31(3):506-8.
189. Gordon SB, Walsh AL, Chaponda M, et al. Bacterial meningitis in Malawian adults: pneumococcal disease is common, severe, and seasonal. *Clin Infect Dis*. Jul 2000;31(1):53-7. doi:10.1086/313910
190. Klinger G, Chin CN, Beyene J, Perlman M. Predicting the outcome of neonatal bacterial meningitis. *Pediatrics*. Sep 2000;106(3):477-82. doi:10.1542/peds.106.3.477
191. Moller K, Hogh P, Larsen FS, Strauss GI, Skinhoj P, Sperling BK, Knudsen GM. Regional cerebral blood flow during hyperventilation in patients with acute bacterial meningitis. *Clin Physiol*. Sep 2000;20(5):399-410. doi:10.1046/j.1365-2281.2000.00276.x
192. Nel E. Neonatal meningitis: mortality, cerebrospinal fluid, and microbiological findings. *J Trop Pediatr*. Aug 2000;46(4):237-9. Not in File. doi:10.1093/tropej/46.4.237
193. Ray G, Aneja S, Jain M, Batra S. Evaluation of free radical status in CSF in childhood meningitis. *Ann Trop Paediatr*. Jun 2000;20(2):115-20. doi:10.1080/02724936.2000.11748119
194. Zanelli S, Gillet Y, Stamm D, Lina G, Floret D. [Bacterial meningitis in infants 1 to 8 weeks old]. *Arch Pediatr*. Jun 2000;7 Suppl 3:565s-571s. Meningites bacteriennes du nourrisson age de une a huit semaines. doi:10.1016/s0929-693x(00)80185-2
195. Almuneef M, Alshaalan M, Memish Z, Alalola S. Bacterial meningitis in Saudi Arabia: the impact of Haemophilus influenzae type b vaccination. *J Chemother*. Apr 2001;13 Suppl 1:34-9. doi:10.1080/1120009x.2001.11782326
196. Berkley JA, Mwangi I, Ngetsa CJ, Mwarumba S, Lowe BS, Marsh K, Newton CR. Diagnosis of acute bacterial meningitis in children at a district hospital in sub-Saharan Africa. *Lancet*. Jun 2 2001;357(9270):1753-7. Not in File. doi:10.1016/S0140-6736(00)04897-2
197. Bonsu BK, Harper MB. Fever interval before diagnosis, prior antibiotic treatment, and clinical outcome for young children with bacterial meningitis. *Clin Infect Dis*. Feb 15 2001;32(4):566-72. doi:10.1086/318700
198. Holt DE, Halket S, de Louvois J, Harvey D. Neonatal meningitis in England and Wales: 10 years on. *Arch Dis Child Fetal Neonatal Ed*. Mar 2001;84(2):F85-9. Not in File. doi:10.1136/fn.84.2.f85
199. Johnson WB, Adedoyin OT, Abdulkarim AA, Olanrewaju WI. Bacterial pathogens and outcome determinants of childhood pyogenic meningitis in Ilorin, Nigeria. *Afr J Med Med Sci*. Dec 2001;30(4):295-303.
200. Madhi SA, Madhi A, Petersen K, Khoosal M, Klugman KP. Impact of human immunodeficiency virus type 1 infection on the epidemiology and outcome of bacterial meningitis in South African children. *Int J Infect Dis*. 2001 2001;5(3):119-25. Not in File. doi:10.1016/s1201-9712(01)90085-2
201. McMillan DA, Lin CY, Aronin SI, Quagliarello VJ. Community-acquired bacterial meningitis in adults: categorization of causes and timing of death. *Clinical infectious diseases : an official publication of the Infectious Diseases Society of America*. 2001-1-1 2001;33(7):969-75. doi:doi:

202. Miner JR, Heegaard W, Mapes A, Biros M. Presentation, time to antibiotics, and mortality of patients with bacterial meningitis at an urban county medical center. *J Emerg Med*. Nov 2001;21(4):387-92. doi:10.1016/s0736-4679(01)00407-3
203. Neuman HB, Wald ER. Bacterial meningitis in childhood at the Children's Hospital of Pittsburgh: 1988-1998. *Clin Pediatr (Phila)*. Nov 2001;40(11):595-600. doi:10.1177/000992280104001102
204. Sahai S, Mahadevan S, Srinivasan S, Kanungo R. Childhood bacterial meningitis in Pondicherry, South India. *Indian J Pediatr*. Sep 2001;68(9):839-41. doi:10.1007/BF02762107
205. Weiss DP, Coplan P, Guess H. Epidemiology of bacterial meningitis among children in Brazil, 1997-1998. *Rev Saude Publica*. Jun 2001;35(3):249-55. doi:10.1590/s0034-89102001000300006
206. Ahsan T, Shahid M, Mahmood T, et al. Role of dexamethasone in acute bacterial meningitis in adults. *J Pak Med Assoc*. Jun 2002;52(6):233-9.
207. Barboza AG, Ioli P, Zamarbide I, Estrago MI, Castineiras F, de Wouters L. [A study of the incidence and a descriptive analysis of adult non-tuberculous primary bacterial meningitis in a population in Argentina]. *Rev Neurol*. Sep 16-30 2002;35(6):508-12. Estudio de incidencia y analisis descriptivo de la meningitis bacteriana primaria no tuberculosa del adulto en una poblacion argentina.
208. Beyrer K, Dreesman J, Thielen H, Windorfer A. [Surveillance system for assessing central nervous infections in Lower Saxony 1998-2000]. *Gesundheitswesen*. Jun 2002;64(6):336-43. Surveillance-System zur Erfassung zentralnervoser Infektionen in Niedersachsen 1998-2000. doi:10.1055/s-2002-32177
209. Chan YC, Wilder-Smith A, Ong BK, Kumarasinghe G, Wilder-Smith E. Adult community acquired bacterial meningitis in a Singaporean teaching hospital. A seven-year overview (1993-2000). *Singapore Med J*. Dec 2002;43(12):632-6.
210. Chinchankar N, Mane M, Bhave S, et al. Diagnosis and outcome of acute bacterial meningitis in early childhood. *Indian Pediatr*. Oct 2002;39(10):914-21.
211. Duke T, Mokela D, Frank D, Michael A, Paulo T, Mgone J, Kurubi J. Management of meningitis in children with oral fluid restriction or intravenous fluid at maintenance volumes: a randomised trial. *Ann Trop Paediatr*. Jun 2002;22(2):145-57. doi:10.1179/027249302125000878
212. Hemalatha R, Bhaskaram P, Balakrishna N, Saraswathi I. Association of tumour necrosis factor alpha & malnutrition with outcome in children with acute bacterial meningitis. *Indian J Med Res*. Feb 2002;115:55-8.
213. Lopez Sastre J, Castrillo" GdH. [Neonatal meningitis. Epidemiological study of the Grupo de Hospitales Castrillo]. *An Esp Pediatr*. Jun 2002;56(6):556-63. Meningitis neonatal. Estudio epidemiologico del Grupo de Hospitales Castrillo.
214. Migliani R, Clouzeau J, Decousser JW, et al. [Non-tubercular bacterial meningitis in children in Antananarivo, Madagascar]. *Arch Pediatr*. Sep 2002;9(9):892-7. Les meningites bacteriennes non tuberculeuses de l'enfant a Antananarivo, Madagascar. doi:10.1016/s0929-693x(02)00018-0
215. Molyneux EM, Walsh AL, Forsyth H, et al. Dexamethasone treatment in childhood bacterial meningitis in Malawi: a randomised controlled trial. *Lancet*. Jul 20 2002;360(9328):211-8. Not in File. doi:10.1016/s0140-6736(02)09458-8
216. Mwangi I, Berkley J, Lowe B, Peshu N, Marsh K, Newton CR. Acute bacterial meningitis in children admitted to a rural Kenyan hospital: increasing antibiotic resistance and outcome. *Pediatr Infect Dis J*. Nov 2002;21(11):1042-8. Not in File. doi:10.1097/00006454-200211000-00013
217. Oostenbrink R, Moons KG, Derksen-Lubsen G, Grobbee DE, Moll HA. Early prediction of neurological sequelae or death after bacterial meningitis. *Acta Paediatr*. 2002 2002;91(4):391-8. Not in File. doi:10.1080/080352502317371616
218. Al-Mazrou YY, Musa EK, Abdalla MN, Al-Jeffri MH, Al-Hajjar SH, Mohamed OM. Disease burden and case management of bacterial meningitis among children under 5 years of age in Saudi Arabia. *Saudi Med J*. Dec 2003;24(12):1300-7.
219. Chang CJ, Chang WN, Huang LT, et al. Neonatal bacterial meningitis in southern Taiwan. *Pediatr Neurol*. Oct 2003;29(4):288-94. doi:10.1016/s0887-8994(03)00273-x
220. Flores-Cordero JM, Amaya-Villar R, Rincon-Ferrari MD, Leal-Noval SR, Garnacho-Montero J, Llanos-Rodriguez AC, Murillo-Cabezas F. Acute community-acquired bacterial meningitis in adults admitted to the intensive care unit: clinical manifestations, management and prognostic factors. *Intensive Care Med*. Nov 2003;29(11):1967-73. doi:10.1007/s00134-003-1935-4
221. Kirimi E, Tuncer O, Arslan S, et al. Prognostic factors in children with purulent meningitis in Turkey. *Acta Med Okayama*. Feb 2003;57(1):39-44. doi:10.18926/AMO/32839
222. Rabbani MA, Khan AA, Ali SS, Ahmad B, Baig SM, Khan MA, Wasay M. Spectrum of complications and mortality of bacterial meningitis: an experience from a developing country. *J Pak Med Assoc*. Dec 2003;53(12):580-3.
223. Celal A, Faruk GM, Salih H, Kemal CM, Serife A, Faruk KO. Characteristics of acute bacterial meningitis in Southeast Turkey. *Indian J Med Sci*. Aug 2004;58(8):327-33.
224. Khwannimit B, Chayakul P, Geater A. Acute bacterial meningitis in adults: a 20 year review. *Southeast Asian J Trop Med Public Health*. Dec 2004;35(4):886-92.

225. Luca V, Gessner BD, Luca C, et al. Incidence and etiological agents of bacterial meningitis among children <5 years of age in two districts of Romania. *Eur J Clin Microbiol Infect Dis*. Jul 2004;23(7):523-8. doi:10.1007/s10096-004-1169-6
226. Ostergaard C, Benfield T, Lundgren JD, Eugen-Olsen J. Soluble urokinase receptor is elevated in cerebrospinal fluid from patients with purulent meningitis and is associated with fatal outcome. *Scand J Infect Dis*. 2004;36(1):14-9. doi:10.1080/00365540310017366
227. Sallam AK. Etiology and presentation of acute bacterial meningitis in children at Al-Thawrah Hospital, Sana'a, Yemen. *J Ayub Med Coll Abbottabad*. Oct-Dec 2004;16(4):40-3.
228. Singhi SC, Khetarpal R, Baranwal AK, Singhi PD. Intensive care needs of children with acute bacterial meningitis: a developing country perspective. *Ann Trop Paediatr*. Jun 2004;24(2):133-40. doi:10.1179/027249304225013402
229. van de Beek D, de Gans J, Spanjaard L, Weisfelt M, Reitsma JB, Vermeulen M. Clinical features and prognostic factors in adults with bacterial meningitis. *N Engl J Med*. Oct 28 2004;351(18):1849-59. Not in File. doi:10.1056/NEJMoa040845
230. Wiersinga WJ, van Dellen QM, Spanjaard L, van Kan HJ, Groen AL, Wetsteyn JC. High mortality among patients with bacterial meningitis in a rural hospital in Tanzania. *Ann Trop Med Parasitol*. Apr 2004;98(3):271-8. doi:10.1179/000349804225003235
231. Amsalu S, Assefa A. Meningitis in children beyond the neonatal period in Gondar University hospital. *Ethiop Med J*. Jul 2005;43(3):175-80.
232. Bekondi C, Bernede C, Passone N, Minssart P, Kamalo C, Mbolidi D, Germani Y. Primary and opportunistic pathogens associated with meningitis in adults in Bangui, Central African Republic, in relation to human immunodeficiency virus serostatus. *Int J Infect Dis*. Sep 2006;10(5):387-95. doi:10.1016/j.ijid.2005.07.004
233. Deeks SL, MacDonald DM, Squires SG, Medaglia A, Tam T. Bacterial meningitis in Canada: hospitalizations (1994-2001). *Can Commun Dis Rep*. Dec 1 2005;31(23):241-7.
234. Farag H, Abdel-Fattah M, Youssri A. Epidemiological, clinical and prognostic profile of acute bacterial meningitis among children in Alexandria, Egypt. *Indian Journal of Medical Microbiology*. 2005;23(2):95-101.
235. Hui AC, Ng KC, Tong PY, Mok V, Chow KM, Wu A, Wong LK. Bacterial meningitis in Hong Kong: 10-years' experience. *Clin Neurol Neurosurg*. Aug 2005;107(5):366-70. doi:10.1016/j.clineuro.2004.10.006
236. Lucena R, Fonseca N, Nunes L, et al. Intra-hospital lethality among infants with pyogenic meningitis. *Pediatr Neurol*. Mar 2005;32(3):180-3. doi:10.1016/j.pediatrneurol.2004.09.016
237. May M, Daley AJ, Donath S, Isaacs D, Australasian Study Group for Neonatal I. Early onset neonatal meningitis in Australia and New Zealand, 1992-2002. *Arch Dis Child Fetal Neonatal Ed*. Jul 2005;90(4):F324-7. Not in File. doi:10.1136/adc.2004.066134
238. Odetola FO, Bratton SL. Characteristics and immediate outcome of childhood meningitis treated in the pediatric intensive care unit. *Intensive Care Med*. Jan 2005;31(1):92-7. doi:10.1007/s00134-004-2501-4
239. Ogunlesi TA, Okeniyi JA, Oyelami OA. Pyogenic meningitis in Ilesa, Nigeria. *Indian Pediatr*. Oct 2005;42(10):1019-23.
240. Al Khorasani A, Banajeh S. Bacterial profile and clinical outcome of childhood meningitis in rural Yemen: a 2-year hospital-based study. *J Infect*. Oct 2006;53(4):228-34. doi:10.1016/j.jinf.2005.12.004
241. Bregani ER, Tarsia P, Pujades E, Van Tien T, Arioli M, Ziglioli E. The 2001 meningitis epidemic in south Chad. *Minerva Med*. Apr 2006;97(2):161-73.
242. Elsaid MF, Flamerzi AA, Bessisso MS, Elshafie SS. Acute bacterial meningitis in Qatar. *Saudi Med J*. Feb 2006;27(2):198-204.
243. Garges HP, Moody MA, Cotten CM, et al. Neonatal meningitis: what is the correlation among cerebrospinal fluid cultures, blood cultures, and cerebrospinal fluid parameters? *Pediatrics*. Apr 2006;117(4):1094-100. doi:10.1542/peds.2005-1132
244. Mbelesso P, Tatangba-Bakozo A, Fikouma V. [Bacterial meningiditis in adult patients in Central African hospitals]. *Bull Soc Pathol Exot*. Oct 2006;99(4):261-3. Les meningites bacteriennes de l'adulte en milieu hospitalier centrafricain.
245. Molyneux E, Riordan FA, Walsh A. Acute bacterial meningitis in children presenting to the Royal Liverpool Children's Hospital, Liverpool, UK and the Queen Elizabeth Central Hospital in Blantyre, Malawi: a world of difference. *Ann Trop Paediatr*. Mar 2006;26(1):29-37. Not in File. doi:10.1179/146532806X90583
246. Pizon AF, Bonner MR, Wang HE, Kaplan RM. Ten years of clinical experience with adult meningitis at an urban academic medical center. *J Emerg Med*. May 2006;30(4):367-70. doi:10.1016/j.jemermed.2005.07.010
247. Shabani IS, Al-Ateeqi W, Abu-Shanab O, El-Sori H, Omar N, Ahmed HF, Al-Musallam M. Childhood meningitis in Kuwait: epidemiology of etiologic agents and the need for pneumococcal disease prevention. *Med Princ Pract*. 2006-1-1 2006;15(6):431-5. doi:10.1159/000095489
248. Singhi SC, Bansal A. Serum cortisol levels in children with acute bacterial and aseptic meningitis. *Pediatr Crit Care Med*. Jan 2006;7(1):74-8. doi:10.1097/01.pcc.0000192317.90862.44

249. Afifi S, Wasfy MO, Azab MA, et al. Laboratory-based surveillance of patients with bacterial meningitis in Egypt (1998-2004). *Eur J Clin Microbiol Infect Dis*. May 2007;26(5):331-40. doi:10.1007/s10096-007-0280-x
250. Boisier P, Mainassara HB, Sidikou F, Djibo S, Kairo KK, Chanteau S. Case-fatality ratio of bacterial meningitis in the African meningitis belt: we can do better. *Vaccine*. Sep 3 2007;25 Suppl 1:A24-9. doi:10.1016/j.vaccine.2007.04.036
251. Dauchy FA, Gruson D, Chene G, et al. Prognostic factors in adult community-acquired bacterial meningitis: a 4-year retrospective study. *Eur J Clin Microbiol Infect Dis*. Oct 2007;26(10):743-6. doi:10.1007/s10096-007-0381-6
252. Faustini A, Arca M, Fusco D, Perucci CA. Prognostic factors and determinants of fatal outcome due to bacterial meningitis in the Lazio region of Italy, 1996-2000. *Int J Infect Dis*. Mar 2007;11(2):137-44. doi:10.1016/j.ijid.2005.12.004
253. Johnson AW, Adedoyin OT, Abdul-Karim AA, Olanrewaju AW. Childhood pyogenic meningitis: clinical and investigative indicators of etiology and outcome. *J Natl Med Assoc*. Aug 2007;99(8):937-47.
254. Krebs VL, Costa GA. Clinical outcome of neonatal bacterial meningitis according to birth weight. *Arq Neuropsiquiatr*. Dec 2007;65(4B):1149-53. doi:10.1590/s0004-282x2007000700011
255. Lepur D, Barsic B. Community-acquired bacterial meningitis in adults: antibiotic timing in disease course and outcome. *Infection*. Jun 2007;35(4):225-31. doi:10.1007/s15010-007-6202-0
256. Nguyen TH, Tran TH, Thwaites G, et al. Dexamethasone in Vietnamese adolescents and adults with bacterial meningitis. *N Engl J Med*. Dec 13 2007;357(24):2431-40. Not in File. doi:10.1056/NEJMoa070852
257. Peltola H, Roine I, Fernandez J, et al. Adjuvant glycerol and/or dexamethasone to improve the outcomes of childhood bacterial meningitis: a prospective, randomized, double-blind, placebo-controlled trial. *Clin Infect Dis*. Nov 15 2007;45(10):1277-86. Not in File. doi:10.1086/522534
258. Scarborough M, Gordon SB, Whitty CJ, et al. Corticosteroids for bacterial meningitis in adults in sub-Saharan Africa. *N Engl J Med*. Dec 13 2007;357(24):2441-50. Not in File. doi:10.1056/NEJMoa065711
259. Theodoridou MN, Vasilopoulou VA, Atsali EE, Pangalis AM, Mostrou GJ, Syriopoulou VP, Hadjichristodoulou CS. Meningitis registry of hospitalized cases in children: epidemiological patterns of acute bacterial meningitis throughout a 32-year period. *BMC Infect Dis*. Aug 30 2007;7:101. Not in File. doi:10.1186/1471-2334-7-101
260. Airede KI, Adeyemi O, Ibrahim T. Neonatal bacterial meningitis and dexamethasone adjunctive usage in Nigeria. *Niger J Clin Pract*. Sep 2008;11(3):235-45.
261. Bercion R, Bobossi-Serengbe G, Gody JC, Beyam EN, Manirakiza A, Le Faou A. Acute bacterial meningitis at the 'Complexe Pediatrique' of Bangui, Central African Republic. *J Trop Pediatr*. Apr 2008;54(2):125-8. doi:10.1093/tropej/fmm075
262. Franco-Paredes C, Lammoglia L, Hernandez I, Santos-Preciado JI. Epidemiology and outcomes of bacterial meningitis in Mexican children: 10-year experience (1993-2003). *Int J Infect Dis*. Jul 2008;12(4):380-6. Not in File. doi:10.1016/j.ijid.2007.09.012
263. Lagunju IA, Falade AG, Akinbami FO, Adegbola R, Bakare RA. Childhood bacterial meningitis in Ibadan, Nigeria--antibiotic sensitivity pattern of pathogens, prognostic indices and outcome. *Afr J Med Med Sci*. Jun 2008;37(2):185-91.
264. Lazzarini L, Toti M, Fabris P, et al. Clinical features of bacterial meningitis in Italy: a multicenter prospective observational study. *J Chemother*. Aug 2008;20(4):478-87. doi:10.1179/joc.2008.20.4.478
265. Mongelluzzo J, Mohamad Z, Ten Have TR, Shah SS. Corticosteroids and mortality in children with bacterial meningitis. *JAMA*. May 7 2008;299(17):2048-55. doi:10.1001/jama.299.17.2048
266. Pelkonen T, Roine I, Monteiro L, et al. Acute childhood bacterial meningitis in Luanda, Angola. *Scand J Infect Dis*. 2008;40(11-12):859-66. doi:10.1080/00365540802262091
267. Sigauque B, Roca A, Sanz S, et al. Acute bacterial meningitis among children, in Manhica, a rural area in Southern Mozambique. *Acta Trop*. Jan 2008;105(1):21-7. doi:10.1016/j.actatropica.2007.01.006
268. Cabellos C, Verdaguer R, Olmo M, et al. Community-acquired bacterial meningitis in elderly patients: experience over 30 years. *Medicine (Baltimore)*. Mar 2009;88(2):115-119. Not in File. doi:10.1097/MD.0b013e31819d50ef
269. Dzupova O, Rozsypal H, Prochazka B, Benes J. Acute bacterial meningitis in adults: predictors of outcome. *Scand J Infect Dis*. 2009-1-1 2009;41(5):348-54. doi:10.1080/00365540902849391
270. Gurley ES, Hossain MJ, Montgomery SP, et al. Etiologies of bacterial meningitis in Bangladesh: results from a hospital-based study. *Am J Trop Med Hyg*. Sep 2009;81(3):475-83.
271. Ishihara M, Kamei S, Taira N, et al. Hospital-based study of the prognostic factors in adult patients with acute community-acquired bacterial meningitis in Tokyo, Japan. *Intern Med*. 2009;48(5):295-300. doi:10.2169/internalmedicine.48.1508
272. Roca A, Bassat Q, Morais L, et al. Surveillance of acute bacterial meningitis among children admitted to a district hospital in rural Mozambique. *Clin Infect Dis*. Mar 1 2009;48 Suppl 2:S172-80. doi:10.1086/596497
273. Tiskumara R, Fakharee SH, Liu CQ, et al. Neonatal infections in Asia. *Arch Dis Child Fetal Neonatal Ed*. Mar 2009;94(2):F144-8. doi:10.1136/adc.2008.139865

274. Traore Y, Tameklo TA, Njanpop-Lafourcade BM, et al. Incidence, seasonality, age distribution, and mortality of pneumococcal meningitis in Burkina Faso and Togo. *Clin Infect Dis*. Mar 1 2009;48 Suppl 2:S181-9. Not in File. doi:10.1086/596498
275. Abdulrab A, Algobaty F, Salem AK, Mohammed YA. Acute bacterial meningitis in adults: a hospital based study in Yemen. *Jpn J Infect Dis*. Mar 2010;63(2):128-31.
276. Aletayeb MH, Ahmad FS, Masood D. Eleven-year study of causes of neonatal bacterial meningitis in Ahvaz, Iran. *Pediatr Int*. Jun 2010;52(3):463-6. doi:10.1111/j.1442-200X.2010.03107.x
277. Ba O, Fleming JA, Dieye Y, et al. Hospital surveillance of childhood bacterial meningitis in Senegal and the introduction of Haemophilus influenzae type b conjugate vaccine. *Am J Trop Med Hyg*. Dec 2010;83(6):1330-5. doi:10.4269/ajtmh.2010.10-0346
278. Bentlin MR, Ferreira GL, Rugolo LM, Silva GH, Mondelli AL, Rugolo Junior A. Neonatal meningitis according to the microbiological diagnosis: a decade of experience in a tertiary center. *Arq Neuropsiquiatr*. Dec 2010;68(6):882-7. doi:10.1590/s0004-282x2010000600010
279. Cho HK, Lee H, Kang JH, et al. The causative organisms of bacterial meningitis in Korean children in 1996-2005. *J Korean Med Sci*. Jun 2010;25(6):895-9. doi:10.3346/jkms.2010.25.6.895
280. Erdem H, Kilic S, Coskun O, et al. Community-acquired acute bacterial meningitis in the elderly in Turkey. *Clin Microbiol Infect*. Aug 2010;16(8):1223-9. doi:10.1111/j.1469-0691.2009.03039.x
281. Hudeckova H, Jesenak M, Maria A, Svihrova V, Banovcin P. National analysis of bacterial meningitis in Slovakia, 1997-2007. *Public Health Rep*. Jan-Feb 2010;125(1):129-36. doi:10.1177/003335491012500117
282. Mankhambo LA, Banda DL, Group IPDS, et al. The role of angiogenic factors in predicting clinical outcome in severe bacterial infection in Malawian children. *Crit Care*. 2010;14(3):R91. doi:10.1186/cc9025
283. Moon SY, Chung DR, Kim SW, et al. Changing etiology of community-acquired bacterial meningitis in adults: a nationwide multicenter study in Korea. *Eur J Clin Microbiol Infect Dis*. Jul 2010;29(7):793-800. doi:10.1007/s10096-010-0929-8
284. Perez AE, Dickinson FO, Rodriguez M. Community acquired bacterial meningitis in Cuba: a follow up of a decade. *BMC Infect Dis*. May 25 2010;10:130. doi:10.1186/1471-2334-10-130
285. Su CM, Chang WN, Tsai NW, Huang CR, Wang HC, Lu CH. Clinical features and outcome of community-acquired bacterial meningitis in adult patients with liver cirrhosis. *Am J Med Sci*. Dec 2010;340(6):452-6. doi:10.1097/MAJ.0b013e3181ee988d
286. Talbert AW, Mwaniki M, Mwarumba S, Newton CR, Berkley JA. Invasive bacterial infections in neonates and young infants born outside hospital admitted to a rural hospital in Kenya. *Pediatr Infect Dis J*. Oct 2010;29(10):945-9. doi:10.1097/INF.0b013e3181dfca8c
287. Vibha D, Bhatia R, Prasad K, Srivastava MV, Tripathi M, Singh MB. Clinical features and independent prognostic factors for acute bacterial meningitis in adults. *Neurocrit Care*. Oct 2010;13(2):199-204. doi:10.1007/s12028-010-9396-4
288. Ajdukiewicz KM, Cartwright KE, Scarborough M, et al. Glycerol adjuvant therapy in adults with bacterial meningitis in a high HIV seroprevalence setting in Malawi: a double-blind, randomised controlled trial. *Lancet Infect Dis*. Apr 2011;11(4):293-300. Not in File. doi:10.1016/S1473-3099(10)70317-0
289. Pelkonen T, Roine I, Cruzeiro ML, Pitkaranta A, Kataja M, Peltola H. Slow initial beta-lactam infusion and oral paracetamol to treat childhood bacterial meningitis: a randomised, controlled trial. *Lancet Infect Dis*. Aug 2011;11(8):613-21. Not in File. doi:10.1016/S1473-3099(11)70055-X
290. Thigpen MC, Whitney CG, Messonnier NE, et al. Bacterial meningitis in the United States, 1998-2007. *N Engl J Med*. May 26 2011;364(21):2016-25. doi:10.1056/NEJMoa1005384
291. Vashishtha VM, Garg A, John TJ. Etiology of acute bacterial meningitis in hospitalized children in western Uttar Pradesh. *Indian Pediatr*. Dec 2011;48(12):985-6.
292. Organization" WH. Meningitis in Burkina Faso, Chad, Niger, Nigeria and Ghana: 2010 epidemic season. *Wkly Epidemiol Rec*. Apr 8 2011;86(15):143-51.
293. Fonseca de Souza S, Costa Mda C, Paim JS, Natividade MS, Pereira SM, Andrade AM, Teixeira MG. Bacterial meningitis and living conditions. *Rev Soc Bras Med Trop*. Jun 2012;45(3):323-8. doi:10.1590/s0037-86822012000300009
294. Juganariu G, Miftode E, Teodor D, Leca D, Dorobat CM. Clinical features and course of bacterial meningitis in children. *Rev Med Chir Soc Med Nat Iasi*. Jul-Sep 2012;116(3):722-6.
295. Kra O, Ouattara B, Aba T, Kadjane NJ, Kadjo K, Bissagnene E, Kadio A. [Morbidity and mortality from infectious diseases at the Military Hospital of Abidjan, Cote d'Ivoire]. *Med Sante Trop*. Jan-Mar 2012;22(1):75-8. Morbimortalite des pathologies infectieuses a l'hopital militaire d'Abidjan, Cote d'Ivoire. doi:10.1684/mst.2012.0043
296. Namani S, Milenkovic Z, Kuchar E, Koci R, Mehmeti M. Mortality from bacterial meningitis in children in Kosovo. *J Child Neurol*. Jan 2012;27(1):46-50. doi:10.1177/0883073811413280

297. Nansera D, Max I, Annet K, Gessner BD. Bacterial meningitis among children under the age of 2 years in a high human immunodeficiency virus prevalence area after Haemophilus influenzae type b vaccine introduction. *J Paediatr Child Health*. Apr 2012;48(4):324-8. doi:10.1111/j.1440-1754.2011.02235.x
298. Tarvij Eslami S, Nassirian H, Mojgan BM, Bahieh ZZ, Elham H, Alimohamad N, Ehsan S. Comparison of cerebrospinal fluid in newborns and in infants </= 2 months old with or without meningitis. *Pediatr Int*. Jun 2012;54(3):336-40. doi:10.1111/j.1442-200X.2011.03551.x
299. Vazquez JA, Adducci Mdel C, Coll C, Godoy Monzon D, Iserson KV. Acute meningitis prognosis using cerebrospinal fluid interleukin-6 levels. *J Emerg Med*. Aug 2012;43(2):322-7. doi:10.1016/j.jemermed.2011.07.029
300. Ben Hamouda H, Ben Haj Khalifa A, Hamza MA, Ayadi A, Soua H, Khedher M, Sfar MT. [Clinical outcome and prognosis of neonatal bacterial meningitis]. *Arch Pediatr*. Sep 2013;20(9):938-44. Aspects cliniques et evolutifs des meningites bacteriennes neonatales. doi:10.1016/j.arcped.2013.05.005
301. Butsashvili M, Kandelaki G, Eloshvili M, Chlikadze R, Imnadze P, Avaliani N. Surveillance of bacterial meningitis in the country of Georgia, 2006-2010. *J Community Health*. Aug 2013;38(4):724-6. doi:10.1007/s10900-013-9670-4
302. Ergaz Z, Benenson S, Cohen MJ, Braunstein R, Bar-Oz B. No change in antibiotic susceptibility patterns in the neonatal ICU over two decades. *Pediatr Crit Care Med*. Feb 2013;14(2):164-70. doi:10.1097/PCC.0b013e31824fbc19
303. Kavuncuoglu S, Gursoy S, Turel O, Aldemir EY, Hosaf E. Neonatal bacterial meningitis in Turkey: epidemiology, risk factors, and prognosis. *J Infect Dev Ctries*. Feb 15 2013;7(2):73-81. doi:10.3855/jidc.2652
304. Khowaja AR, Mohiuddin S, Cohen AL, et al. Mortality and neurodevelopmental outcomes of acute bacterial meningitis in children aged <5 years in Pakistan. *J Pediatr*. Jul 2013;163(1 Suppl):S86-S91 e1. doi:10.1016/j.jpeds.2013.03.035
305. Mahmoudi S, Zandi H, Pourakbari B, Ashtiani MT, Mamishi S. Acute bacterial meningitis among children admitted into an Iranian referral children's hospital. *Jpn J Infect Dis*. 2013-1-1 2013;66(6):503-6. doi:10.7883/yoken.66.503
306. Porobic-Jahic H, Piljic D, Jahic R, Ahmetagic S, Numanovic F. Etiology of bacterial meningitis in children in Tuzla Canton. *Med Arch*. 2013;67(1):13-6. doi:10.5455/medarh.2013.67.13-16
307. Scott S, Altanseseg D, Sodbayer D, et al. Impact of Haemophilus influenzae Type b conjugate vaccine in Mongolia: prospective population-based surveillance, 2002-2010. *J Pediatr*. Jul 2013;163(1 Suppl):S8-S11. doi:10.1016/j.jpeds.2013.03.024
308. Snaebjarnardottir K, Erlendsdottir H, Reynisson IK, et al. Bacterial meningitis in children in Iceland, 1975-2010: a nationwide epidemiological study. *Scand J Infect Dis*. Nov 2013;45(11):819-24. doi:10.3109/00365548.2013.817680
309. Teleb N, Pilishvili T, Van Beneden C, et al. Bacterial meningitis surveillance in the Eastern Mediterranean region, 2005-2010: successes and challenges of a regional network. *J Pediatr*. Jul 2013;163(1 Suppl):S25-31. doi:10.1016/j.jpeds.2013.03.027
310. Banajeh SM, Ashoor O, Al-Magramy AS. Childhood very severe pneumonia and meningitis-related hospitalization and death in Yemen, before and after introduction of H. influenzae type b (Hib) vaccine. *East Mediterr Health J*. Jul 8 2014;20(7):431-41.
311. Bodilsen J, Dalager-Pedersen M, Schonheyder HC, Nielsen H. Dexamethasone treatment and prognostic factors in community-acquired bacterial meningitis: a Danish retrospective population-based cohort study. *Scand J Infect Dis*. Jun 2014;46(6):418-25. doi:10.3109/00365548.2014.887223
312. Levy C, Varon E, Taha MK, Bechet S, Bonacorsi S, Cohen R, Bingen E. [Changes in bacterial meningitis in French children resulting from vaccination]. *Arch Pediatr*. Jul 2014;21(7):736-44. Evolution des meningites bacteriennes de l'enfant en France sous l'effet des vaccinations. doi:10.1016/j.arcped.2014.04.025
313. Molyneux EM, Kawaza K, Phiri A, et al. Glycerol and acetaminophen as adjuvant therapy did not affect the outcome of bacterial meningitis in Malawian children. *Pediatr Infect Dis J*. Feb 2014;33(2):214-6. doi:10.1097/INF.0000000000000122
314. Namani SA, Koci RA, Qehaja-Bucaj E, Ajazaj-Berisha L, Mehmeti M. The epidemiology of bacterial meningitis in Kosovo. *J Infect Dev Ctries*. Jul 14 2014;8(7):823-30. doi:10.3855/jidc.3553
315. Okike IO, Johnson AP, Henderson KL, et al. Incidence, etiology, and outcome of bacterial meningitis in infants aged <90 days in the United kingdom and Republic of Ireland: prospective, enhanced, national population-based surveillance. *Clin Infect Dis*. Nov 15 2014;59(10):e150-7. doi:10.1093/cid/ciu514
316. Thornorethardottir A, Erlendsdottir H, Sigurethardottir B, Harethardottir H, Reynisson IK, Gottfreethsson M, Guethmundsson S. Bacterial meningitis in adults in Iceland, 1995-2010. *Scand J Infect Dis*. May 2014;46(5):354-60. doi:10.3109/00365548.2014.880184
317. Correa-Lima AR, de Barros Miranda-Filho D, Valenca MM, Andrade-Valenca L. Risk Factors for Acute Symptomatic Seizure in Bacterial Meningitis in Children. *J Child Neurol*. Aug 2015;30(9):1182-5. doi:10.1177/0883073814555907

318. Hu R, Gong Y, Wang Y. Relationship of Serum Procalcitonin Levels to Severity and Prognosis in Pediatric Bacterial Meningitis. *Clin Pediatr (Phila)*. Oct 2015;54(12):1141-4. doi:10.1177/0009922815569203
319. Kamoun F, Dowlut MB, Ameur SB, et al. Neonatal purulent meningitis in southern Tunisia: Epidemiology, bacteriology, risk factors and prognosis. *Fetal Pediatr Pathol*. 2015-1-1 2015;34(4):233-40. doi:10.3109/15513815.2015.1051252
320. Lin MC, Chiu NC, Chi H, Ho CS, Huang FY. Evolving trends of neonatal and childhood bacterial meningitis in northern Taiwan. *J Microbiol Immunol Infect*. Jun 2015;48(3):296-301. doi:10.1016/j.jmii.2013.08.012
321. Mora Mora LA, Arco Espinosa ME, Plumet J, Micheli F. [Community acquired bacterial meningitis in patients over 60]. *Medicina (B Aires)*. 2015-1-1 2015;75(6):367-72. Meningitis bacteriana adquirida en la comunidad en mayores de 60 anos.
322. Olson D, Lamb MM, Gaensbauer JT, Todd JK, Halsey NA, Asturias EJ, Guatemala Pediatric Bacterial Surveillance Working G. Risk Factors for Death and Major Morbidity in Guatemalan Children with Acute Bacterial Meningitis. *Pediatr Infect Dis J*. Jul 2015;34(7):724-8. doi:10.1097/INF.0000000000000720
323. Shrestha RG, Tandukar S, Ansari S, et al. Bacterial meningitis in children under 15 years of age in Nepal. *BMC Pediatr*. Aug 19 2015;15:94. doi:10.1186/s12887-015-0416-6
324. Softic I, Tahirovic H, Hasanhodzic M. Neonatal bacterial meningitis: Results from a cross-sectional hospital based study. *Acta Med Acad*. 2015-1-1 2015;44(2):117-23. doi:10.5644/ama2006-124.139
325. Tan J, Kan J, Qiu G, Zhao D, Ren F, Luo Z, Zhang Y. Clinical Prognosis in Neonatal Bacterial Meningitis: The Role of Cerebrospinal Fluid Protein. *PLoS One*. 2015-1-1 2015;10(10):e0141620. doi:10.1371/journal.pone.0141620
326. Bari A, Zeeshan F, Zafar A, Ejaz H, Iftikhar A, Rathore AW. Childhood Acute Bacterial Meningitis: Clinical Spectrum, Bacteriological Profile and Outcome. *J Coll Physicians Surg Pak*. Oct 2016;26(10):822-826. doi:2450
327. Baunbaek-Knudsen G, Solling M, Farre A, Benfield T, Brandt CT. Improved outcome of bacterial meningitis associated with use of corticosteroid treatment. *Infect Dis (Lond)*. Apr 2016;48(4):281-286. doi:10.3109/23744235.2015.1109705
328. Coldiron ME, Salou H, Sidikou F, et al. Case-Fatality Rates and Sequelae Resulting from Neisseria meningitidis Serogroup C Epidemic, Niger, 2015. *Emerg Infect Dis*. Oct 2016;22(10):1827-9. doi:10.3201/eid2210.160731
329. Glimaker M, Brink M, Naucler P, Sjolin J. Betamethasone and dexamethasone in adult community-acquired bacterial meningitis: a quality registry study from 1995 to 2014. *Clin Microbiol Infect*. Sep 2016;22(9):814 e1-814 e7. doi:10.1016/j.cmi.2016.06.019
330. Kambire D, Soeters HM, Ouedraogo-Traore R, et al. Nationwide Trends in Bacterial Meningitis before the Introduction of 13-Valent Pneumococcal Conjugate Vaccine-Burkina Faso, 2011-2013. *PLoS One*. 2016;11(11):e0166384. doi:10.1371/journal.pone.0166384
331. Wee LY, Tanugroho RR, Thoon KC, et al. A 15-year retrospective analysis of prognostic factors in childhood bacterial meningitis. *Acta Paediatr*. Jan 2016;105(1):e22-9. doi:10.1111/apa.13228
332. Gudina EK, Tesfaye M, Adane A, et al. Adjunctive dexamethasone therapy in unconfirmed bacterial meningitis in resource limited settings: is it a risk worth taking? *BMC Neurol*. Aug 26 2016;16(1):153. doi:10.1186/s12883-016-0678-0
333. Hasbun R, Rosenthal N, Balada-Llasat JM, et al. Epidemiology of Meningitis and Encephalitis in the United States, 2011-2014. *Clin Infect Dis*. Aug 1 2017;65(3):359-363. doi:10.1093/cid/cix319
334. Kaburi BB, Kubio C, Kenu E, Nyarko KM, Mahama JY, Sackey SO, Afari EA. Evaluation of the enhanced meningitis surveillance system, Yendi municipality, northern Ghana, 2010-2015. *BMC Infect Dis*. Apr 24 2017;17(1):306. doi:10.1186/s12879-017-2410-0
335. Kafle DR, Subedi M, Thapa M. Outcome of Patients with Meningitis and Encephalitis at Tertiary Care Hospital in Eastern Nepal. *Kathmandu Univ Med J (KUMJ)*. Jan.-Mar. 2017;15(57):40-44.
336. Lien CY, Huang CR, Tsai WC, et al. Epidemiologic trend of adult bacterial meningitis in southern Taiwan (2006-2015). *J Clin Neurosci*. Aug 2017;42:59-65. doi:10.1016/j.jocn.2017.03.017
337. Ouchenir L, Renaud C, Khan S, et al. The Epidemiology, Management, and Outcomes of Bacterial Meningitis in Infants. *Pediatrics*. Jul 2017;140(1)doi:10.1542/peds.2017-0476
338. Park BS, Kim SE, Park SH, et al. Procalcitonin as a potential predicting factor for prognosis in bacterial meningitis. *J Clin Neurosci*. Feb 2017;36:129-133. doi:10.1016/j.jocn.2016.10.005
339. Polkowska A, Toropainen M, Ollgren J, Lyytikainen O, Nuorti JP. Bacterial meningitis in Finland, 1995-2014: a population-based observational study. *BMJ Open*. Jun 6 2017;7(5):e015080. doi:10.1136/bmjopen-2016-015080
340. Sadeq H, Husain EH, Alkoot A, et al. Childhood meningitis in Kuwait in the era of post pneumococcal conjugate vaccination: A multicenter study. *J Infect Public Health*. Nov-Dec 2017;10(6):766-769. doi:10.1016/j.jiph.2016.11.009
341. Wall EC, Mukaka M, Denis B, et al. Goal directed therapy for suspected acute bacterial meningitis in adults and adolescents in sub-Saharan Africa. *PLoS One*. 2017;12(10):e0186687. doi:10.1371/journal.pone.0186687

342. Amare AT, Kebede ZT, Welch HD. Epidemiology of bacterial meningitis in children admitted to Gondar University Hospital in the post pneumococcal vaccine era. *Pan Afr Med J*. 2018;31:193. doi:10.11604/pamj.2018.31.193.10254
343. Jumanne S, Meda J, Hokororo A, Leshabari K. Clinical Predictors of Malaria, Acute Bacterial Meningitis and Treatment Outcomes among Febrile Children Admitted with Altered Mental Status in Northwestern Tanzania. *J Trop Pediatr*. Oct 1 2018;64(5):426-433. doi:10.1093/tropej/fmx090
344. Kumar M, Tripathi S, Kumar H, Singh SN. Predictors of Poor Outcome in Neonates with Pyogenic Meningitis in a Level-Three Neonatal Intensive Care Unit of Developing Country. *J Trop Pediatr*. Aug 1 2018;64(4):297-303. doi:10.1093/tropej/fmx066
345. Brown BL, Fidell A, Ingolia G, Murad E, Beckham JD. Infectious causes and outcomes in patients presenting with cerebral spinal fluid pleocytosis. *J Neurovirol*. Aug 2019;25(4):448-456. doi:10.1007/s13365-019-00739-w
346. De Almeida SM, Barros NC, Petterle R, Nogueira K. Comparison of cerebrospinal fluid lactate with physical, cytological, and other biochemical characteristics as prognostic factors in acute bacterial meningitis. *Arq Neuropsiquiatr*. Dec 2019;77(12):871-880. doi:10.1590/0004-282X20190185
347. El-Naggar W, Afifi J, McMillan D, Toye J, Ting J, Yoon EW, Shah PS. Epidemiology of Meningitis in Canadian Neonatal Intensive Care Units. *Pediatr Infect Dis J*. May 2019;38(5):476-480. doi:10.1097/inf.0000000000002247
348. Fuentes-Antras J, Ramirez-Torres M, Osorio-Martinez E, Lorente M, Lorenzo-Almoros A, Lorenzo O, Gorgolas M. Acute Community-Acquired Bacterial Meningitis: Update on Clinical Presentation and Prognostic factors. *New Microbiol*. Apr 2019;41(4):81-87.
349. Haydar SM, Hallit SR, Hallit RR, Salameh PR, Faddoul LJ, Chahine BA, Malaeb DN. Adherence to international guidelines for the treatment of meningitis infections in Lebanon. *Saudi Med J*. Mar 2019;40(3):260-265. doi:10.15537/smj.2019.3.23965
350. Larsen F, Brandt CT, Larsen L, et al. Risk factors and prognosis of seizures in adults with community-acquired bacterial meningitis in Denmark: observational cohort studies. *BMJ Open*. Jul 1 2019;9(7):e030263. doi:10.1136/bmjopen-2019-030263
351. Mwenda JM, Soda E, Weldegebriel G, et al. Pediatric Bacterial Meningitis Surveillance in the World Health Organization African Region Using the Invasive Bacterial Vaccine-Preventable Disease Surveillance Network, 2011-2016. *Clin Infect Dis*. Sep 5 2019;69(Suppl 2):S49-S57. doi:10.1093/cid/ciz472
352. Pruitt CM, Neuman MI, Shah SS, et al. Factors Associated with Adverse Outcomes among Febrile Young Infants with Invasive Bacterial Infections. *J Pediatr*. Jan 2019;204:177-182 e1. doi:10.1016/j.jpeds.2018.08.066
353. Sonko MA, Dube FS, Okoi CB, et al. Changes in the Molecular Epidemiology of Pediatric Bacterial Meningitis in Senegal After Pneumococcal Conjugate Vaccine Introduction. *Clin Infect Dis*. Sep 5 2019;69(Suppl 2):S156-S163. doi:10.1093/cid/ciz517
354. Tagbo BN, Bancroft RE, Fajolu I, et al. Pediatric Bacterial Meningitis Surveillance in Nigeria From 2010 to 2016, Prior to and During the Phased Introduction of the 10-Valent Pneumococcal Conjugate Vaccine. *Clin Infect Dis*. Sep 5 2019;69(Suppl 2):S81-S88. doi:10.1093/cid/ciz474
355. Tsolenyanu E, Bancroft RE, Sesay AK, et al. Etiology of Pediatric Bacterial Meningitis Pre- and Post-PCV13 Introduction Among Children Under 5 Years Old in Lome, Togo. *Clin Infect Dis*. Sep 5 2019;69(Suppl 2):S97-S104. doi:10.1093/cid/ciz473
356. Huang YH, Yan JH, Kuo KC, Wu WT, Su CM, Chiu IM. Early antibiotics use in young infants with invasive bacterial infection visiting emergency department, a single medical center's experience. *Pediatr Neonatol*. Apr 2020;61(2):155-159. doi:10.1016/j.pedneo.2019.08.003
357. Johansson Kostenniemi U, Karlsson L, Silfverdal SA, Mehle C. MeningiSSS: A New Predictive Score to Support Decision on Invasive Procedures to Monitor or Manage the Intracerebral Pressure in Children with Bacterial Meningitis. *Neurocrit Care*. Apr 2020;32(2):586-595. doi:10.1007/s12028-019-00792-7
358. Liu G, He S, Zhu X, Li Z. Early onset neonatal bacterial meningitis in term infants: the clinical features, perinatal conditions, and in-hospital outcomes: A single center retrospective analysis. *Medicine (Baltimore)*. Oct 16 2020;99(42):e22748. doi:10.1097/MD.0000000000022748
359. Loutfi A, M ELH, Jayche S, et al. Epidemiological, Cytochemical and Bacteriological Profile of Meningitis among Adults and Children in North West of Morocco. *Pak J Biol Sci*. Jan 2020;23(7):891-897. doi:10.3923/pjbs.2020.891.897
360. Matulyte E, Kiveryte S, Paulauskiene R, Liukpetryte E, Vaikutyte R, Matulionyte R. Retrospective analysis of the etiology, clinical characteristics and outcomes of community-acquired bacterial meningitis in the University Infectious Diseases Centre in Lithuania. *BMC Infect Dis*. Oct 7 2020;20(1):733. doi:10.1186/s12879-020-05462-0
361. Peros T, van Schuppen J, Bohte A, Hodiamont C, Aronica E, de Haan T. Neonatal bacterial meningitis versus ventriculitis: a cohort-based overview of clinical characteristics, microbiology and imaging. *Eur J Pediatr*. Dec 2020;179(12):1969-1977. doi:10.1007/s00431-020-03723-3

362. Pomar V, de Benito N, Mauri A, Coll P, Gurgui M, Domingo P. Characteristics and outcome of spontaneous bacterial meningitis in patients with diabetes mellitus. *BMC Infect Dis*. Apr 20 2020;20(1):292. doi:10.1186/s12879-020-05023-5
363. Tubiana S, Varon E, Biron C, et al. Community-acquired bacterial meningitis in adults: in-hospital prognosis, long-term disability and determinants of outcome in a multicentre prospective cohort. *Clin Microbiol Infect*. Sep 2020;26(9):1192-1200. doi:10.1016/j.cmi.2019.12.020
364. Adil SM, Hodges SE, Charalambous LT, et al. Paediatric bacterial meningitis in the USA: outcomes and healthcare resource utilization of nosocomial versus community-acquired infection. *J Med Microbiol*. Jan 2021;70(1)doi:10.1099/jmm.0.001276
365. Aimbudlop K, Bruminhent J, Kiertiburanakul S. Infectious causes of acute meningitis among Thai adults in a university hospital. *J Infect Chemother*. Feb 2021;27(2):198-204. doi:10.1016/j.jiac.2020.09.002
366. Bumburidi Y, Utepbergenova G, Yerezhepov B, et al. Etiology of acute meningitis and encephalitis from hospital-based surveillance in South Kazakhstan oblast, February 2017-January 2018. *PLoS One*. 2021;16(5):e0251494. doi:10.1371/journal.pone.0251494
367. Kumar D, Pannu AK, Dhibar DP, Singh R, Kumari S. The epidemiology and clinical spectrum of infections of the central nervous system in adults in north India. *Trop Doct*. Jan 2021;51(1):48-57. doi:10.1177/0049475520959905
368. Pelkonen T, Urtti S, Cardoso O, Kyaw MH, Roine I, Peltola H. Risk factors for death in suspected severe bacterial infection in infants aged <90 days in Luanda, Angola. *Int J Infect Dis*. May 2021;106:223-227. doi:10.1016/j.ijid.2021.03.070
369. Salmanov AG, Ishchak OM, Dobarin Scapital A C, Susidko OM, Mosendz OV, Korniyenko SM, Voloshyn OA. Perinatal Infections in Ukraine: Results of a Multicenter Study. *Wiad Lek*. 2021;74(9 cz 1):2025-2032.
370. Savonius O, Rugemalira E, Roine I, Cruzeiro ML, Peltola H, Pelkonen T. Extended Continuous beta-Lactam Infusion With Oral Acetaminophen in Childhood Bacterial Meningitis: A Randomized, Double-blind Clinical Trial. *Clin Infect Dis*. May 18 2021;72(10):1738-1744. doi:10.1093/cid/ciaa341
371. Sunwoo JS, Shin HR, Lee HS, et al. A hospital-based study on etiology and prognosis of bacterial meningitis in adults. *Sci Rep*. Mar 16 2021;11(1):6028. doi:10.1038/s41598-021-85382-4
372. Wong CH, Duque JR, Wong JSC, et al. Epidemiology and Trends of Infective Meningitis in Neonates and Infants Less than 3 Months Old in Hong Kong. *Int J Infect Dis*. Oct 2021;111:288-294. doi:10.1016/j.ijid.2021.06.025
373. Feibush JS, Murphy EJ, Lubart A. Pneumococcal meningitis in adults. *Ann Intern Med*. Jul 1952;37(1):65-74. doi:10.7326/0003-4819-37-1-65
374. Pengelly CD. Pneumococcal meningitis; a short survey of 78 patients in the Bristol clinical area. *Br Med J*. Apr 9 1955;1(4918):870-4. doi:10.1136/bmj.1.4918.870
375. Wehrle PF, Mathies AW, Leedom JM, Ivler D. Bacterial meningitis. *Ann N Y Acad Sci*. Sep 27 1967;145(2):488-98. doi:10.1111/j.1749-6632.1967.tb50249.x
376. Pirame Y, Patacq-Croutzet J, Nguyen Trung L, Dujeu G, Héraut L, Sawadogo R. [Commentary apropos of 126 cases of pneumococcal meningitis observed at Wagadugu Hospital (Upper Volta)]. *Bull Soc Pathol Exot Filiales*. 1968;61(2):154-62. Commentaires à propos de 126 cas de méningite à pneumocoques observés à l'hôpital de Quagadougou (Haute-Volta).
377. Haddock DR. Forty-seven cases of pyogenic meningitis in adults in Korle Bu Hospital, Accra. *Ghana Med J*. Mar 1971;10(1):3-8.
378. Levin S, Nelson KE, Spies HW, Lepper MH. Pneumococcal meningitis: the problem of the unseen cerebrospinal fluid leak. *Am J Med Sci*. Oct 1972;264(4):319-27. doi:10.1097/00000441-197210000-00010
379. Baird DR, Whittle HC, Greenwood BM. Mortality from pneumococcal meningitis. *Lancet*. Dec 18 1976;2(7999):1344-6. doi:10.1016/s0140-6736(76)91985-1
380. Laxer RM, Marks MI. Pneumococcal meningitis in children. *Am J Dis Child*. Aug 1977;131(8):850-3. doi:10.1001/archpedi.1977.02120210028004
381. Marr JS, Galaid EI, Clark J. Mortality from pneumococcal meningitis. *Lancet*. Jan 15 1977;1(8003):147.
382. Rees P. Pneumococcal meningitis. *Lancet*. Feb 5 1977;1(8006):307. doi:10.1016/s0140-6736(77)91852-9
383. Bademosi O, Osuntokun BO. Prednisolone in the treatment of pneumococcal meningitis. *Trop Geogr Med*. Mar 1979;31(1):53-6.
384. Cadoz M, Denis F, Chiron JP, Sow A, Diop Mar I. [The prognosis and treatment of pneumococcal meningitis in Africa. 402 cases (author's transl)]. *Nouv Presse Med*. Feb 17 1979;8(8):573-6. Pronostic et traitement des meningites a pneumocoque en Afrique. 402 observations.
385. Jacobs NM, Lerdkachornsuk S, Metzger WI. Pneumococcal bacteremia in infants and children: a ten-year experience at the Cook County Hospital with special reference to the pneumococcal serotypes isolated. *Pediatrics*. Sep 1979;64(3):296-300.
386. Ostroy PR. Bacterial meningitis in Washington state. *West J Med*. Oct 1979;131(4):339-43.

387. Berkowitz FE. Pneumococcal bacteraemia--a study of 75 black children. *Ann Trop Paediatr*. Dec 1981;1(4):229-35. doi:10.1080/02724936.1981.11748094
388. Gallais H, De Mel D, Kadio A, Raoult D, Morvan D. [Diagnostic and therapeutic aspects of pneumococcal meningitis in Africa (apropos of 119 cases)]. *Med Trop (Mars)*. Mar-Apr 1983;43(2):163-9. Aspects diagnostiques et therapeutiques de la meningite a pneumocoque en Afrique (a propos de 119 observations).
389. Nottidge VA. Pneumococcal meningitis in sickle cell disease in childhood. *Am J Dis Child*. Jan 1983;137(1):29-31. doi:10.1001/archpedi.1983.02140270025008
390. Pedersen FK, Henrichsen J. Pneumococcal meningitis and bacteraemia in Danish children 1969-1978. Serotypes, incidence and outcome. *Acta Pathol Microbiol Immunol Scand B*. Apr 1983;91(2):129-34. doi:10.1111/j.1699-0463.1983.tb00021.x
391. Coulehan JL, Michaels RH, Hallowell C, Schults R, Welty TK, Kuo JS. Epidemiology of Haemophilus influenzae type B disease among Navajo Indians. *Public Health Rep*. Jul-Aug 1984;99(4):404-9.
392. Burman LA, Norrby R, Trollfors B. Invasive pneumococcal infections: incidence, predisposing factors, and prognosis. *Rev Infect Dis*. Mar-Apr 1985;7(2):133-42. doi:10.1093/clinids/7.2.133
393. Chan-Lui WY. Pneumococcal meningitis in infants. *Brain Dev*. 1985;7(6):590-8. doi:10.1016/s0387-7604(85)80007-3
394. Gransden WR, Eykyn SJ, Phillips I. Pneumococcal bacteraemia: 325 episodes diagnosed at St Thomas's Hospital. *Br Med J (Clin Res Ed)*. Feb 16 1985;290(6467):505-8. doi:10.1136/bmj.290.6467.505
395. Lecour H, Seara A, Miranda AM, Cordeiro J. Cefotaxime in pneumococcal meningitis. *Infection*. 1985;13 Suppl 1:S73-5. doi:10.1007/BF01644223
396. Gray BM, Dillon HC, Jr. Clinical and epidemiologic studies of pneumococcal infection in children. *Pediatr Infect Dis*. Mar-Apr 1986;5(2):201-7. doi:10.1097/00006454-198603000-00009
397. Bruyn GA, Kremer HP, de Marie S, Padberg GW, Hermans J, van Furth R. Clinical evaluation of pneumococcal meningitis in adults over a twelve-year period. *Eur J Clin Microbiol Infect Dis*. Aug 1989;8(8):695-700. doi:10.1007/BF01963754
398. Kennedy WA, Hoyt MJ, McCracken GH, Jr. The role of corticosteroid therapy in children with pneumococcal meningitis. *Am J Dis Child*. Dec 1991;145(12):1374-8. doi:10.1001/archpedi.1991.02160120042016
399. Viladrich PF, Gudiol F, Linares J, Pallares R, Sabate I, Rufi G, Ariza J. Evaluation of vancomycin for therapy of adult pneumococcal meningitis. *Antimicrob Agents Chemother*. Dec 1991;35(12):2467-72. doi:10.1128/AAC.35.12.2467
400. Barsic B, Lisic M, Himbele J, Beus I, Marton E, Balentic V, Palic J. Pneumococcal meningitis in the elderly. *Neurol Croat*. 1992;41(3):131-9.
401. Davidson M, Parkinson AJ, Bulkow LR, Fitzgerald MA, Peters HV, Parks DJ. The epidemiology of invasive pneumococcal disease in Alaska, 1986-1990--ethnic differences and opportunities for prevention. *J Infect Dis*. Aug 1994;170(2):368-76. doi:10.1093/infdis/170.2.368
402. Kirkpatrick B, Reeves DS, MacGowan AP. A review of the clinical presentation, laboratory features, antimicrobial therapy and outcome of 77 episodes of pneumococcal meningitis occurring in children and adults. *J Infect*. Sep 1994;29(2):171-82. doi:10.1016/s0163-4453(94)90698-x
403. Kragsbjerg P, Kallman J, Olcen P. Pneumococcal meningitis in adults. *Scand J Infect Dis*. 1994;26(6):659-66. doi:10.3109/00365549409008633
404. Voss L, Lennon D, Okesene-Gafa K, Ameratunga S, Martin D. Invasive pneumococcal disease in a pediatric population, Auckland, New Zealand. *Pediatr Infect Dis J*. Oct 1994;13(10):873-8. doi:10.1097/00006454-199410000-00005
405. Davis CW, McIntyre PB. Invasive pneumococcal infection in children, 1981-92: a hospital-based study. *J Paediatr Child Health*. Aug 1995;31(4):317-22. doi:10.1111/j.1440-1754.1995.tb00819.x
406. Kanra GY, Ozen H, Secmeer G, Ceyhan M, Ecevit Z, Belgin E. Beneficial effects of dexamethasone in children with pneumococcal meningitis. *Pediatr Infect Dis J*. Jun 1995;14(6):490-4. doi:10.1097/00006454-199506000-00005
407. Kornelisse RF, Westerbeek CM, Spoor AB, van der Heijde B, Spanjaard L, Neijens HJ, de Groot R. Pneumococcal meningitis in children: prognostic indicators and outcome. *Clin Infect Dis*. Dec 1995;21(6):1390-7. doi:10.1093/clinids/21.6.1390
408. Urwin G, Yuan MF, Hall LM, Brown K, Efstratiou A, Feldman RA. Pneumococcal meningitis in the North East Thames Region UK: epidemiology and molecular analysis of isolates. *Epidemiol Infect*. Aug 1996;117(1):95-102. doi:10.1017/s0950268800001175
409. Arditi M, Mason EO, Jr., Bradley JS, et al. Three-year multicenter surveillance of pneumococcal meningitis in children: clinical characteristics, and outcome related to penicillin susceptibility and dexamethasone use. *Pediatrics*. Nov 1998;102(5):1087-97. doi:10.1542/peds.102.5.1087
410. Venetz I, Schopfer K, Muhlemann K. Paediatric, invasive pneumococcal disease in Switzerland, 1985-1994. Swiss Pneumococcal Study Group. *Int J Epidemiol*. Dec 1998;27(6):1101-4. doi:10.1093/ije/27.6.1101

411. Gomez-Barreto D, Calderon-Jaimes E, Rodriguez RS, Espinosa de los Monteros LE, Juarez M. [Clinico-microbiological characteristics of meningitis caused by penicillin-resistant Streptococcus pneumoniae]]. *Salud Publica Mex*. Sep-Oct 1999;41(5):397-404. Caracteristicas clinico-microbiologicas de la meningitis por Streptococcus pneumoniae resistente a la penicilina.
412. Muhe L, Klugman KP. Pneumococcal and Haemophilus influenzae meningitis in a children's hospital in Ethiopia: serotypes and susceptibility patterns. *Trop Med Int Health*. Jun 1999;4(6):421-7. doi:10.1046/j.1365-3156.1999.00417.x
413. Schneider O, Michel U, Zysk G, Dubuis O, Nau R. Clinical outcome in pneumococcal meningitis correlates with CSF lipoteichoic acid concentrations. *Neurology*. Oct 22 1999;53(7):1584-7. doi:10.1212/wnl.53.7.1584
414. Stanek RJ, Mufson MA. A 20-year epidemiological study of pneumococcal meningitis. *Clin Infect Dis*. Jun 1999;28(6):1265-72. doi:10.1086/514777
415. Fiore AE, Moroney JF, Farley MM, et al. Clinical outcomes of meningitis caused by Streptococcus pneumoniae in the era of antibiotic resistance. *Clin Infect Dis*. Jan 2000;30(1):71-7. doi:10.1086/313606
416. Goetghebuer T, West TE, Wermenbol V, et al. Outcome of meningitis caused by Streptococcus pneumoniae and Haemophilus influenzae type b in children in The Gambia. *Trop Med Int Health*. Mar 2000;5(3):207-13. doi:10.1046/j.1365-3156.2000.00535.x
417. Mencia Bartolome S, Casado Flores J, Marin Barba C, Gonzalez-Vicent M, Ruiz Lopez MJ. [Pneumococcal meningitis in children. Review of 28 cases]. *An Esp Pediatr*. Aug 2000;53(2):94-9. Meningitis neumococica en la infancia. Revision de 28 casos.
418. Soult Rubio JA, Rangel Pineda C, Munoz Saez M, Parrilla Parrilla JS, Diaz Fernandez F, Lopez Castilla JD, Tovaruela Santos A. [Pneumococcal meningitis: epidemiological, clinical and bacteriological characteristics]. *An Esp Pediatr*. Oct 2001;55(4):315-20. Meningitis neumococica: caracteristicas epidemiologicas, clinicas y bacteriologicas.
419. Chomarat M, Fredenucci I, Barbe G, et al. [Rhone-Alpes observatory of Streptococcus pneumoniae in 1999: 35 cases of meningitis]. *Pathol Biol (Paris)*. Dec 2002;50(10):595-8. Observatoire Rhone-Alpes du pneumocoque en 1999: 35 cas de meningites. doi:10.1016/s0369-8114(02)00356-5
420. Ma JS, Chen PY, Mak SC, Chi CS, Lau YJ. Clinical outcome of invasive pneumococcal infection in children: a 10-year retrospective analysis. *J Microbiol Immunol Infect*. Mar 2002;35(1):23-8.
421. Ulloa-Gutierrez R, Avila-Aguero ML, Herrera ML, Herrera JF, Arguedas A. Invasive pneumococcal disease in Costa Rican children: a seven year survey. *Pediatr Infect Dis J*. Dec 2003;22(12):1069-74. doi:10.1097/01.inf.0000101475.45195.b1
422. Ispahani P, Slack RC, Donald FE, Weston VC, Rutter N. Twenty year surveillance of invasive pneumococcal disease in Nottingham: serogroups responsible and implications for immunisation. *Arch Dis Child*. Aug 2004;89(8):757-62. doi:10.1136/adc.2003.036921
423. Buckingham SC, McCullers JA, Lujan-Zilbermann J, Knapp KM, Orman KL, English BK. Early vancomycin therapy and adverse outcomes in children with pneumococcal meningitis. *Pediatrics*. May 2006;117(5):1688-94. doi:10.1542/peds.2005-2282
424. Lovera D, Arbo A. Risk factors for mortality in Paraguayan children with pneumococcal bacterial meningitis. *Trop Med Int Health*. Dec 2005;10(12):1235-41. doi:10.1111/j.1365-3156.2005.01513.x
425. McIntyre PB, Macintyre CR, Gilmour R, Wang H. A population based study of the impact of corticosteroid therapy and delayed diagnosis on the outcome of childhood pneumococcal meningitis. *Arch Dis Child*. Apr 2005;90(4):391-6. doi:10.1136/adc.2003.037523
426. Ostergaard C, Konradsen HB, Samuelsson S. Clinical presentation and prognostic factors of Streptococcus pneumoniae meningitis according to the focus of infection. *BMC Infect Dis*. Oct 27 2005;5:93. doi:10.1186/1471-2334-5-93
427. Yaro S, Lourd M, Traore Y, et al. Epidemiological and molecular characteristics of a highly lethal pneumococcal meningitis epidemic in Burkina Faso. *Clin Infect Dis*. Sep 15 2006;43(6):693-700. doi:10.1086/506940
428. Carrol ED, Guiver M, Nkhoma S, et al. High pneumococcal DNA loads are associated with mortality in Malawian children with invasive pneumococcal disease. *Pediatr Infect Dis J*. May 2007;26(5):416-22. doi:10.1097/01.inf.0000260253.22994.61
429. Holliman RE, Liddy H, Johnson JD, Adjei O. Epidemiology of invasive pneumococcal disease in Kumasi, Ghana. *Trans R Soc Trop Med Hyg*. Apr 2007;101(4):405-13. doi:10.1016/j.trstmh.2006.08.014
430. Kallel H, Maaloul I, Mahjoubi F, et al. [Prognostic factors of pneumococcal meningitis. Retrospective study of 31 cases]. *Tunis Med*. Aug 2007;85(8):692-6. Facteurs de pronostic des meningites a pneumocoque. Etude retrospective de 31 cas.
431. Pagliano P, Fusco U, Attanasio V, Rossi M, Pantosti A, Conte M, Faella FS. Pneumococcal meningitis in childhood: a longitudinal prospective study. *FEMS Immunol Med Microbiol*. Dec 2007;51(3):488-95. doi:10.1111/j.1574-695X.2007.00324.x

432. Thabet F, Tilouche S, Tabarki B, et al. [Pneumococcal meningitis mortality in children. Prognostic factors in a series of 73 cases]. *Arch Pediatr*. Apr 2007;14(4):334-7. Mortalite par meningites a pneumocoque chez l'enfant. Facteurs pronostiques a propos d'une serie de 73 observations. doi:10.1016/j.arcped.2006.11.012
433. Chong CY, Koh-Cheng T, Yee-Hui M, Nancy TW. Invasive pneumococcal disease in Singapore children. *Vaccine*. Jun 25 2008;26(27-28):3427-31. doi:10.1016/j.vaccine.2008.04.035
434. Ishiwada N, Kurosaki T, Terashima I, Kohno Y. The incidence of pediatric invasive pneumococcal disease in Chiba prefecture, Japan (2003-2005). *J Infect*. Dec 2008;57(6):455-8. doi:10.1016/j.jinf.2008.09.029
435. Manga NM, Ndour CT, Diop SA, et al. [Adult purulent meningitis caused by Streptococcus pneumoniae in Dakar, Senegal]. *Med Trop (Mars)*. Dec 2008;68(6):625-8. Les meningites purulentes a Streptococcus pneumoniae de l'adulte a Dakar.
436. Rajasingham CR, Bonsu BK, Chapman JI, Cohen DM, Barson WJ. Serious neurologic sequelae in cases of meningitis arising from infection by conjugate vaccine-related and nonvaccine-related serogroups of Streptococcus pneumoniae. *Pediatr Infect Dis J*. Sep 2008;27(9):771-5. doi:10.1097/INF.0b013e3181710976
437. Tsai MH, Chen SH, Hsu CY, et al. Pneumococcal meningitis in Taiwanese children: emphasis on clinical outcomes and prognostic factors. *J Trop Pediatr*. Dec 2008;54(6):390-4. doi:10.1093/tropej/fmn046
438. Falade AG, Lagunju IA, Bakare RA, Odekanmi AA, Adegbola RA. Invasive pneumococcal disease in children aged <5 years admitted to 3 urban hospitals in Ibadan, Nigeria. *Clin Infect Dis*. Mar 1 2009;48 Suppl 2:S190-6. doi:10.1086/596500
439. Gil Prieto R, San Roman Montero J, Gomez Alejandre C, Alvaro Meca LA, Rivero A, Gil de Miguel A. Epidemiology of pneumococcal meningitis hospitalizations in pediatric population in Spain (1998-2006). *Vaccine*. May 5 2009;27(20):2669-73. doi:10.1016/j.vaccine.2009.02.063
440. Kisakye A, Makumbi I, Nansera D, et al. Surveillance for Streptococcus pneumoniae meningitis in children aged <5 years: implications for immunization in Uganda. *Clin Infect Dis*. Mar 1 2009;48 Suppl 2:S153-61. doi:10.1086/596495
441. Trotman H, Olugbuyi O, Barton M, McGregor D, Thomas S. Pneumococcal meningitis in Jamaican children. *West Indian Med J*. Dec 2009;58(6):585-8.
442. Brouwer MC, Heckenberg SG, de Gans J, Spanjaard L, Reitsma JB, van de Beek D. Nationwide implementation of adjunctive dexamethasone therapy for pneumococcal meningitis. *Neurology*. Oct 26 2010;75(17):1533-9. doi:10.1212/WNL.0b013e3181f96297
443. Memish ZA, El-Saed A, Al-Otaibi B, Shaalan MA, Alola SA, Thaqafi AO. Epidemiology of invasive pneumococcal infection in children aged five years and under in Saudi Arabia: a five-year retrospective surveillance study. *Int J Infect Dis*. Aug 2010;14(8):e708-12. doi:10.1016/j.ijid.2010.02.2242
444. Ochoa TJ, Egoavil M, Castillo ME, et al. Invasive pneumococcal diseases among hospitalized children in Lima, Peru. *Rev Panam Salud Publica*. Aug 2010;28(2):121-7. doi:10.1590/s1020-49892010000800008
445. Gouveia EL, Reis JN, Flannery B, et al. Clinical outcome of pneumococcal meningitis during the emergence of pencillin-resistant Streptococcus pneumoniae: an observational study. *BMC Infect Dis*. Nov 21 2011;11:323. doi:10.1186/1471-2334-11-323
446. Lucey JM, Gavin P, Cafferkey M, Butler KM. Pneumococcal meningitis: clinical outcomes in a pre-vaccine era at a Dublin paediatric hospital, 1999-2007. *Ir J Med Sci*. Mar 2011;180(1):47-50. doi:10.1007/s11845-010-0620-1
447. Novaes HM, Sartori AM, Soarez PC. Hospitalization rates for pneumococcal disease in Brazil, 2004 - 2006. *Rev Saude Publica*. Jun 2011;45(3):539-47. doi:10.1590/s0034-89102011005000028
448. Nyasulu P, Cohen C, De Gouveia L, et al. Increased risk of death in human immunodeficiency virus-infected children with pneumococcal meningitis in South Africa, 2003-2005. *Pediatr Infect Dis J*. Dec 2011;30(12):1075-80. doi:10.1097/INF.0b013e31822cca05
449. Choi SH, Chung JW, Kim BN, et al. Clinical implication of extended-spectrum cephalosporin nonsusceptibility in Streptococcus pneumoniae meningitis. *Eur J Clin Microbiol Infect Dis*. Nov 2012;31(11):3029-34. doi:10.1007/s10096-012-1657-z
450. Stockmann C, Ampofo K, Byington CL, et al. Pneumococcal meningitis in children: epidemiology, serotypes, and outcomes from 1997-2010 in Utah. *Pediatrics*. Sep 2013;132(3):421-8. doi:10.1542/peds.2013-0621
451. Thomas K, Mukkai Kesavan L, Veeraraghavan B, et al. Invasive pneumococcal disease associated with high case fatality in India. *J Clin Epidemiol*. Jan 2013;66(1):36-43. doi:10.1016/j.jclinepi.2012.04.006
452. Tsai HY, Lauderdale TL, Wang JT, et al. Updated antibiotic resistance and clinical spectrum of infections caused by Streptococcus pneumoniae in Taiwan: Emphasis on risk factors for penicillin nonsusceptibilities. *J Microbiol Immunol Infect*. Oct 2013;46(5):345-51. doi:10.1016/j.jmii.2012.07.012
453. Berberian G, Perez MG, Epelbaum C, Ceinos Mdel C, Lopardo H, Rosanova MT. Pneumococcal meningitis: a 12 year experience in a children's hospital prior to the universal immunization with a conjugate vaccine. *Arch Argent Pediatr*. Aug 2014;112(4):332-6. doi:10.5546/aap.2014.eng.332

454. Browall S, Backhaus E, Naucler P, et al. Clinical manifestations of invasive pneumococcal disease by vaccine and non-vaccine types. *Eur Respir J*. Dec 2014;44(6):1646-57. doi:10.1183/09031936.00080814
455. Erdem H, Elaldi N, Öztoprak N, et al. Mortality indicators in pneumococcal meningitis: therapeutic implications. *Int J Infect Dis*. Feb 2014;19:13-9. doi:10.1016/j.ijid.2013.09.012
456. Levy C, Varon E, Picard C, Bechet S, Martinot A, Bonacorsi S, Cohen R. Trends of pneumococcal meningitis in children after introduction of the 13-valent pneumococcal conjugate vaccine in France. *Pediatr Infect Dis J*. Dec 2014;33(12):1216-21. doi:10.1097/INF.0000000000000451
457. Paulke-Korinek M, Kollaritsch H, Kundi M, et al. Characteristics of invasive pneumococcal disease in hospitalized children in Austria. *Eur J Pediatr*. Apr 2014;173(4):469-76. doi:10.1007/s00431-013-2193-2
458. von Mollendorf C, Cohen C, de Gouveia L, et al. Factors associated with ceftriaxone nonsusceptibility of Streptococcus pneumoniae: analysis of South African national surveillance data, 2003 to 2010. *Antimicrob Agents Chemother*. Jun 2014;58(6):3293-305. doi:10.1128/aac.02580-13
459. Casez P, Fauconnier J, Jorgensen L, et al. Longitudinal DRG-based survey of all-cause and pneumococcal pneumonia and meningitis for inpatients in France (2005-2010). *Med Mal Infect*. Nov-Dec 2015;45(11-12):446-55. doi:10.1016/j.medmal.2015.10.004
460. Grando IM, Moraes C, Flannery B, Ramalho WM, Horta MA, Pinho DL, Nascimento GL. Impact of 10-valent pneumococcal conjugate vaccine on pneumococcal meningitis in children up to two years of age in Brazil. *Cad Saude Publica*. Feb 2015;31(2):276-84. doi:10.1590/0102-311x00169913
461. Jung J, Park KH, Park SY, et al. Comparison of the clinical characteristics and outcomes of Klebsiella pneumoniae and Streptococcus pneumoniae meningitis. *Diagn Microbiol Infect Dis*. May 2015;82(1):87-91. doi:10.1016/j.diagmicrobio.2015.02.006
462. Navarro-Torne A, Dias JG, Hruba F, Lopalco PL, Pastore-Celentano L, Gauci AJ, Invasive Pneumococcal Disease Study G. Risk factors for death from invasive pneumococcal disease, Europe, 2010. *Emerg Infect Dis*. Mar 2015;21(3):417-25. doi:10.3201/eid2103.140634
463. Buchholz G, Koedel U, Pfister HW, Kastenbauer S, Klein M. Dramatic reduction of mortality in pneumococcal meningitis. *Crit Care*. Oct 2 2016;20(1):312. doi:10.1186/s13054-016-1498-8
464. Lundbo LF, Harboe ZB, Clausen LN, et al. Genetic Variation in NFKBIE Is Associated With Increased Risk of Pneumococcal Meningitis in Children. *EBioMedicine*. Jan 2016;3:93-99. doi:10.1016/j.ebiom.2015.11.048
465. Rojas JP, Leal AL, Patino J, et al. [Characterization of patients who died of invasive pneumococcal disease in the child population of Bogota, Colombia]. *Rev Chil Pediatr*. Jan-Feb 2016;87(1):48-52. Caracterizacion de pacientes fallecidos por enfermedad neumococcica invasiva en la poblacion infantil de Bogota, Colombia. doi:10.1016/j.rchipe.2015.10.005
466. Saha SK, Hossain B, Islam M, et al. Epidemiology of Invasive Pneumococcal Disease in Bangladeshi Children Before Introduction of Pneumococcal Conjugate Vaccine. *Pediatr Infect Dis J*. Jun 2016;35(6):655-61. doi:10.1097/INF.0000000000001037
467. Webber S, Cooper G, DeMuri G, Wald ER. Pneumococcal Meningitis in the PCV13 Era: A Cluster of Cases With Increased Morbidity and Mortality. *Clin Pediatr (Phila)*. Nov 2016;55(13):1252-1255. doi:10.1177/0009922816629761
468. Lim S, Chung DR, Kim YS, et al. Predictive risk factors for Listeria monocytogenes meningitis compared to pneumococcal meningitis: a multicenter case-control study. *Infection*. Feb 2017;45(1):67-74. doi:10.1007/s15010-016-0939-2
469. Moisi JC, Makawa MS, Tall H, et al. Burden of Pneumococcal Disease in Northern Togo before the Introduction of Pneumococcal Conjugate Vaccine. *PLoS One*. 2017;12(1):e0170412. doi:10.1371/journal.pone.0170412
470. Pirez MC, Mota MI, Giachetto G, et al. Pneumococcal Meningitis Before and After Universal Vaccination With Pneumococcal Conjugate Vaccines 7/13, Impact on Pediatric Hospitalization in Public and Nonpublic Institutions, in Uruguay. *Pediatr Infect Dis J*. Oct 2017;36(10):1000-1001. doi:10.1097/INF.0000000000001671
471. Coldiron ME, Toure O, Frank T, Bouygues N, Grais RF. Outbreak of Pneumococcal Meningitis, Paoua Subprefecture, Central African Republic, 2016-2017. *Emerg Infect Dis*. Sep 2018;24(9):1720-1722. doi:10.3201/eid2409.171058
472. Ben Salah A, El Mhamdi S, Ben Fredj M, et al. Hospital cost of invasive pneumococcal disease in children aged under 15 years old in Tunisia. *East Mediterr Health J*. Dec 29 2019;25(12):861-871. doi:10.26719/emhj.19.036
473. Irfan S, Farooqi J, Kumar H, Zafar A. Antimicrobial sensitivity pattern, demographic findings and risk factors amongst meningitis and non-meningitis invasive Streptococcus pneumoniae at Aga Khan University Hospital Clinical Laboratory, Karachi, Pakistan. *J Pak Med Assoc*. Aug 2019;69(8):1124-1130.
474. Jayaraman R, Varghese R, Kumar JL, et al. Invasive pneumococcal disease in Indian adults: 11 years' experience. *J Microbiol Immunol Infect*. Oct 2019;52(5):736-742. doi:10.1016/j.jmii.2018.03.004
475. Mihret W, Sletbakk Brusletto B, Ovstebo R, et al. Molecular studies of meningococcal and pneumococcal meningitis patients in Ethiopia. *Innate Immun*. Apr 2019;25(3):158-167. doi:10.1177/1753425918806363

476. Oligbu G, Collins S, Djennad A, et al. Effect of Pneumococcal Conjugate Vaccines on Pneumococcal Meningitis, England and Wales, July 1, 2000-June 30, 2016. *Emerg Infect Dis*. Sep 2019;25(9):1708-1718. doi:10.3201/eid2509.180747
477. Tenforde MW, Mokomane M, Leeme TB, et al. Mortality in adult patients with culture-positive and culture-negative meningitis in the Botswana national meningitis survey: a prevalent cohort study. *Lancet Infect Dis*. Jul 2019;19(7):740-749. doi:10.1016/S1473-3099(19)30066-0
478. Blanco BP, Branas P, Yoshioka CRM, Ferronato AE. Pediatric bacterial meningitis and meningococcal disease profile in a Brazilian General Hospital. *Braz J Infect Dis*. Jul-Aug 2020;24(4):337-342. doi:10.1016/j.bjid.2020.06.001
479. Hernstadt H, Cheung A, Hurem D, et al. Changing Epidemiology and Predisposing Factors for Invasive Pneumococcal Disease at Two Australian Tertiary Hospitals. *Pediatr Infect Dis J*. Jan 2020;39(1):1-6. doi:10.1097/INF.0000000000002489
480. Szymanski W, Simon K, Rorat M. Differences in the courses of meningococcal and pneumococcal cerebrospinal meningitis. *Neurol Neurochir Pol*. 2020;54(1):39-46. doi:10.5603/PJNNS.a2020.0002
481. Hanada S, Takata M, Morozumi M, Iwata S, Fujishima S, Ubukata K, Invasive Pneumococcal Diseases Surveillance Study G. Multiple comorbidities increase the risk of death from invasive pneumococcal disease under the age of 65 years. *J Infect Chemother*. Sep 2021;27(9):1311-1318. doi:10.1016/j.jiac.2021.04.018
482. Iwata S, Takata M, Morozumi M, Miyairi I, Matsubara K, Ubukata K, Pneumococcal Meningitis Surveillance Study G. Drastic reduction in pneumococcal meningitis in children owing to the introduction of pneumococcal conjugate vaccines: Longitudinal analysis from 2002 to 2016 in Japan. *J Infect Chemother*. Apr 2021;27(4):604-612. doi:10.1016/j.jiac.2020.11.019
483. Muller A, Schramm DB, Kleynhans J, et al. Cytokine response in cerebrospinal fluid of meningitis patients and outcome associated with pneumococcal serotype. *Sci Rep*. Oct 7 2021;11(1):19920. doi:10.1038/s41598-021-99190-3
484. Polkowska A, Rinta-Kokko H, Toropainen M, Palmu AA, Nuorti JP. Long-term population effects of infant 10-valent pneumococcal conjugate vaccination on pneumococcal meningitis in Finland. *Vaccine*. May 27 2021;39(23):3216-3224. doi:10.1016/j.vaccine.2021.02.030
485. Stevens JP, Lively A, Jerris R, Yildirim I, Lantis P. Recognition and Outcomes of Pneumococcal Meningitis in 2 Tertiary Pediatric Hospitals Since the Introduction of the 13-Valent Pneumococcal Conjugate Vaccine. *Pediatr Emerg Care*. Jan 1 2022;38(1):e354-e359. doi:10.1097/PEC.0000000000002288
486. Petuelli F. [Contribution to meningococcal diseases in childhood]. *Wien Med Wochenschr*. Apr 1 1967;117(13):324-31. Beitrag zu den Meningokokkenerkrankungen im Kindesalter.
487. Vassiliadis P, Kanellakis A, Papadakis J. Sulphadiazine-resistant group A meningococci isolated during the 1968 meningitis epidemic in Greece. *J Hyg (Lond)*. Jun 1969;67(2):279-88. doi:10.1017/s0022172400041681
488. Gendron Y. [Cerebrospinal meningitis in a rural area in Upper Volta. Clinical aspects and results]. *Med Trop (Mars)*. Sep-Oct 1972;32(5):625-30. La meningite cerebrospinale en milieu rural voltaique. Aspects cliniques et resultats.
489. Greenwood BM, Whittle HC. Antigen-negative meningitis due to group A Neisseria meningitidis. *J Infect Dis*. Feb 1974;129(2):201-4. doi:10.1093/infdis/129.2.201
490. Evans-Jones LG, Whittle HC, Onyewotu, II, Egler LJ, Greenwood BM. Comparative study of group A and group C meningococcal infection. *Arch Dis Child*. Apr 1977;52(4):320-3. doi:10.1136/adc.52.4.320
491. Andersen BM. Mortality in meningococcal infections. *Scand J Infect Dis*. 1978;10(4):277-82. doi:10.3109/inf.1978.10.issue-4.04
492. Ellsworth J, Marks MI, Vose A. Meningococcal meningitis in children. *Can Med Assoc J*. Jan 20 1979;120(2):155-8.
493. Feldman RA, Mohs E, Faingezicht I, Lizano C, Barboza O. [Meningococcal meningitis in Costa Rica, 1970--1973. Epidemiological study]. *Bol Med Hosp Infant Mex*. Mar-Apr 1979;36(2):279-86. Meningitis meningococica en Costa Rica, 1970--1973 Estudio epidemiologico.
494. Olcen P, Barr J, Kjellander J. Meningitis and bacteremia due to Neisseria meningitidis: clinical and laboratory findings in 69 cases from Orebro county, 1965 to 1977. *Scand J Infect Dis*. 1979;11(2):111-9. doi:10.3109/inf.1979.11.issue-2.03
495. Ambrosch F, Stanek G. [Epidemiology of meningococcal meningitis in Austria]. *Wien Med Wochenschr*. Mar 15 1980;130(5):177-83. Die Epidemiologie der Meningokokken-Meningitis in Osterreich.
496. Conner WT, Minielly JA. Cerebral oedema in fatal meningococcaemia. *Lancet*. Nov 1 1980;2(8201):967-9. doi:10.1016/s0140-6736(80)92119-4
497. Binkin N, Band J. Epidemic of meningococcal meningitis in Bamako, Mali: epidemiological features and analysis of vaccine efficacy. *Lancet*. Aug 7 1982;2(8293):315-8. doi:10.1016/s0140-6736(82)90282-3
498. Hansman D. Meningococcal disease in South Australia: incidence and serogroup distribution 1971-1980. *J Hyg (Lond)*. Feb 1983;90(1):49-54. doi:10.1017/s0022172400063828

499. De Wals P, Hertoghe L, Reginster G, Borlee I, Bouckaert A, Dachy A, Lechat MF. Mortality in meningococcal disease in Belgium. *J Infect*. May 1984;8(3):264-73. doi:10.1016/s0163-4453(84)94123-9
500. Fallon RJ, Brown WM, Lore W. Meningococcal infections in Scotland 1972-82. *J Hyg (Lond)*. Oct 1984;93(2):167-80. doi:10.1017/s0022172400064688
501. Mohammed I, Obineche EN, Onyemelukwe GC, Zaruba K. Control of epidemic meningococcal meningitis by mass vaccination. I. Further epidemiological evaluation of groups A and C vaccines in northern Nigeria. *J Infect*. Sep 1984;9(2):190-6. doi:10.1016/s0163-4453(84)91429-4
502. Report from the PHLS Communicable Disease Surveillance Centre. *Br Med J (Clin Res Ed)*. May 31 1986;292(6533):1447-8. doi:10.1136/bmj.292.6533.1447
503. Spanjaard L, Bol P, de Marie S, Zanen HC. Association of meningococcal serogroups with the course of disease in the Netherlands, 1959-83. *Bull World Health Organ*. 1987;65(6):861-8.
504. Valmari P, Kataja M, Peltola H. Invasive haemophilus influenzae and meningococcal infections in Finland. A climatic, epidemiologic and clinical approach. *Scand J Infect Dis*. 1987;19(1):19-27. doi:10.3109/00365548709032373
505. Annapurna ME, Bhave GG, Mathur M. An outbreak of meningitis caused by Neisseria meningitidis Group A. *J Commun Dis*. Mar 1989;21(1):24-6.
506. Halstensen A, Sjursen H, Vollset SE, Froholm LO, Naess A, Matre R, Solberg CO. Serum opsonins to serogroup B meningococci in meningococcal disease. *Scand J Infect Dis*. 1989;21(3):267-76. doi:10.3109/00365548909035696
507. Salih MA, Ahmed HS, Osman KA, Kamil I, Palmgren H, Hofvander Y, Olcén P. Clinical features and complications of epidemic group A meningococcal disease in Sudanese children. *Ann Trop Paediatr*. 1990;10(3):231-8. doi:10.1080/02724936.1990.11747436
508. Tesoro LJ, Selbst SM. Factors affecting outcome in meningococcal infections. *Am J Dis Child*. Feb 1991;145(2):218-20. doi:10.1001/archpedi.1991.02160020112029
509. Fakhir S, Ahmad SH, Ahmad P. Prognostic factors influencing mortality in meningococcal meningitis. *Ann Trop Paediatr*. 1992;12(2):149-54. doi:10.1080/02724936.1992.11747560
510. Fekade D, Zawde D. Epidemic meningococcal meningitis in adult Ethiopians in Addis Abeba, Ethiopia, 1988. *Ethiop Med J*. Jul 1992;30(3):135-42.
511. Palmer SR, Corson J, Hall R, et al. Meningococcal disease in Wales: clinical features, outcome and public health management. *J Infect*. Nov 1992;25(3):321-8. doi:10.1016/0163-4453(92)91699-c
512. Kristos TG, Muhe L. Epidemic meningococcal meningitis in children. A retrospective analysis of cases admitted to ESCH (1988). *Ethiop Med J*. Jan 1993;31(1):9-14.
513. Patel MS, Merianos A, Hanna JN, Vartto K, Tait P, Morey F, Jayathissa S. Epidemic meningococcal meningitis in central Australia, 1987-1991. *Med J Aust*. Mar 1 1993;158(5):336-40. doi:10.5694/j.1326-5377.1993.tb121793.x
514. WHO. Control of a cerebrospinal meningitis epidemic. *Wkly Epidemiol Rec*. Aug 13 1993;68(33):237-8.
515. Scholten RJ, Bijlmer HA, Valkenburg HA, Dankert J. Patient and strain characteristics in relation to the outcome of meningococcal disease: a multivariate analysis. *Epidemiol Infect*. Feb 1994;112(1):115-24. doi:10.1017/s0950268800057472
516. Alhan E, Bozdemir N, Yuksel B, Onenli N, Kocabas E, Aksaray N. Epidemiology of meningococcal infections in children in mid-southern part of Turkey. *Eur J Epidemiol*. Aug 1995;11(4):393-6. doi:10.1007/BF01721223
517. Flaegstad T, Kaaresen PI, Stokland T, Gutteberg T. Factors associated with fatal outcome in childhood meningococcal disease. *Acta Paediatr*. Oct 1995;84(10):1137-42. doi:10.1111/j.1651-2227.1995.tb13513.x
518. Riordan FA, Marzouk O, Thomson AP, Sills JA, Hart CA. The changing presentations of meningococcal disease. *Eur J Pediatr*. Jun 1995;154(6):472-4. doi:10.1007/BF02029358
519. Munro R, Kociuba K, Jelfs J, Brown J, Crone S, Chant K. Meningococcal disease in urban south western Sydney, 1990-1994. *Aust N Z J Med*. Aug 1996;26(4):526-32. doi:10.1111/j.1445-5994.1996.tb00599.x
520. Semba RD, Bulterys M, Munyeshuli V, Gatsinzi T, Saah A, Chao A, Dushimimana A. Vitamin A deficiency and T-cell subpopulations in children with meningococcal disease. *J Trop Pediatr*. Oct 1996;42(5):287-90. doi:10.1093/tropej/42.5.287
521. Aplogan A, Batchassi E, Yakoua Y, et al. [An epidemic of meningococcal meningitis in the region of Savanes in Togo in 1997: research and control strategies]. *Sante*. Nov-Dec 1997;7(6):384-90. Une epidemie de meningite a meningocoque dans la region des Savanes au Togo en 1997: investigation et strategies de controle.
522. Juncal AR, Perez del Molino ML, Rodriguez I, Cid A, Guillan B, Pardo F. [Clinical and epidemiologic study of meningococcal meningitis in the health region of Santiago de Compostela (1990-1997)]. *Enferm Infecc Microbiol Clin*. Dec 1997;15(10):510-4. Estudio clinico y epidemiologico de las meningitis meningococicas en el area sanitaria de Santiago de Compostela (1990-1997).
523. Luaces Cubells C, Garcia Garcia JJ, Roca Martinez J, Latorre Otin CL. Clinical data in children with meningococcal meningitis in a Spanish hospital. *Acta Paediatr*. Jan 1997;86(1):26-9. doi:10.1111/j.1651-2227.1997.tb08826.x

524. Ndihokubwayo JB, Nyongabo T, Nkurikiye S, Adegbinni R. [A new epidemic of meningococcal meningitis in Burundi]. *Med Trop (Mars)*. 1997;57(1):98-9. Nouvelle epidemie de meningites a meningocoque au Burundi.
525. Heyman SN, Ginosar Y, Niel L, Amir J, Marx N, Shapiro M, Maayan S. Meningococcal meningitis among Rwandan refugees: diagnosis, management, and outcome in a field hospital. *Int J Infect Dis*. Jan-Mar 1998;2(3):137-42. doi:10.1016/s1201-9712(98)90115-1
526. Tapsall J, Programme" TAMS. Annual report of the Australian Meningococcal Surveillance Programme, 1998. The Australian Meningococcal Surveillance Programme. *Commun Dis Intell*. Nov 25 1999;23(12):317-23.
527. Pancharoen C, Hongsiriwon S, Swasdichai K, et al. Epidemiology of invasive meningococcal disease in 13 government hospitals in Thailand, 1994-1999. *Southeast Asian J Trop Med Public Health*. Dec 2000;31(4):708-11.
528. Seydi M, Soumare M, Sow AI, et al. [Clinical, bacteriological and therapeutic aspects of meningococcal meningitis in Dakar in 1999]. *Med Trop (Mars)*. 2002;62(2):137-40. Aspects cliniques, bacteriologiques et therapeutiques des meningites cerebro-spinales a Dakar en 1999.
529. Karima TM, Bukhari SZ, Fatani MI, Yasin KA, Al-Afif KA, Hafiz FH. Clinical and microbiological spectrum of meningococcal disease in adults during Hajj 2000: an implication of quadrivalent vaccination policy. *J Pak Med Assoc*. Jan 2003;53(1):3-7.
530. Mengistu G, Mitiku K, Teferi W. Analysis and reporting of meningococcal meningitis epidemic in north Gondar 2001-2002. *Ethiop Med J*. Oct 2003;41(4):319-31.
531. Dominguez A, Cardenosa N, Panella H, et al. The case-fatality rate of meningococcal disease in Catalonia, 1990-1997. *Scand J Infect Dis*. 2004;36(4):274-9. doi:10.1080/00365540410020163
532. Tapsall J, Programme" TAMS. Annual report of the Australian Meningococcal Surveillance Programme, 2003. *Commun Dis Intell Q Rep*. 2004;28(2):194-206.
533. Nathan N, Borel T, Djibo A, et al. Ceftriaxone as effective as long-acting chloramphenicol in short-course treatment of meningococcal meningitis during epidemics: a randomised non-inferiority study. *Lancet*. Jul 23-29 2005;366(9482):308-13. doi:10.1016/S0140-6736(05)66792-X
534. Sanou I, Ouedrago-Traore R, Ki-Zerbo GA, et al. [W135 meningococcus meningitis: study of 148 cases observed in 2002 and 2003 at the National Teaching Hospital of Ouagadougou, Burkina Faso]. *Med Trop (Mars)*. Apr 2006;66(2):137-42. Meningites a meningocoque du serogroupe W135: etude de 148 cas observes en 2002 et en 2003 au CHU-YO de Ouagadougou, Burkina Faso.
535. Tapsall J, Programme" TAMS. Annual report of the Australian Meningococcal Surveillance Programme, 2006. *Commun Dis Intell Q Rep*. Jun 2007;31(2):185-94.
536. Dash N, Ameen AS, Sheek-Hussein MM, Smego RA, Jr. Epidemiology of meningitis in Al-Ain, United Arab Emirates, 2000-2005. *Int J Infect Dis*. Jul 2007;11(4):309-12. doi:10.1016/j.ijid.2006.05.009
537. Gryniewicz O, Kolbusz J, Rosinska M, Zielinski A, Stefanoff P. Epidemiology of meningococcal meningitis and changes in the surveillance system in Poland, 1970-2006. *Euro Surveill*. May 1 2007;12(5):E7-8. doi:10.2807/esm.12.05.00707-en
538. Howitz M, Lambertsen L, Simonsen JB, Christensen JJ, Molbak K. Morbidity, mortality and spatial distribution of meningococcal disease, 1974-2007. *Epidemiol Infect*. Nov 2009;137(11):1631-40. doi:10.1017/S0950268809002428
539. Jhamb U, Chawla V, Khanna S. Clinical profile of group A meningococcal outbreak in Delhi. *Indian Pediatr*. Sep 2009;46(9):794-6.
540. Mutonga DM, Pimentel G, Muindi J, et al. Epidemiology and risk factors for serogroup X meningococcal meningitis during an outbreak in western Kenya, 2005-2006. *Am J Trop Med Hyg*. Apr 2009;80(4):619-24.
541. Weiss D, Stern EJ, Zimmerman C, et al. Epidemiologic investigation and targeted vaccination initiative in response to an outbreak of meningococcal disease among illicit drug users in Brooklyn, New York. *Clin Infect Dis*. Apr 1 2009;48(7):894-901. doi:10.1086/597257
542. Cohen C, Singh E, Wu HM, et al. Increased incidence of meningococcal disease in HIV-infected individuals associated with higher case-fatality ratios in South Africa. *AIDS*. Jun 1 2010;24(9):1351-60. doi:10.1097/QAD.0b013e32833a2520
543. Tolaj I, Dreshaj S, Qehaja E, Tolaj J, Doda-Ejupi T, Mehmeti M. Dexamethasone as adjuvant therapy in the treatment of invasive meningococcal diseases. *Med Arh*. 2010;64(4):228-30.
544. Gil-Prieto R, Garcia-Garcia L, Alvaro-Meca A, Gonzalez-Escalada A, Viguera Ester P, Gil De Miguel A. The burden of hospitalizations for meningococcal infection in Spain (1997-2008). *Vaccine*. Aug 5 2011;29(34):5765-70. doi:10.1016/j.vaccine.2011.05.089
545. Steindl G, Liu YL, Schmid D, Orendi U, Kormann-Klement A, Heuberger S. Epidemiology of invasive meningococcal disease in Austria 2010. *Wien Klin Wochenschr*. Oct 2011;123 Suppl 1:10-4. doi:10.1007/s00508-011-0058-0
546. Yameogo TM, Kyelem CG, Poda GE, Sombie I, Ouedraogo MS, Millogo A. [Meningitis epidemic: assessment of surveillance and treatment of cases in the health centers of a Burkina Faso district]. *Bull Soc Pathol Exot*. Feb

2011;104(1):68-73. Epidemie de meningite: evaluation de la surveillance et du traitement des cas dans les formations sanitaires d'un district du Burkina Faso. doi:10.1007/s13149-010-0123-3
547. Levy C, Taha MK, Bingen E, Cohen R, Groupe des Pediatres et Microbiologistes de l'Observatoire National des Meningites Bacteriennes de lE. [Paediatric meningococcal meningitis in France: ACTIV/GPIP network results]. *Arch Pediatr*. Sep 2012;19 Suppl 2:S49-54. Meningites a meningocoques de l'enfant en France: resultats de l'observatoire ACTIV/GPIP. doi:10.1016/S0929-693X(12)71273-3
548. Xu XH, Ye Y, Hu LF, Jin YH, Jiang QQ, Li JB. Emergence of serogroup C meningococcal disease associated with a high mortality rate in Hefei, China. *BMC Infect Dis*. Sep 4 2012;12:205. doi:10.1186/1471-2334-12-205
549. Dass Hazarika R, Deka NM, Khyriem AB, et al. Invasive meningococcal infection: analysis of 110 cases from a tertiary care centre in North East India. *Indian J Pediatr*. May 2013;80(5):359-64. doi:10.1007/s12098-012-0855-0
550. Stein-Zamir C, Shoob H, Sokolov I, Kunbar A, Abramson N, Zimmerman D. The clinical features and long-term sequelae of invasive meningococcal disease in children. *Pediatr Infect Dis J*. Jul 2014;33(7):777-9. doi:10.1097/INF.0000000000000282
551. Osuorah D, Shah B, Manjang A, Secka E, Ekwochi U, Ebenebe J. Outbreak of serotype W135 Neisseria meningitidis in central river region of the Gambia between February and June 2012: a hospital-based review of paediatric cases. *Niger J Clin Pract*. Jan-Feb 2015;18(1):41-7. doi:10.4103/1119-3077.146977
552. Sadarangani M, Scheifele DW, Halperin SA, Vaudry W, Le Saux N, Tsang R, Bettinger JA. Outcomes of invasive meningococcal disease in adults and children in Canada between 2002 and 2011: a prospective cohort study. *Clin Infect Dis*. Apr 15 2015;60(8):e27-35. doi:10.1093/cid/civ028
553. Stoof SP, Rodenburg GD, Knol MJ, et al. Disease Burden of Invasive Meningococcal Disease in the Netherlands Between June 1999 and June 2011: A Subjective Role for Serogroup and Clonal Complex. *Clin Infect Dis*. Oct 15 2015;61(8):1281-92. doi:10.1093/cid/civ506
554. Bassey BE, Vaz RG, Gasasira AN, et al. Pattern of the meningococcal meningitis outbreak in Northern Nigeria, 2009. *Int J Infect Dis*. Feb 2016;43:62-67. doi:10.1016/j.ijid.2015.12.016
555. Strelow VL, Miranda EJ, Kolbe KR, Framil JV, Oliveira AP, Vidal JE. Meningococcal meningitis: clinical and laboratorial characteristics, fatality rate and variables associated with in-hospital mortality. *Arq Neuropsiquiatr*. Nov 2016;74(11):875-880. doi:10.1590/0004-282X20160143
556. Patel JC, George J, Vuong J, et al. Rapid Laboratory Identification of Neisseria meningitidis Serogroup C as the Cause of an Outbreak - Liberia, 2017. *MMWR Morb Mortal Wkly Rep*. Oct 27 2017;66(42):1144-1147. doi:10.15585/mmwr.mm6642a5
557. Sall O, Stenmark B, Glimaker M, Jacobsson S, Molling P, Olcen P, Fredlund H. Clinical presentation of invasive disease caused by Neisseria meningitidis serogroup Y in Sweden, 1995 to 2012. *Epidemiol Infect*. Jul 2017;145(10):2137-2143. doi:10.1017/S0950268817000929
558. Menichetti F, Fortunato S, Ricci A, et al. Invasive Meningococcal Disease due to group C N. meningitidis ST11 (cc11): The Tuscany cluster 2015-2016. *Vaccine*. Sep 25 2018;36(40):5962-5966. doi:10.1016/j.vaccine.2018.08.050
559. Schulkind ML, Altemeier WA, 3rd, Ayoub EM. A comparison of ampicillin and chloramphenicol therapy in Hemophilus influenzae meningitis. *Pediatrics*. Sep 1971;48(3):411-6.
560. Barrett FF, Taber LH, Morris CR, Stephenson WB, Clark DJ, Yow MD. A 12 year review of the antibiotic management of Hemophilus influenzae meningitis. Comparison of ampicillin and conventional therapy including chloramphenicol. *J Pediatr*. Aug 1972;81(2):370-7. doi:10.1016/s0022-3476(72)80316-0
561. Parke JC, Jr., Schneerson R, Robbins JB. The attack rate, age incidence, racial distribution, and case fatality rate of Hemophilus influenzae type b meningitis in Mecklenbury County, North Carolina. *J Pediatr*. Oct 1972;81(4):765-9. doi:10.1016/s0022-3476(72)80099-4
562. Herrera Labarca P, Prenzel Leupolt I, Garcia Henriquez I. [Severe infections by Haemophilus influenzae in children]. *Bol Med Hosp Infant Mex*. May-Jun 1977;34(3):661-8. Infecciones graves por Haemophilus influenzae en ninos.
563. Koskiniemi M, Pettay O, Raivio M, Sarna S. Haemophilus influenzae meningitis. A comparison between chloramphenicol and ampicillin therapy with special reference to impaired hearing. *Acta Paediatr Scand*. Jan 1978;67(1):17-24. doi:10.1111/j.1651-2227.1978.tb16271.x
564. Koo W, Oley C, Munro R, Tomlinson P. Systemic Haemophilus influenzae infection in childhood. *Med J Aust*. Jul 24 1982;2(2):77-80. doi:10.5694/j.1326-5377.1982.tb124233.x
565. Broughton SJ, Warren RE. A review of Haemophilus influenzae infections in Cambridge 1975-1981. *J Infect*. Jul 1984;9(1):30-42. doi:10.1016/s0163-4453(84)94446-3
566. Istre GR, Conner JS, Glode MP, Hopkins RS. Increasing ampicillin-resistance rates in Hemophilus influenzae meningitis. *Am J Dis Child*. Apr 1984;138(4):366-9. doi:10.1001/archpedi.1984.02140420032012
567. Nottidge VA. Haemophilus influenzae meningitis: a 5-year study in Ibadan, Nigeria. *J Infect*. Sep 1985;11(2):109-17. doi:10.1016/s0163-4453(85)91931-0

568. Campos J, Garcia-Tornel S, Gairi JM, Fabregues I. Multiply resistant Haemophilus influenzae type b causing meningitis: comparative clinical and laboratory study. *J Pediatr*. Jun 1986;108(6):897-902. doi:10.1016/s0022-3476(86)80923-4
569. Dyas A, George RH. Ten years' experience of Haemophilus influenzae infection at Birmingham Children's Hospital. *J Infect*. Sep 1986;13(2):179-85. doi:10.1016/s0163-4453(86)93093-8
570. Nesheim SR, Wilcox WD. Systemic Hemophilus influenzae disease in children. A 10-year retrospective study of an urban hospital population. *Clin Pediatr (Phila)*. Dec 1986;25(12):605-9. doi:10.1177/000992288602501203
571. Taft TA, Chusid MJ, Sty JR. Cerebral infarction in Hemophilus influenzae type B meningitis. *Clin Pediatr (Phila)*. Apr 1986;25(4):177-80. doi:10.1177/000992288602500401
572. Ward JI, Lum MK, Hall DB, Silimperi DR, Bender TR. Invasive Haemophilus influenzae type b disease in Alaska: background epidemiology for a vaccine efficacy trial. *J Infect Dis*. Jan 1986;153(1):17-26. doi:10.1093/infdis/153.1.17
573. Trollfors B, Claesson BA, Strangert K, Taranger J. Haemophilus influenzae meningitis in Sweden 1981-1983. *Arch Dis Child*. Dec 1987;62(12):1220-3. doi:10.1136/adc.62.12.1220
574. Cordtz T, Jepsen OB, Arpi M, Honberg P. Antibiotic therapy of meningitis caused by ampicillin-resistant Haemophilus influenzae in Denmark and Greenland 1981 to 1987. *Eur J Clin Microbiol Infect Dis*. Oct 1988;7(5):646-50. doi:10.1007/BF01964243
575. Kaplan SL, Mason SK, Mason EO, Jr., Murphy M, Smith EO. Follow-up of prospective randomized trial of ampicillin or chloramphenicol versus moxalactam treatment of Haemophilus influenzae type b meningitis. *J Pediatr*. May 1988;112(5):795-8. doi:10.1016/s0022-3476(88)80707-8
576. Tudor-Williams G, Frankland J, Isaacs D, et al. Haemophilus influenzae type b disease in the Oxford region. *Arch Dis Child*. Apr 1989;64(4):517-9. doi:10.1136/adc.64.4.517
577. Ferreccio C, Ortiz E, Astroza L, Rivera C, Clemens J, Levine MM. A population-based retrospective assessment of the disease burden resulting from invasive Haemophilus influenzae in infants and young children in Santiago, Chile. *Pediatr Infect Dis J*. Jul 1990;9(7):488-94. doi:10.1097/00006454-199007000-00007
578. Peltola H, Rod TO, Jonsdottir K, Bottiger M, Coolidge JA. Life-threatening Haemophilus influenzae infections in Scandinavia: a five-country analysis of the incidence and the main clinical and bacteriologic characteristics. *Rev Infect Dis*. Jul-Aug 1990;12(4):708-15. doi:10.1093/clinids/12.4.708
579. Bijlmer HA, van Alphen L. A prospective, population-based study of Haemophilus influenzae type b meningitis in The Gambia and the possible consequences. *J Infect Dis*. Jun 1992;165 Suppl 1:S29-32. doi:10.1093/infdis/165-supplement_1-s29
580. Murphy TV, Granoff DM, Pierson LM, Pastor P, White KE, Clements JF, Osterholm MT. Invasive Haemophilus influenzae type b disease in children less than 5 years of age in Minnesota and in Dallas County, Texas, 1983-1984. *J Infect Dis*. Jun 1992;165 Suppl 1:S7-10. doi:10.1093/infdis/165-supplement_1-s7
581. Gervaix A, Suter S. Need for prevention of invasive Haemophilus influenzae type b infections in Geneva, Switzerland. *Vaccine*. 1993;11 Suppl 1:S34-7. doi:10.1016/0264-410x(93)90157-s
582. McIntyre P, Jepson R, Leeder S, Irwig L. The outcome of childhood Haemophilus influenzae meningitis. A population based study. *Med J Aust*. Dec 6-20 1993;159(11-12):766-72.
583. Reinert P, Liwartowski A, Dabernat H, Guyot C, Boucher J, Carrere C. Epidemiology of Haemophilus influenzae type b disease in France. *Vaccine*. 1993;11 Suppl 1:S38-42. doi:10.1016/0264-410x(93)90158-t
584. Spencker FB, Wasser S, Rieske K, Springer W, Handrick W. [Haemophilus influenzae meningitis in childhood]. *Padiatr Grenzgeb*. 1993;31(4):207-18. Haemophilus-influenzae-Meningitis im Kindesalter.
585. Likitnukul S. Systemic Haemophilus influenzae disease in Thai children. *Southeast Asian J Trop Med Public Health*. Dec 1994;25(4):672-7.
586. Rauter L, Mutz I. [Haemophilus influenzae meningitis 1983 to 1992--epidemiology and sequelae of the disease]. *Wien Klin Wochenschr*. 1994;106(7):187-92. Haemophilus influenzae-Meningitis der Jahre 1983 bis 1992--Epidemiologie und Folgen der Erkrankung.
587. Dabernat H, Scheimberg A, Astruc J. Analysis of oral antibiotic treatment that failed to prevent the development of Haemophilus influenzae meningitis: consequences on mortality. *J Antimicrob Chemother*. Oct 1996;38(4):679-89. doi:10.1093/jac/38.4.679
588. Limcangco MR, Salole EG, Armour CL. Epidemiology of Haemophilus influenzae type b meningitis in Manila, Philippines, 1994 to 1996. *Pediatr Infect Dis J*. Jan 2000;19(1):7-11. doi:10.1097/00006454-200001000-00003
589. Steinhoff MC, Thomas K, Lalitha MK, Network" IBISGotICE. Are Haemophilus influenzae infections a significant problem in India? A prospective study and review. *Clin Infect Dis*. Apr 1 2002;34(7):949-57. doi:10.1086/339327
590. Kim JS, Jang YT, Kim JD, et al. Incidence of Haemophilus influenzae type b and other invasive diseases in South Korean children. *Vaccine*. Sep 28 2004;22(29-30):3952-62. doi:10.1016/j.vaccine.2004.04.003

591. Yaro S, Lourd M, Naccro B, et al. The epidemiology of Haemophilus influenzae type b meningitis in Burkina Faso. *Pediatr Infect Dis J*. May 2006;25(5):415-9. doi:10.1097/01.inf.0000217371.38080.8a
592. Miranzi Sde S, de Moraes SA, de Freitas IC. Impact of the Haemophilus influenzae type b vaccination program on HIB meningitis in Brazil. *Cad Saude Publica*. Jul 2007;23(7):1689-95. doi:10.1590/s0102-311x2007000700021
593. Ribeiro GS, Lima JB, Reis JN, et al. Haemophilus influenzae meningitis 5 years after introduction of the Haemophilus influenzae type b conjugate vaccine in Brazil. *Vaccine*. May 30 2007;25(22):4420-8. doi:10.1016/j.vaccine.2007.03.024
594. Thoon KC, Chong CY, Ng WY, Kilgore PE, Nyambat B. Epidemiology of invasive Haemophilus influenzae type b disease in Singapore children, 1994-2003. *Vaccine*. Aug 29 2007;25(35):6482-9. doi:10.1016/j.vaccine.2007.06.037
595. Lee EH, Lewis RF, Makumbi I, et al. Haemophilus influenzae type b conjugate vaccine is highly effective in the Ugandan routine immunization program: a case-control study. *Trop Med Int Health*. Apr 2008;13(4):495-502. doi:10.1111/j.1365-3156.2008.02027.x
596. Rahman M, Hossain S, Baqui AH, et al. Haemophilus influenzae type-b and non-b-type invasive diseases in urban children (<5years) of Bangladesh: implications for therapy and vaccination. *J Infect*. Mar 2008;56(3):191-6. doi:10.1016/j.jinf.2007.12.008
597. Nyambat B, Dang DA, Nguyen HA, Mai TQ, Rani M, Slack MP, Kilgore PE. Rapid assessment of Hib disease burden in Vietnam. *BMC Public Health*. Apr 25 2011;11:260. doi:10.1186/1471-2458-11-260
598. Rubach MP, Bender JM, Mottice S, et al. Increasing incidence of invasive Haemophilus influenzae disease in adults, Utah, USA. *Emerg Infect Dis*. Sep 2011;17(9):1645-50. doi:10.3201/eid1709.101991
599. Ubukata K, Chiba N, Morozumi M, Iwata S, Sunakawa K, Working Group of Nationwide Surveillance for Bacterial M. Longitudinal surveillance of Haemophilus influenzae isolates from pediatric patients with meningitis throughout Japan, 2000-2011. *J Infect Chemother*. Feb 2013;19(1):34-41. doi:10.1007/s10156-012-0448-x
600. Bamberger EE, Ben-Shimol S, Abu Raya B, et al. Pediatric invasive Haemophilus influenzae infections in Israel in the era of Haemophilus influenzae type b vaccine: a nationwide prospective study. *Pediatr Infect Dis J*. May 2014;33(5):477-81. doi:10.1097/INF.0000000000000193
601. Lavetter A, Leedom JM, Mathies AW, Jr., Ivler D, Wehrle PF. Meningitis due to Listeria monocytogenes. A review of 25 cases. *N Engl J Med*. Sep 9 1971;285(11):598-603. doi:10.1056/NEJM197109092851103
602. Bowmer EJ, McKiel JA, Cockcroft WH, Schmitt N, Rappay DE. Listeria monocytogenes infections in Canada. *Can Med Assoc J*. Jul 21 1973;109(2):125-9 passim.
603. Iwarson S, Larsson S. Outcome of Listeria monocytogenes infection in compromised and non-compromised adults; a comparative study of seventy-two cases. *Infection*. 1979;7(2):54-6. doi:10.1007/BF01641612
604. Cherubin CE, Marr JS, Sierra MF, Becker S. Listeria and gram-negative bacillary meningitis in New York City, 1972-1979. Frequent causes of meningitis in adults. *Am J Med*. Aug 1981;71(2):199-209. doi:10.1016/0002-9343(81)90106-6
605. Nau R, Schuchardt V, Prange HW. [Listeriosis of the central nervous system]. *Fortschr Neurol Psychiatr*. Nov 1990;58(11):408-22. Zur Listeriose des Zentralnervensystems. doi:10.1055/s-2007-1001204
606. Bula CJ, Bille J, Glauser MP. An epidemic of food-borne listeriosis in western Switzerland: description of 57 cases involving adults. *Clin Infect Dis*. Jan 1995;20(1):66-72. doi:10.1093/clinids/20.1.66
607. Mylonakis E, Hohmann EL, Calderwood SB. Central nervous system infection with Listeria monocytogenes. 33 years' experience at a general hospital and review of 776 episodes from the literature. *Medicine (Baltimore)*. Sep 1998;77(5):313-36. doi:10.1097/00005792-199809000-00002
608. Amaya-Villar R, Garcia-Cabrera E, Sulleiro-Igual E, et al. Three-year multicenter surveillance of community-acquired Listeria monocytogenes meningitis in adults. *BMC Infect Dis*. Nov 11 2010;10:324. doi:10.1186/1471-2334-10-324
609. Roed C, Engsig FN, Omland LH, Skinhoj P, Obel N. Long-term mortality in patients diagnosed with Listeria monocytogenes meningitis: a Danish nationwide cohort study. *J Infect*. Jan 2012;64(1):34-40. doi:10.1016/j.jinf.2011.10.003
610. Pelegrin I, Moragas M, Suarez C, et al. Listeria monocytogenes meningoencephalitis in adults: analysis of factors related to unfavourable outcome. *Infection*. Oct 2014;42(5):817-27. doi:10.1007/s15010-014-0636-y
611. Arslan F, Meynet E, Sunbul M, et al. The clinical features, diagnosis, treatment, and prognosis of neuroinvasive listeriosis: a multinational study. *Eur J Clin Microbiol Infect Dis*. Jun 2015;34(6):1213-21. doi:10.1007/s10096-015-2346-5
612. Thonnings S, Knudsen JD, Schonheyder HC, et al. Antibiotic treatment and mortality in patients with Listeria monocytogenes meningitis or bacteraemia. *Clin Microbiol Infect*. Aug 2016;22(8):725-30. doi:10.1016/j.cmi.2016.06.006
613. Koopmans MM, Bijlsma MW, Brouwer MC, van de Beek D, van der Ende A. Listeria monocytogenes meningitis in the Netherlands, 1985-2014: A nationwide surveillance study. *J Infect*. Jul 2017;75(1):12-19. doi:10.1016/j.jinf.2017.04.004
614. McCracken GH, Jr., Sarff LD, Glode MP, et al. Relation between Escherichia coli K1 capsular polysaccharide antigen and clinical outcome in neonatal meningitis. *Lancet*. Aug 3 1974;2(7875):246-50. doi:10.1016/s0140-6736(74)91413-5

615. Heckmatt JZ. Coliform meningitis in the newborn. *Arch Dis Child*. Aug 1976;51(8):569-75. doi:10.1136/adc.51.8.569
616. Houdouin V, Bonacorsi S, Bidet P, et al. Association between mortality of Escherichia coli meningitis in young infants and non-virulent clonal groups of strains. *Clin Microbiol Infect*. Jul 2008;14(7):685-90. doi:10.1111/j.1469-0691.2008.02019.x
617. Basmaci R, Bonacorsi S, Bidet P, et al. Escherichia Coli Meningitis Features in 325 Children From 2001 to 2013 in France. *Clin Infect Dis*. Sep 1 2015;61(5):779-86. doi:10.1093/cid/civ367
618. Tauzin M, Ouldali N, Levy C, Bechet S, Cohen R, Caeymaex L. Combination therapy with ciprofloxacin and third-generation cephalosporin versus third-generation cephalosporin monotherapy in Escherichia coli meningitis in infants: a multicentre propensity score-matched observational study. *Clin Microbiol Infect*. Aug 2019;25(8):1006-1012. doi:10.1016/j.cmi.2018.12.026
619. Yun KW, Park SH, Cho EY, Lee H. Clinical features and outcomes of community-acquired late-onset sepsis: Focusing on group B Streptococcus and Escherichia coli. *Pediatr Int*. Dec 2019;61(12):1210-1215. doi:10.1111/ped.14012
620. Haslam RH, Allen JR, Dorsen MM, Kanofsky DL, Mellitus ED, Norris DA. The sequelae of group B beta-hemolytic streptococcal meningitis in early infancy. *Am J Dis Child*. Aug 1977;131(8):845-9. doi:10.1001/archpedi.1977.02120210023003
621. Schrag SJ, Zywicki S, Farley MM, et al. Group B streptococcal disease in the era of intrapartum antibiotic prophylaxis. *N Engl J Med*. Jan 6 2000;342(1):15-20. doi:10.1056/NEJM200001063420103
622. Madhi SA, Radebe K, Crewe-Brown H, Frasch CE, Arakere G, Mokhachane M, Kimura A. High burden of invasive Streptococcus agalactiae disease in South African infants. *Ann Trop Paediatr*. Mar 2003;23(1):15-23. doi:10.1179/000349803125002814
623. Georget-Bouquinet E, Bingen E, Aujard Y, Levy C, Cohen R, Groupe des Pediatres et Microbiologistes de l'Observatoire National des Meningites Bacteriennes de lE. [Group B streptococcal meningitis'clinical, biological and evolutive features in children]. *Arch Pediatr*. Dec 2008;15 Suppl 3:S126-32. Caracteristiques cliniques, biologiques et evolutives des meningites a streptocoque du groupe B de l'enfant. doi:10.1016/S0929-693X(08)75495-2
624. Apostol M, Gershman K, Arnold K, et al. Trends in perinatal group B streptococcal disease - United States, 2000-2006. *MMWR Morb Mortal Wkly Rep*. Feb 13 2009;58(5):109-12.
625. Libster R, Edwards KM, Levent F, et al. Long-term outcomes of group B streptococcal meningitis. *Pediatrics*. Jul 2012;130(1):e8-15. doi:10.1542/peds.2011-3453
626. Joubrel C, Tazi A, Six A, et al. Group B streptococcus neonatal invasive infections, France 2007-2012. *Clin Microbiol Infect*. Oct 2015;21(10):910-6. doi:10.1016/j.cmi.2015.05.039
627. Bartlett AW, Smith B, George CR, McMullan B, Kesson A, Lahra MM, Palasanthiran P. Epidemiology of Late and Very Late Onset Group B Streptococcal Disease: Fifteen-Year Experience From Two Australian Tertiary Pediatric Facilities. *Pediatr Infect Dis J*. Jan 2017;36(1):20-24. doi:10.1097/INF.0000000000001345
628. Lo CW, Liu HC, Lee CC, Lin CL, Chen CL, Jeng MJ, Chiu CH. Serotype distribution and clinical correlation of Streptococcus agalactiae causing invasive disease in infants and children in Taiwan. *J Microbiol Immunol Infect*. Aug 2019;52(4):578-584. doi:10.1016/j.jmii.2017.09.002
629. van Kassel MN, Bijlsma MW, Brouwer MC, van der Ende A, van de Beek D. Community-acquired group B streptococcal meningitis in adults: 33 cases from prospective cohort studies. *J Infect*. Jan 2019;78(1):54-57. doi:10.1016/j.jinf.2018.07.009
630. Geteneh A, Kassa T, Alemu Y, et al. Enhanced identification of Group B streptococcus in infants with suspected meningitis in Ethiopia. *PLoS One*. 2020;15(11):e0242628. doi:10.1371/journal.pone.0242628
631. Nakwa FL, Lala SG, Madhi SA, Dangor Z. Neurodevelopmental Impairment at 1 Year of Age in Infants With Previous Invasive Group B Streptococcal Sepsis and Meningitis. *Pediatr Infect Dis J*. Sep 2020;39(9):794-798. doi:10.1097/INF.0000000000002695
