# Supplementary material for: Global Case Fatality of Bacterial Meningitis During an 80-Year Period: A Systematic Review and Meta-Analysis
Source: JAMA Netw Open. 2024 Aug 2;7(8):e2424802. doi: 10.1001/jamanetworkopen.2024.24802 (PMC11297475; doi:10.1001/jamanetworkopen.2024.24802)
Supplement: Supplement 2. — Data Sharing Statement [file jamanetwopen-e2424802-s002.pdf]

Data Sharing Statement

Data
Data available: Yes
Data types: Data (not involving human participants)
How to access data: https://github.com/fdlbern/mortbm
When available: With publication

Supporting Documents
Document types: Statistical/analytic code
How to access documents: https://github.com/fdlbern/mortbm
When available: With publication

Additional Information
Who can access the data: Anyone requesting the data
Types of analyses: For any purpose
Mechanisms of data availability: After approval without investigator support
Any additional restrictions: None
